# Twist exome capture allows for lower average sequence coverage in clinical exome sequencing

**DOI:** 10.1186/s40246-023-00485-5

**Published:** 2023-05-03

**Authors:** Burcu Yaldiz, Erdi Kucuk, Juliet Hampstead, Tom Hofste, Rolph Pfundt, Jordi Corominas Galbany, Tuula Rinne, Helger G. Yntema, Alexander Hoischen, Marcel Nelen, Christian Gilissen, Solve-RD consortium, Olaf Riess, Tobias B. Haack, Holm Graessner, Birte Zurek, Kornelia Ellwanger, Stephan Ossowski, German Demidov, Marc Sturm, Julia M. Schulze-Hentrich, Rebecca Schüle, Jishu Xu, Christoph Kessler, Melanie Wayand, Matthis Synofzik, Carlo Wilke, Andreas Traschütz, Ludger Schöls, Holger Hengel, Holger Lerche, Josua Kegele, Peter Heutink, Han Brunner, Hans Scheffer, Nicoline Hoogerbrugge, Alexander Hoischen, Peter A. C.’t Hoen, Lisenka E. L. M. Vissers, Christian Gilissen, Wouter Steyaert, Karolis Sablauskas, Richarda M. de Voer, Erik-Jan Kamsteeg, Bart van de Warrenburg, Nienke van Os, Iris te Paske, Erik Janssen, Elke de Boer, Marloes Steehouwer, Burcu Yaldiz, Tjitske Kleefstra, Anthony J. Brookes, Colin Veal, Spencer Gibson, Vatsalya Maddi, Mehdi Mehtarizadeh, Umar Riaz, Greg Warren, Farid Yavari Dizjikan, Thomas Shorter, Ana Töpf, Volker Straub, Chiara Marini Bettolo, Jordi Diaz Manera, Sophie Hambleton, Karin Engelhardt, Jill Clayton-Smith, Siddharth Banka, Elizabeth Alexander, Adam Jackson, Laurence Faivre, Christel Thauvin, Antonio Vitobello, Anne-Sophie Denommé-Pichon, Yannis Duffourd, Ange-Line Bruel, Christine Peyron, Aurore Pélissier, Sergi Beltran, Ivo Glynne Gut, Steven Laurie, Davide Piscia, Leslie Matalonga, Anastasios Papakonstantinou, Gemma Bullich, Alberto Corvo, Marcos Fernandez-Callejo, Carles Hernández, Daniel Picó, Ida Paramonov, Hanns Lochmüller, Gulcin Gumus, Virginie Bros-Facer, Ana Rath, Marc Hanauer, David Lagorce, Oscar Hongnat, Maroua Chahdil, Emeline Lebreton, Giovanni Stevanin, Alexandra Durr, Claire-Sophie Davoine, Léna Guillot-Noel, Anna Heinzmann, Giulia Coarelli, Gisèle Bonne, Teresinha Evangelista, Valérie Allamand, Isabelle Nelson, Rabah Ben Yaou, Corinne Metay, Bruno Eymard, Enzo Cohen, Antonio Atalaia, Tanya Stojkovic, Milan Macek, Marek Turnovec, Dana Thomasová, Radka Pourová Kremliková, Vera Franková, Markéta Havlovicová, Petra Lišková, Pavla Doležalová, Helen Parkinson, Thomas Keane, Mallory Freeberg, Coline Thomas, Dylan Spalding, Peter Robinson, Daniel Danis, Glenn Robert, Alessia Costa, Christine Patch, Mike Hanna, Henry Houlden, Mary Reilly, Jana Vandrovcova, Stephanie Efthymiou, Heba Morsy, Elisa Cali, Francesca Magrinelli, Sanjay M. Sisodiya, Jonathan Rohrer, Francesco Muntoni, Irina Zaharieva, Anna Sarkozy, Vincent Timmerman, Jonathan Baets, Geert de Vries, Jonathan De Winter, Danique Beijer, Peter de Jonghe, Liedewei Van de Vondel, Willem De Ridder, Sarah Weckhuysen, Vincenzo Nigro, Margherita Mutarelli, Manuela Morleo, Michele Pinelli, Alessandra Varavallo, Sandro Banfi, Annalaura Torella, Francesco Musacchia, Giulio Piluso, Alessandra Ferlini, Rita Selvatici, Francesca Gualandi, Stefania Bigoni, Rachele Rossi, Marcella Neri, Stefan Aretz, Isabel Spier, Anna Katharina Sommer, Sophia Peters, Carla Oliveira, Jose Garcia Pelaez, Ana Rita Matos, Celina São José, Marta Ferreira, Irene Gullo, Susana Fernandes, Luzia Garrido, Pedro Ferreira, Fátima Carneiro, Morris A. Swertz, Lennart Johansson, Joeri K. van der Velde, Gerben van der Vries, Pieter B. Neerincx, David Ruvolo, Kristin M. Abbott, Wilhemina SKerstjens Frederikse, Eveline Zonneveld-Huijssoon, Dieuwke Roelofs-Prins, Marielle van Gijn, Sebastian Köhler, Alison Metcalfe, Alain Verloes, Séverine Drunat, Delphine Heron, Cyril Mignot, Boris Keren, Jean-Madeleine de Sainte Agathe, Caroline Rooryck, Didier Lacombe, Aurelien Trimouille, Manuel Posada De la Paz, Eva Bermejo Sánchez, Estrella López Martín, Beatriz Martínez Delgado, F. Javier Alonso García de la Rosa, Andrea Ciolfi, Bruno Dallapiccola, Simone Pizzi, Francesca Clementina Radio, Marco Tartaglia, Alessandra Renieri, Simone Furini, Chiara Fallerini, Elisa Benetti, Peter Balicza, Maria Judit Molnar, Ales Maver, Borut Peterlin, Alexander Münchau, Katja Lohmann, Rebecca Herzog, Martje Pauly, Alfons Macaya, Ana Cazurro-Gutiérrez, Belén Pérez-Dueñas, Francina Munell, Clara Franco Jarava, Laura Batlle Masó, Anna Marcé-Grau, Roger Colobran, Andrés Nascimento Osorio, Daniel Natera de Benito, Hanns Lochmüller, Rachel Thompson, Kiran Polavarapu, Bodo Grimbacher, David Beeson, Judith Cossins, Peter Hackman, Mridul Johari, Marco Savarese, Bjarne Udd, Rita Horvath, Patrick F. Chinnery, Thiloka Ratnaike, Fei Gao, Katherine Schon, Gabriel Capella, Laura Valle, Elke Holinski-Feder, Andreas Laner, Verena Steinke-Lange, Evelin Schröck, Andreas Rump, Ayşe Nazlı Başak, Dimitri Hemelsoet, Bart Dermaut, Nika Schuermans, Bruce Poppe, Hannah Verdin, Davide Mei, Annalisa Vetro, Simona Balestrini, Renzo Guerrini, Kristl Claeys, Gijs W. E. Santen, Emilia K. Bijlsma, Mariette J. V. Hoffer, Claudia A. L. Ruivenkamp, Kaan Boztug, Matthias Haimel, Isabelle Maystadt, Isabelle Cordts, Marcus Deschauer, Ioannis Zaganas, Evgenia Kokosali, Mathioudakis Lambros, Athanasios Evangeliou, Martha Spilioti, Elisabeth Kapaki, Mara Bourbouli, Pasquale Striano, Federico Zara, Antonella Riva, Michele Iacomino, Paolo Uva, Marcello Scala, Paolo Scudieri, Maria-Roberta Cilio, Evelina Carpancea, Chantal Depondt, Damien Lederer, Yves Sznajer, Sarah Duerinckx, Sandrine Mary, Christel Depienne, Andreas Roos, Patrick May

**Affiliations:** 1 Department of Human Genetics, Radboud Institute for Molecular Life Sciences, Radboud University Medical Centre, Geert Grooteplein 10, 6525 GA Nijmegen, The Netherlands; 2 Department of Human Genetics, Donders Institute for Brain, Cognition and Behaviour, Radboud University Medical Centre, Geert Grooteplein 10, 6525 GA Nijmegen, The Netherlands

**Keywords:** Exome sequencing, Genome sequencing, Uniformity of coverage

## Abstract

**Background:**

Exome and genome sequencing are the predominant techniques in the diagnosis and research of genetic disorders. Sufficient, uniform and reproducible/consistent sequence coverage is a main determinant for the sensitivity to detect single-nucleotide (SNVs) and copy number variants (CNVs). Here we compared the ability to obtain comprehensive exome coverage for recent exome capture kits and genome sequencing techniques.

**Results:**

We compared three different widely used enrichment kits (Agilent SureSelect Human All Exon V5, Agilent SureSelect Human All Exon V7 and Twist Bioscience) as well as short-read and long-read WGS. We show that the Twist exome capture significantly improves complete coverage and coverage uniformity across coding regions compared to other exome capture kits. Twist performance is comparable to that of both short- and long-read whole genome sequencing. Additionally, we show that even at a reduced average coverage of 70× there is only minimal loss in sensitivity for SNV and CNV detection.

**Conclusion:**

We conclude that exome sequencing with Twist represents a significant improvement and could be performed at lower sequence coverage compared to other exome capture techniques.

**Supplementary Information:**

The online version contains supplementary material available at 10.1186/s40246-023-00485-5.

## Background

Next-generation sequencing (NGS) techniques are widely used across clinical and research applications in genetics. With the improvements in targeted sequencing approaches, whole exome sequencing (WES) has become a standard tool in clinical diagnostics [1–6].

There are various exome capture kits with different target enrichment strategies. Selection of target genomic regions, sequence features, length of probes and exome capture mechanisms are the major differences among these kits. These characteristics may give rise to differences in the overall coverage uniformity and capture efficiency of specific targets, resulting in decreased variant calling sensitivity. Several studies that compared exome capture technologies have shown that there are major differences in their performance [7–10] and that high average read depth does not guarantee coverage for individual targets. In these comparative studies, extreme GC content [11–13] and mappability issues [12, 14] are shown to be the major sources of coverage bias.

Sufficient, uniform and reproducible/consistent sequence coverage is required for robust and sensitive single-nucleotide variant (SNV) and copy number variant (CNV) detection in exome data. While CNVs are not routinely detected from WES in each laboratory or pipeline, their additional clinical utility [15–17] urges for reliable CNV detection from exomes, especially when patient cohorts are not routinely pre-screened by CNV-microarrays. CNV detection from WES data particularly fully depends on the analysis of read depth variations at sequencing targets. Large sets of reference samples are typically required in order to robustly compare CNV coverage profiles in exome data. Therefore, over- and underrepresentation of target regions due to extreme GC content and mappability issues can dramatically affect the robustness of CNV calling from exome data [15]. Short- and long-read whole genome sequencing (SR-WGS and LR-WGS, respectively) approaches generally yield more uniform and complete coverage profiles than exome sequencing, and the gapless nature of WGS data enables more accurate detection of CNVs and structural variants (SVs). However, lower sequencing and storage costs as well as the demonstration of diagnostic yield of CNV detection have led WES to be proposed as a first-tier diagnostic test in recent studies [18, 19].

In the last few years, new exome capture and sequencing technologies, particularly the Twist exome capture kit and long read sequencing (LRS) technologies, have been applied in clinical sequencing studies [20–22]. Here, we compared the Twist exome capture kit’s coding sequence coverage and SNV detection sensitivity to other widely used exome kits as well as to SR- and LR-WGS. As further benchmarks, we utilized the SR- and LR-WGS methods which are purported to provide optimal uniformity and coverage profiles [22]. We assessed the sensitivity of SNV and CNV calling of Twist exome capture kit at reduced average coverage levels.

## Methods

### Sample collection

#### Whole exome sequencing

Various studies have evaluated the effectiveness of established enrichment technologies such as Agilent SureSelect, Nimblegen SeqCap and Illumina TruSeq [8–10, 23–26]. These comparisons have shown relatively modest differences between the most recent versions of these technologies, mostly due to differences in target design. In this study, we investigated a completely novel capture method by Twist Bioscience (Twist). Twist uses a silicon-based DNA synthesis technology that allows for the production of larger quantities of oligonucleotides, resulting in more probes and improved rebalancing, which was expected to yield significant improvements in target coverage and coverage uniformity. We compared Twist exome capture to one of the latest Agilent SureSelect Human All Exon V7 (Agilent V7) which has been shown to perform on par with other commonly used exome capture technologies. In addition, we included an older version of the Agilent SureSelect Human All Exon V5 (Agilent V5) which has been widely used in the past to provide a point of reference. We collected 20 whole blood patient samples sequenced using each of the three kits randomly (Table 1; Additional file 4. These samples were downsampled to 100× as described below:

- Samples sequenced using the Agilent V5 enrichment kit with a mean coverage of 274.8×.
- Samples sequenced using the Agilent V7 with a mean coverage of 239.6×.
- Samples sequenced using the Twist enrichment kit with a mean coverage of 139.2×.

**Table 1:** Overview of samples used in this study

Enrichment/library	Average coverage	Coverage range (min–max)	Number of samples
Agilent V5	274.8	163.8–345.4	20
Agilent V7	239.6	131.1–370.6	20
Twist	139.2	119.7–158.5	20
WGS	59.3	50.86–69.33	20
LRS	29.4	24.24–38.86	18

Columns depict (from left to the right) the exome kits and the platforms; the average coverage across the target regions of the enrichment kits for the exomes; the range of coverage; the number of samples used in the analysis

In addition to these samples, 7 exome samples captured with Twist enrichment kit with lower average coverage of 69.95×, five exome samples collected from three different tissues [amniotic fluid, basal mucosa (buccal swap) and fibroblasts] captured by Twist enrichment kit were also used for further comparisons (Additional file 1: Table S1). Besides, 14 Twist samples with previously validated CNVs and an additional 100 Twist samples as a reference pool were used for performing CNV analysis (Additional file 1: Table S2). These additional samples were used as control samples for normalization of the read counts, and they were not involved in other comparisons.

All samples were sequenced on an Illumina NovaSeq 6000 sequencer using 2 × 150 paired-end sequencing. All exome samples were aligned by the Burrows Wheeler Aligner (BWA) [27] to the hg19/GRCh37 assembly of the human reference genome. Duplicates were marked as GATK best practices were followed during the mapping process.

#### Short-read whole genome sequencing

A total of 20 SR-WGS samples were sequenced using 2 × 150 bp paired-end on an Illumina NovaSeq 6000 sequencer to 59.3× mean coverage (Additional file 1: Table S1). Alignment was performed by using Burrows Wheel Aligner (BWA) [27] to the hg19/GRCh37 assembly of the human reference genome.

#### Long-read whole genome sequencing

We also sequenced 6 trios (18 samples) with a Pacific Biosciences Sequel II instrument. We used three SMRT chips per sample, targeting 30× mean coverage with HiFi reads (Additional file 1: Table S1). Reads were aligned to the hg19/GRCh37 assembly of the human reference genome with pbmm2 (version 1.4.0) using default parameters.

### Gene definitions

Genes and coding regions were defined using NCBI RefSeq (Release 61) [28] and EMBL-EBI Ensembl GENCODE (Release 91) [29] transcripts of the hg19/GRCh37 assembly of the human reference genome. Transcripts of both databases were downloaded from the UCSC Table Browser [30]. We generated transcript files for only protein coding regions on chromosomes 1–22 and X in bed format using a custom Python script. Overlapping regions were merged using BEDTools v2.28.0 [31]. RefSeq contained 197,736 exons and 19,259 genes and Ensembl 209,103 exons and 20,691 genes.

Disease genes were derived from the Online Mendelian Inheritance in Man (OMIM)’s Synopsis. The coding regions for the longest transcripts of 4531 OMIM genes with the highest level of evidence were extracted from the RefSeq transcripts.

### Downsampling, coverage calculation, GC content and evenness scores

Sequence data were downsampled using SAMTools v1.10. [32] for all samples. Single base-pair coverage of human protein coding regions was calculated for samples in all coverage level groups using BEDTools v.2.28.0. GC content was also calculated using BEDTools v2.28.0. The distribution of coverage over target regions was assessed by calculating an evenness score as defined by Mokry et al*.* [33]. The evenness score represents the fraction of sequenced bases that do not have to be redistributed from above-average coverage to below-average coverage positions to obtain completely even coverage for all targeted positions. This is a measurement that is relatively independent on sequencing depth.

### Variant comparison

Variants for all Illumina samples (WES and SR WGS) were called using the GATK HaplotypeCaller (version 3.4) [34]. Target exonic regions for respective kits were extended 200 bp upstream and downstream for variant calling. DeepVariant (version 1.1.0) was used for variant calling with default parameters for LR WGS samples. All variants were subsequently annotated by our in-house pipeline based on the Ensembl Variant Effect Predictor (VEP). Coding variants were compared by selecting true positive variants with allele frequencies > 0.001 (ExAC v0.2).

### CNV comparison for twist

To examine the effect of coverage level on the sensitivity of copy number variation (CNV) detection, we used two independent data sets as described in Sample Collection. We used 20 randomly selected Twist samples (Additional file 1: Table S1) and additional 14 Twist samples with previously validated CNVs (Additional file 1: Table S2). We used an additional 100 Twist samples as a reference pool for CNV calling (Additional file 1: Table S2). All samples were downsampled to both 100× and 70× coverage for comparison. Since Conifer is used in in-house diagnostic pipeline, CNV calling was performed using Conifer v.0.2.2. We considered true CNVs to be calls with SVD-ZRPKM values smaller than -1.7 (deletions) or 1.7 (duplications). We additionally removed 3 singular values based on the inflection point of scree plots (Additional file 1: Fig. S1).

## Results

We compared three different widely used enrichment kits (Agilent V5, Agilent V7 and Twist) as well as SR- and LR-WGS. Randomly selected whole blood and tissue samples for all kits and SR-WGS were sequenced on an Illumina NovaSeq 6000 sequencer using 2 × 150 paired-end sequencing, and LR-WGS samples were sequenced on a Pacific Biosciences Sequel II instrument.

### Percentage of coding regions covered (RefSeq and Ensembl) in WES and WGS

Differences in sequence coverage foremost stem from differences in the target design. Therefore, we compared the overlap between the extended targets (± 200 bp) of three capture kits analyzed (Agilent v5, Agilent v7 and Twist) with coding regions as defined using RefSeq and Ensembl data (see “Methods” section). While the older Agilent v5 capture kit did not target about 980 kb of RefSeq coding sequence, the newer Agilent v7 and Twist kits perform substantially better (148 kb missing, Agilent v7; 83 kb missing, Twist; Additional file 1: Table S3). The coding regions as defined by Ensembl data are broader than those defined using RefSeq data. We found that Twist does not target about 753 kB of these regions, whereas Agilent v7 does not target about 348 kB (Additional file 2).

**Fig. 1:**
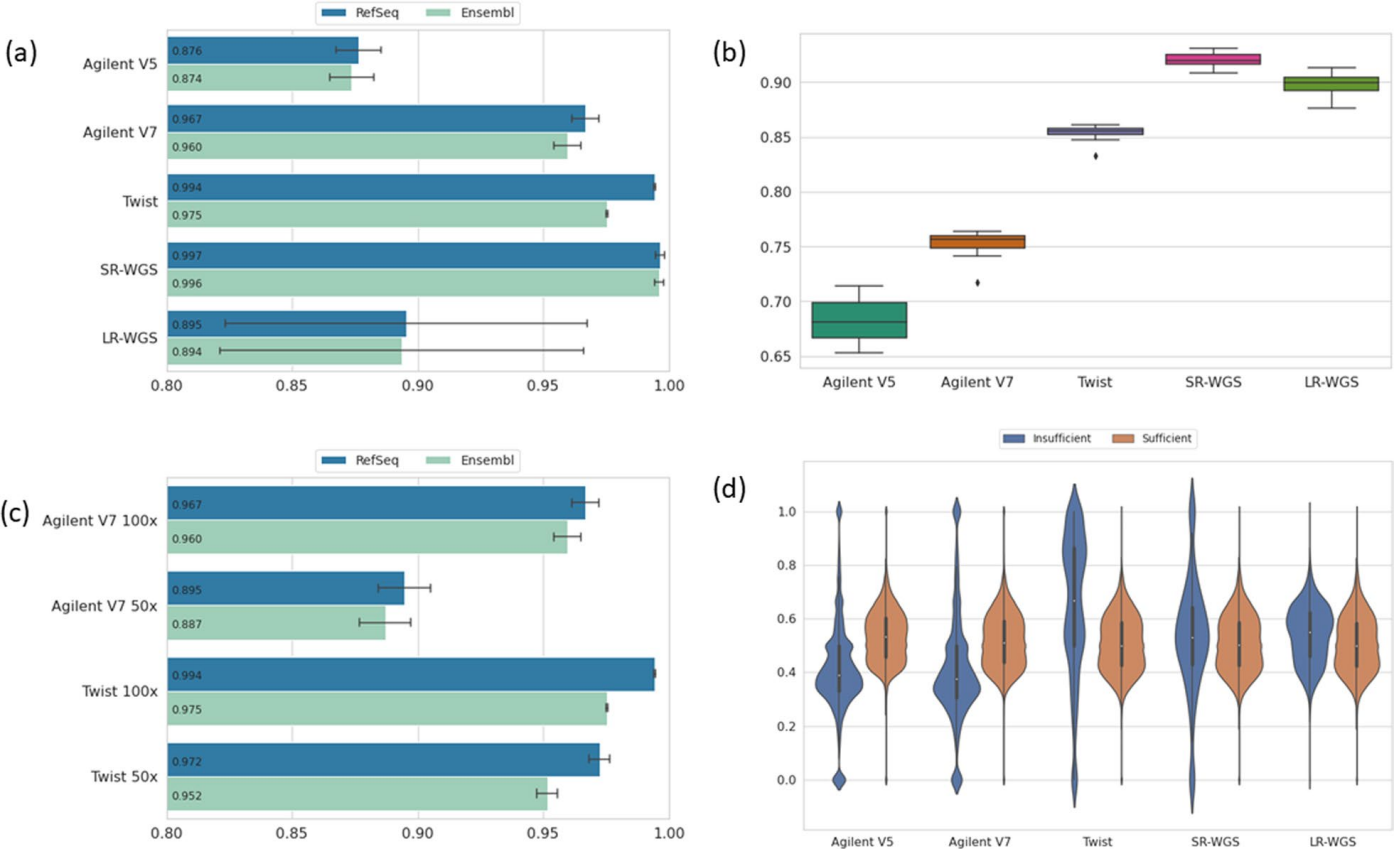
Comparison of exome kits and sequencing platforms. **A** Ratio of coding regions covered at ≥ 20× for different enrichment and sequencing platforms for RefSeq and Ensembl. **B** Boxplots of evenness scores for different enrichment kits and sequencing platforms. **C** Ratio of coding regions covered at ≥ 20× for different enrichment platforms when down-sampled to 50×. **D** GC content of insufficiently and sufficiently covered targets is significantly different for all kits and platforms (Mann–Whitney U-Test *p* value < 0.001)

We then compared the percentage of the coding regions covered by at least 20× across WES data sequenced using each of the three exome capture kits, SR-WGS data and LR-WGS data (Table 1). All exome samples were downsampled to 100× average coverage (Additional file 1: Table S4). The highest coverage ratio at > 20× for both RefSeq and Ensembl coding regions was obtained with Twist enrichment kits (Fig. 1A). Twist covered 99.4% of the RefSeq and 97.5% of the Ensembl coding regions by 20×, while Agilent v7 and Agilent v5 covered 96.7% and 87.6% of RefSeq coding regions and 96% and 87.4% of Ensembl coding regions, respectively. However, SR-WGS is superior to all three WES capture kits by this metric, covering 99.7% and 99.6% of RefSeq and Ensembl coding regions at 20×. LR-WGS reached only 89.5%, likely due to the lower average coverage of only 30× (Additional file 1: Table S5a). This is also the reason for the high standard deviation for LR-WGS. When we considered 10× minimal coverage sufficient in all LR-WGS samples, we found that LR-WGS performed similarly to SR-WGS (SR-WGS: 99.90%, LR-WGS: 99.2% for 10× RefSeq coverage; Additional file 1: Table S5b).

### Evenness of coverage

We also calculated an evenness of coverage score for all samples (“Methods” section). Twist exomes have better uniformity of sequence coverage using this metric compared to Agilent v5 and v7 exomes (Fig. 1B, Additional file 1: Table S6). An advantage of uniform coverage is that samples can potentially be sequenced at lower average coverage, thereby providing considerable cost-savings. To investigate this in our data, we downsampled Agilent v7 and Twist exome samples to 50× mean coverage. Downsampled Twist exomes achieved a 97.2% and 95.2% coverage ratio for RefSeq and Ensembl coding regions, respectively, constituting a 2.2% and 2.3% decrease in sufficiently covered regions (Fig. 1C). In downsampled Agilent v7 exomes, the decrease in sufficiently covered regions was 7.2% and 7.3% resulting in 89.5% and 88.7% coverage ratios for RefSeq and Ensembl coding regions, respectively.

### GC content

A well-known reason for poor performing enrichment targets is extreme GC content. Therefore, we assessed the GC content of regions with insufficient coverage (< 20×) (“Methods” section). The median GC ratio of insufficiently covered regions in our data was 38.8%, 37.5%, 66.6%, 53.1% and 55% for Agilent v5, Agilent v7, Twist, WGS and LRS samples, respectively (Fig. 1D). In regions that were well covered, the median GC content for all platforms was between 50 and 53.2%. Interestingly, while Agilent v5 and v7 typically perform poorly in low GC regions, in Twist samples most low coverage regions have an high GC content (> 65%). As expected, the GC content distribution of well and poorly covered regions in SR- and LR-WGS data are similar.

### Twist enrichment kits have lower minimum average coverage requirements than Agilent V7 kits

**Fig. 2:**
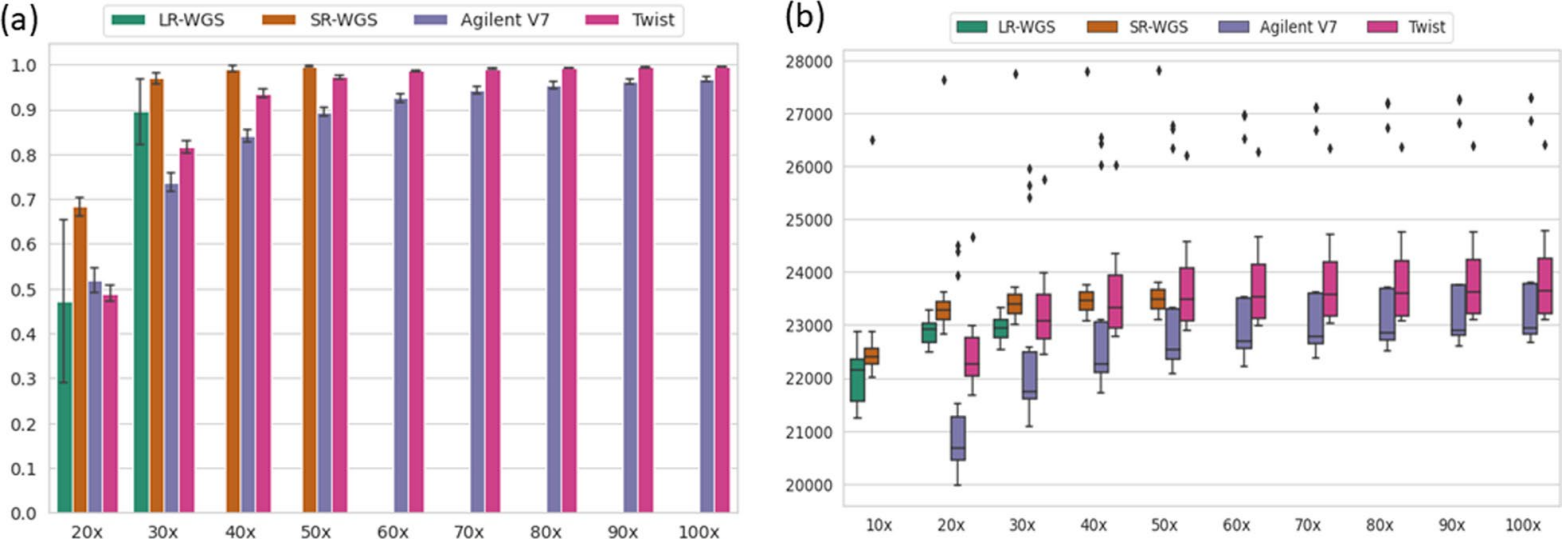
Comparison of enrichment kits and sequencing platforms at different coverage levels. **A** Overview of base pair coverage ratio at least 20× per platform for RefSeq coding regions. *X*-axis represents the mean coverage levels of the samples in each platform, *y*-axis represents the average ratio of base pairs that exceeds 20× coverage level for all samples in the corresponding kit/platform. **B** Boxplots represent the distribution number of coding variants for samples of each platform at different coverage levels. *X*-axis depicts the coverage levels, and *y*-axis shows the number of number of coding variants

Next, we wanted to establish a minimum level of average coverage sufficient to obtain results comparable to 100× average coverage in exome data. To do this, we assessed the effect of gradually downsampling average coverage to 20× in exome data (Twist and Agilent v7 kits) and 10× in genome data (Fig. 2A, Additional file 1: Table S7). We show that the percentage of covered coding regions declines more rapidly in downsampled Agilent v7 exomes compared to Twist exomes. For example, when downsampling from 70× to 60× average coverage the percentage of covered coding regions declines by 1.7% in Agilent v7 exomes (94.2–92.5) versus just 0.1% in Twist exomes (99–98.9%). When average coverage is reduced to 30×, only 74% and 82% of coding sequence is covered more than 20× for Agilent v7 and Twist, respectively. We verified that these results are also valid for samples with DNA from other tissues (amniotic fluid, basal mucosa and fibroblasts) than blood enriched with Twist (Additional file 1: Table S8; Fig. S2).

To investigate how lower average coverage might impact variant detection, we selected all common coding variants with an ExAC allele frequency > 0.001 (0.1%) in all WES and WGS samples. In gradually downsampled Twist exomes, the median number of coding variants decreased only slightly up to 40×. While the difference between median number of coding variants was 360 between 100× and 40×, this difference increased to 690 variants between 40× and 20× for Twist samples (Fig. 2B). Similarly, the median number of coding variants remains relatively consistent down to 20× for SR-WGS samples, after which we observed a strong decline. However, for Agilent V7 samples median number of coding variants decreased by 255 when average coverage of samples reduced to 60× from 100× and this difference was 2019 when average coverage reduced to 40× from 60×. On average, the number of detected coding variants with ExAC allele frequency > 0.1% was consistently smaller for Agilent V7 samples compared to Twist samples at each level of average coverage.

### Coverage of clinically relevant genes

Our results show that Twist outperforms other kits and performs similar to WGS in terms of coverage and SNV detection. Additionally, we show that reducing average coverage to 70× in Twist exome data would likely have a negligible impact on the percentage of sufficiently covered regions and sensitivity of SNV detection. To determine whether 70× Twist exomes could be used in clinical diagnostics, we performed further detailed comparisons between Twist samples with average 70× and 100× coverage. RefSeq coding regions were used for further comparisons since Twist targets cover RefSeq regions better than Ensembl regions.

First, we verified that our downsampling procedure did not affect our results by repeating the coding region coverage analyses for 7 samples that were originally sequenced at 70× average coverage. On average, 98.8% of the RefSeq coding regions were covered by at least 20× in these samples (Additional file 1: Table S1; Table S7a).

**Fig. 3:**
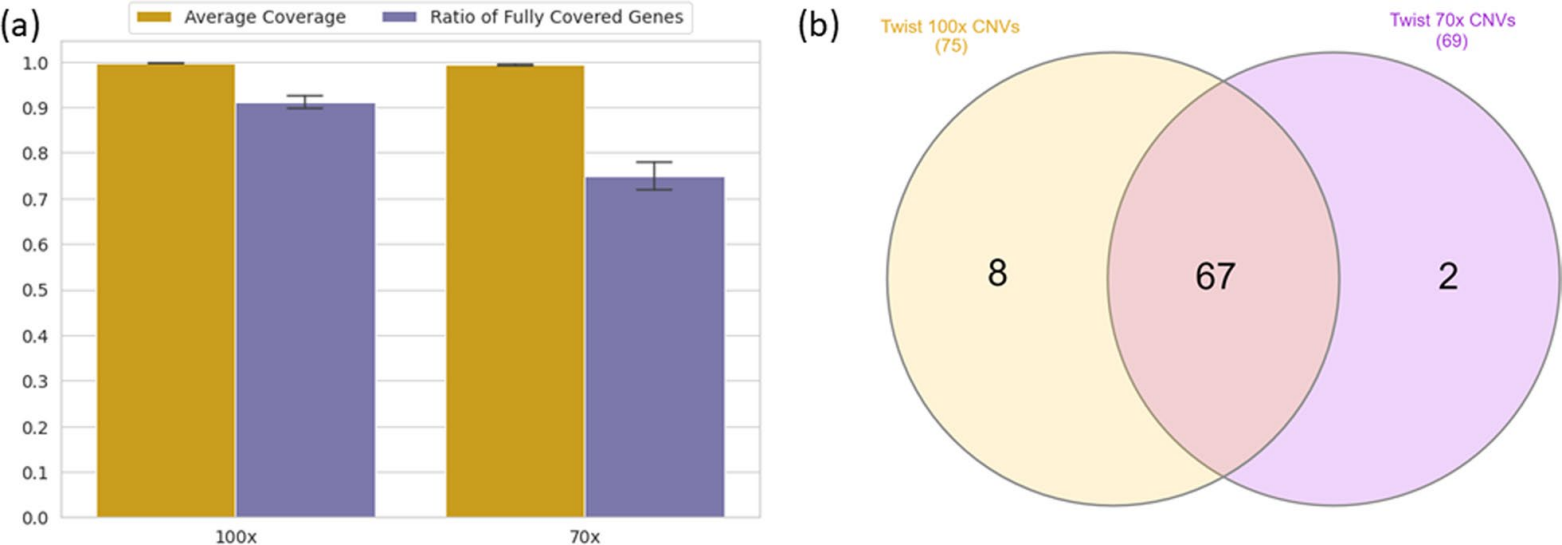
Comparison of Twist enrichment kit for 100× and 70× coverage levels. **A** Percentage of base pairs that exceeds 20× coverage level for OMIM genes (yellow) percentage of genes which were fully covered with at least 20× coverage (purple) **B** Venn diagram that represents the number of CNVs for samples enriched with TWIST at 100× (yellow) and 70× (purple) coverage levels

To better understand the clinical importance of differences in coding region coverage, we assessed the coverage of transcripts of 4,531 OMIM transcripts which consist of ± 10 mb distributed over 62,233 exons extracted from RefSeq coding regions (“Methods” section). We examined the percentage of these transcripts with at least 20× coverage at all bases. In 100× Twist exome samples, an average of 91% of OMIM transcripts were fully covered. In 70× Twist exome samples, we observe a substantial decrease in the complete coverage of these transcripts (74.8%, Fig. 3A). This drop is driven by a relatively small proportion of coding bases: 95% of bases exceed 20× coverage in 95% of OMIM transcripts in 70× Twist data (Additional file 1: Table S9).

### Genuine SNVs can still be detected in 70× twist exomes

The number of ExAC AF > 0.001 variants detected in 70× Twist exomes was comparable to that in 100× Twist exomes (0.5% of variants not detected at 70×). Although the total number of detected variants decreased only slightly for Twist when down-sampling from 100 to 70× coverage, we were interested in which variants specifically were lost. 20% were located in genes such as *MUC6, TAS2R45, HLA-DRB5* and *MUC4* that have previously been associated with mapping artifacts due homologous regions (Additional file 1: Fig. S3 [35]). 80% were mapped to various genes in different samples. In addition, we wondered whether down-sampling had an effect on GATK quality scores, since these are commonly used to select less reliable calls for orthogonal validation. While we observed that GATK quality scores were highly correlated in 70× and 100× Twist exomes (Additional file 1: Fig. S4), we also show that the tails of the quality score distribution may be affected by the drop in coverage. Only 9% of variants had GATK quality scores less than 500 in 100× Twist exomes, while this increased to 19% in 70× samples.

### CNVs can still be detected in 70× twist exomes

Another potential concern with having lower average coverage is the ability to call copy number variants (CNVs) based on depth of coverage using a relatively heterogeneous reference pool of only 100 samples. To address this, we examined the effect of lower coverage on CNV detection using Conifer. We compared CNV calls in 20 Twist samples with downsampled 70× coverage to those with 100× coverage (see “Methods” section). To do this, samples in the reference pool were also downsampled to 100× and 70× average coverage. SVD normalization enables Conifer to remove coverage biases introduced by the capture and sequencing of exomes and detect only rare CNVs. Accordingly, in this study 67 CNVs were called in samples at both 100× (75 CNVs in total) and 70× (71 CNVs in total) coverage (Fig. 3B). In downsampled 70× Twist exomes, 6 duplications and 1 deletion did not exceed the SVD-ZRPKM value threshold (“Methods” section) and 1 duplication was not called. In comparison, 1 duplication and 1 deletion did not exceed filtering thresholds in 100× Twist samples (Additional file 1: Table S10).

We also compared the CNV calls for 100× and 70× average coverage levels in another group of unsampled Twist exomes with a set of previously validated CNVs (Additional file 1: Table S2). In 100× Twist samples, 10 out of 15 CNVs were called, 3 CNVs did not exceed the filter thresholds and 2 CNVs were not called (Table 2). In 70× Twist samples, 8 CNVs were called and 5 CNVs did not exceed the filter threshold. The same 2 CNVs that were missed in 100× Twist samples were also undetected. Although 3 CNVs did not exceed the SVD-ZRPKM threshold for both coverage levels, they could be easily identified based on visual inspection of the coverage bedgraphs (Additional file 1: Figure S5). Almost all CNVs detected by 100× samples were also detected by 70× samples; however, a few of them were filtered out since they did not exceed the SVD-ZRPKM threshold value in both sample sets.

**Table 2:** CNV Status of 100× and 70× samples for the validated CNVs

Validated CNVs					CNV Status of 100× and 70× Samples	
Sample	Chromosome	Start position	End position	CNV type	100× Samples	70× Samples
CNV_Sample_1	17	2,516,458	2,808,662	Deletion	Cannot exceed threshold	Cannot exceed threshold
CNV_Sample_2	15	23,572,075	28,567,878	Deletion	Called	Cannot exceed threshold
CNV_Sample_3	8	116,085	43,218,462	Duplication	Not called	Not called
CNV_Sample_4	22	21,562,426	22,937,526	Deletion	2/3 segments	2/2 segments
CNV_Sample_5	16	14,927,708	16,367,932	Duplication	2/3 segments	1/2 segments
CNV_Sample_6	22	18,893,887	21,414,817	Deletion	3/4 segments	3/5 segments
CNV_Sample_7	23	24,190,859	26,236,246	Duplication	Cannot exceed threshold	Cannot exceed threshold
CNV_Sample_8	19	11,105,503	11,141,569	Deletion	Not called	Not called
CNV_Sample_9	11	pter	926,088	Duplication	Called	Cannot exceed threshold
CNV_Sample_10	16	15,457,515	17,564,653	Deletion	2/2 segments	2/3 segments
CNV_Sample_11	17	1,082,960	1,490,254	Duplication	Called	Called
CNV_Sample_12	8	12,051,483	43,218,462	Duplication	2/5 segments	6/19 segments
CNV_Sample_12	8	pter	7,079,475	Deletion	1/2 segments	1/2 segments
CNV_Sample_13	6	160,638,463	qter	Deletion	4/7 segments	3/9 segments
CNV_Sample_14	22	50,297,485	50,757,432	Deletion	Cannot exceed threshold	Cannot exceed threshold

Columns depict (from left to right): sample ID of the samples with previously validated CNVs by visual inspection and concordance with phenotype; chromosome number; start position; end position of the validated CNV; CNV type; status of validated CNV for samples at 100× coverage; status of known CNV for sample at 70× coverage

## Discussion

Whereas for whole genome sequencing it is customary to only obtain 30–40× average coverage, this is not the same for exome sequencing due to the more uneven coverage that is the result of differences in capture efficiency for individual probes. Various studies have tried to help investigators make an informed decision on which sequencing platform to choose by comparing the performance of different WES kits with each other and with WGS using coverage and variant identification statistics [26, 36, 37]. Here we showed that Twist exome coverage is more uniform and consistent than coverage from other exome kits and that there is a substantially smaller fraction of insufficiently covered coding bases. Although not as good as WGS, the results are very similar. These improvements are likely a result of the more or better balanced pool of oligonucleotides, i.e., baits, in the exome kit; however, usually the individual sequence details and molarities are not shared by the providers.

Our results suggest that with lower average coverage than the commonly used 100–120× [38], Twist exomes will achieve a similar performance as other exome kits at higher coverage. We find that at 70× average coverage the sensitivity for SNV detection is hardly affected and that there is only a small effect on the sensitivity of detecting CNVs. In our experience, the sensitivity of CNV detection is likely to be more dependent on the size and quality of reference cohort that is used for CNV detection. We verified that these results are consistent for samples that are originally sequenced at 70× and for different tissues than blood. However, QC thresholds may be adjusted by considering the strong increase in the variants with score below 500 and missed CNVs due to the SVD-ZRPKM thresholds in Twist 70× samples.

One class of variants that was not considered here are mosaic variants. It is unavoidable that the detection of mosaic variants will suffer from reduced overall coverage and this could be a reason to sequence at higher coverage. However, mosaic variants are relatively rare, and the sensitivity to detect high level mosaic variants (> 10% VAF) will not substantially decrease [39].

We estimate that by performing WES at only 70× average coverage compared to 120× a 40% reduction ((120–70)/120) in sequencing costs can be achieved. Depending on the price for library preparation and exome capture kit, we estimate an overall price reduction for WES of 20–30% could be possible. In addition, our results may be used to re-evaluate minimal average coverage thresholds, for clinical exome sequencing, and lead to fewer resequencing of samples with insufficient coverage.

We also compared our results to LR-WGS data. Whereas we previously found that LR-WGS provides coverage in regions that are missed by short-read sequencing [40], we find that for coding regions on average LRS has slightly lower coverage than SR-WGS, although still better than WES. This may have to do with the novelty of the technology and may improve over time to surpass SR-WGS (Additional file 3).

In conclusion, we found that Twist exome capture represents a significant improvement compared to other exome capture techniques. Exome coverage of Twist is more uniform and consistent than other enrichment kits. Because of more uniform coverage distribution, a minimum average coverage of 70× will provide sensitivity to detect both SNVs and CNVs similar to 150× WES samples with other enrichment kits.

## Supplementary Information

**Additional file 1: Table S1**. Initial mean coverage of all samples.** Table S2**. Initial mean coverage of all samples used in CNV analysis.** Table S3**. Overview coding bases that are not targeted by different enrichment kits, based on the extended target regions of manufacturers. Length of Ensembl is 35,123,365 bp and RefSeq is 33,879,640 bp.** Table S4**. Coverage statistics of the samples after downsampling.** Table S5**. Overview of base pair coverage for RefGene and Ensembl coding regions. Mean and standard deviation of base pair coverage by at least 20× per platform. Mean and standard deviation of base pair coverage by at least 10× per platform.** Table S6**. Average Evenness of coding regions for different platforms.** Table S7**. Overview of base pair coverage ratio for samples with different coverage levels. Ratio of covered regions by at least 20×Ratio of covered regions by at least 10×.** Table S8**. Overview of base pair coverage ratio by at least 20× for samples with different coverage levels for blood and tissue samples enriched with Twist.** Table S9**. Percentage of the OMIM transcripts that are covered at certain level of base pair coverage ratio by at least 20×.** Table S10**. CNVs called for 20 Twist samples.** Fig. S1**. Scree plots of singular values generated with Conifer a Scree plot generated for 20 Twist samples at 100× coverage b Scree plot generated for 20 Twist samples at 70× coverage c Scree plot generated for 14 Twist samples with validated CNVs at 100× coverage d. Scree plot generated for 14 Twist samples with validated CNVs at 70× coverage.** Fig. S2**. Overview of base pair coverage ratio by at least 20× for blood samples and tissue samples enriched with Twist.** Fig. S3**. Missing variants in samples with average coverage 70× compared to 100×.** Fig. S4**. A GATK quality scores of variants identified in 100× average coverage samples compared to 70× average samples. B Zoom in of the plot in A for scores smaller than 10,000.** Fig. S5**. CNVs can't exceed the threshold for samples in both 100× and 70× coverage levels.** Fig. S6**. CNVs called by samples with 100× average coverage and not exceed threshold for 70× coverage level. Fig. S7. Visual graphs for segmentedly called CNVs.

**Additional file 2**. Ensembl Coding Regions Missed by Twist and Agilent V7 Kits.

**Additional file 3**. List of SolveRD consortium members with affiliations.

**Additional file 4**. DNA Concentration and QC Values.

## Data Availability

Target files for enrichment kits are available from the corresponding author upon request.
