# Supplementary material for: Twist exome capture allows for lower average sequence coverage in clinical exome sequencing
Source: Hum Genomics. 2023 May 3;17:39. doi: 10.1186/s40246-023-00485-5 (PMC10155375; doi:10.1186/s40246-023-00485-5)
Supplement: Supplementary file 1 — Additional file 1: Table S1. Initial mean coverage of all samples. Table S2. Initial mean coverage of all samples used in CNV analysis. Table S3. Overview coding bases that are not targeted by different enrichment kits, based on the extended target regions of manufacturers. Length of Ensembl is 35,123,365 bp and RefSeq is 33,879,640 bp. Table S4. Coverage statistics of the samples after downsampling. Table S5. Overview of base pair coverage for RefGene and Ensembl coding regions. Mean and standard deviation of base pair coverage by at least 20× per platform. Mean and standard deviation of base pair coverage by at least 10× per platform. Table S6. Average Evenness of coding regions for different platforms. Table S7. Overview of base pair coverage ratio for samples with different coverage levels. Ratio of covered regions by at least 20×Ratio of covered regions by at least 10×. Table S8. Overview of base pair coverage ratio by at least 20× for samples with different coverage levels for blood and tissue samples enriched with Twist. Table S9. Percentage of the OMIM transcripts that are covered at certain level of base pair coverage ratio by at least 20×. Table S10. CNVs called for 20 Twist samples. Fig. S1. Scree plots of singular values generated with Conifer a Scree plot generated for 20 Twist samples at 100× coverage b Scree plot generated for 20 Twist samples at 70× coverage c Scree plot generated for 14 Twist samples with validated CNVs at 100× coverage d. Scree plot generated for 14 Twist samples with validated CNVs at 70× coverage. Fig. S2. Overview of base pair coverage ratio by at least 20× for blood samples and tissue samples enriched with Twist. Fig. S3. Missing variants in samples with average coverage 70× compared to 100×. Fig. S4. A GATK quality scores of variants identified in 100× average coverage samples compared to 70× average samples. B Zoom in of the plot in A for scores smaller than 10,000. Fig. S5. CNVs can't exceed the threshold for samples in both [file 40246_2023_485_MOESM1_ESM.pdf]

Supplementary Tables

***Table S1** Initial mean coverage of all samples*

Agilent V5		Agilent V7		Twist		Twist 70x		Twist Tissue		LR WGS		SR WGS	
Sample	***Coverage***	***Sample***	***Coverage***	***Sample***	***Coverage***	***Sample***	***Coverage***	***Tissue Type***	***Coverage***	***Sample***	***Coverage***	***Sample***	***Coverage***
V5 _1	163.8269	V7_1	131.133	Twist_1	119.7242	T_70x_1	69.2939	Amniotic fluid	105.492	LRS_1	26.4703	SRS_1	50.8632
V5_2	181.0402	V7_2	142.1496	Twist_2	121.0787	T_70x_2	69.317	Buccal swap	115.813	LRS_2	24.2411	SRS_2	50.9306
V5_3	205.4801	V7_3	165.3523	Twist_3	122.8173	T_70x_3	69.4421	Skin biopsy	116.2917	LRS_3	24.8533	SRS_3	50.9787
V5_4	222.4174	V7_4	177.8389	Twist_4	126.1653	T_70x_4	69.7561	Fibroblasts	132.0071	LRS_4	24.5654	SRS_4	51.2223
V5_5	253.6392	V7_5	187.6178	Twist_5	127.496	T_70x_5	70.1313	Amniotic fluid	161.4039	LRS_5	27.014	SRS_5	52.1352
V5_6	263.6631	V7_6	197.3693	Twist_6	131.5026	T_70x_6	70.6575			LRS_6	29.2191	SRS_6	54.5899
V5_7	269.2651	V7_7	200.9092	Twist_7	134.4042	T_70x_7	71.0797			LRS_7	34.9063	SRS_7	55.3958
V5_8	273.7999	V7_8	205.1483	Twist_8	134.6166					LRS_8	33.8751	SRS_8	58.1557
V5_9	274.162	V7_9	205.6777	Twist_9	136.0387					LRS_9	29.6375	SRS_9	58.1796
V5_10	275.2554	V7_10	212.0162	Twist_10	137.6197					LRS_10	27.7202	SRS_10	59.3881
V5_11	289.4508	V7_11	212.5724	Twist_11	138.8225					LRS_11	30.9037	SRS_11	59.5685
V5_12	299.1956	V7_12	218.7635	Twist_12	140.3604					LRS_12	33.5223	SRS_12	59.8503
V5_13	300.1645	V7_13	227.2871	Twist_13	142.573					LRS_13	25.82	SRS_13	61.903
V5_14	305.6527	V7_14	260.105	Twist_14	148.1992					LRS_14	30.9923	SRS_14	62.4113
V5_15	325.5734	V7_15	278.6044	Twist_15	149.8331					LRS_15	38.8612	SRS_15	65.6226
V5_16	334.6465	V7_16	313.0177	Twist_16	150.2781					LRS_16	32.2388	SRS_16	65.796
V5_17	343.6106	V7_17	358.3344	Twist_17	153.6342					LRS_17	28.0269	SRS_17	66.0597
V5_18	345.4356	V7_18	361.2591	Twist_18	154.7937					LRS_18	27.0959	SRS_18	66.5119
V5_19	348.3071	V7_19	367.0602	Twist_19	156.0731							SRS_19	67.2763
V5_20	348.4157	V7_20	370.6141	Twist_20	158.4619							SRS_20	69.3369

***Table S2** Initial mean coverage of all samples used in CNV analysis*

Samples in the Sample Pool								Samples with Validated CNVs	
**Sample**	**Coverage**	**Sample**	**Coverage**	**Sample**	**Coverage**	**Sample**	**Coverage**	**Sample**	**Coverage**
Pool_Sample_1	77.8009	Pool_Sample_26	100.8479	Pool_Sample_51	116.2502	Pool_Sample_76	132.424	CNV_Sample_1	112.4598
Pool_Sample_2	81.0305	Pool_Sample_27	101.0613	Pool_Sample_52	117.3081	Pool_Sample_77	132.6484	CNV_Sample_2	126.7293
Pool_Sample_3	81.3313	Pool_Sample_28	101.0642	Pool_Sample_53	118.1962	Pool_Sample_78	135.3167	CNV_Sample_3	127.9073
Pool_Sample_4	85.6739	Pool_Sample_29	101.5435	Pool_Sample_54	118.2279	Pool_Sample_79	140.3374	CNV_Sample_4	141.2862
Pool_Sample_5	86.435	Pool_Sample_30	101.6191	Pool_Sample_55	118.3598	Pool_Sample_80	142.2199	CNV_Sample_5	143.8176
Pool_Sample_6	87.8774	Pool_Sample_31	102.6865	Pool_Sample_56	119.1644	Pool_Sample_81	146.628	CNV_Sample_6	145.9675
Pool_Sample_7	90.0065	Pool_Sample_32	103.16	Pool_Sample_57	119.6708	Pool_Sample_82	153.0622	CNV_Sample_7	147.8338
Pool_Sample_8	90.1778	Pool_Sample_33	103.6261	Pool_Sample_58	120.3024	Pool_Sample_83	156.9894	CNV_Sample_8	155.0938
Pool_Sample_9	90.5957	Pool_Sample_34	104.9515	Pool_Sample_59	120.5371	Pool_Sample_84	185.9375	CNV_Sample_9	156.6482
Pool_Sample_10	90.9312	Pool_Sample_35	105.1666	Pool_Sample_60	121.3109	Pool_Sample_85	187.535	CNV_Sample_10	166.4002
Pool_Sample_11	92.2221	Pool_Sample_36	105.2931	Pool_Sample_61	122.8756	Pool_Sample_86	191.0678	CNV_Sample_11	171.7781
Pool_Sample_12	93.0199	Pool_Sample_37	105.8588	Pool_Sample_62	122.9562	Pool_Sample_87	193.1219	CNV_Sample_12	172.2187
Pool_Sample_13	95.519	Pool_Sample_38	106.8717	Pool_Sample_63	123.4615	Pool_Sample_88	223.7725	CNV_Sample_13	175.9155
Pool_Sample_14	95.8274	Pool_Sample_39	108.0398	Pool_Sample_64	123.872	Pool_Sample_89	225.4539	CNV_Sample_14	225.2847
Pool_Sample_15	96.2298	Pool_Sample_40	108.1191	Pool_Sample_65	124.7883	Pool_Sample_90	225.5861		
Pool_Sample_16	97.6624	Pool_Sample_41	108.7199	Pool_Sample_66	124.924	Pool_Sample_91	233.901		
Pool_Sample_17	98.032	Pool_Sample_42	108.8726	Pool_Sample_67	125.8822	Pool_Sample_92	246.4346		
Pool_Sample_18	98.1653	Pool_Sample_43	110.1024	Pool_Sample_68	126.5052	Pool_Sample_93	253.3785		
Pool_Sample_19	98.6428	Pool_Sample_44	110.786	Pool_Sample_69	127.6069	Pool_Sample_94	256.4577		
Pool_Sample_20	98.9098	Pool_Sample_45	112.4812	Pool_Sample_70	128.4637	Pool_Sample_95	260.9494		
Pool_Sample_21	99.2598	Pool_Sample_46	113.0354	Pool_Sample_71	128.9281	Pool_Sample_96	261.5433		
Pool_Sample_22	100.1575	Pool_Sample_47	113.0413	Pool_Sample_72	128.9807	Pool_Sample_97	263.0404		
Pool_Sample_23	100.2788	Pool_Sample_48	113.1695	Pool_Sample_73	129.4933	Pool_Sample_98	264.9999		
Pool_Sample_24	100.4116	Pool_Sample_49	114.402	Pool_Sample_74	130.8537	Pool_Sample_99	266.8803		
Pool_Sample_25	100.6118	Pool_Sample_50	115.7672	Pool_Sample_75	130.9245	Pool_Sample_100	313.429		

Table S3 *Overview coding bases that are not targeted by different enrichment kits, based on the extended target regions (+/-200bp) of manufacturers. Length of Ensembl is 35,123,365 bp and RefSeq is 33,879,640bp*

	**Agilent v5**		**Agilent v7**		**Twist**	
	**Number of Missing Bases**	**Missing Bases (%)**	**Number of Missing Bases**	**Missing Bases (%)**	**Number of Missing Bases**	**Missing Bases (%)**
**Ensembl 91**	1,098,042	3.1	348,077	0.9	753,531	2.1
**RefSeq 61**	980,702	2.8	148,330	0.4	83,690	0.2

Columns depict (from left to right) Number of missing bases; ratio of missing bases in Agilent V5 targets; number of missing bases; ratio of missing bases in Agilent V7 targets; number of missing bases; ratio of missing bases in Twist targets compared to Ensembl 91 and RefSeq 61 transcripts

Table S4 *Coverage statistics of the samples after downsampling*

	**Mean**	**Median**	**Sd**
**Twist**	100.28	100.34	0.47
**Agilent v5**	101.24	101.29	0.76
**Agilent v7**	100.82	100.74	0.68
**SR WGS**	50.43	50.43	0.36
**LR WGS**	29.44	28.62	4.04

Table S5 *Overview of base pair coverage for RefGene and Ensembl coding regions (a) Mean and standard deviation of base pair coverage by at least 20x per platform (b) Mean and standard deviation of base pair coverage by at least 10x per platform*

(a)

		**Agilent_V5**	**Agilent_V7**	**Twist**	**SR WGS**	**LR WGS**
RefSeq	*Mean*	87.60%	96.7%	99.40%	99.70%	89.5%
	SD	$9x10^{-3}$	$6x10^{-3}$	$3x10^{-4}$	$2x10^{-3}$	$7x10^{-2}$
Ensembl	*Mean*	87.40%	96.0%	97.50%	99.60%	89.4%
	SD	$9x10^{-3}$	$6x10^{-3}$	$3x10^{-4}$	$2x10^{-3}$	$7x10^{-2}$

(b)

		**Agilent_V5**	**Agilent_V7**	**Twist**	**SR WGS**	**LR WGS**
RefSeq	*Mean*	92.60%	98.40%	99.60%	99.90%	99.2%
	SD	$7x10^{-3}$	$3x10^{-3}$	$2x10^{-4}$	$2x10^{-4}$	$4x10^{-3}$
Ensembl	*Mean*	92.30%	97.80%	97.80%	99.90%	99.1%
	SD	$7x10^{-3}$	$3x10^{-3}$	$2x10^{-4}$	$2x10^{-4}$	$4x10^{-3}$

Table S6 *Average Evenness of coding regions (RefSeq) for different platforms*

**Platform**	**Evenness Score**	**SD**
Agilent V5	0.68	0.02
Agilent V7	0.75	0.01
Twist	0.85	0.006
SR WGS	0.92	0.006
LR WGS	0.90	0.01

Table S7 *Overview of basepair coverage ratio for samples with different coverage levels (RefSeq coding regions) (a) Ratio of covered regions by at least 20x (b) Ratio of covered regions by at least 10x*

(a)

		**20x**	**30x**	**40x**	**50x**	**60x**	**70x**	**80x**	**90x**	**100x**
**LR WGS**	*Mean*	47.20%	89.50%							
	SD	18%	7.4%							
**SR WGS**	*Mean*	68.30%	96.90%	99.10%	99.60%					
	SD	2.1%	1.4%	0.6%	0.2%					
**Twist**	*Mean*	48.90%	81.60%	93.50%	97.50%	98.90%	99%	99.20%	99.30%	99.40%
	SD	1.8%	1.3%	0.9%	0.4%	0.2%	0.1%	0.06%	0.04%	0.03%
**Agilent V7**	*Mean*	51.90%	73.70%	84.10%	89.50%	92.50%	94.20%	95.40%	96.10%	96.70%
	SD	2.8%	2.2%	1.3%	1.1%	1%	0.8%	0.7%	0.6%	0.6%

(b)

		**10x**	**20x**	**30x**	**40x**	**50x**	**60x**	**70x**	**80x**	**90x**	**100x**
**LR WGS**	*Mean*	49.20%	95.80%	99.20%							
	SD	14.2%	2.8%	0.4%							
**SR WGS**	*Mean*	66.90%	98.80%	99.70%	99.90%	99.90%					
	SD	2.6%	0.6%	0.1%	0.02%	0.01%					
**Twist**	*Mean*		91.20%	97.90%	99%	99.30%	99.40%	99.50%	99.60%	99.60%	99.60%
	SD		1%	0.3%	0.1%	0.04%	0.02%	0.02%	0.02%	0.02%	0.01%
**Agilent V7**	*Mean*		83.70%	92%	95%	96.40%	97.20%	97.70%	98%	98.2	98.40%
	SD		1.9%	1.1%	0.7%	0.5%	0.5%	0.4%	0.4%	0.3%	0.3%

Table S8 *Overview of basepair coverage ratio by at least 20x for samples with different coverage levels for blood and tissue samples enriched with Twist (based on RefSeq coding regions)*

		**20x**	**30x**	**40x**	**50x**	**60x**	**70x**	**80x**	**90x**	**100x**
**Twist Blood**	*Mean*	48.90%	81.60%	93.50%	97.20%	98.90%	99%	99.20%	99.30%	99.40%
	SD	1.8%	1.3%	0.9%	0.4%	0.2%	0.1%	0.06%	0.04%	0.03%
**Twist Tissue**	*Mean*	55.8%	85.5%	96%	97.8%	98.8%	99.2%	99.4%	99.5%	99.5%
	SD	3%	2%	1%	0.3%	0.1%	0.07%	0.05%	0.04%	0.04%

Table S9 *Percentage of the OMIM transcripts that are covered at certain level of base pair coverage ratio by at least 20x*

	**100% Covered**		**99% Covered**		**95% Covered**	
	Mean	Range	Mean	Range	Mean	Range
**Twist 100x**	91%	86.6%-92.4%	94.10%	92.8%-95.3%	97.30%	96.9%-97.9%
**Twist 70x**	74.80%	66.7%-78.7%	87.60%	81.1%-89.5%	95.40%	93.8%-96.7%

Table S10 *CNVs called for 20 Twist samples. * indicates the CNVs called for samples with 70x and cannot exceed threshold value for 100x. ** indicates the CNVs called for samples with 100x and cannot exceed threshold for 70x*

**Twist Samples with 100x Coverage**						**Twist Samples with 70x Coverage**					
Sample ID	Chromosome	Start	Stop	State	Value	Sample ID	Chromosome	Start	Stop	State	Value
Twist_1	chr1	145323690	145327665	dup	1.739773	Twist_1	chr1	145323690	145327610	dup	1.868432
Twist_1	chr1	146643567	146726631	del	-2.09332	Twist_1	chr1	146656070	146724390	del	-1.84108
Twist_1	chr10	12707593	13151288	dup	1.939201	Twist_1	chr10	12706412	13151288	dup	1.921496
Twist_1	chr10	134916200	135094940	dup	1.901974	Twist_1	chr10	134912143	135094940	dup	1.838963
Twist_1	chr14	19553416	19889755	dup	2.487039	Twist_1	chr14	19553416	19889802	dup	2.343711
Twist_1	chr14	20007561	20444724	dup	2.487039	Twist_1	chr14	20007561	20404761	dup	2.343711
Twist_1	chr2	179253735	179316496	dup	2.100524	Twist_1	chr2	179260177	179316496	dup	2.102964
Twist_2	chr14	106471528	106494445	dup	1.804253	Twist_2	chr14	106471635	106494577	dup	1.720859
Twist_2	chr22	42537196	42540465	dup	1.783131	Twist_2	chr22	42537196	42539582	dup	1.763164
Twist_2*	chr5	175306734	175462130	dup	1.603465	Twist_2	chr5	175306734	175462130	dup	1.825411
Twist_2	chr8	56715015	56911058	dup	2.124152	Twist_2	chr8	56717451	56882352	dup	1.98131
Twist_3	chr15	43873199	43988322	dup	1.788842	Twist_3	chr15	43873424	43941032	dup	1.858622
Twist_3	chr15	75580269	75586602	dup	1.788842	Twist_3	chr15	75580269	75586602	dup	1.858622
Twist_4	chr1	161519486	161600992	del	-2.04054	Twist_4	chr1	161519486	161600992	del	-1.99292
Twist_4	chr1	196716240	196801129	del	-2.04054	Twist_4	chr1	196716240	196801129	del	-1.99292
Twist_4	chr14	19889612	19890244	dup	1.803516	Twist_4	chr14	19889612	19890244	dup	1.727706
Twist_4	chr14	24435491	24473601	del	-1.82196	Twist_4	chr14	24435491	24470726	del	-1.75908
Twist_4	chr22	39357391	39410384	del	-1.78463	Twist_4	chr22	39357391	39410384	del	-1.7997
Twist_4	chr22	42522575	42525187	del	-1.78463	Twist_4	chr22	42522852	42525187	del	-1.7997
Twist_4	chr6	32410224	32498001	dup	1.985615	Twist_4	chr6	32485515	32498001	dup	1.900236
Twist_5	chr15	32686865	32691050	dup	2.113434	Twist_5	chr15	32686865	32691050	dup	1.851641
Twist_5	chr6	31964205	32011906	dup	2.928705	Twist_5	chr6	31963983	32011669	dup	2.787062
Twist_5*	chr7	100320286	100336236	del	-1.61163	Twist_5	chr7	100320286	100334972	del	-1.792
Twist_5	chr9	117088571	117099586	dup	1.759014	Twist_5**	chr9	117092184	117095414	dup	1.633007
Twist_6	chr17	34432617	34500284	dup	2.084154	Twist_6	chr17	34432617	34499760	dup	1.807286
Twist_6	chr22	22310633	22453622	dup	1.870889	Twist_6	chr22	22310633	22441285	dup	1.899432

Twist_6	chr3	15676930	15720891	dup	1.914563	Twist_6	chr3	15676930	15720891	dup	2.196561
Twist_6	chr7	74197281	74234553	del	-2.09338	Twist_6	chr7	74193602	74234553	del	-2.07424
Twist_6	chr7	141765514	141794297	del	-2.09338	Twist_6	chr7	141765514	141794297	del	-2.07424
Twist_7	chr10	124344786	124353114	dup	1.79497	Twist_7	chr10	124344786	124352111	dup	1.798133
Twist_7	chr11	1213031	1213532	dup	1.754228	Twist_7	chr11	1212870	1213532	dup	1.772421
Twist_7	chr16	55844838	55855433	dup	1.847259	Twist_7**	chr16	55844838	55855433	dup	1.661555
Twist_7	chr19	45821082	45872405	dup	1.752789	Twist_7**	chr19	45821082	45872405	dup	1.646623
Twist_7	chr19	45883447	45901576	dup	1.782285	Twist_7	chr19	45883447	45901576	dup	1.80885
Twist_7	chr19	54743776	54754819	dup	1.782285	Twist_7	chr19	54742828	54754819	dup	1.80885
Twist_8	chr14	106573311	106780945	dup	2.390154	Twist_8	chr14	106573311	106780945	dup	2.281659
Twist_8	chr15	22074595	22473374	dup	2.240572	Twist_8	chr15	22074595	22473224	dup	2.14372
Twist_8	chr21	44816249	44837654	dup	1.807308	Twist_8	chr21	44816249	44837654	dup	1.899367
Twist_9	chr14	107113740	107199231	dup	1.98799	Twist_9	chr14	107114148	107179262	dup	1.968001
Twist_9	chr15	20740295	21051328	dup	2.42194	Twist_9	chr15	20739496	21051328	dup	2.338623
Twist_9	chr15	22074595	22473374	dup	2.42194	Twist_9	chr15	22074595	22473374	dup	2.338623
Twist_9	chr19	55328988	55365501	del	-1.76077	Twist_9**	chr19	55328988	55363737	del	-1.69019
Twist_10	chr17	61949485	61973871	dup	2.002563	Twist_10	chr17	61949485	61973871	dup	1.938781
Twist_10	chr9	452010	842954	dup	1.939476	Twist_10	chr9	446369	842954	dup	1.909863
Twist_11	chr1	1635262	1636464	dup	1.855168	Twist_11	chr1	1635262	1637171	dup	1.91582
Twist_11	chr1	22313023	22413041	del	-1.85573	Twist_11	chr1	22313023	22408287	del	-1.87225
Twist_11	chr1	145295422	145296371	dup	2.004312			NOT CALLED			
Twist_11	chr1	148004716	148015777	dup	2.004312	Twist_11	chr1	148004732	148015797	dup	1.91582
Twist_11	chr14	19889803	19890244	dup	1.863943	Twist_11	chr14	19889803	19890244	dup	2.065342
Twist_11	chr14	106471437	106478411	dup	1.863943	Twist_11	chr14	106471437	106478411	dup	2.065342
Twist_11	chr17	44407815	44606225	dup	1.961138	Twist_11	chr17	44409917	44430296	dup	1.818138
Twist_11	chr19	43371205	43530524	del	-1.86406	Twist_11	chr19	43371285	43530524	del	-1.82327
Twist_11	chr22	18656559	19028807	del	-2.23901	Twist_11	chr22	18656559	19028807	del	-2.09519
Twist_11	chr22	42477928	42537195	dup	3.649475	Twist_11	chr22	42477928	42537195	dup	3.671541
Twist_11	chr7	141765514	141794297	del	-2.04247	Twist_11	chr7	141765514	141794297	del	-1.99169
Twist_11	chr8	144922877	144940776	dup	2.032342	Twist_11	chr8	144922877	144940776	dup	1.989712
Twist_12	chr9	43626644	43815988	dup	1.730222	Twist_12	chr9	43626644	43815988	dup	1.778092

Twist_13	chr10	135345088	135381952	dup	1.894089	Twist_13	chr10	135341984	135371718	dup	1.716743
Twist_13	chr15	30906348	31085613	del	-1.7783	Twist_13	chr15	30905893	31085613	del	-1.72357
Twist_13	chr16	15092196	15122889	del	-1.78101	Twist_13	chr16	15092196	15120563	del	-1.71141
Twist_13	chr22	42537271	42557364	dup	1.789145	Twist_13**	chr22	42537543	42557364	dup	1.618994
Twist_13	chr3	100348441	100447702	dup	2.52851	Twist_13	chr3	100348441	100438902	dup	2.522464
Twist_14	chr17	36099430	36339990	dup	2.130303	Twist_14	chr17	36104531	36340198	dup	2.044515
Twist_14	chr17	36343885	36455434	dup	2.130303	Twist_14	chr17	36343885	36455434	dup	2.044515
Twist_14	chr6	32609748	32630025	dup	2.020833	Twist_14	chr6	32609748	32632844	dup	1.931862
Twist_14	chr6	168343808	168694621	dup	1.943216	Twist_14	chr6	168343808	168482218	dup	1.889236
Twist_14	chr8	98735106	98900470	dup	1.898946	Twist_14	chr8	98735106	98837381	dup	1.813518
Twist_16	chr12	132905551	133067447	dup	2.008262	Twist_16	chr12	132905551	133067447	dup	2.122999
Twist_18	chr14	19889612	19990665	dup	1.762541	Twist_18**	chr14	19889612	19890244	dup	1.682322
Twist_18	chr14	20181094	20389737	dup	1.762541	Twist_18**	chr14	20211828	20345371	dup	1.682322
Twist_18	chr15	22074595	22473224	del	-1.84039	Twist_18	chr15	22074595	22473224	del	-1.82146
Twist_19	chr10	124343837	124353114	del	-2.25046	Twist_19	chr10	124344786	124357528	del	-2.17242
Twist_20	chr1	110214094	110232988	dup	2.126673	Twist_20	chr1	110213908	110232988	dup	2.022867
Twist_20	chr1	145323653	145330811	dup	2.126673	Twist_20	chr1	145323653	145330811	dup	2.022867
Twist_20	chr16	28604762	28649801	dup	1.755581	Twist_20	chr16	28617141	28634518	dup	1.759205
Twist_20	chr17	25950339	26087204	dup	1.869645	Twist_20	chr17	25950339	26086101	dup	1.761447
Twist_20	chr9	43624654	43816801	del	-1.92698	Twist_20	chr9	43624654	43815988	del	-1.91772

Columns depict(from left to the right) Sample ID; chromosome number; start position; end position for the identified CNVs; CNV type; SVD-ZRPKM value calculated by Conifer

Supplementary Figures

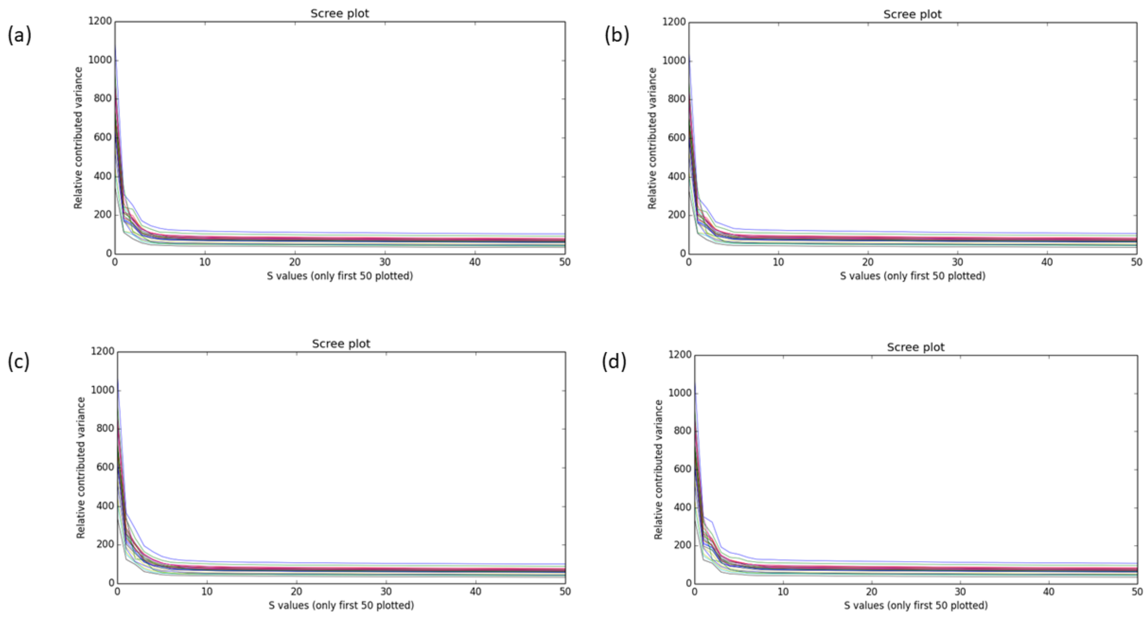

Figure S1 *Scree plots of singular values generated with Conifer a. Scree plot generated for 20 Twist samples at 100x coverage b. Scree plot generated for 20 Twist samples at 70x coverage c. Scree plot generated for 14 Twist samples with validated CNVs at 100x coverage d. Scree plot generated for 14 Twist samples with validated CNVs at 70x coverage*

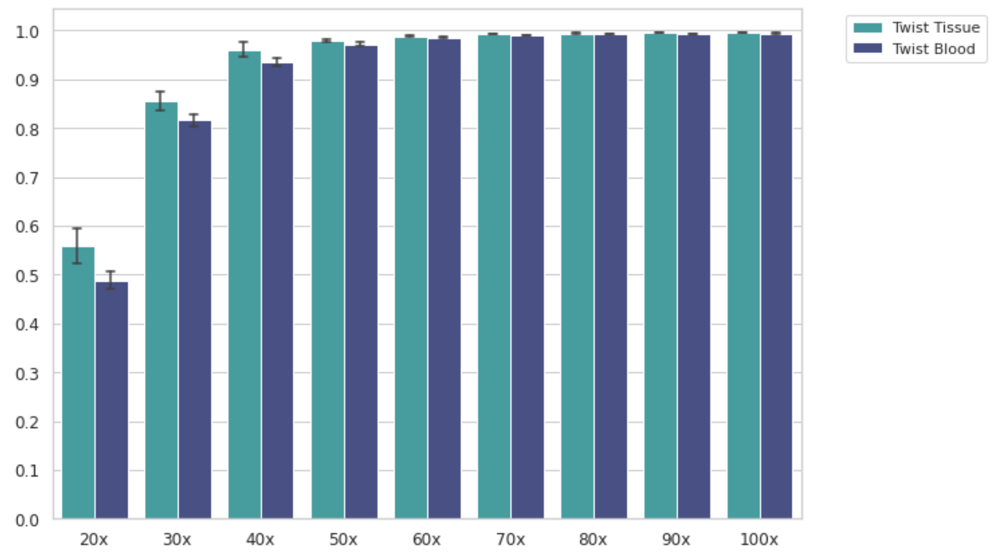

Figure S2 *Overview of basepair coverage ratio by at least 20x for blood samples and tissue samples enriched with Twist. y-axis depicts the ratio of basepairs that exceeds 20x coverage level , x-axis shows the average coverage level of the samples*

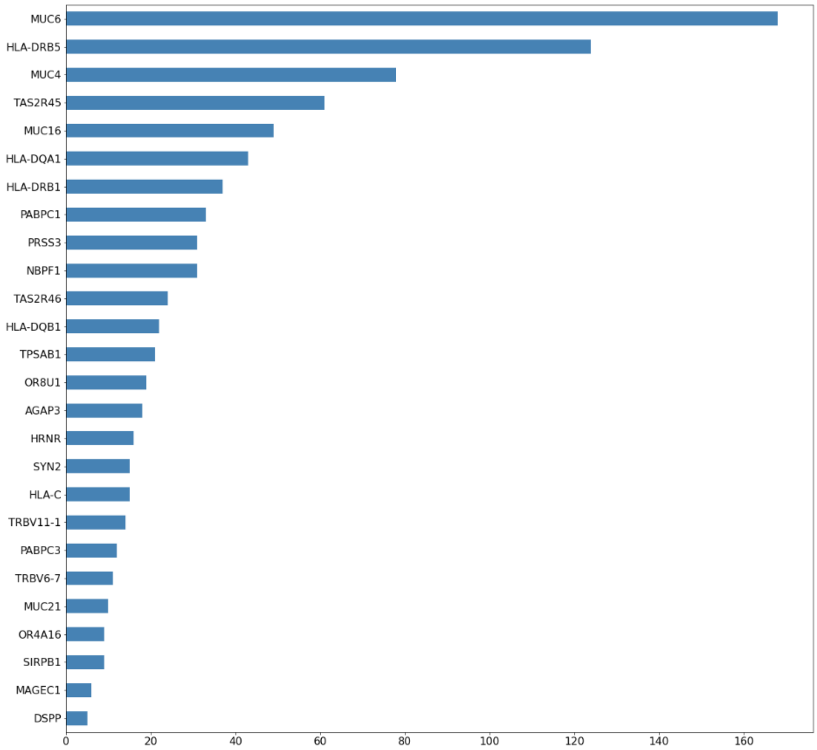

Figure S3 *Missing variants in samples with average coverage 70x compared to 100x. x-axis indicates the total number of variants in 20 samples and y-axis shows the genes that these variants are mapped to.*

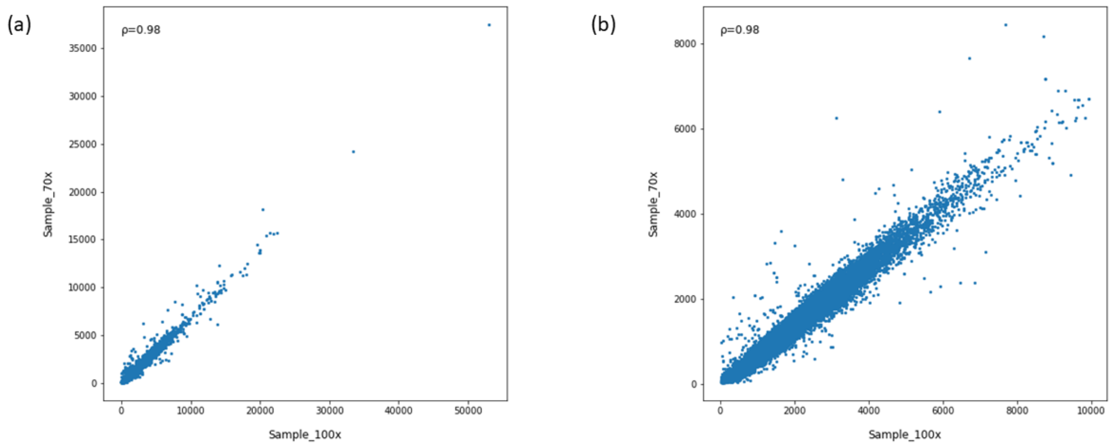

Figure S4 *A) GATK quality scores of variants identified in 100x average coverage samples compared to 70x average samples. B) Zoom in of the plot in A for scores smaller than 10000*

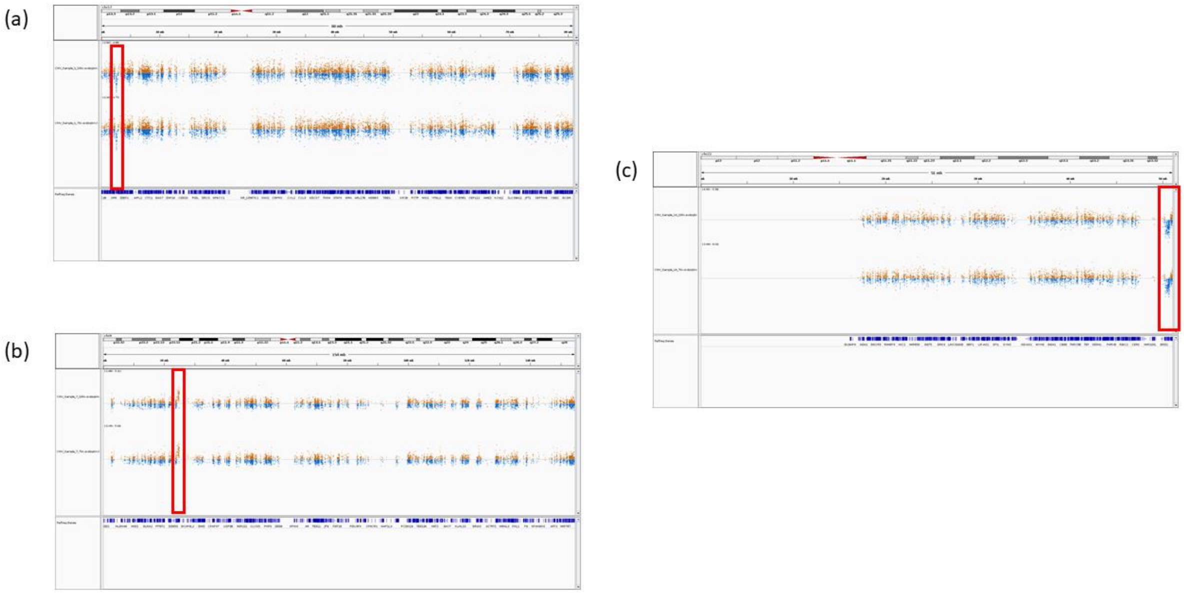

Figure S5 *CNVs can't exceed the threshold for samples in both 100x and 70x coverage levels (a) CNV_Sample_1 (b) CNV_Sample_7 (c) CNV_Sample_14*

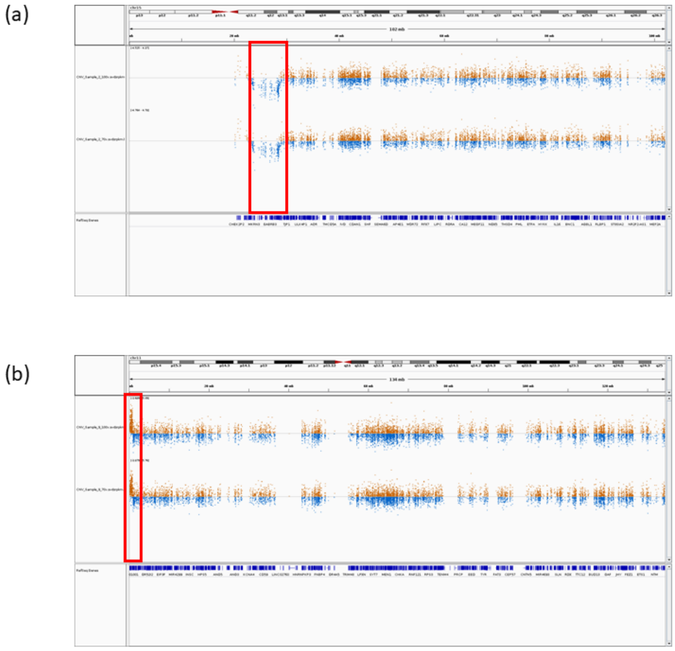

Figure S6 *CNVs called by samples with 100x average coverage and not exceed threshold for 70x coverage level (a) CNV_Sample_2 (b) CNV_Sample_9*

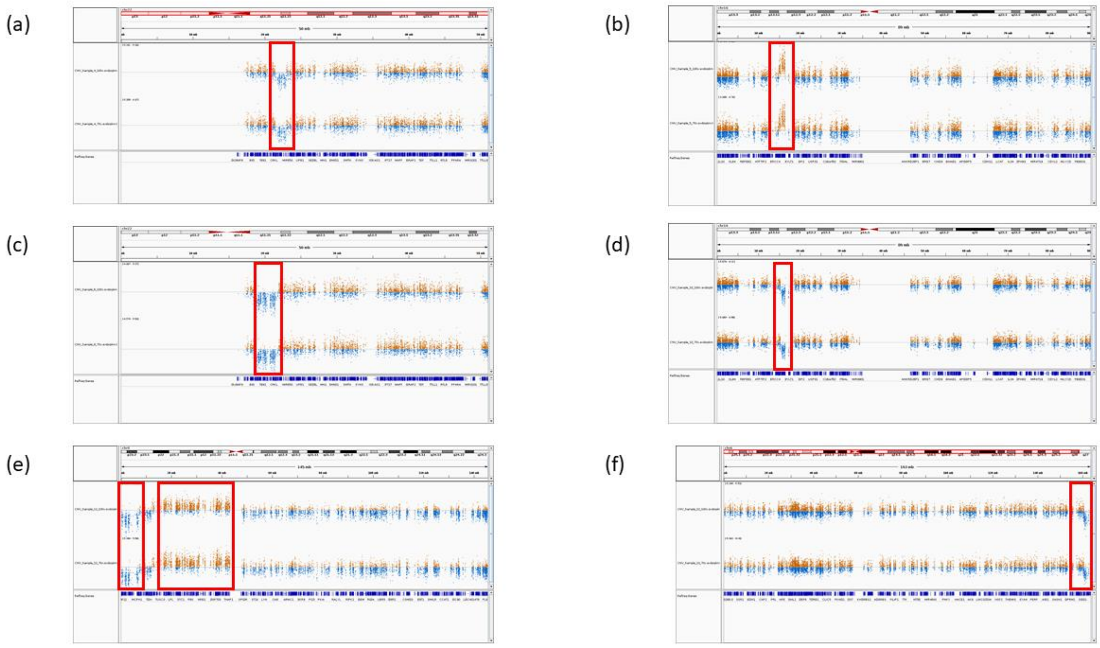

Figure S7 *Visual graphs for segmentedly called CNVs (a) CNV_Sample_4 (b) CNV_Sample_5 (c) CNV_Sample_6 (d) CNV_Sample_10 (e) CNV_Sample_12 (f) CNV_Sample_13*
