# Supplementary material for: Twist exome capture allows for lower average sequence coverage in clinical exome sequencing
Source: Hum Genomics. 2023 May 3;17:39. doi: 10.1186/s40246-023-00485-5 (PMC10155375; doi:10.1186/s40246-023-00485-5)
Supplement: Supplementary file 2 — Additional file 2. Ensembl Coding Regions Missed by Twist and Agilent V7 Kits. [file 40246_2023_485_MOESM2_ESM.pdf]

Chromosome	Start Position	End Position	Gene	TWIST
chr1	138530	139309	AL627309.1	
chr1	367659	368597	OR4F29	
chr1	367659	368597	RP4-669L17.10	
chr1	738532	738618	AL669831.1	
chr1	738532	738618	RP11-206L10.9	
chr1	738532	738618	RP11-206L10.8	
chr1	738788	738812	AL669831.1	
chr1	738788	738812	RP11-206L10.9	
chr1	738788	738812	RP11-206L10.8	
chr1	739121	739137	AL669831.1	
chr1	739121	739137	RP11-206L10.9	
chr1	739121	739137	RP11-206L10.8	
chr1	818043	818058	AL645608.2	
chr1	819496	819513	AL645608.2	
chr1	819961	819983	AL645608.2	
chr1	863255	863261	AL645608.1	
chr1	863255	863261	SAMD11	
chr1	865990	865996	AL645608.1	
chr1	865990	865996	SAMD11	
chr1	1020631	1020690	C1orf159	
chr1	1242717	1242763	ACAP3	
chr1	1334932	1335069	RP4-758J18.2	
chr1	1335361	1335509	RP4-758J18.2	
chr1	1335538	1335687	RP4-758J18.2	
chr1	1335985	1336114	RP4-758J18.2	
chr1	1418427	1418486	ATAD3B	
chr1	1510357	1510659	AL645728.1	
chr1	1919563	1919572	C1orf222	
chr1	2013760	2013899	PRKCZ	
chr1	2118597	2118645	C1orf86	
chr1	2121490	2122032	AL590822.2	
chr1	2121490	2122032	C1orf86	
chr1	2258581	2259042	AL589739.1	
chr1	2258581	2259042	MORN1	
chr1	2310498	2310666	MORN1	
chr1	2342198	2342307	PEX10	
chr1	3303240	3303260	PRDM16	
chr1	3311056	3311058	PRDM16	
chr1	3388222	3388355	ARHGEF16	
chr1	3463562	3463687	MEGF6	
chr1	3789578	3789608	DFFB	
chr1	5948638	5948670	NPHP4	
chr1	6101389	6101399	AL035406.1	
chr1	6101389	6101399	KCNAB2	
chr1	6145800	6146109	KCNAB2	
chr1	6146218	6146374	KCNAB2	

chr1	6156181	6156186	KCNAB2
chr1	6165428	6165487	CHD5
chr1	6173371	6173476	CHD5
chr1	6187143	6187224	CHD5
chr1	6294505	6294571	ICMT
chr1	6342784	6342856	ACOT7
chr1	6342941	6342948	ACOT7
chr1	6366923	6366942	ACOT7
chr1	6694586	6694844	THAP3
chr1	6694586	6694844	DNAJC11
chr1	6711077	6711240	DNAJC11
chr1	6946300	6946317	CAMTA1
chr1	7996575	7996623	TNFRSF9
chr1	9713837	9714101	C1orf200
chr1	9713837	9714101	PIK3CD
chr1	9714256	9714494	C1orf200
chr1	9714256	9714494	PIK3CD
chr1	9778196	9778216	PIK3CD
chr1	10064151	10064257	RBP7
chr1	10226302	10226322	UBE4B
chr1	11075581	11075703	TARDBP
chr1	11076180	11076184	TARDBP
chr1	11155602	11155604	EXOSC10
chr1	11352106	11352201	UBIAD1
chr1	12462621	12462640	VPS13D
chr1	12469924	12469947	VPS13D
chr1	12538855	12538928	VPS13D
chr1	13035543	13035835	PRAMEF22
chr1	13035543	13035835	PRAMEF6
chr1	13036222	13036800	PRAMEF22
chr1	13036222	13036800	PRAMEF6
chr1	13037808	13038381	PRAMEF22
chr1	13037808	13038381	PRAMEF6
chr1	13388458	13389033	PRAMEF8
chr1	13389416	13389702	PRAMEF8
chr1	13424967	13425548	PRAMEF9
chr1	13427298	13427859	PRAMEF9
chr1	13474689	13475262	PRAMEF18
chr1	13476271	13476849	PRAMEF18
chr1	13477236	13477522	PRAMEF18
chr1	13495284	13495570	PRAMEF16
chr1	13495967	13496545	PRAMEF16
chr1	13497570	13498128	PRAMEF16
chr1	13521973	13522259	PRAMEF21
chr1	13523737	13524315	PRAMEF21
chr1	13526312	13526873	PRAMEF21
chr1	13922322	13922335	PDPN

chr1	15573776	15573891	FHAD1
chr1	15754394	15754505	EFHD2
chr1	15808140	15808244	CELA2B
chr1	15960293	15960305	DDI2
chr1	16200610	16200890	SPEN
chr1	16555368	16555558	C1orf134
chr1	16555978	16556038	C1orf134
chr1	16713790	16713796	SZRD1
chr1	16713790	16713796	SPATA21
chr1	16713997	16714207	SZRD1
chr1	16713997	16714207	SPATA21
chr1	16776620	16776715	NECAP2
chr1	17655507	17655529	PADI4
chr1	17655507	17655529	AC004824.2
chr1	17658126	17658219	PADI4
chr1	17658126	17658219	AC004824.2
chr1	17664217	17664284	PADI4
chr1	17664217	17664284	AC004824.2
chr1	17669346	17669388	PADI4
chr1	17669346	17669388	AC004824.2
chr1	17669394	17669415	PADI4
chr1	17669394	17669415	AC004824.2
chr1	17675982	17676070	PADI4
chr1	17675982	17676070	AC004824.2
chr1	17966327	17966348	ARHGEF10L
chr1	18605339	18605378	IGSF21
chr1	21625310	21625318	RP5-1071N3.1
chr1	21625310	21625318	ECE1
chr1	22059157	22059193	USP48
chr1	22180301	22180328	HSPG2
chr1	22304389	22304479	CELA3B
chr1	22324644	22324682	CELA3B
chr1	22336631	22336639	CELA3A
chr1	23695791	23696171	C1orf213
chr1	23695791	23696171	ZNF436
chr1	23697632	23697706	C1orf213
chr1	23697967	23698000	C1orf213
chr1	23698006	23698015	C1orf213
chr1	23956045	23956047	MDS2
chr1	23965599	23965741	MDS2
chr1	23966170	23966285	MDS2
chr1	23966879	23967039	MDS2
chr1	24164555	24164788	HMGCL
chr1	24695837	24695897	STPG1
chr1	24784224	24784323	NIPAL3
chr1	24973570	24973575	SRRM1
chr1	25255531	25255533	RUNX3

chr1	25570707	25570715	C1orf63
chr1	25570915	25570967	C1orf63
chr1	25570981	25570989	C1orf63
chr1	26146744	26147250	AL020996.1
chr1	26146744	26147250	RP1-317E23.6
chr1	26146744	26147250	MTFR1L
chr1	26149837	26149930	RP1-317E23.6
chr1	26149837	26149930	MTFR1L
chr1	26793784	26793876	DHDDS
chr1	26857236	26857343	RPS6KA1
chr1	26870566	26870669	RPS6KA1
chr1	26879309	26879387	RPS6KA1
chr1	27182676	27182717	ZDHHC18
chr1	27659852	27659926	TMEM222
chr1	27661158	27661182	TMEM222
chr1	28086469	28086567	FAM76A
chr1	28526709	28526743	DNAJC8
chr1	28527013	28527030	AL353354.1
chr1	28527013	28527030	DNAJC8
chr1	28527105	28527152	AL353354.1
chr1	28527105	28527152	AL353354.2
chr1	28527105	28527152	DNAJC8
chr1	28527229	28527375	AL353354.2
chr1	28527229	28527375	DNAJC8
chr1	28843351	28843365	RCC1
chr1	28845173	28845469	RCC1
chr1	28859186	28859269	RCC1
chr1	29492521	29492576	SRSF4
chr1	29493241	29493424	SRSF4
chr1	29495015	29495150	SRSF4
chr1	31528216	31528227	PUM1
chr1	31733818	31734023	SNRNP40
chr1	31841960	31841983	FABP3
chr1	32045889	32046339	TINAGL1
chr1	32100447	32100544	PEF1
chr1	32230281	32230403	BAI2
chr1	32379735	32380052	AL136115.1
chr1	32379735	32380052	RP11-84A19.4
chr1	32379735	32380052	PTP4A2
chr1	32834827	32834939	BSDC1
chr1	33127847	33127851	RBBP4
chr1	33139060	33139181	RBBP4
chr1	33299635	33299639	S100PBP
chr1	33439268	33439642	FKSG48
chr1	33791426	33791562	RP11-415J8.3
chr1	33791426	33791562	PHC2
chr1	35183993	35184010	SMIM12

chr1	36032424	36032470	NCDN
chr1	36643159	36643175	MAP7D1
chr1	36881649	36881696	OSCP1
chr1	38200336	38200359	EPHA10
chr1	38292232	38292262	AL929472.1
chr1	38292232	38292262	MTF1
chr1	38293873	38293886	AL929472.1
chr1	38293873	38293886	MTF1
chr1	39339358	39339581	RP5-864K19.4
chr1	39339358	39339581	MYCBP
chr1	39339358	39339581	GJA9
chr1	39391626	39391701	RHBDL2
chr1	39764924	39764929	MACF1
chr1	39925490	39925504	MACF1
chr1	40026777	40026794	PABPC4
chr1	40037279	40037377	PABPC4
chr1	40037279	40037377	RP11-69E11.8
chr1	40341734	40341804	TRIT1
chr1	40782323	40782362	COL9A2
chr1	40828054	40828108	SMAP2
chr1	41229035	41229161	NFYC
chr1	41237056	41237157	NFYC
chr1	41292310	41292366	KCNQ4
chr1	41447659	41447666	CTPS1
chr1	42986886	42986927	CCDC30
chr1	43002863	43002931	CCDC30
chr1	43008460	43008501	CCDC30
chr1	43046503	43046514	CCDC30
chr1	43217312	43217505	LEPRE1
chr1	43262231	43262445	C1orf50
chr1	43875164	43875172	SZT2
chr1	44266953	44267044	ST3GAL3
chr1	44344699	44344717	ST3GAL3
chr1	44345007	44345052	ST3GAL3
chr1	44749658	44749741	ERI3
chr1	45287514	45287576	PTCH2
chr1	45406301	45406438	EIF2B3
chr1	45795738	45795740	MUTYH
chr1	45879506	45879542	TESK2
chr1	46054323	46054431	NASP
chr1	47048549	47048595	MKNK1
chr1	47060540	47060615	MKNK1
chr1	47099437	47099500	ATPAF1
chr1	47129569	47129595	ATPAF1
chr1	47129569	47129595	EFCAB14
chr1	47150755	47150816	EFCAB14
chr1	47150755	47150816	EFCAB14-AS1

chr1	47277849	47277943	CYP4B1
chr1	48569306	48569878	AL109659.1
chr1	48569306	48569878	SKINTL
chr1	48707616	48707693	SLC5A9
chr1	48858927	48858972	SPATA6
chr1	48866445	48866453	SPATA6
chr1	48866462	48866488	SPATA6
chr1	50460708	50461031	AL645730.2
chr1	50460708	50461031	AGBL4
chr1	50917758	50917865	FAF1
chr1	51078003	51078020	FAF1
chr1	51808679	51808750	TTC39A
chr1	51888880	51888950	EPS15
chr1	52135450	52135486	OSBPL9
chr1	52215174	52215212	OSBPL9
chr1	52260845	52260880	RP4-657D16.3
chr1	52260845	52260880	NRD1
chr1	52496208	52496220	TXNDC12
chr1	52526100	52526210	BTF3L4
chr1	52818845	52818895	CC2D1B
chr1	53379739	53379767	ECHDC2
chr1	53380939	53380964	ECHDC2
chr1	53383277	53383287	ECHDC2
chr1	53740372	53740492	LRP8
chr1	54356224	54356249	YIPF1
chr1	54514323	54514355	TMEM59
chr1	54636764	54637144	AL357673.1
chr1	54636764	54637144	RP11-446E24.4
chr1	54641289	54641333	RP11-446E24.4
chr1	54641289	54641333	CYB5RL
chr1	54686284	54686529	MRPL37
chr1	54703740	54704042	SSBP3-AS1
chr1	54703740	54704042	SSBP3
chr1	55124788	55124862	MROH7
chr1	55124788	55124862	MROH7-TTC4
chr1	55250400	55250549	TTC22
chr1	55516865	55516959	PCSK9
chr1	58939545	58939640	OMA1
chr1	58939545	58939640	DAB1
chr1	60163320	60163331	FGGY
chr1	60474320	60474413	C1orf87
chr1	61598367	61598383	NFIA
chr1	61905123	61905190	NFIA
chr1	62189996	62190097	TM2D1
chr1	63073438	63073806	AL138847.1
chr1	63073438	63073806	DOCK7
chr1	63301057	63301068	ATG4C

chr1	63913257	63913285	ITGB3BP
chr1	64014979	64015044	DLEU2L
chr1	64014979	64015044	EFCAB7
chr1	64014979	64015044	ITGB3BP
chr1	64015160	64015261	DLEU2L
chr1	64015160	64015261	EFCAB7
chr1	64015160	64015261	ITGB3BP
chr1	65720218	65720332	DNAJC6
chr1	66618211	66618215	PDE4B
chr1	67131894	67132439	AL139147.1
chr1	67131894	67132439	SGIP1
chr1	67313924	67313994	WDR78
chr1	67384790	67384799	WDR78
chr1	67474070	67474130	SLC35D1
chr1	67579429	67579439	C1orf141
chr1	68652271	68652332	GNG12-AS1
chr1	68652271	68652332	WLS
chr1	68693928	68693938	WLS
chr1	68697386	68697508	WLS
chr1	68948956	68948988	RP4-694A7.2
chr1	68948956	68948988	DEPDC1
chr1	70696240	70696298	SRSF11
chr1	70697542	70697591	SRSF11
chr1	71328052	71328080	PTGER3
chr1	71331372	71331516	PTGER3
chr1	71334927	71335002	PTGER3
chr1	71349632	71349766	PTGER3
chr1	71351961	71352011	PTGER3
chr1	74610124	74610229	LRRIQ3
chr1	74666376	74666431	FPGT
chr1	74666376	74666431	FPGT-TNNI3K
chr1	74666376	74666431	TNNI3K
chr1	74841249	74841288	FPGT-TNNI3K
chr1	74841249	74841288	TNNI3K
chr1	74841249	74841288	RP11-439H8.4
chr1	74867523	74867641	FPGT-TNNI3K
chr1	74867523	74867641	TNNI3K
chr1	74883852	74883861	FPGT-TNNI3K
chr1	74883852	74883861	TNNI3K
chr1	75088865	75088969	C1orf173
chr1	76234862	76234906	ACADM
chr1	78389895	78389927	NEXN
chr1	78997898	78997922	PTGFR
chr1	79355500	79355506	ELTD1
chr1	82446808	82446834	LPHN2
chr1	82453553	82453570	LPHN2
chr1	84352958	84352967	TTLL7

chr1	84810519	84810653	RP11-376N17.4
chr1	84810519	84810653	SAMD13
chr1	84853963	84854046	UOX
chr1	84853963	84854046	SAMD13
chr1	85096297	85096570	C1orf180
chr1	85097293	85097390	C1orf180
chr1	85115619	85115631	SSX2IP
chr1	85121148	85121271	SSX2IP
chr1	85502522	85502551	MCOLN3
chr1	85502522	85502551	WDR63
chr1	86201937	86202242	COL24A1
chr1	87599648	87599745	LINC01140
chr1	87599648	87599745	HS2ST1
chr1	87599648	87599745	RP5-1052I5.2
chr1	89294693	89294704	PKN2
chr1	89485809	89485894	GBP3
chr1	90171861	90171899	LRRC8C
chr1	90171861	90171899	RP11-302M6.4
chr1	92251761	92251784	TGFBR3
chr1	92266524	92266612	TGFBR3
chr1	93624372	93624442	TMED5
chr1	94484130	94484135	ABCA4
chr1	94511278	94511521	ABCA4
chr1	94620148	94620204	ARHGAP29
chr1	95291389	95291497	SLC44A3
chr1	96457908	96458065	RP11-147C23.1
chr1	96461677	96461750	RP11-147C23.1
chr1	96488089	96488171	RP11-147C23.1
chr1	98165760	98165858	DPYD
chr1	99128262	99128372	SNX7
chr1	101437833	101437853	SLC30A7
chr1	107946262	107946267	NTNG1
chr1	108996669	108996675	NBPF6
chr1	110027954	110028131	ATXN7L2
chr1	110164807	110164943	AMPD2
chr1	110452990	110453049	CSF1
chr1	110582446	110582503	STRIP1
chr1	111785839	111785844	CHI3L2
chr1	111833484	111833572	CHIA
chr1	111858356	111858494	CHIA
chr1	112032249	112032284	ADORA3
chr1	113229588	113229621	MOV10
chr1	113248227	113248261	RHOC
chr1	113248227	113248261	RP11-426L16.10
chr1	113257256	113257349	PPM1J
chr1	114406820	114407004	PTPN22
chr1	114406820	114407004	AP4B1-AS1

chr1	114695245	114695308	SYT6
chr1	116952757	116952778	ATP1A1OS
chr1	116952757	116952778	ATP1A1
chr1	118067711	118067781	MAN1A2
chr1	120839834	120839985	FAM72B
chr1	120841975	120842052	FAM72B
chr1	120845995	120846119	FAM72B
chr1	120927137	120927417	RP11-439A17.10
chr1	120927137	120927417	RP11-439A17.9
chr1	120927137	120927417	FCGR1B
chr1	120930039	120930293	RP11-439A17.10
chr1	120930039	120930293	RP11-439A17.9
chr1	120930039	120930293	FCGR1B
chr1	120935448	120935468	RP11-439A17.10
chr1	120935448	120935468	FCGR1B
chr1	120935864	120935894	FCGR1B
chr1	144146808	144147021	NBPF8
chr1	144148790	144148892	NBPF8
chr1	144149727	144149941	NBPF8
chr1	144150982	144151054	NBPF8
chr1	144151519	144151724	NBPF8
chr1	144153013	144153064	NBPF8
chr1	144156972	144157135	NBPF8
chr1	144158178	144158229	NBPF8
chr1	144158871	144159043	NBPF8
chr1	144161297	144161348	NBPF8
chr1	144162001	144162173	NBPF8
chr1	144162888	144162996	NBPF8
chr1	144164519	144164570	NBPF8
chr1	144165171	144165343	NBPF8
chr1	144166053	144166104	NBPF8
chr1	144166761	144166933	NBPF8
chr1	144167648	144167756	NBPF8
chr1	144169252	144169303	NBPF8
chr1	144170791	144170842	NBPF8
chr1	144171501	144171673	NBPF8
chr1	144172388	144172496	NBPF8
chr1	144173982	144174033	NBPF8
chr1	144175579	144175687	NBPF8
chr1	144176291	144176463	NBPF8
chr1	144177173	144177224	NBPF8
chr1	144181064	144181236	NBPF8
chr1	144181951	144182059	NBPF8
chr1	144182696	144182868	NBPF8
chr1	144183588	144183639	NBPF8
chr1	144184252	144184424	NBPF8
chr1	144185134	144185185	NBPF8

chr1	144185828	144186000	NBPF8
chr1	144186715	144186823	NBPF8
chr1	144189894	144189945	NBPF8
chr1	144190582	144190754	NBPF8
chr1	144191469	144191577	NBPF8
chr1	144193098	144193149	NBPF8
chr1	144193760	144193932	NBPF8
chr1	144194642	144194693	NBPF8
chr1	144195344	144195516	NBPF8
chr1	144196231	144196339	NBPF8
chr1	144198520	144198692	NBPF8
chr1	144199402	144199453	NBPF8
chr1	144200106	144200278	NBPF8
chr1	144200993	144201101	NBPF8
chr1	144201705	144201877	NBPF8
chr1	144202587	144202638	NBPF8
chr1	144203299	144203471	NBPF8
chr1	144204186	144204294	NBPF8
chr1	144204925	144205097	NBPF8
chr1	144205817	144205868	NBPF8
chr1	144206477	144206649	NBPF8
chr1	144207359	144207410	NBPF8
chr1	144209660	144209832	NBPF8
chr1	144210542	144210593	NBPF8
chr1	144211246	144211418	NBPF8
chr1	144214429	144214601	NBPF8
chr1	144215316	144215424	NBPF8
chr1	144216028	144216200	NBPF8
chr1	144216910	144216961	NBPF8
chr1	144218499	144218607	NBPF8
chr1	144221669	144221720	NBPF8
chr1	144222379	144222551	NBPF8
chr1	144223266	144223374	NBPF8
chr1	144224003	144224213	NBPF8
chr1	144520647	144520832	RP11-640M9.1
chr1	144520647	144520832	AL592284.1
chr1	144825783	144825788	NBPF9
chr1	144890975	144890989	PDE4DIP
chr1	144989641	144990021	AL590452.1
chr1	144989641	144990021	PDE4DIP
chr1	145017977	145018002	PDE4DIP
chr1	145038917	145038939	PDE4DIP
chr1	145074964	145075060	PDE4DIP
chr1	145314216	145314267	RP11-458D21.5
chr1	145314216	145314267	NBPF10
chr1	145329079	145329251	NBPF10
chr1	145359907	145359958	NBPF10

chr1	145361449	145361500	NBPF10
chr1	145362097	145362269	NBPF10
chr1	145362979	145363030	NBPF10
chr1	145363679	145363851	NBPF10
chr1	145364566	145364674	NBPF10
chr1	145577724	145577753	PIAS3
chr1	145883868	145883998	GPR89C
chr1	145884855	145884935	GPR89C
chr1	145897085	145897194	GPR89C
chr1	145898863	145898918	GPR89C
chr1	145899406	145899494	GPR89C
chr1	145900398	145900490	GPR89C
chr1	145916725	145916820	GPR89C
chr1	145918958	145919047	GPR89C
chr1	145923028	145923093	GPR89C
chr1	145923237	145923443	GPR89C
chr1	146034165	146034408	NBPF11
chr1	146034165	146034408	WI2-3658N16.1
chr1	146035862	146036030	NBPF11
chr1	146035862	146036030	WI2-3658N16.1
chr1	146036689	146036740	NBPF11
chr1	146036689	146036740	WI2-3658N16.1
chr1	146037450	146037622	NBPF11
chr1	146037450	146037622	WI2-3658N16.1
chr1	146038219	146038270	NBPF11
chr1	146038219	146038270	WI2-3658N16.1
chr1	146038990	146039162	NBPF11
chr1	146038990	146039162	WI2-3658N16.1
chr1	146039796	146039847	NBPF11
chr1	146039796	146039847	WI2-3658N16.1
chr1	146039874	146039923	NBPF11
chr1	146039874	146039923	WI2-3658N16.1
chr1	146040890	146041053	NBPF11
chr1	146040890	146041053	WI2-3658N16.1
chr1	146044949	146045000	NBPF11
chr1	146044949	146045000	WI2-3658N16.1
chr1	146046290	146046495	NBPF11
chr1	146046290	146046495	WI2-3658N16.1
chr1	146046960	146047032	NBPF11
chr1	146046960	146047032	WI2-3658N16.1
chr1	146048069	146048283	NBPF11
chr1	146048069	146048283	WI2-3658N16.1
chr1	146049117	146049219	NBPF11
chr1	146049117	146049219	WI2-3658N16.1
chr1	146050998	146051207	NBPF11
chr1	146050998	146051207	WI2-3658N16.1
chr1	146052552	146052763	NBPF11

chr1	146052552	146052763	WI2-3658N16.1
chr1	146053226	146053298	NBPF11
chr1	146053226	146053298	WI2-3658N16.1
chr1	146054299	146054513	NBPF11
chr1	146054299	146054513	WI2-3658N16.1
chr1	146055345	146055447	NBPF11
chr1	146055345	146055447	WI2-3658N16.1
chr1	146057275	146057484	NBPF11
chr1	146057275	146057484	WI2-3658N16.1
chr1	146066115	146066241	NBPF11
chr1	146066115	146066241	WI2-3658N16.1
chr1	146418506	146418563	NBPF12
chr1	146425676	146425727	NBPF12
chr1	146446186	146446237	NBPF12
chr1	147631967	147632428	BX842679.1
chr1	147631967	147632428	NBPF24
chr1	148007800	148007972	NBPF14
chr1	148010113	148010164	NBPF14
chr1	148257494	148257602	NBPF20
chr1	148259905	148260077	NBPF20
chr1	148260686	148260737	NBPF20
chr1	148262258	148262366	NBPF20
chr1	148263081	148263253	NBPF20
chr1	148265446	148265497	NBPF20
chr1	148276556	148276664	NBPF20
chr1	148277379	148277551	NBPF20
chr1	148282940	148283048	NBPF20
chr1	148283763	148283935	NBPF20
chr1	148284596	148284647	NBPF20
chr1	148288550	148288722	NBPF20
chr1	148289331	148289382	NBPF20
chr1	148294101	148294152	NBPF20
chr1	148302089	148302140	NBPF20
chr1	148303631	148303682	NBPF20
chr1	148309977	148310085	NBPF20
chr1	148311625	148311676	NBPF20
chr1	148319511	148319619	NBPF20
chr1	148320334	148320506	NBPF20
chr1	148321145	148321196	NBPF20
chr1	148322687	148322738	NBPF20
chr1	148323458	148323630	NBPF20
chr1	148739735	148739909	NBPF16
chr1	148741690	148741792	NBPF16
chr1	148742627	148742841	NBPF16
chr1	148743883	148743955	NBPF16
chr1	148744420	148744625	NBPF16
chr1	148745913	148745964	NBPF16

chr1	148749832	148749995	NBPF16
chr1	148751038	148751089	NBPF16
chr1	149450957	149451051	FAM72C
chr1	149459425	149459549	FAM72C
chr1	149553078	149553572	PPIAL4C
chr1	149765520	149765643	HIST2H2BF
chr1	149765520	149765643	RP11-196G18.21
chr1	150533557	150533946	ADAMTSL4-AS1
chr1	150666584	150666649	GOLPH3L
chr1	150720641	150720761	CTSS
chr1	150779725	150779900	CTSK
chr1	150825724	150825754	ARNT
chr1	150899254	150899284	SETDB1
chr1	151017356	151017365	BNIPL
chr1	151252672	151252734	RP11-126K1.2
chr1	151254019	151254135	RP11-126K1.2
chr1	151254019	151254135	ZNF687
chr1	151317917	151317919	RFX5
chr1	151682969	151683382	CELF3
chr1	151682969	151683382	RIIAD1
chr1	151682969	151683382	AL589765.1
chr1	151689231	151689274	CELF3
chr1	151689231	151689274	RIIAD1
chr1	151689231	151689274	AL589765.1
chr1	151754443	151754495	TDRKH
chr1	151862653	151862690	THEM4
chr1	153921365	153921376	CRTC2
chr1	154585067	154585080	AL606500.1
chr1	154585067	154585080	ADAR
chr1	154585184	154585190	AL606500.1
chr1	154585184	154585190	ADAR
chr1	155706399	155706485	DAP3
chr1	155706399	155706485	MSTO1
chr1	155706399	155706485	MSTO2P
chr1	155716587	155716726	MSTO1
chr1	155716587	155716726	MSTO2P
chr1	155716920	155716933	MSTO1
chr1	155716920	155716933	MSTO2P
chr1	155717354	155717741	MSTO1
chr1	155717354	155717741	MSTO2P
chr1	155942046	155942111	ARHGEF2
chr1	155982318	155982356	SSR2
chr1	155989024	155989057	SSR2
chr1	156190468	156190570	PMF1-BGLAP
chr1	156190468	156190570	PMF1
chr1	156217748	156217847	PAQR6
chr1	156219529	156219745	SMG5

chr1	156303007	156303075	CCT3
chr1	156306898	156307031	CCT3
chr1	156344398	156344504	RHBG
chr1	156389963	156390128	MIR9-1
chr1	156389963	156390128	C1orf61
chr1	157516003	157516070	FCRL5
chr1	158323265	158323316	CD1E
chr1	159879884	159880159	AL590560.1
chr1	160186655	160186698	DCAF8
chr1	160190890	160191000	DCAF8
chr1	160190890	160191000	DCAF8
chr1	160233055	160233252	DCAF8
chr1	160233055	160233252	DCAF8
chr1	160253921	160253930	DCAF8
chr1	160253921	160253930	DCAF8
chr1	160253921	160253930	PEX19
chr1	160313494	160313592	NCSTN
chr1	160585526	160585558	SLAMF1
chr1	160768616	160768719	LY9
chr1	161029958	161030052	ARHGAP30
chr1	161035655	161035697	AL591806.1
chr1	161035655	161035697	ARHGAP30
chr1	161035837	161035943	AL591806.1
chr1	161035837	161035943	ARHGAP30
chr1	161309352	161309376	SDHC
chr1	161720922	161720961	DUSP12
chr1	162330602	162330714	NOS1AP
chr1	162765411	162765453	HSD17B7
chr1	162770003	162770083	HSD17B7
chr1	166028072	166028467	AL626787.1
chr1	166028072	166028467	FAM78B
chr1	167051482	167051548	GPA33
chr1	167288251	167288271	POU2F1
chr1	167356418	167356438	POU2F1
chr1	167385286	167385326	POU2F1
chr1	167988783	167988823	DCAF6
chr1	169079869	169079941	ATP1B1
chr1	169159914	169159943	NME7
chr1	169216282	169216298	NME7
chr1	169795234	169795258	C1orf112
chr1	170502605	170502792	GORAB
chr1	170505451	170505507	GORAB
chr1	170984151	170984159	MROH9
chr1	171107351	171107545	FMO6P
chr1	171112310	171112498	FMO6P
chr1	171115496	171115658	FMO6P
chr1	171116765	171116907	FMO6P

chr1	171118699	171118898	FMO6P
chr1	171121049	171121404	FMO6P
chr1	171123281	171123353	FMO6P
chr1	171130202	171130204	FMO6P
chr1	172434747	172434805	C1orf105
chr1	176137820	176137884	RFWD2
chr1	178494506	178494603	TEX35
chr1	178499256	178499312	TEX35
chr1	178500667	178500693	TEX35
chr1	178514056	178514163	C1orf220
chr1	178514056	178514163	C1ORF220
chr1	178514056	178514163	TEX35
chr1	178514348	178514560	C1orf220
chr1	178514348	178514560	C1ORF220
chr1	178514348	178514560	TEX35
chr1	178514615	178515019	C1orf220
chr1	178514615	178515019	C1ORF220
chr1	178514615	178515019	TEX35
chr1	179418206	179418348	HNRNPA1P54
chr1	179418206	179418348	AXDND1
chr1	179443716	179443776	AL160286.1
chr1	179443716	179443776	AXDND1
chr1	179457738	179457805	AL160286.1
chr1	179457738	179457805	AXDND1
chr1	179852749	179852838	TOR1AIP1
chr1	179924239	179924277	CEP350
chr1	180941695	180941705	RP11-46A10.5
chr1	180941695	180941705	AL162431.1
chr1	180949712	180949868	AL162431.1
chr1	180949712	180949868	STX6
chr1	181714484	181714495	CACNA1E
chr1	182429759	182429891	RGSL1
chr1	182778320	182778419	NPL
chr1	182921072	182921119	SHCBP1L
chr1	183496761	183496831	SMG7
chr1	184027295	184027301	TSEN15
chr1	184039750	184039810	TSEN15
chr1	184760159	184760199	FAM129A
chr1	186344691	186344760	TPR
chr1	186364123	186364150	AL596220.1
chr1	186364123	186364150	C1orf27
chr1	186365764	186365811	AL596220.1
chr1	186365764	186365811	C1orf27
chr1	186365850	186365908	AL596220.1
chr1	186365850	186365908	C1orf27
chr1	196312186	196312235	KCNT2
chr1	197886489	197886672	LHX9

chr1	199998161	199998438	NR5A2
chr1	200007829	200007900	NR5A2
chr1	200026632	200026892	NR5A2
chr1	201084723	201084796	ASCL5
chr1	201346751	201346806	TNNT2
chr1	201346751	201346806	LAD1
chr1	202745233	202745348	KDM5B
chr1	202794635	202795252	RP11-480I12.4
chr1	203186493	203186500	CHIT1
chr1	203697279	203697362	ATP2B4
chr1	204116588	204116703	ETNK2
chr1	204435899	204436165	PIK3C2B
chr1	204507042	204507119	MDM4
chr1	204527769	204527809	MDM4
chr1	204915440	204915457	NFASC
chr1	204915838	204915852	RP11-494K3.2
chr1	204915838	204915852	NFASC
chr1	204962889	204962899	NFASC
chr1	205325661	205325697	KLHDC8A
chr1	205683471	205683719	AC119673.1
chr1	205683471	205683719	NUCKS1
chr1	206670927	206671050	C1orf147
chr1	207094686	207094778	FAIM3
chr1	207288021	207288049	C4BPA
chr1	207523483	207523496	CD55
chr1	207888657	207888663	CR1L
chr1	210330224	210330225	SYT14
chr1	211444870	211444877	RCOR3
chr1	211464866	211464941	RCOR3
chr1	211485697	211485719	RCOR3
chr1	212968088	212968150	TATDN3
chr1	213146766	213146783	VASH2
chr1	215754315	215754411	KCTD3
chr1	215791430	215791478	KCTD3
chr1	215803531	215803602	USH2A
chr1	217838621	217838623	SPATA17
chr1	218683438	218683529	C1orf143
chr1	218696695	218696792	C1orf143
chr1	218698749	218698870	C1orf143
chr1	220191468	220191488	EPRS
chr1	220384630	220384719	RAB3GAP2
chr1	220603286	220603370	AC096644.1
chr1	220607683	220607710	AC096644.1
chr1	220607984	220608023	AC096644.1
chr1	220927710	220927722	2-Mar
chr1	220982201	220982234	1-Mar
chr1	222821281	222821296	MIA3

chr1	222838225	222838280	MIA3
chr1	222886086	222886123	AIDA
chr1	222886086	222886123	BROX
chr1	223152964	223153009	DISP1
chr1	223532400	223532463	SUSD4
chr1	223533545	223533653	SUSD4
chr1	223711371	223711481	CAPN8
chr1	223712763	223712834	CAPN8
chr1	223712839	223712841	CAPN8
chr1	223830489	223830535	CAPN8
chr1	224215760	224215846	AC138393.1
chr1	224216016	224216040	AC138393.1
chr1	224216349	224216371	AC138393.1
chr1	224363631	224363649	DEGS1
chr1	224490642	224490723	NVL
chr1	225601576	225601923	AC092811.1
chr1	225601576	225601923	LBR
chr1	228247903	228248005	WNT3A
chr1	228351818	228352150	IBA57-AS1
chr1	228391754	228391856	C1orf145
chr1	228393088	228393148	C1orf145
chr1	229463283	229463305	CCSAP
chr1	229634941	229634967	NUP133
chr1	230498019	230498123	PGBD5
chr1	230503699	230503890	PGBD5
chr1	230797071	230797145	COG2
chr1	231071904	231072010	TTC13
chr1	231092296	231092306	TTC13
chr1	231353591	231353594	TRIM67
chr1	231355670	231355698	TRIM67
chr1	231480452	231480460	SPRTN
chr1	231910385	231910563	DISC1
chr1	231910385	231910563	TSNAX-DISC1
chr1	233248686	233248712	PCNXL2
chr1	233337896	233337996	PCNXL2
chr1	233785687	233785795	KCNK1
chr1	235319910	235319996	RBM34
chr1	235319910	235319996	ARID4B
chr1	235340205	235340218	ARID4B
chr1	236974945	236974964	MTR
chr1	237430900	237430933	RYR2
chr1	237496916	237496926	RYR2
chr1	237516141	237516155	RYR2
chr1	240408863	240408865	FMN2
chr1	240629183	240629225	AL646016.1
chr1	240629183	240629225	FMN2
chr1	240643479	240643504	AL646016.1

chr1	241756666	241756699	KMO
chr1	241756666	241756699	OPN3
chr1	241779156	241779238	OPN3
chr1	243340077	243340118	CEP170
chr1	243388058	243388102	AC092782.1
chr1	243388058	243388102	CEP170
chr1	243395894	243395911	AC092782.1
chr1	243395894	243395911	CEP170
chr1	243468314	243468446	SDCCAG8
chr1	244227916	244228404	AL590483.1
chr1	247143251	247143306	ZNF695
chr1	247143251	247143306	ZNF670
chr1	247690258	247690356	GCSAML
chr1	247701978	247702093	GCSAML
chr2	269301	269347	ACP1
chr2	676095	676238	TMEM18
chr2	1099091	1099128	SNTG2
chr2	1696301	1696342	PXDN
chr2	3322628	3322729	TSSC1
chr2	3356977	3357094	TSSC1
chr2	3466629	3466760	TRAPPC12
chr2	3488491	3488649	TRAPPC12
chr2	3605430	3605433	RNASEH1
chr2	3688830	3688852	COLEC11
chr2	6112057	6112536	DKFZP761K2322
chr2	6112057	6112536	LINC01105
chr2	7019148	7019172	RSAD2
chr2	8867023	8867073	KIDINS220
chr2	8889537	8889551	KIDINS220
chr2	8966041	8966043	KIDINS220
chr2	9002443	9002452	MBOAT2
chr2	9114488	9114564	MBOAT2
chr2	10085341	10085353	GRHL1
chr2	10093161	10093189	GRHL1
chr2	10223908	10224072	AC104794.4
chr2	10225272	10225336	AC104794.4
chr2	10226147	10226228	AC104794.4
chr2	10762217	10762276	NOL10
chr2	10994493	10994726	AC092687.4
chr2	10994989	10995036	AC092687.4
chr2	11295344	11295392	PQLC3
chr2	11312877	11312911	PQLC3
chr2	11595596	11595600	E2F6
chr2	11603927	11603937	AC099344.1
chr2	11603927	11603937	E2F6
chr2	11606250	11606274	AC099344.1
chr2	11606250	11606274	E2F6

chr2	15318270	15318281	NBAS
chr2	15533788	15533851	NBAS
chr2	15623481	15623492	NBAS
chr2	15840525	15840659	AC008271.1
chr2	15841353	15841499	AC008271.1
chr2	15883613	15883647	AC008271.1
chr2	15884350	15884396	AC008271.1
chr2	17894796	17894847	SMC6
chr2	17922012	17922089	SMC6
chr2	20100791	20100882	TTC32
chr2	20423952	20424041	SDC1
chr2	20526090	20526146	PUM2
chr2	20797980	20798025	HS1BP3
chr2	23729913	23730102	AC011239.1
chr2	23729913	23730102	KLHL29
chr2	23746821	23746961	AC011239.1
chr2	23746821	23746961	KLHL29
chr2	24055078	24055119	ATAD2B
chr2	24079279	24079312	ATAD2B
chr2	24256037	24256052	C2orf44
chr2	24256037	24256052	MFSD2B
chr2	24388242	24388303	FAM228B
chr2	24388242	24388303	RP11-507M3.1
chr2	24392225	24392268	AC008073.7
chr2	24392225	24392268	FAM228B
chr2	24392225	24392268	RP11-507M3.1
chr2	24401980	24402138	FAM228A
chr2	24401980	24402138	RP11-507M3.1
chr2	24423449	24423613	AC008073.9
chr2	24423449	24423613	FAM228A
chr2	24429116	24429144	ITSN2
chr2	24465074	24465075	ITSN2
chr2	25100791	25100799	ADCY3
chr2	25181438	25181470	DNAJC27
chr2	25622315	25622335	DTNB
chr2	26157013	26157025	KIF3C
chr2	26161166	26161217	KIF3C
chr2	26251074	26251481	AC013449.1
chr2	26671229	26671292	DRC1
chr2	27015370	27015420	CENPA
chr2	27291916	27291962	AGBL5
chr2	27549056	27549085	GTF3C2
chr2	27724557	27724680	GCKR
chr2	27732394	27732438	GCKR
chr2	27930448	27931032	AC074091.13
chr2	27998851	27998862	AC110084.1
chr2	27998851	27998862	MRPL33

chr2	28005439	28005561	AC110084.1
chr2	28005439	28005561	MRPL33
chr2	28005439	28005561	RBKS
chr2	28009122	28009148	AC110084.1
chr2	28009122	28009148	MRPL33
chr2	28009122	28009148	RBKS
chr2	28009163	28009186	AC110084.1
chr2	28009163	28009186	MRPL33
chr2	28009163	28009186	RBKS
chr2	28032847	28032912	MRPL33
chr2	28032847	28032912	RBKS
chr2	28084392	28084413	MRPL33
chr2	28084392	28084413	RBKS
chr2	28093655	28093757	MRPL33
chr2	28093655	28093757	RBKS
chr2	28456010	28456182	BRE
chr2	28771367	28771432	PLB1
chr2	29001217	29001311	PPP1CB
chr2	29152924	29153015	WDR43
chr2	29430910	29430912	ALK
chr2	30479568	30479570	LBH
chr2	30699268	30699358	LCLAT1
chr2	31355561	31355578	GALNT14
chr2	32412221	32412305	SLC30A6
chr2	32929928	32929984	TTC27
chr2	32991535	32991576	TTC27
chr2	33005121	33005162	TTC27
chr2	36605500	36605513	CRIM1
chr2	36756296	36756309	CRIM1
chr2	36759001	36759164	AC007401.2
chr2	36759001	36759164	CRIM1
chr2	36779199	36779262	AC007401.2
chr2	36779199	36779262	FEZ2
chr2	36779274	36779311	AC007401.2
chr2	36779274	36779311	FEZ2
chr2	36781398	36781405	FEZ2
chr2	36783120	36783203	FEZ2
chr2	37014932	37015022	VIT
chr2	37068327	37068530	AC007382.1
chr2	37195611	37195744	HEATR5B
chr2	37495444	37495515	PRKD3
chr2	37595785	37595802	QPCT
chr2	38581209	38581319	ATL2
chr2	38585667	38585830	ATL2
chr2	38805098	38805144	HNRNPLL
chr2	38827040	38827045	HNRNPLL
chr2	38907202	38907291	GALM

chr2	38968247	38968312	GALM
chr2	38974209	38974272	SRSF7
chr2	38976294	38976315	SRSF7
chr2	39117023	39117092	MORN2
chr2	39117023	39117092	ARHGEF33
chr2	39530256	39530258	MAP4K3
chr2	39984488	39984508	THUMPD2
chr2	40403117	40403126	SLC8A1-AS1
chr2	40403117	40403126	SLC8A1
chr2	40488147	40488178	AC007377.1
chr2	40488147	40488178	SLC8A1
chr2	40490099	40490117	AC007377.1
chr2	40490099	40490117	SLC8A1
chr2	40564465	40564468	SLC8A1
chr2	42560914	42561009	COX7A2L
chr2	42808925	42809010	MTA3
chr2	42979374	42979390	MTA3
chr2	43393800	43393905	THADA
chr2	43657785	43657789	THADA
chr2	43906656	43906739	PLEKHH2
chr2	44002848	44002927	DYNC2LI1
chr2	44008767	44008903	DYNC2LI1
chr2	44055509	44055607	ABCG5
chr2	44117494	44117501	LRPPRC
chr2	44512779	44512877	SLC3A1
chr2	46787663	46787828	RHOQ
chr2	46799867	46799923	RP11-417F21.1
chr2	46799867	46799923	RHOQ
chr2	47043964	47044051	LINC01118
chr2	47043964	47044051	LINC01119
chr2	47044387	47044572	LINC01118
chr2	47044387	47044572	LINC01119
chr2	47045319	47045370	LINC01118
chr2	47045319	47045370	LINC01119
chr2	47049240	47049297	LINC01118
chr2	47049240	47049297	LINC01119
chr2	47083062	47083129	LINC01119
chr2	47085225	47085349	LINC01119
chr2	47133059	47133121	AC016722.4
chr2	47133059	47133121	MCFD2
chr2	47178741	47178833	TTC7A
chr2	47799674	47800126	AC138655.1
chr2	48064127	48064175	FBXO11
chr2	48706623	48706695	PPP1R21
chr2	48948825	48948918	STON1-GTF2A1L
chr2	48948825	48948918	GTF2A1L
chr2	48948825	48948918	LHCGR

chr2	48960032	48960064	STON1-GTF2A1L
chr2	48960032	48960064	GTF2A1L
chr2	48960032	48960064	LHCGR
chr2	50246375	50246411	NRXN1
chr2	50966402	50966408	NRXN1
chr2	51125736	51125740	NRXN1
chr2	51150542	51150553	NRXN1
chr2	51206409	51206413	NRXN1
chr2	53831967	53832052	GPR75-ASB3
chr2	54144396	54144413	PSME4
chr2	54450134	54450155	ACYP2
chr2	54557303	54557346	C2orf73
chr2	58275713	58275722	VRK2
chr2	58285487	58285530	VRK2
chr2	61385013	61385147	RP11-493E12.1
chr2	61385013	61385147	C2orf74
chr2	61385013	61385147	KIAA1841
chr2	61386453	61386476	RP11-493E12.1
chr2	61386453	61386476	C2orf74
chr2	61386453	61386476	KIAA1841
chr2	62063941	62064007	FAM161A
chr2	62074087	62074131	FAM161A
chr2	62142817	62142867	COMMD1
chr2	62196104	62196220	COMMD1
chr2	62282575	62282592	COMMD1
chr2	62901221	62901228	EHBP1
chr2	63820757	63820762	MDH1
chr2	63820757	63820762	WDPCP
chr2	64089975	64089983	UGP2
chr2	64141033	64141059	VPS54
chr2	65332530	65332537	RAB1A
chr2	65333510	65333524	RAB1A
chr2	65604747	65604817	SPRED2
chr2	65604747	65604817	AC012370.2
chr2	66672325	66672428	MEIS1
chr2	66681841	66681920	MEIS1
chr2	66723158	66723304	MEIS1
chr2	66736059	66736255	MEIS1
chr2	66789214	66789251	MEIS1
chr2	68359136	68359138	WDR92
chr2	68359136	68359138	RP11-474G23.1
chr2	68388347	68388400	PNO1
chr2	68388347	68388400	RP11-474G23.1
chr2	68762415	68762453	APLF
chr2	69626485	69626514	NFU1
chr2	69657119	69657153	NFU1
chr2	70129482	70129603	AC019206.1

chr2	70129482	70129603	MXD1
chr2	70129482	70129603	SNRNP27
chr2	70129772	70129841	AC019206.1
chr2	70129772	70129841	MXD1
chr2	70129772	70129841	SNRNP27
chr2	70452489	70452525	TIA1
chr2	70452489	70452525	C2orf42
chr2	70676737	70676753	TGFA
chr2	71175500	71175505	ATP6V1B1
chr2	71175500	71175505	AC007040.11
chr2	71175500	71175505	AC007040.7
chr2	71602440	71602465	ZNF638
chr2	71635829	71635909	ZNF638
chr2	73441863	73441877	SMYD5
chr2	73497951	73498043	FBXO41
chr2	73689923	73689937	ALMS1
chr2	74000599	74000624	DUSP11
chr2	74135417	74135479	ACTG2
chr2	74159725	74159771	DGUOK
chr2	74361811	74361906	MGC10955
chr2	74418517	74418627	CATX-2
chr2	74607626	74607679	DCTN1
chr2	74617710	74617754	DCTN1
chr2	74617710	74617754	DCTN1-AS1
chr2	74645468	74645569	C2orf81
chr2	74645468	74645569	HMGA1P8
chr2	74882394	74882455	SEMA4F
chr2	75897705	75897749	MRPL19
chr2	75897705	75897749	GCFC2
chr2	80555424	80555474	CTNNA2
chr2	84676053	84676071	SUCLG1
chr2	85122209	85122233	AC022210.1
chr2	85122209	85122233	TRABD2A
chr2	85122317	85122345	AC022210.1
chr2	85122317	85122345	TRABD2A
chr2	85614221	85614247	RP11-717A5.2
chr2	85614221	85614247	ELMOD3
chr2	86078714	86078826	ST3GAL5
chr2	86079983	86080214	ST3GAL5
chr2	86097320	86097381	ST3GAL5
chr2	86334241	86334323	PTCD3
chr2	86507144	86507185	REEP1
chr2	87092511	87092525	AC111200.1
chr2	87092795	87092827	AC111200.1
chr2	89890601	89890616	IGKV2D-40
chr2	89986352	89986400	IGKV2D-29
chr2	89998825	89998873	IGKV2D-28

chr2	90458201	90458249	CH17-132F21.1
chr2	90458374	90458671	CH17-132F21.1
chr2	90537839	90538139	RP11-685N3.1
chr2	90538315	90538372	RP11-685N3.1
chr2	95538562	95538741	TEKT4
chr2	95538562	95538741	AC097374.2
chr2	95830776	95830817	ZNF514
chr2	96514587	96514602	ANKRD36C
chr2	96541715	96541725	ANKRD36C
chr2	96991942	96992046	ITPRIPL1
chr2	97259000	97259027	KANSL3
chr2	97281258	97281281	KANSL3
chr2	97293790	97293821	KANSL3
chr2	97294309	97294385	KANSL3
chr2	97299900	97299915	KANSL3
chr2	97402937	97402954	LMAN2L
chr2	97643554	97643669	FAM178B
chr2	97654025	97654061	FAM178B
chr2	97674734	97674921	FAM178B
chr2	97684092	97684175	AC079395.1
chr2	97684092	97684175	FAM178B
chr2	98795707	98795780	VWA3B
chr2	98858720	98858761	VWA3B
chr2	98948162	98948212	AC092675.3
chr2	98949677	98949767	AC092675.3
chr2	98949904	98949966	AC092675.3
chr2	98950386	98950414	AC092675.3
chr2	100067935	100067963	REV1
chr2	100247465	100247477	AFF3
chr2	100986705	100987007	AC012493.2
chr2	101031503	101031573	CHST10
chr2	101033451	101033491	CHST10
chr2	101640087	101640103	RPL31
chr2	101640087	101640103	TBC1D8
chr2	101889766	101889803	RNF149
chr2	102413726	102413845	MAP4K4
chr2	102509223	102509804	FLJ20373
chr2	102509223	102509804	MAP4K4
chr2	102957648	102957817	IL1RL1
chr2	102957648	102957817	IL18R1
chr2	105707780	105707824	MRPS9
chr2	105867759	105867773	AC012360.1
chr2	105869587	105869601	AC012360.1
chr2	105882490	105882585	AC012360.2
chr2	105882490	105882585	TGFBRAP1
chr2	105994566	105994574	AC012360.6
chr2	105994566	105994574	FHL2

chr2	106006564	106006684	FHL2
chr2	107029217	107029251	RGPD3
chr2	107099728	107099749	AC108868.1
chr2	108499618	108499652	RGPD4
chr2	109499127	109499203	CCDC138
chr2	110855076	110855181	MALL
chr2	110959982	110960056	NPHP1
chr2	112778181	112778231	MERTK
chr2	113303408	113303452	POLR1B
chr2	114204992	114205429	AC016745.1
chr2	114204992	114205429	CBWD2
chr2	118702309	118702373	CCDC93
chr2	120124012	120124086	C2orf76
chr2	120187942	120187979	TMEM37
chr2	120313613	120313777	PCDP1
chr2	120314584	120314616	PCDP1
chr2	120337756	120337780	PCDP1
chr2	120347181	120347227	PCDP1
chr2	120360194	120360212	PCDP1
chr2	120406019	120406067	PCDP1
chr2	121223153	121223686	LINC01101
chr2	122227842	122227853	CLASP1
chr2	122494679	122494761	TSN
chr2	128227926	128227961	IWS1
chr2	128227926	128227961	AC010976.2
chr2	128860653	128860668	UGGT1
chr2	131097184	131097335	CCDC115
chr2	131114173	131114226	PTPN18
chr2	131328413	131328553	AC140481.2
chr2	132044682	132045038	CYP4F31P
chr2	132044682	132045038	PLEKHB2
chr2	132911126	132911267	ANKRD30BL
chr2	133875457	133875834	AC011755.1
chr2	133875457	133875834	NCKAP5
chr2	133881356	133881371	NCKAP5
chr2	136028885	136028965	ZRANB3
chr2	136505228	136505335	UBXN4
chr2	137664018	137664031	THSD7B
chr2	138237045	138237053	THSD7B
chr2	141026837	141026950	LRP1B
chr2	144345255	144345346	ARHGAP15
chr2	144345255	144345346	RP11-570L15.1
chr2	144936506	144936527	GTDC1
chr2	145188105	145188137	ZEB2
chr2	149791569	149791574	KIF5C
chr2	150016990	150016994	LYPD6B
chr2	150062533	150062540	LYPD6B

chr2	150320678	150320691	LYPD6
chr2	151857842	151857904	AC023469.2
chr2	151857842	151857904	AC023469.1
chr2	151890323	151890405	AC023469.1
chr2	151900283	151900387	AC023469.1
chr2	151904193	151904217	AC023469.1
chr2	152266470	152266547	RIF1
chr2	152667070	152667075	ARL5A
chr2	153549982	153549990	PRPF40A
chr2	153576412	153576461	ARL6IP6
chr2	153589045	153589085	ARL6IP6
chr2	155092559	155092562	GALNT13
chr2	157352074	157352142	GPD2
chr2	157467236	157467239	GPD2
chr2	158295464	158295475	CYTIP
chr2	159145301	159145348	CCDC148
chr2	159434681	159434723	PKP4
chr2	159718921	159719218	OR7E28P
chr2	159718921	159719218	DAPL1
chr2	162059374	162059385	TANK
chr2	162072900	162072950	TANK
chr2	162076235	162076294	TANK
chr2	162758538	162758595	SLC4A10
chr2	163054801	163054808	FAP
chr2	163078207	163078222	FAP
chr2	165560171	165560181	COBLL1
chr2	165630217	165630264	COBLL1
chr2	165636165	165636271	COBLL1
chr2	165693938	165693999	COBLL1
chr2	167292857	167292950	SCN7A
chr2	169674682	169674806	NOSTRIN
chr2	169712396	169712418	NOSTRIN
chr2	169712396	169712418	SPC25
chr2	170440877	170441000	PPIG
chr2	170629830	170629867	KLHL23
chr2	170632951	170632982	KLHL23
chr2	170821380	170821476	UBR3
chr2	170873343	170873429	UBR3
chr2	171503419	171503449	AC007277.3
chr2	171503419	171503449	MYO3B
chr2	171570141	171570953	LINC01124
chr2	171786251	171786409	GORASP2
chr2	171793134	171793237	GORASP2
chr2	171879634	171879639	TLK1
chr2	171938198	171938218	TLK1
chr2	172303896	172304013	DCAF17
chr2	172329380	172329465	DCAF17

chr2	172379747	172379822	CYBRD1
chr2	172560844	172560883	AC068039.1
chr2	172560844	172560883	DYNC1I2
chr2	172567289	172567314	AC068039.1
chr2	172567289	172567314	DYNC1I2
chr2	172702279	172702368	SLC25A12
chr2	172709178	172709198	SLC25A12
chr2	173428780	173428786	PDK1
chr2	175223173	175223264	CIR1
chr2	175234715	175234742	CIR1
chr2	175686457	175686507	CHN1
chr2	175712447	175712473	CHN1
chr2	175809979	175809989	CHN1
chr2	175957342	175957344	ATF2
chr2	176796880	176796890	KIAA1715
chr2	177168969	177168971	MTX2
chr2	178092634	178092681	NFE2L2
chr2	178175692	178175733	AC074286.1
chr2	178175692	178175733	NFE2L2
chr2	178764294	178764317	PDE11A
chr2	178990452	178990478	RBM45
chr2	179310261	179310263	PRKRA
chr2	179323920	179323927	DFNB59
chr2	179337859	179337869	FKBP7
chr2	179353695	179353723	PLEKHA3
chr2	179367063	179367205	PLEKHA3
chr2	180039159	180039185	SESTD1
chr2	183995723	183995794	NUP35
chr2	187361840	187361857	AC018867.1
chr2	187361840	187361857	ZC3H15
chr2	187364346	187364389	AC018867.1
chr2	187364346	187364389	ZC3H15
chr2	187365355	187365393	AC018867.1
chr2	187365355	187365393	ZC3H15
chr2	187372122	187372201	ZC3H15
chr2	190313971	190314048	WDR75
chr2	190642909	190642936	AC013468.1
chr2	190642909	190642936	ORMDL1
chr2	190643138	190643157	AC013468.1
chr2	190643138	190643157	ORMDL1
chr2	190722211	190722230	PMS1
chr2	191115101	191115103	HIBCH
chr2	191116261	191116284	HIBCH
chr2	191208718	191208914	HIBCH
chr2	191208718	191208914	INPP1
chr2	191840099	191840114	STAT1
chr2	197584898	197584902	CCDC150

chr2	197638393	197638450	GTF3C3
chr2	197786905	197786910	PGAP1
chr2	198012593	198012628	ANKRD44
chr2	198263693	198263752	SF3B1
chr2	198381334	198381457	HSPD1
chr2	198381334	198381457	HSPE1-MOB4
chr2	198381334	198381457	MOB4
chr2	198557835	198557940	AC011997.1
chr2	198563534	198563588	AC011997.1
chr2	198638740	198638781	BOLL
chr2	198638740	198638781	AC011997.1
chr2	200262529	200262563	SATB2
chr2	200870466	200870500	C2orf47
chr2	200873189	200873203	C2orf47
chr2	201257973	201257996	SPATS2L
chr2	201325561	201325718	SPATS2L
chr2	201677607	201677608	BZW1
chr2	201753679	201753738	PPIL3
chr2	201886782	201886837	FAM126B
chr2	202007368	202007485	CFLAR-AS1
chr2	202007368	202007485	CFLAR
chr2	202499600	202499616	TMEM237
chr2	202526174	202526187	MPP4
chr2	202526278	202526294	MPP4
chr2	202531320	202531444	MPP4
chr2	202538252	202538279	MPP4
chr2	202563310	202563331	MPP4
chr2	202584759	202584851	ALS2
chr2	202941655	202941822	AC079354.1
chr2	202990551	202990624	AC079354.1
chr2	203096266	203096455	AC079354.6
chr2	203096266	203096455	SUMO1
chr2	203104292	203104435	AC079354.2
chr2	203142935	203142966	NOP58
chr2	203651422	203651498	ICA1L
chr2	203838205	203838223	WDR12
chr2	203838205	203838223	CARF
chr2	204090846	204090873	NBEAL1
chr2	204206920	204206953	ABI2
chr2	204216278	204216388	ABI2
chr2	204219877	204219939	ABI2
chr2	204239543	204239575	ABI2
chr2	207220463	207220843	AC017081.1
chr2	208436458	208436492	CREB1
chr2	208626361	208626405	CCNYL1
chr2	208717514	208717559	PLEKHM3
chr2	209436738	209436794	PTH2R

chr2	210568711	210568758	MAP2
chr2	210868162	210868192	RPE
chr2	211336320	211336369	LANCL1
chr2	213886437	213886444	IKZF2
chr2	213962665	213962684	IKZF2
chr2	214150610	214150642	SPAG16
chr2	215595632	215595647	BARD1
chr2	216212344	216212361	ATIC
chr2	216213407	216213454	ATIC
chr2	217311385	217311441	SMARCAL1
chr2	217735570	217735703	AC007563.1
chr2	217735570	217735703	AC007557.1
chr2	217735570	217735703	AC007563.5
chr2	217736179	217736338	AC007563.1
chr2	217736179	217736338	AC007557.1
chr2	217736179	217736338	AC007563.5
chr2	218744168	218744235	TNS1
chr2	218770019	218770168	TNS1
chr2	218801698	218801703	TNS1
chr2	218867563	218867595	TNS1
chr2	219091719	219091726	ARPC2
chr2	219099572	219099579	ARPC2
chr2	219117668	219117755	ARPC2
chr2	219297238	219297285	VIL1
chr2	219438718	219438819	RQCD1
chr2	219553114	219553158	STK36
chr2	219959996	219960077	NHEJ1
chr2	219959996	219960077	SLC23A3
chr2	220117501	220117509	TUBA4A
chr2	220117718	220117789	TUBA4A
chr2	220173656	220173699	PTPRN
chr2	220264162	220264222	DNPEP
chr2	220264162	220264222	AC053503.4
chr2	220306280	220306316	SPEG
chr2	220408809	220409099	TMEM198
chr2	222367248	222367280	EPHA4
chr2	223785672	223785786	ACSL3
chr2	224638045	224638068	AP1S3
chr2	225649454	225649459	DOCK10
chr2	225758357	225758369	DOCK10
chr2	225848521	225848526	DOCK10
chr2	228373340	228373366	AGFG1
chr2	228741159	228741210	DAW1
chr2	228776970	228777106	DAW1
chr2	231099653	231099681	SP140
chr2	231334166	231334167	SP100
chr2	232153373	232153381	ARMC9

chr2	232317248	232317521	AC017104.2
chr2	232317795	232317864	AC017104.2
chr2	232571517	232571621	MGC4771
chr2	232571517	232571621	PTMA
chr2	232656764	232656792	COPS7B
chr2	232673131	232673285	COPS7B
chr2	232975907	232975965	DIS3L2
chr2	233200851	233200967	DIS3L2
chr2	233205434	233205640	DIS3L2
chr2	233503116	233503122	EFHD1
chr2	233630665	233630689	AC064852.4
chr2	233630665	233630689	GIGYF2
chr2	233653243	233653300	GIGYF2
chr2	233740138	233740265	C2orf82
chr2	233755644	233755690	NGEF
chr2	233770355	233770559	NGEF
chr2	233877562	233877679	AC106876.2
chr2	233877562	233877679	NGEF
chr2	233877850	233877957	AC106876.2
chr2	233877850	233877957	NGEF
chr2	233880417	233880451	AC106876.2
chr2	233998690	233998706	INPP5D
chr2	233998840	233998870	INPP5D
chr2	234053919	234053927	INPP5D
chr2	234363003	234363110	DGKD
chr2	234598545	234598550	UGT1A8
chr2	234598545	234598550	UGT1A10
chr2	234598545	234598550	UGT1A9
chr2	234598545	234598550	UGT1A7
chr2	234599166	234599207	UGT1A8
chr2	234599166	234599207	UGT1A10
chr2	234599166	234599207	UGT1A9
chr2	234599166	234599207	UGT1A7
chr2	234626439	234626441	UGT1A5
chr2	234626439	234626441	UGT1A8
chr2	234626439	234626441	UGT1A10
chr2	234626439	234626441	UGT1A9
chr2	234626439	234626441	UGT1A7
chr2	234626439	234626441	UGT1A6
chr2	234856221	234856226	AC005538.5
chr2	234856221	234856226	TRPM8
chr2	236433180	236433183	AGAP1
chr2	236682576	236682626	AC064874.1
chr2	236682576	236682626	AGAP1
chr2	236682947	236683066	AC064874.1
chr2	236682947	236683066	AGAP1
chr2	236685601	236685685	AC064874.1

chr2	236685601	236685685	AGAP1
chr2	236691958	236692031	AC064874.1
chr2	236691958	236692031	AGAP1
chr2	236954581	236954675	AGAP1
chr2	237252345	237252406	IQCA1
chr2	237996107	237996152	COPS8
chr2	238165759	238165836	AC112715.2
chr2	238166073	238166177	AC112715.2
chr2	238295227	238295252	COL6A3
chr2	238330183	238330483	AC112721.1
chr2	238330768	238330787	AC112721.1
chr2	238499812	238499910	RAB17
chr2	238499812	238499910	AC104667.3
chr2	238500515	238500674	RAB17
chr2	238500515	238500674	AC104667.3
chr2	238503583	238503588	RAB17
chr2	238503583	238503588	AC104667.3
chr2	238882291	238882316	UBE2F
chr2	238882291	238882316	UBE2F-SCLY
chr2	238894804	238894832	UBE2F
chr2	238894804	238894832	UBE2F-SCLY
chr2	238899066	238899109	UBE2F
chr2	238899066	238899109	UBE2F-SCLY
chr2	238912010	238912056	UBE2F
chr2	238912010	238912056	UBE2F-SCLY
chr2	238982424	238982488	SCLY
chr2	238982424	238982488	UBE2F-SCLY
chr2	239007871	239007994	SCLY
chr2	239007871	239007994	UBE2F-SCLY
chr2	239077012	239077128	FAM132B
chr2	239133774	239134140	AC016757.3
chr2	239136325	239136439	AC016757.3
chr2	239139842	239139860	AC016757.3
chr2	239149033	239149303	HES6
chr2	239149033	239149303	AC096574.4
chr2	239192329	239192351	AC012485.1
chr2	239192329	239192351	PER2
chr2	239192447	239192498	AC012485.1
chr2	239192447	239192498	PER2
chr2	240062574	240062669	HDAC4
chr2	240083493	240083504	AC017028.1
chr2	240083493	240083504	HDAC4
chr2	240083802	240084053	AC017028.1
chr2	240083802	240084053	HDAC4
chr2	240112674	240112676	HDAC4
chr2	240323442	240323663	AC062017.1
chr2	240323769	240323940	AC062017.1

chr2	240684580	240684626	AC093802.1
chr2	240701998	240702119	AC093802.1
chr2	240721535	240721542	AC093802.1
chr2	240834636	240834731	NDUFA10
chr2	241053907	241053937	AC013469.1
chr2	241064285	241064304	AC013469.1
chr2	241389247	241389338	AC110619.2
chr2	241389247	241389338	GPC1
chr2	241390028	241390339	AC110619.2
chr2	241390028	241390339	AC110619.1
chr2	241390028	241390339	GPC1
chr2	241390558	241390748	AC110619.2
chr2	241390558	241390748	AC110619.1
chr2	241390558	241390748	GPC1
chr2	241392228	241392511	AC110619.2
chr2	241392228	241392511	GPC1
chr2	241394422	241394437	AC110619.2
chr2	241394422	241394437	GPC1
chr2	241396014	241396080	AC110619.2
chr2	241396014	241396080	GPC1
chr2	241396851	241396886	GPC1
chr2	241397382	241397534	GPC1
chr2	241471493	241471552	ANKMY1
chr2	241531896	241531915	CAPN10
chr2	241535351	241535407	CAPN10
chr2	241624478	241624705	AC011298.1
chr2	241688082	241688135	KIF1A
chr2	241869102	241869113	AC104809.3
chr2	241980303	241980316	SNED1
chr2	241980303	241980316	AC005237.4
chr2	242078967	242079010	PASK
chr2	242090889	242090967	PPP1R7
chr2	242122794	242122878	PPP1R7
chr2	242188773	242188811	HDLBP
chr2	242259661	242259702	2-Sep
chr2	242443453	242443511	STK25
chr2	242447858	242447873	STK25
chr2	242621643	242621748	DTYMK
chr2	242754694	242754807	NEU4
chr2	242755286	242755405	NEU4
chr2	242756867	242756886	NEU4
chr2	242813143	242813152	CXXC11
chr2	242841266	242841289	AC131097.4
chr2	242841266	242841289	AC131097.3
chr3	239330	239611	CHL1
chr3	1134420	1134611	CNTN6
chr3	1214888	1214969	CNTN6

chr3	2671210	2671263	CNTN4
chr3	2968982	2969021	CNTN4
chr3	3178360	3178409	TRNT1
chr3	3869104	3869193	LRRN1
chr3	3869104	3869193	SUMF1
chr3	4867430	4867546	AC018816.3
chr3	4867430	4867546	ITPR1
chr3	4872631	4872702	AC018816.3
chr3	4872631	4872702	ITPR1
chr3	4927407	4927450	AC018816.3
chr3	5170145	5170158	ARL8B
chr3	6811740	6811805	GRM7-AS3
chr3	6811740	6811805	GRM7
chr3	6828279	6828373	GRM7-AS3
chr3	6828279	6828373	GRM7
chr3	6870318	6870363	GRM7
chr3	6998254	6998306	GRM7
chr3	7444865	7444868	GRM7
chr3	7732726	7732769	GRM7
chr3	7732897	7732934	GRM7
chr3	8701427	8701487	SSUH2
chr3	8757379	8757572	SSUH2
chr3	8784145	8784208	SSUH2
chr3	8784145	8784208	CAV3
chr3	8817251	8817350	CAV3
chr3	8817251	8817350	RAD18
chr3	9471511	9471598	SETD5
chr3	9520285	9520296	SETD5
chr3	9701406	9701493	MTMR14
chr3	9717289	9717355	MTMR14
chr3	9829412	9829415	OGG1
chr3	9829412	9829415	TADA3
chr3	9841563	9841575	ARPC4
chr3	9841563	9841575	ARPC4-TTLL3
chr3	9845220	9845231	ARPC4
chr3	9845220	9845231	ARPC4-TTLL3
chr3	9857758	9857888	ARPC4-TTLL3
chr3	9857758	9857888	TTLL3
chr3	9860239	9860291	ARPC4-TTLL3
chr3	9860239	9860291	TTLL3
chr3	10144675	10144773	FANCD2OS
chr3	11518608	11518616	ATG7
chr3	11667831	11667882	VGLL4
chr3	11734606	11734637	VGLL4
chr3	11874581	11874625	TAMM41
chr3	12413350	12413390	PPARG
chr3	12440847	12440872	PPARG

chr3	12892158	12892359	AC034198.7
chr3	12892158	12892359	CAND2
chr3	12913235	12913257	CAND2
chr3	13028471	13028536	IQSEC1
chr3	13522215	13522273	HDAC11
chr3	13573971	13574007	FBLN2
chr3	14105677	14106435	TPRXL
chr3	14170355	14170543	TMEM43
chr3	14486246	14486355	SLC6A6
chr3	15132981	15133000	ZFYVE20
chr3	15252309	15252317	CAPN7
chr3	15509275	15509416	COLQ
chr3	15855799	15855816	ANKRD28
chr3	16357362	16357393	OXNAD1
chr3	16357362	16357393	RFTN1
chr3	16457697	16457718	RFTN1
chr3	16679392	16679508	DAZL
chr3	17122680	17122736	PLCL2
chr3	19490420	19490503	KCNH8
chr3	20012040	20012074	RAB5A
chr3	20025748	20025788	RAB5A
chr3	20025748	20025788	PP2D1
chr3	21553214	21553249	ZNF385D
chr3	23365006	23365077	UBE2E2
chr3	23929729	23929752	UBE2E1
chr3	24004715	24004795	NR1D2
chr3	25900180	25900289	LINC00692
chr3	25902182	25902243	LINC00692
chr3	25904479	25904544	LINC00692
chr3	25904776	25904870	LINC00692
chr3	27464272	27464331	SLC4A7
chr3	28502459	28502512	ZCWPW2
chr3	28519780	28519878	ZCWPW2
chr3	28552494	28552618	ZCWPW2
chr3	28578602	28578742	ZCWPW2
chr3	28579373	28579429	ZCWPW2
chr3	32608105	32608227	DYNC1LI1
chr3	32806542	32806610	CNOT10
chr3	33344566	33344649	FBXL2
chr3	33358969	33359001	FBXL2
chr3	33556157	33556171	CLASP2
chr3	33852589	33852616	PDCD6IP
chr3	33876746	33876769	PDCD6IP
chr3	37953164	37953252	CTDSPL
chr3	38009707	38009835	CTDSPL
chr3	38024057	38024134	CTDSPL
chr3	38025739	38025757	CTDSPL

chr3	38042413	38042430	VILL
chr3	38540417	38540480	EXOG
chr3	38541172	38541226	EXOG
chr3	38583258	38583437	EXOG
chr3	39143001	39143061	GORASP1
chr3	39148484	39148666	GORASP1
chr3	39448319	39448573	RPSA
chr3	39541530	39541558	MOBP
chr3	40435800	40435922	ENTPD3
chr3	40435800	40435922	ENTPD3-AS1
chr3	42192827	42192897	TRAK1
chr3	42607372	42607375	SEC22C
chr3	42608292	42608328	SEC22C
chr3	44868488	44868530	KIF15
chr3	44961695	44961829	ZDHHC3
chr3	45956913	45957110	LZTFL1
chr3	46502055	46502087	LTF
chr3	46599303	46599818	LUZPP1
chr3	46599303	46599818	LRRC2
chr3	46927699	46927728	AC109583.1
chr3	46927699	46927728	PTH1R
chr3	46930133	46930171	AC109583.1
chr3	46930133	46930171	PTH1R
chr3	47106104	47106120	SETD2
chr3	47161243	47161270	SETD2
chr3	47339052	47339114	KLHL18
chr3	47544837	47544939	ELP6
chr3	48341767	48341780	NME6
chr3	48491899	48491937	ATRIP
chr3	48518443	48518500	SHISA5
chr3	48539776	48539885	SHISA5
chr3	48540568	48540626	SHISA5
chr3	48596933	48597038	PFKFB4
chr3	48608919	48608981	COL7A1
chr3	48701364	48701567	RP11-148G20.1
chr3	48701364	48701567	NCKIPSD
chr3	48933188	48933248	SLC25A20
chr3	49036922	49036986	P4HTM
chr3	49046342	49046445	WDR6
chr3	49140136	49140165	QARS
chr3	49265751	49265776	CCDC36
chr3	49298046	49298486	RP11-3B7.1
chr3	49314619	49314643	C3orf62
chr3	49410673	49410775	RHOA
chr3	49941498	49941523	CTD-2330K9.3
chr3	49953875	49954046	CTD-2330K9.3
chr3	49953875	49954046	MON1A

chr3	50130412	50130474	RBM5
chr3	50130412	50130474	RBM6
chr3	50330324	50330349	IFRD2
chr3	50330324	50330349	HYAL3
chr3	50364599	50364655	TUSC2
chr3	50599939	50600019	C3orf18
chr3	51424340	51424345	MANF
chr3	52097076	52097567	LINC00696
chr3	52781346	52781467	NEK4
chr3	52870640	52870651	TMEM110-MUSTN1
chr3	52870640	52870651	TMEM110
chr3	52881518	52881567	TMEM110-MUSTN1
chr3	52881518	52881567	TMEM110
chr3	52929934	52929971	TMEM110-MUSTN1
chr3	52929934	52929971	TMEM110
chr3	53112200	53112227	RP11-894J14.5
chr3	53277599	53277638	TKT
chr3	53744422	53744505	CACNA1D
chr3	53830368	53830427	CACNA1D
chr3	56594890	56594970	CCDC66
chr3	56595285	56595299	CCDC66
chr3	56603341	56603390	CCDC66
chr3	56932600	56932666	ARHGEF3
chr3	57009790	57009848	ARHGEF3
chr3	57176481	57176534	IL17RD
chr3	58138435	58138508	FLNB
chr3	58244130	58244166	ABHD6
chr3	58319277	58319351	PXK
chr3	58496706	58496781	ACOX2
chr3	58703315	58703355	C3orf67
chr3	58720281	58720285	C3orf67
chr3	58722914	58722951	C3orf67
chr3	58780051	58780057	C3orf67
chr3	58898443	58898468	RP11-147N17.1
chr3	58898443	58898468	C3orf67
chr3	59957215	59957520	NPCDR1
chr3	59957215	59957520	FHIT
chr3	59957569	59957583	NPCDR1
chr3	59957569	59957583	FHIT
chr3	62536520	62536564	CADPS
chr3	63831862	63831893	C3orf49
chr3	63831862	63831893	THOC7
chr3	65583356	65583410	MAGI1
chr3	65583490	65583561	MAGI1
chr3	66354877	66354888	SLC25A26
chr3	66406455	66406481	SLC25A26
chr3	69138963	69138966	ARL6IP5

chr3	69149123	69149220	ARL6IP5
chr3	69277531	69277542	FRMD4B
chr3	69277739	69277749	FRMD4B
chr3	69342124	69342212	FRMD4B
chr3	69359589	69359668	FRMD4B
chr3	77613601	77613613	ROBO2
chr3	78683578	78683730	ROBO1
chr3	79582222	79582259	ROBO1
chr3	97278435	97278499	EPHA6
chr3	97331222	97331275	EPHA6
chr3	97464203	97464224	EPHA6
chr3	97643241	97643255	CRYBG3
chr3	97651956	97651967	CRYBG3
chr3	97671039	97671096	MINA
chr3	98234609	98234676	CLDND1
chr3	98281238	98281286	CPOX
chr3	98475213	98475262	ST3GAL6
chr3	99549323	99549449	CMSS1
chr3	99549323	99549449	FILIP1L
chr3	99860374	99860426	CMSS1
chr3	99878740	99878760	CMSS1
chr3	99904531	99904722	TMEM30C
chr3	99908785	99908934	TMEM30C
chr3	99986082	99986092	TBC1D23
chr3	100633272	100633308	ABI3BP
chr3	100664438	100664520	ABI3BP
chr3	101481953	101482030	CEP97
chr3	105468568	105468609	CBLB
chr3	107430122	107430211	BBX
chr3	107774367	107774413	CD47
chr3	110607766	110607773	RP11-553A10.1
chr3	110611076	110611450	RP11-553A10.1
chr3	110611985	110612209	RP11-553A10.1
chr3	110789158	110789176	PVRL3-AS1
chr3	110789158	110789176	PVRL3
chr3	110994160	110994192	PVRL3
chr3	111766255	111766292	TMPRSS7
chr3	112556834	112556857	CD200R1L
chr3	112560094	112560191	CD200R1L
chr3	113012862	113012885	WDR52
chr3	113499579	113499630	ATP6V1A
chr3	116728077	116728221	LSAMP
chr3	116858048	116858080	LSAMP
chr3	117716029	117716095	LSAMP
chr3	117716029	117716095	RP11-384F7.2
chr3	118878740	118878775	C3orf30
chr3	118878740	118878775	RP11-484M3.5

chr3	119043793	119043805	ARHGAP31
chr3	119154752	119154782	TMEM39A
chr3	119170144	119170164	TMEM39A
chr3	119197181	119197288	POGLUT1
chr3	119338923	119338987	PLA1A
chr3	121387308	121387355	GOLGB1
chr3	122176579	122176695	KPNA1
chr3	122477619	122477700	HSPBAP1
chr3	123218483	123218487	PTPLB
chr3	123798870	123798939	KALRN
chr3	123801100	123801179	KALRN
chr3	124223750	124223797	KALRN
chr3	124396411	124396454	KALRN
chr3	124576079	124576160	ITGB5
chr3	124689978	124689992	HEG1
chr3	125786424	125786429	SLC41A3
chr3	125878393	125878409	ALDH1L1
chr3	126327984	126328031	TXNRD3
chr3	126499183	126499194	CHCHD6
chr3	127329622	127329828	MCM2
chr3	127398416	127398463	ABTB1
chr3	127787753	127787756	SEC61A1
chr3	127787753	127787756	RUVBL1
chr3	128292123	128292572	C3orf27
chr3	128363450	128363489	RPN1
chr3	128862958	128863032	ISY1
chr3	128862958	128863032	ISY1-RAB43
chr3	128992907	128992918	COPG1
chr3	129174957	129175006	IFT122
chr3	129176765	129176820	IFT122
chr3	129178158	129178263	IFT122
chr3	129279877	129279899	PLXND1
chr3	130308818	130308880	COL6A6
chr3	130365223	130365235	COL6A6
chr3	130852121	130852199	NEK11
chr3	132077261	132077367	ACPP
chr3	132256002	132256060	DNAJC13
chr3	132436912	132436961	NPHP3
chr3	133493723	133493783	TF
chr3	133794023	133794057	RYK
chr3	134696808	134696848	EPHB1
chr3	135797992	135798054	PPP2R3A
chr3	135970085	135970134	PCCB
chr3	135971145	135971231	PCCB
chr3	136310383	136310391	STAG1
chr3	136688115	136688220	IL20RB-AS1
chr3	136688115	136688220	IL20RB

chr3	136705536	136705588	IL20RB
chr3	138043258	138043436	NME9
chr3	138881971	138882052	MRPS22
chr3	139068170	139068175	RP11-219D15.3
chr3	139068170	139068175	MRPS22
chr3	139294833	139294836	RP11-319G6.1
chr3	139294833	139294836	NMNAT3
chr3	140685190	140685215	SLC25A36
chr3	140685776	140685810	SLC25A36
chr3	140685842	140685855	SLC25A36
chr3	140851031	140851140	SPSB4
chr3	140981085	140981086	ACPL2
chr3	141461486	141461637	RNF7
chr3	141620978	141621069	ATP1B3
chr3	141635218	141635250	ATP1B3
chr3	141635397	141635416	ATP1B3
chr3	141708203	141708356	TFDP2
chr3	141892016	141892039	GK5
chr3	142169375	142169444	RP11-383G6.4
chr3	142169375	142169444	ATR
chr3	142217972	142218034	ATR
chr3	142669391	142669418	PAQR9
chr3	142670636	142670648	PAQR9
chr3	142671633	142671692	PAQR9
chr3	142717307	142717336	RP11-372E1.6
chr3	142717307	142717336	U2SURP
chr3	142743326	142743365	U2SURP
chr3	145782380	145782562	AC107021.1
chr3	145782380	145782562	RP11-274H2.2
chr3	145841052	145841112	PLOD2
chr3	145881381	145881405	PLOD2
chr3	146113692	146113708	RP11-758I14.3
chr3	146113692	146113708	PLSCR2
chr3	148935653	148935772	CP
chr3	149258003	149258053	WWTR1
chr3	149258802	149258847	WWTR1
chr3	149293821	149293825	WWTR1
chr3	149468164	149468169	COMMD2
chr3	149481732	149481776	ANKUB1
chr3	149689067	149689528	AC117395.1
chr3	149689067	149689528	PFN2
chr3	150155985	150156027	TSC22D2
chr3	150275372	150275465	SERP1
chr3	150275372	150275465	EIF2A
chr3	150282978	150283013	SERP1
chr3	150282978	150283013	EIF2A
chr3	150336495	150336497	SELT

chr3	151054295	151054324	MED12L
chr3	151143435	151143528	IGSF10
chr3	151143435	151143528	MED12L
chr3	152058316	152058693	TMEM14E
chr3	152058316	152058693	MBNL1
chr3	155461005	155461391	AC104472.1
chr3	155461005	155461391	PLCH1
chr3	155521856	155521962	C3orf33
chr3	155523386	155523512	C3orf33
chr3	155991362	155991465	KCNAB1
chr3	156398955	156398978	TIPARP
chr3	156869695	156869719	CCNL1
chr3	157206929	157206949	VEPH1
chr3	157849875	157849941	RSRC1
chr3	157943446	157943522	RSRC1
chr3	158306641	158306713	MLF1
chr3	158451740	158451826	MFSD1
chr3	158451740	158451826	RP11-379F4.4
chr3	158787586	158787594	IQCJ
chr3	158787586	158787594	IQCJ-SCHIP1
chr3	159733853	159733867	LINC01100
chr3	159733853	159733867	IL12A-AS1
chr3	159736924	159736985	LINC01100
chr3	159736924	159736985	IL12A-AS1
chr3	159738433	159738531	LINC01100
chr3	159738433	159738531	IL12A-AS1
chr3	159744397	159744605	LINC01100
chr3	159744397	159744605	IL12A-AS1
chr3	160017052	160017062	RP11-432B6.3
chr3	160017052	160017062	IFT80
chr3	160061544	160061603	RP11-432B6.3
chr3	160061544	160061603	IFT80
chr3	160166514	160166583	RP11-432B6.3
chr3	160166514	160166583	TRIM59
chr3	160167472	160167482	RP11-432B6.3
chr3	160167472	160167482	TRIM59
chr3	160203147	160203164	TRIM59
chr3	165495579	165495583	BCHE
chr3	165496438	165496526	BCHE
chr3	165545797	165545857	BCHE
chr3	166966400	166966437	ZBBX
chr3	166969935	166969999	ZBBX
chr3	167084602	167084712	ZBBX
chr3	167351197	167351320	WDR49
chr3	167415737	167415766	PDCD10
chr3	169283757	169283828	MECOM
chr3	169644001	169644007	SAMD7

chr3	169687075	169687171	SEC62
chr3	169868395	169868497	PHC3
chr3	169894684	169894713	PHC3
chr3	170943323	170943424	TNIK
chr3	171844818	171844847	FNDC3B
chr3	171852151	171852172	FNDC3B
chr3	172019438	172019686	AC092964.2
chr3	172019438	172019686	FNDC3B
chr3	172034218	172034233	AC092964.1
chr3	172034218	172034233	FNDC3B
chr3	172034972	172035032	AC092964.1
chr3	172034972	172035032	FNDC3B
chr3	172039389	172039416	AC092964.1
chr3	172039389	172039416	FNDC3B
chr3	172039421	172039513	AC092964.1
chr3	172039421	172039513	FNDC3B
chr3	172361483	172361740	AC007919.2
chr3	172361483	172361740	NCEH1
chr3	178542346	178542370	KCNMB2
chr3	178542346	178542370	RP11-385J1.2
chr3	179293191	179293215	ACTL6A
chr3	179333344	179333403	NDUFB5
chr3	180331634	180331652	TTC14
chr3	180331634	180331652	CCDC39
chr3	180397396	180397587	CCDC39
chr3	180426556	180426613	CCDC39
chr3	180466034	180466069	CCDC39
chr3	182808754	182808819	MCCC1
chr3	183660221	183660303	ABCC5
chr3	183727733	183727765	ABCC5-AS1
chr3	183727733	183727765	ABCC5
chr3	183874148	183874220	DVL3
chr3	183874148	183874220	EIF2B5
chr3	184402274	184402285	EIF2B5
chr3	184554035	184554118	VPS8
chr3	184600137	184600220	VPS8
chr3	184611022	184611115	VPS8
chr3	185264666	185264760	LIPH
chr3	185300343	185300605	SENP2
chr3	185431216	185431276	C3orf65
chr3	185431216	185431276	IGF2BP2
chr3	185434229	185434599	C3orf65
chr3	185434229	185434599	IGF2BP2
chr3	185439907	185440046	C3orf65
chr3	185439907	185440046	IGF2BP2
chr3	185801958	185802069	ETV5
chr3	185826246	185826297	ETV5

chr3	185826246	185826297	DGKG
chr3	186506099	186506108	EIF4A2
chr3	186782308	186782404	ST6GAL1
chr3	187420318	187420327	RTP2
chr3	187420318	187420327	RP11-211G3.3
chr3	187433410	187433528	RP11-211G3.3
chr3	187450159	187450182	BCL6
chr3	187450159	187450182	RP11-211G3.3
chr3	187896972	187897346	AC022498.1
chr3	187896972	187897346	LPP
chr3	189561986	189562201	TP63
chr3	190333097	190333143	IL1RAP
chr3	190577595	190577608	GMNC
chr3	191986440	191986538	FGF12-AS1
chr3	191986440	191986538	FGF12
chr3	193090009	193090074	ATP13A5
chr3	194337289	194337316	TMEM44
chr3	194337499	194337620	TMEM44
chr3	194928187	194928227	XXYLT1
chr3	194990899	194990934	XXYLT1
chr3	195046173	195046184	ACAP2
chr3	195076797	195076941	ACAP2
chr3	195131713	195131738	ACAP2
chr3	195133575	195133645	ACAP2
chr3	195303933	195304054	APOD
chr3	195309488	195309537	APOD
chr3	195466718	195466801	MUC20
chr3	195467853	195467858	MUC20
chr3	195497751	195497757	MUC4
chr3	195603315	195603379	TNK2
chr3	195603482	195603607	TNK2
chr3	195622924	195623097	TNK2
chr3	195754054	195754131	TFRC
chr3	195926391	195926393	ZDHHC19
chr3	195941093	195941244	SLC51A
chr3	195941093	195941244	PCYT1A
chr3	195941341	195941382	SLC51A
chr3	195941341	195941382	PCYT1A
chr3	195950752	195950795	SLC51A
chr3	195950752	195950795	PCYT1A
chr3	195968399	195968482	SLC51A
chr3	195968399	195968482	PCYT1A
chr3	195994151	195994236	RP11-447L10.1
chr3	195994151	195994236	PCYT1A
chr3	196032563	196032644	RP11-447L10.1
chr3	196032563	196032644	TCTEX1D2
chr3	196448048	196448090	PIGX

chr3	196453526	196453570	PIGX
chr3	196658785	196658810	SENP5
chr3	196750725	196750781	MFI2
chr3	196987130	196987159	DLG1
chr3	197476404	197476521	KIAA0226
chr3	197476404	197476521	FYTTD1
chr3	197490337	197490467	FYTTD1
chr3	197680359	197680368	RPL35A
chr3	197680359	197680368	IQCG
chr4	60248	60296	ZNF595
chr4	494890	494959	PIGG
chr4	500550	500561	PIGG
chr4	942199	942225	TMEM175
chr4	942299	942340	TMEM175
chr4	980594	980639	DGKQ
chr4	980594	980639	SLC26A1
chr4	1215590	1215627	CTBP1
chr4	1217208	1217300	CTBP1
chr4	1243437	1243458	CTBP1
chr4	1243437	1243458	CTBP1-AS2
chr4	1291744	1291878	MAEA
chr4	1720828	1720955	TMEM129
chr4	1992145	1992228	NELFA
chr4	1994130	1994351	NELFA
chr4	2008828	2008926	NELFA
chr4	2501064	2501083	RNF4
chr4	2825122	2825176	SH3BP2
chr4	3265612	3265686	MSANTD1
chr4	3344231	3344305	RGS12
chr4	3511523	3511678	AL590235.1
chr4	3511523	3511678	LRPAP1
chr4	3589633	3589777	LINC00955
chr4	3590661	3591100	LINC00955
chr4	4238803	4238805	TMEM128
chr4	4463629	4463664	STX18
chr4	5141924	5141971	STK32B
chr4	5527058	5527223	C4orf6
chr4	5527922	5528058	C4orf6
chr4	5544858	5544939	EVC2
chr4	6056528	6056530	JAKMIP1
chr4	6693782	6694189	AC093323.1
chr4	8000375	8000378	ABLIM2
chr4	8007126	8007129	ABLIM2
chr4	8216899	8216957	SH3TC1
chr4	8234434	8234443	SH3TC1
chr4	8243092	8243140	SH3TC1
chr4	8612946	8612996	GPR78

chr4	8612946	8612996	CPZ
chr4	9385921	9386325	RP11-1396O13.13
chr4	9387384	9387466	RP11-1396O13.13
chr4	9388003	9388098	RP11-1396O13.13
chr4	9388875	9389001	RP11-1396O13.13
chr4	9390698	9390709	RP11-1396O13.13
chr4	10525580	10525585	CLNK
chr4	13627898	13627936	BOD1L1
chr4	15065362	15065364	CPEB2
chr4	15065362	15065364	RP11-665G4.1
chr4	15643218	15643250	FBXL5
chr4	15977813	15977818	PROM1
chr4	16057002	16057072	PROM1
chr4	16675845	16675942	LDB2
chr4	17511593	17511598	QDPR
chr4	17627279	17627294	MED28
chr4	20256637	20256698	SLIT2
chr4	20555931	20555963	SLIT2
chr4	20622027	20622038	SLIT2
chr4	21764612	21764655	KCNIP4
chr4	22348445	22348447	GPR125
chr4	25001389	25001528	LGI2
chr4	25779408	25779513	SEL1L3
chr4	26274256	26274383	RBPJ
chr4	26411664	26411670	RBPJ
chr4	26578414	26578419	TBC1D19
chr4	26578422	26578451	TBC1D19
chr4	26586804	26586806	TBC1D19
chr4	27021022	27021118	STIM2
chr4	27023116	27023234	STIM2
chr4	28364117	28364214	RP11-180C1.1
chr4	28372322	28372431	RP11-180C1.1
chr4	28383446	28383533	RP11-180C1.1
chr4	28394826	28394926	RP11-180C1.1
chr4	36210997	36211036	ARAP2
chr4	37666825	37666975	RELL1
chr4	38628340	38628882	RP11-617D20.1
chr4	38628340	38628882	AC021860.1
chr4	39065192	39065204	KLHL5
chr4	39343224	39343285	RFC1
chr4	39870323	39870355	PDS5A
chr4	40101656	40101726	N4BP2
chr4	41941738	41941804	TMEM33
chr4	41955777	41955828	TMEM33
chr4	42020406	42020431	SLC30A9
chr4	42461502	42461549	ATP8A1
chr4	44295816	44295848	KCTD8

chr4	47879086	47879208	NFXL1
chr4	48039296	48039309	NIPAL1
chr4	48682947	48683142	FRYL
chr4	48839177	48839178	OCIAD1
chr4	52714371	52714420	DCUN1D4
chr4	52753289	52753366	DCUN1D4
chr4	52771355	52771378	DCUN1D4
chr4	52775086	52775141	DCUN1D4
chr4	52898056	52898127	SGCB
chr4	53495801	53495852	USP46
chr4	53524362	53524448	USP46
chr4	54927213	54927377	AC110792.1
chr4	54927213	54927377	CHIC2
chr4	54927213	54927377	FIP1L1
chr4	55144986	55145061	PDGFRA
chr4	55144986	55145061	FIP1L1
chr4	57270146	57270284	PPAT
chr4	68376727	68376743	CENPC
chr4	69056959	69057034	FTLP10
chr4	69056959	69057034	RP11-646E20.6
chr4	69056959	69057034	TMPRSS11BNL
chr4	69057125	69057242	FTLP10
chr4	69057125	69057242	RP11-646E20.6
chr4	69057125	69057242	TMPRSS11BNL
chr4	69078080	69078195	FTLP10
chr4	69078080	69078195	RP11-646E20.6
chr4	69078080	69078195	TMPRSS11BNL
chr4	69083624	69083631	RP11-646E20.6
chr4	69083624	69083631	TMPRSS11BNL
chr4	69809040	69809096	UGT2A3
chr4	71020463	71020492	C4orf40
chr4	71065819	71065827	ODAM
chr4	71529819	71529866	ENAM
chr4	71529819	71529866	IGJ
chr4	71531577	71531603	ENAM
chr4	71531577	71531603	IGJ
chr4	74262831	74262877	ALB
chr4	75179846	75179849	EPGN
chr4	75480839	75480899	AREGB
chr4	75480839	75480899	AC142293.3
chr4	75482006	75482254	AREGB
chr4	75482006	75482254	AC142293.3
chr4	75484523	75484724	AREGB
chr4	75484523	75484724	AC142293.3
chr4	75485854	75486006	AREGB
chr4	75485854	75486006	AC142293.3
chr4	75488019	75488112	AREGB

chr4	75488019	75488112	AC142293.3
chr4	76488420	76488475	C4orf26
chr4	76655092	76655164	USO1
chr4	76688671	76688682	USO1
chr4	77066708	77066730	NUP54
chr4	77067257	77067282	NUP54
chr4	77068632	77068793	NUP54
chr4	77105888	77105933	SCARB2
chr4	77135428	77135577	FAM47E
chr4	77211055	77211213	FAM47E-STBD1
chr4	77211055	77211213	FAM47E-STBD1
chr4	77895958	77895960	11-Sep
chr4	77996625	77996696	CCNI
chr4	78100109	78100219	CCNG2
chr4	78106250	78106262	CCNG2
chr4	78740744	78740769	CNOT6L
chr4	78829479	78829529	MRPL1
chr4	78989707	78989718	FRAS1
chr4	79240514	79240590	FRAS1
chr4	79843559	79843575	PAQR3
chr4	79844055	79844137	PAQR3
chr4	79854364	79854385	PAQR3
chr4	80945807	80945909	ANTXR2
chr4	81222437	81222454	FGF5
chr4	81341368	81341386	C4orf22
chr4	81560872	81560989	C4orf22
chr4	83294272	83294303	HNRNPD
chr4	83619200	83619235	SCD5
chr4	83752090	83752128	SEC31A
chr4	83827014	83827057	THAP9
chr4	83831850	83831862	THAP9
chr4	83831850	83831862	LIN54
chr4	84405293	84405319	FAM175A
chr4	87811236	87811311	C4orf36
chr4	87970804	87970914	AFF1
chr4	89079729	89079823	ABCG2
chr4	89080357	89080391	ABCG2
chr4	89680313	89680315	FAM13A
chr4	91246747	91246857	CCSER1
chr4	91563091	91563102	CCSER1
chr4	91584996	91585055	CCSER1
chr4	91832406	91832508	CCSER1
chr4	92240235	92240284	RP11-763F8.1
chr4	92240235	92240284	CCSER1
chr4	92240796	92240877	RP11-763F8.1
chr4	92240796	92240877	CCSER1
chr4	92246265	92246402	RP11-763F8.1

chr4	92246265	92246402	CCSER1
chr4	93218651	93218780	RP11-9B6.1
chr4	93221950	93221993	RP11-9B6.1
chr4	94085885	94085896	GRID2
chr4	94223734	94223754	GRID2
chr4	95134186	95134208	SMARCAD1
chr4	95169632	95169661	SMARCAD1
chr4	95498359	95498376	PDLIM5
chr4	95498535	95498574	PDLIM5
chr4	95577969	95577971	PDLIM5
chr4	98108886	98108984	STPG2
chr4	99218069	99218073	RAP1GDS1
chr4	99317092	99317261	RAP1GDS1
chr4	99405581	99405592	TSPAN5
chr4	99405699	99405749	TSPAN5
chr4	99952659	99952684	METAP1
chr4	99952798	99952813	METAP1
chr4	100001139	100001142	ADH5
chr4	100006749	100006784	ADH5
chr4	100242048	100242071	ADH1B
chr4	100457121	100457135	C4orf17
chr4	100491857	100491933	MTTP
chr4	100827266	100827310	DNAJB14
chr4	100849714	100849774	DNAJB14
chr4	102092346	102092383	PPP3CA
chr4	102269538	102269930	AP001816.1
chr4	103675123	103675152	MANBA
chr4	103730290	103730341	UBE2D3
chr4	103803928	103803968	CISD2
chr4	103941437	103941546	SLC9B2
chr4	104012870	104012894	BDH2
chr4	106326915	106327105	PPA2
chr4	106369230	106369250	PPA2
chr4	106377244	106377246	PPA2
chr4	106377405	106377448	PPA2
chr4	106761348	106761377	INTS12
chr4	106761348	106761377	GSTCD
chr4	106892271	106892356	NPNT
chr4	106924871	106924911	RP11-710F7.3
chr4	106924871	106924911	NPNT
chr4	107158663	107158695	TBCK
chr4	107172238	107172351	TBCK
chr4	107176198	107176204	TBCK
chr4	109683541	109683543	ETNPPL
chr4	110569770	110569805	CCDC109B
chr4	110602591	110602625	CCDC109B
chr4	111014262	111014290	ELOVL6

chr4	111454242	111454249	ENPEP
chr4	113333574	113333583	ALPK1
chr4	113532002	113532175	C4orf21
chr4	113549777	113549785	C4orf21
chr4	113549993	113550007	C4orf21
chr4	113551088	113551171	C4orf21
chr4	113566707	113566810	LARP7
chr4	119607705	119607854	METTL14
chr4	119737899	119738005	SEC24D
chr4	119746688	119746773	SEC24D
chr4	120116569	120116723	RP11-455G16.1
chr4	120116866	120116996	RP11-455G16.1
chr4	120133497	120133667	RP11-455G16.1
chr4	120187455	120187466	USP53
chr4	121733929	121733969	PRDM5
chr4	122059055	122059170	TNIP3
chr4	122687200	122687499	AC079341.1
chr4	122851926	122851983	TRPC3
chr4	128597060	128597105	INTU
chr4	128606597	128606645	INTU
chr4	133356957	133357092	RP11-62N21.1
chr4	133367527	133367650	RP11-62N21.1
chr4	133368187	133368279	RP11-62N21.1
chr4	140039061	140039104	ELF2
chr4	140809739	140809756	MAML3
chr4	142144923	142144928	ZNF330
chr4	143268680	143268709	INPP4B
chr4	143344963	143344970	INPP4B
chr4	144125570	144125647	USP38
chr4	144317134	144317142	GAB1
chr4	144338586	144338605	GAB1
chr4	144794585	144794628	GYPE
chr4	144929580	144929602	RP11-673E1.4
chr4	144929580	144929602	GYPB
chr4	144937955	144938037	RP11-673E1.4
chr4	144937955	144938037	GYPB
chr4	145059314	145059396	GYPA
chr4	145059314	145059396	GYPB
chr4	146042756	146042758	ABCE1
chr4	146042756	146042758	OTUD4
chr4	147752832	147752946	TTC29
chr4	151174626	151174643	DCLK2
chr4	151762101	151762205	LRBA
chr4	152021117	152021118	RPS3A
chr4	153269826	153269881	FBXW7
chr4	154282655	154282759	MND1
chr4	155407595	155407672	DCHS2

chr4	155470816	155470924	PLRG1
chr4	155486338	155486361	FGB
chr4	156271383	156271391	MAP9
chr4	156271383	156271391	AC097467.2
chr4	157555875	157556023	RP11-171N4.2
chr4	157557720	157557758	RP11-171N4.2
chr4	157563417	157563474	RP11-171N4.2
chr4	157699353	157699382	PDGFC
chr4	157700299	157700310	PDGFC
chr4	158005816	158005834	GLRB
chr4	158281861	158281901	GRIA2
chr4	159080668	159080695	FAM198B
chr4	159531785	159531818	RXFP1
chr4	160188408	160188461	RAPGEF2
chr4	160203790	160203792	RAPGEF2
chr4	164415594	164415711	TMA16
chr4	164477063	164477125	1-Mar
chr4	166302433	166302515	CPE
chr4	167932419	167932453	SPOCK3
chr4	169621170	169621270	PALLD
chr4	169930110	169930150	CBR4
chr4	169930165	169930205	CBR4
chr4	171010412	171010451	AADAT
chr4	174311007	174311043	SCRG1
chr4	175177243	175177296	FBXO8
chr4	176892024	176892036	GPM6A
chr4	178163809	178163857	RP11-487E13.1
chr4	178167182	178167274	RP11-487E13.1
chr4	178169350	178169432	RP11-487E13.1
chr4	178882004	178882059	LINC01098
chr4	178887829	178887932	LINC01098
chr4	178896958	178897097	LINC01098
chr4	178911633	178911662	LINC01098
chr4	183370152	183370236	TENM3
chr4	184190572	184190649	WWC2
chr4	185328333	185328437	IRF2
chr4	185620729	185620756	CENPU
chr4	185734950	185735062	RP11-701P16.2
chr4	185734950	185735062	ACSL1
chr4	185736172	185736252	RP11-701P16.2
chr4	185736172	185736252	ACSL1
chr4	185742198	185742252	RP11-701P16.2
chr4	185742198	185742252	ACSL1
chr4	186323416	186323498	UFSP2
chr4	186391836	186391870	CCDC110
chr4	186393269	186393391	RP11-279O9.4
chr4	187111913	187112626	AC110771.1

chr4	187196336	187196339	F11
chr4	187513847	187513906	FAT1
chr4	190862988	190863054	FRG1
chr4	190882584	190882705	FRG1
chr4	190992204	190993661	DUX4L7
chr4	190993806	190993820	DUX4L7
chr4	190995497	190996954	DUX4L6
chr4	190997099	190997113	DUX4L6
chr4	190998790	191000247	DUX4L5
chr4	191000392	191000406	DUX4L5
chr4	191002090	191003541	DUX4L4
chr4	191003686	191003691	DUX4L4
chr4	191005384	191006841	DUX4
chr4	191006986	191007000	DUX4
chr4	191008677	191010134	DUX4L3
chr4	191010279	191010293	DUX4L3
chr4	191011977	191013434	DUX4L2
chr4	191013579	191013593	DUX4L2
chr5	220355	220452	SDHA
chr5	442578	442937	C5orf55
chr5	471908	471938	CTD-2228K2.5
chr5	471908	471938	EXOC3
chr5	472023	472080	CTD-2228K2.5
chr5	472023	472080	EXOC3
chr5	824149	824208	ZDHHC11
chr5	869383	869519	BRD9
chr5	893757	893835	TRIP13
chr5	979585	979634	RP11-661C8.3
chr5	981151	981286	RP11-661C8.3
chr5	1036895	1037018	NKD2
chr5	7654130	7654309	ADCY2
chr5	7872343	7872354	MTRR
chr5	9280914	9280919	SEMA5A
chr5	10237009	10237152	FAM173B
chr5	10505590	10505636	RP11-1C1.5
chr5	10506581	10506687	RP11-1C1.5
chr5	10509169	10509296	RP11-1C1.5
chr5	10624837	10624937	ANKRD33B
chr5	10986795	10986853	CTNND2
chr5	14490928	14490929	TRIO
chr5	14531853	14531883	TRIO
chr5	16916164	16916217	MYO10
chr5	23951673	23951912	C5orf17
chr5	23976106	23976159	C5orf17
chr5	23977890	23978031	C5orf17
chr5	23980046	23980095	C5orf17
chr5	32296538	32296576	MTMR12

chr5	32594677	32594736	SUB1
chr5	32789508	32789873	AC026703.1
chr5	32789508	32789873	NPR3
chr5	33972139	33972140	RP11-1084J3.1
chr5	33972139	33972140	SLC45A2
chr5	33994114	33994225	AMACR
chr5	33994114	33994225	RP11-1084J3.4
chr5	34716160	34716186	RAI14
chr5	35051012	35051053	AC010368.2
chr5	35051012	35051053	PRLR
chr5	35051193	35051198	AC010368.2
chr5	35051193	35051198	PRLR
chr5	35643607	35643630	SPEF2
chr5	35676038	35676094	SPEF2
chr5	35702255	35702421	CTD-2113L7.1
chr5	35702255	35702421	SPEF2
chr5	35764644	35764871	CTD-2113L7.1
chr5	35764644	35764871	SPEF2
chr5	36612848	36613010	SLC1A3
chr5	38305534	38305580	EGFLAM
chr5	39105659	39105865	AC008964.1
chr5	39105659	39105865	FYB
chr5	42646434	42646465	GHR
chr5	42647754	42647774	GHR
chr5	43120025	43120153	ZNF131
chr5	43168032	43168131	ZNF131
chr5	43384067	43384164	CCL28
chr5	43648026	43648045	NNT
chr5	51307412	51307436	CTD-2203A3.1
chr5	51374718	51374824	CTD-2203A3.1
chr5	51376577	51376696	CTD-2203A3.1
chr5	52869438	52869489	NDUFS4
chr5	54252147	54252350	RP11-45H22.3
chr5	54253522	54253626	RP11-45H22.3
chr5	54422182	54422267	CDC20B
chr5	54990014	54990032	SLC38A9
chr5	55037988	55038056	SLC38A9
chr5	55037988	55038056	DDX4
chr5	55224468	55224532	AC008914.1
chr5	55224563	55224569	AC008914.1
chr5	55753716	55753770	CTC-236F12.4
chr5	55759836	55759943	CTC-236F12.4
chr5	55760814	55760848	CTC-236F12.4
chr5	56234123	56234165	MIER3
chr5	56255213	56255243	MIER3
chr5	57838181	57838214	CTD-2117L12.1
chr5	57841919	57841986	CTD-2117L12.1

chr5	57842962	57843198	CTD-2117L12.1
chr5	57854013	57854036	CTD-2117L12.1
chr5	58386921	58386952	PDE4D
chr5	59456633	59456644	PDE4D
chr5	59726565	59726939	FKSG52
chr5	59726565	59726939	PDE4D
chr5	59984857	59984877	DEPDC1B
chr5	60064506	60064517	ELOVL7
chr5	60220334	60220336	ERCC8
chr5	60411538	60411762	AC008498.1
chr5	60411538	60411762	NDUFAF2
chr5	61808070	61808309	CKS1B
chr5	61808070	61808309	KIF2A
chr5	61808070	61808309	IPO11
chr5	61874531	61874588	LRRC70
chr5	61874531	61874588	IPO11
chr5	63989914	63989935	FAM159B
chr5	64820428	64820504	CENPK
chr5	64837198	64837263	CENPK
chr5	64909668	64909682	TRIM23
chr5	65036722	65036827	NLN
chr5	65156636	65156683	NLN
chr5	65167273	65167299	NLN
chr5	65454637	65454740	SREK1
chr5	66198477	66198555	MAST4
chr5	66226575	66226581	MAST4
chr5	66259868	66259874	MAST4
chr5	67492310	67492317	RP11-404L6.2
chr5	67494018	67494171	RP11-404L6.2
chr5	68688598	68688647	RAD17
chr5	70267276	70267398	NAIP
chr5	70307585	70307613	NAIP
chr5	70346090	70346132	GTF2H2
chr5	71412249	71412274	MAP1B
chr5	71489105	71489155	MAP1B
chr5	72350896	72350917	FCHO2
chr5	72861568	72861737	UTP15
chr5	72894203	72894224	AC008387.1
chr5	72896823	72896845	AC008387.1
chr5	74864502	74864519	POLK
chr5	75924510	75924571	IQGAP2
chr5	76775981	76776014	WDR41
chr5	78523714	78523911	AC016559.1
chr5	78523714	78523911	DMGDH
chr5	79410337	79410474	SERINC5
chr5	79412131	79412146	SERINC5
chr5	79731581	79731615	ZFYVE16

chr5	79739613	79739635	ZFYVE16
chr5	79801762	79801814	FAM151B
chr5	80512783	80512804	CKMT2-AS1
chr5	80512783	80512804	RASGRF2
chr5	81538616	81538678	ATG10
chr5	86513756	86514167	AC008394.1
chr5	86534632	86534684	AC008394.1
chr5	86662168	86662177	RASA1
chr5	87517501	87517577	TMEM161B
chr5	89816261	89816278	LYSMD3
chr5	89825299	89825332	LYSMD3
chr5	89825299	89825332	GPR98
chr5	93073588	93073646	POU5F2
chr5	93073588	93073646	FAM172A
chr5	94916596	94916641	ARSK
chr5	95010463	95010492	RFESD
chr5	95010463	95010492	SPATA9
chr5	95066581	95066600	CTD-2154I11.2
chr5	95066581	95066600	RHOBTB3
chr5	95129723	95129735	RHOBTB3
chr5	95129723	95129735	GLRX
chr5	95159541	95159725	RHOBTB3
chr5	95192612	95192685	C5orf27
chr5	95194508	95194724	C5orf27
chr5	95865525	95865584	CAST
chr5	95865525	95865584	CTD-2337A12.1
chr5	96111819	96111898	ERAP1
chr5	96111819	96111898	CAST
chr5	96149950	96150013	CTD-2260A17.2
chr5	96158763	96158820	CTD-2260A17.2
chr5	96208989	96209081	CTD-2260A17.2
chr5	96209218	96209261	CTD-2260A17.2
chr5	96217132	96217184	ERAP2
chr5	96217132	96217184	CTD-2260A17.2
chr5	96438767	96438798	CTD-2215E18.1
chr5	96438767	96438798	LIX1
chr5	96472808	96472817	CTD-2215E18.1
chr5	96472808	96472817	LIX1
chr5	96501971	96502075	RIOK2
chr5	96501971	96502075	CTD-2215E18.1
chr5	96519209	96519261	CTD-2215E18.1
chr5	100236140	100236167	ST8SIA4
chr5	101570447	101570719	AC008948.1
chr5	101570447	101570719	SLCO4C1
chr5	102456117	102456192	PPIP5K2
chr5	102471697	102471776	PPIP5K2
chr5	109219598	109219671	AC011366.3

chr5	109220138	109220174	AC011366.3
chr5	110840713	110840741	STARD4
chr5	111755295	111756116	EPB41L4A-AS2
chr5	112145823	112145852	APC
chr5	112159004	112159057	APC
chr5	112257417	112257507	REEP5
chr5	113822395	113822472	KCNN2
chr5	115176245	115176309	ATG12
chr5	115176609	115176631	ATG12
chr5	122189369	122189404	SNX24
chr5	122301138	122301155	SNX24
chr5	122738946	122739038	CEP120
chr5	122739388	122739434	CEP120
chr5	125929858	125929959	ALDH7A1
chr5	126392145	126392198	C5orf63
chr5	127777992	127778065	FBN2
chr5	130496517	130496537	HINT1
chr5	130733902	130734027	CDC42SE2
chr5	130775917	130775921	RAPGEF6
chr5	130775917	130775921	CTC-432M15.3
chr5	130867794	130867847	RAPGEF6
chr5	130867794	130867847	CTC-432M15.3
chr5	131755585	131755632	C5orf56
chr5	131755585	131755632	AC116366.5
chr5	131785342	131785381	C5orf56
chr5	131792704	131792858	C5orf56
chr5	131796254	131796546	C5orf56
chr5	131805732	131805757	AC116366.6
chr5	131805732	131805757	C5orf56
chr5	131811359	131811624	C5orf56
chr5	131928456	131928642	RAD50
chr5	131978697	131978781	RAD50
chr5	131978697	131978781	AC004041.2
chr5	132072467	132072469	KIF3A
chr5	132918956	132919087	FSTL4
chr5	133480951	133480985	TCF7
chr5	133487347	133487414	TCF7
chr5	133487347	133487414	SKP1
chr5	133506701	133506729	SKP1
chr5	133506701	133506729	CTD-2410N18.5
chr5	133559948	133560010	CTD-2410N18.5
chr5	133559948	133560010	PPP2CA
chr5	133559948	133560010	CDKL3
chr5	133559948	133560010	CTD-2410N18.4
chr5	133622064	133622250	CDKL3
chr5	133622064	133622250	CTD-2410N18.4
chr5	133724668	133724743	UBE2B

chr5	133944737	133944823	SAR1B
chr5	134210827	134210878	TXNDC15
chr5	134211032	134211085	TXNDC15
chr5	134368535	134368825	PITX1
chr5	134690739	134690810	H2AFY
chr5	134690739	134690810	C5orf66
chr5	135170835	135170839	SLC25A48
chr5	135522849	135522866	SMAD5
chr5	136988763	136988765	KLHL3
chr5	137493581	137493583	BRD8
chr5	137499282	137499332	BRD8
chr5	137508409	137508540	BRD8
chr5	137775371	137775515	REEP2
chr5	137896470	137896620	HSPA9
chr5	138117979	138118041	CTNNA1
chr5	138216731	138216793	CTNNA1
chr5	138611828	138611839	MATR3
chr5	138611828	138611839	SIL1
chr5	138725098	138725109	MZB1
chr5	138754440	138754522	DNAJC18
chr5	138754824	138754861	DNAJC18
chr5	140027732	140027747	IK
chr5	140028468	140028565	IK
chr5	140063250	140063252	HARS
chr5	140242397	140242975	AC005609.1
chr5	140242397	140242975	PCDHA14
chr5	140242397	140242975	PCDHA1
chr5	140242397	140242975	PCDHA2
chr5	140242397	140242975	PCDHA3
chr5	140242397	140242975	PCDHA4
chr5	140242397	140242975	PCDHA5
chr5	140242397	140242975	PCDHA6
chr5	140242397	140242975	PCDHA7
chr5	140242397	140242975	PCDHA8
chr5	140242397	140242975	PCDHA9
chr5	140242397	140242975	PCDHA10
chr5	140535577	140537340	PCDHB17
chr5	140940458	140940469	CTD-2024I7.13
chr5	140940458	140940469	DIAPH1
chr5	141247114	141247179	PCDH1
chr5	142152346	142152415	ARHGAP26
chr5	142595752	142595846	ARHGAP26
chr5	145889630	145889633	TCERG1
chr5	145971699	145971756	CTB-99A3.1
chr5	145971699	145971756	PPP2R2B
chr5	146420298	146420368	PPP2R2B
chr5	146434188	146434222	PPP2R2B

chr5	147468582	147468656	SPINK5
chr5	147468780	147468835	SPINK5
chr5	147698550	147698623	AC091948.1
chr5	147698550	147698623	RP11-373N22.3
chr5	147701843	147701891	AC091948.1
chr5	147701843	147701891	RP11-373N22.3
chr5	147701843	147701891	SPINK9
chr5	147870370	147870409	HTR4
chr5	147900485	147900526	HTR4
chr5	148383301	148383405	SH3TC2
chr5	148414059	148414083	SH3TC2
chr5	148425516	148425524	SH3TC2
chr5	148880694	148880785	CTB-89H12.4
chr5	148880694	148880785	CSNK1A1
chr5	148909276	148909282	CSNK1A1
chr5	149372773	149372775	SLC26A2
chr5	149372773	149372775	TIGD6
chr5	149437824	149437834	CSF1R
chr5	149824188	149824204	RPS14
chr5	150077288	150077300	RBM22
chr5	150158132	150158534	SMIM3
chr5	150158132	150158534	AC010441.1
chr5	150257542	150257598	IRGM
chr5	150409631	150409684	TNIP1
chr5	150480450	150480523	ANXA6
chr5	150595152	150595247	CCDC69
chr5	150595152	150595247	GM2A
chr5	151167326	151167355	G3BP1
chr5	151171606	151171716	G3BP1
chr5	153735713	153735753	GALNT10
chr5	153735713	153735753	SAP30L-AS1
chr5	154166037	154166047	LARP1
chr5	154171506	154171541	LARP1
chr5	154184334	154184397	LARP1
chr5	154184421	154184442	LARP1
chr5	154247252	154247294	CNOT8
chr5	155820763	155820819	SGCD
chr5	156640578	156640603	ITK
chr5	156640578	156640603	CTB-4E7.1
chr5	156715621	156715629	CYFIP2
chr5	156738045	156738058	CYFIP2
chr5	156822574	156822687	CTB-109A12.1
chr5	156822574	156822687	CYFIP2
chr5	156822574	156822687	ADAM19
chr5	156825169	156825222	CTB-109A12.1
chr5	156825169	156825222	ADAM19
chr5	156904690	156904743	ADAM19

chr5	156999278	156999283	ADAM19
chr5	157165630	157165647	THG1L
chr5	159492284	159492443	TTC1
chr5	159492284	159492443	PWWP2A
chr5	159516592	159516617	PWWP2A
chr5	159655480	159655503	FABP6
chr5	159696466	159696551	CCNJL
chr5	159742389	159742512	CCNJL
chr5	159766452	159766528	CCNJL
chr5	161114486	161114567	RP11-348M17.2
chr5	161114486	161114567	GABRA6
chr5	161276039	161276041	GABRA1
chr5	161510507	161510555	GABRG2
chr5	162873776	162873821	NUDCD2
chr5	162873776	162873821	AC112205.1
chr5	167566511	167566522	CTB-178M22.1
chr5	167566511	167566522	TENM2
chr5	167576433	167576447	CTB-178M22.1
chr5	167576433	167576447	TENM2
chr5	169424978	169425001	DOCK2
chr5	169910064	169910104	KCNIP1
chr5	169988118	169988119	KCNIP1
chr5	170455876	170455896	RANBP17
chr5	171201346	171201737	CTB-78H18.1
chr5	171404640	171404672	FBXW11
chr5	171423957	171424055	FBXW11
chr5	171472430	171472484	STK10
chr5	171760729	171760773	SH3PXD2B
chr5	172036245	172036436	AC027309.1
chr5	172333063	172333183	ERGIC1
chr5	172587827	172587873	BNIP1
chr5	173493207	173493278	NSG2
chr5	175345102	175345175	RP11-91H12.4
chr5	175345102	175345175	THOC3
chr5	175353638	175353744	RP11-91H12.4
chr5	175353638	175353744	THOC3
chr5	175438101	175438103	THOC3
chr5	175477606	175477735	RP11-826N14.2
chr5	175487235	175487293	RP11-826N14.2
chr5	175731320	175731421	SIMC1
chr5	175828742	175828753	ARL10
chr5	175828742	175828753	CLTB
chr5	175875168	175875170	FAF2
chr5	175993947	175994011	CDHR2
chr5	176291817	176291848	UNC5A
chr5	176292551	176292722	UNC5A
chr5	176656766	176656875	NSD1

chr5	176941181	176941289	DDX41
chr5	176950233	176950266	FAM193B
chr5	176980681	176980803	FAM193B
chr5	177032046	177032049	B4GALT7
chr5	177210798	177210856	RP11-1026M7.2
chr5	177220343	177220472	RP11-1026M7.2
chr5	177459744	177459887	FAM153C
chr5	177650428	177650452	PHYKPL
chr5	177682788	177682890	COL23A1
chr5	177989027	177989239	COL23A1
chr5	178047566	178047674	CLK4
chr5	178049459	178049461	CLK4
chr5	178153188	178153228	ZNF354A
chr5	178982651	178982791	RUFY1
chr5	179078298	179078353	AC136604.1
chr5	179078574	179079445	AC136604.1
chr5	179125503	179125604	CANX
chr5	179126027	179126103	CANX
chr5	179278243	179278337	C5orf45
chr5	179278346	179278424	C5orf45
chr5	179287310	179287780	CTC-241N9.1
chr5	179287310	179287780	C5orf45
chr5	179289542	179289598	TBC1D9B
chr5	179779372	179779879	GFPT2
chr5	180669554	180669620	GNB2L1
chr5	180670286	180670405	SNORD95
chr5	180670286	180670405	GNB2L1
chr5	180674124	180674376	GNB2L1
chr5	180674124	180674376	CTC-338M12.4
chr5	180682781	180683218	CTC-338M12.4
chr5	180682781	180683218	TRIM52
chr5	180682781	180683218	AC008443.1
chr6	406766	406786	IRF4
chr6	1101508	1101531	AL033381.1
chr6	1102041	1102415	AL033381.1
chr6	2623673	2624056	C6orf195
chr6	3244327	3244360	PSMG4
chr6	3323513	3323552	SLC22A23
chr6	3983402	3983710	C6ORF50
chr6	4115641	4115704	C6orf201
chr6	4135349	4135539	ECI2
chr6	4773382	4773412	CDYL
chr6	5001328	5001364	RPP40
chr6	5113550	5113651	LYRM4
chr6	6206790	6206835	F13A1
chr6	6320820	6320892	F13A1
chr6	6321033	6321103	F13A1

chr6	7389516	7389528	CAGE1
chr6	8027613	8027632	BLOC1S5
chr6	8027613	8027632	EEF1E1-BLOC1S5
chr6	8027613	8027632	BLOC1S5-TXNDC5
chr6	8078908	8078973	EEF1E1-BLOC1S5
chr6	8078908	8078973	EEF1E1
chr6	8095611	8095728	EEF1E1-BLOC1S5
chr6	8095611	8095728	EEF1E1
chr6	8101998	8102120	EEF1E1-BLOC1S5
chr6	8101998	8102120	EEF1E1
chr6	8419047	8419118	SLC35B3
chr6	9707995	9708205	OFCC1
chr6	9769164	9769288	OFCC1
chr6	9776040	9776122	OFCC1
chr6	9809810	9809947	OFCC1
chr6	9819637	9819657	OFCC1
chr6	9840547	9840747	OFCC1
chr6	9842749	9842869	OFCC1
chr6	9845750	9845826	OFCC1
chr6	9977647	9977801	OFCC1
chr6	10393757	10393794	TFAP2A
chr6	10633993	10634929	GCNT6
chr6	10647263	10647501	GCNT6
chr6	10798071	10798106	TMEM14B
chr6	10798071	10798106	MAK
chr6	10798071	10798106	SYCP2L
chr6	10798071	10798106	RP11-637O19.3
chr6	11249372	11249431	RP3-510L9.1
chr6	11249372	11249431	NEDD9
chr6	11305299	11305392	NEDD9
chr6	13288633	13288645	RP1-257A7.4
chr6	13288633	13288645	TBC1D7
chr6	13288633	13288645	PHACTR1
chr6	13323943	13324010	TBC1D7
chr6	13585219	13585229	SIRT5
chr6	13804309	13804547	MCUR1
chr6	15401182	15401248	JARID2
chr6	15546273	15546298	DTNBP1
chr6	17111055	17111111	STMND1
chr6	17282568	17282629	RBM24
chr6	17493610	17493624	CAP2
chr6	17536469	17536495	CAP2
chr6	17936608	17936616	KIF13A
chr6	18244739	18244834	DEK
chr6	18368986	18369049	RNF144B
chr6	24353533	24353547	DCDC2
chr6	25479336	25479475	LRRC16A

chr6	26463025	26463050	BTN2A1
chr6	27094201	27094241	HIST1H2BJ
chr6	27253865	27253927	POM121L2
chr6	28321639	28321671	ZKSCAN3
chr6	28321639	28321671	ZSCAN31
chr6	28875269	28875340	TRIM27
chr6	28911681	28911803	C6orf100
chr6	28911999	28912064	C6orf100
chr6	29632407	29632486	MOG
chr6	29704192	29704200	HLA-F
chr6	29704192	29704200	HLA-F-AS1
chr6	30137269	30137275	TRIM15
chr6	30309166	30309222	TRIM39
chr6	30309166	30309222	TRIM39-RPP21
chr6	30853553	30853588	DDR1
chr6	30867825	30867859	DDR1
chr6	31134775	31134785	TCF19
chr6	31134775	31134785	POU5F1
chr6	31165537	31165659	XXbac-BPG299F13.17
chr6	31165537	31165659	HCG27
chr6	31165915	31165958	HCG27
chr6	31170149	31170594	HCG27
chr6	31466428	31466483	MICB
chr6	31502263	31502288	ATP6V1G2-DDX39B
chr6	31502263	31502288	DDX39B
chr6	31508930	31508972	SNORD84
chr6	31508930	31508972	ATP6V1G2-DDX39B
chr6	31508930	31508972	DDX39B
chr6	31509411	31509477	ATP6V1G2-DDX39B
chr6	31509411	31509477	DDX39B
chr6	31524474	31524517	NFKBIL1
chr6	31650827	31651118	LY6G5C
chr6	31651765	31651817	LY6G5C
chr6	31900252	31900270	C2
chr6	31900252	31900270	CFB
chr6	31900252	31900270	CFB
chr6	31973945	31973975	CYP21A1P
chr6	31973945	31973975	AL645922.1
chr6	31974844	31974881	CYP21A1P
chr6	31974844	31974881	AL645922.1
chr6	32606689	32606768	HLA-DQA1
chr6	32920061	32920238	XXbac-BPG181M17.5
chr6	32920061	32920238	HLA-DMA
chr6	32942798	32942889	BRD2
chr6	33054316	33054324	HLA-DPB1
chr6	33368169	33368291	RPL12P1
chr6	33368169	33368291	KIFC1

chr6	33553402	33553498	GGNBP1
chr6	33554447	33554565	GGNBP1
chr6	33554447	33554565	LINC00336
chr6	33556690	33556803	GGNBP1
chr6	33556690	33556803	LINC00336
chr6	35057961	35058013	ANKS1A
chr6	35105550	35105629	TCP11
chr6	35283882	35283923	DEF6
chr6	35761265	35761312	CLPSL1
chr6	35806483	35806575	SRPK1
chr6	35872656	35872749	SRPK1
chr6	35992197	35992416	SLC26A8
chr6	35992514	35992633	SLC26A8
chr6	36567598	36567631	SRSF3
chr6	36944274	36944374	MTCH1
chr6	37012636	37012965	COX6A1P2
chr6	37326959	37326997	RNF8
chr6	37329114	37329143	RN7SL273P
chr6	37329114	37329143	RNF8
chr6	37971467	37971559	ZFAND3
chr6	38110035	38110069	ZFAND3
chr6	38472332	38472338	BTBD9
chr6	39551208	39551255	KIF6
chr6	39585504	39585529	KIF6
chr6	39872034	39872038	MOCS1
chr6	39872034	39872038	DAAM2
chr6	41020016	41020102	OARD1
chr6	41200101	41200113	TREML4
chr6	41538016	41538132	FOXP4
chr6	41721636	41721706	PGC
chr6	41748123	41748171	PRICKLE4
chr6	41748123	41748171	FRS3
chr6	41885533	41885630	MED20
chr6	43482931	43483044	POLR1C
chr6	43482931	43483044	YIPF3
chr6	43587987	43588070	GTPBP2
chr6	44136679	44136768	CAPN11
chr6	44400472	44400720	CDC5L
chr6	44400472	44400720	AL133262.1
chr6	46626226	46626265	SLC25A27
chr6	46668736	46668799	TDRD6
chr6	52130033	52130183	MCM3
chr6	52959490	52959639	FBXO9
chr6	52962959	52962985	FBXO9
chr6	53795019	53795075	RP11-411K7.1
chr6	53795019	53795075	MLIP
chr6	53847556	53847648	RP11-411K7.1

chr6	53847556	53847648	MLIP
chr6	55428659	55428712	HMGCLL1
chr6	55431415	55431426	HMGCLL1
chr6	55942891	55942902	COL21A1
chr6	55942951	55942986	COL21A1
chr6	56466803	56466804	DST
chr6	63989288	63989332	LGSN
chr6	64388729	64388733	PHF3
chr6	64454879	64454913	PHF3
chr6	64454879	64454913	EYS
chr6	66201463	66201482	EYS
chr6	70769654	70769689	COL19A1
chr6	70840945	70840950	COL19A1
chr6	71469531	71469606	SMAP1
chr6	72885064	72885075	RIMS1
chr6	73752791	73752936	KCNQ5
chr6	73835131	73835155	KCNQ5
chr6	73835243	73835247	KCNQ5
chr6	74196321	74196381	MTO1
chr6	75951538	75951654	COX7A2
chr6	76332467	76332545	SENP6
chr6	76644640	76644766	IMPG1
chr6	82204789	82204830	FAM46A
chr6	82262307	82262537	AL359693.1
chr6	82262307	82262537	FAM46A
chr6	82884695	82884719	IBTK
chr6	83742016	83742029	UBE3D
chr6	83759488	83759491	UBE3D
chr6	83782692	83782711	AL139333.1
chr6	83782692	83782711	DOPEY1
chr6	83782821	83782854	AL139333.1
chr6	83782821	83782854	DOPEY1
chr6	83879399	83879560	DOPEY1
chr6	83879399	83879560	PGM3
chr6	84321157	84321162	SNAP91
chr6	84400109	84400155	SNAP91
chr6	84928886	84928932	KIAA1009
chr6	86200753	86200844	NT5E
chr6	86298581	86298622	RP11-321N4.5
chr6	86298581	86298622	SNX14
chr6	87959030	87959143	ZNF292
chr6	88081311	88081352	RP1-102H19.8
chr6	88107933	88108095	C6orf164
chr6	88107933	88108095	RP1-102H19.8
chr6	88109156	88109223	C6orf164
chr6	88109156	88109223	RP1-102H19.8
chr6	88274262	88274380	RARS2

chr6	89675362	89675796	AL079342.1
chr6	89914398	89914413	GABRR1
chr6	97694024	97694032	MMS22L
chr6	97718154	97718159	MMS22L
chr6	99916024	99916046	USP45
chr6	99949815	99949885	USP45
chr6	100014774	100014914	CCNC
chr6	105272780	105272782	HACE1
chr6	105306371	105306407	HACE1
chr6	106546549	106546596	PRDM1
chr6	106989093	106989170	AIM1
chr6	108061353	108061360	SCML4
chr6	108277192	108277310	SEC63
chr6	108543441	108543524	SNX3
chr6	109201131	109201161	ARMC2
chr6	109524918	109525070	C6orf183
chr6	109527555	109527656	C6orf183
chr6	109538428	109538589	C6orf183
chr6	109726148	109726251	PPIL6
chr6	110106874	110106917	FIG4
chr6	111793339	111793343	REV3L
chr6	112381216	112381278	WISP3
chr6	112501104	112501151	LAMA4
chr6	116323848	116323887	FRK
chr6	116466601	116466658	COL10A1
chr6	116466601	116466658	NT5DC1
chr6	116575457	116575458	RP3-486I3.7
chr6	116575457	116575458	DSE
chr6	116579609	116580130	RP3-486I3.4
chr6	116579609	116580130	DSE
chr6	119653101	119653169	MAN1A1
chr6	121451021	121451070	TBC1D32
chr6	123819645	123819790	TRDN
chr6	124603384	124603392	NKAIN2
chr6	125292647	125292744	RNF217
chr6	125367753	125367806	RNF217
chr6	125369358	125369405	RNF217
chr6	126221142	126221167	NCOA7
chr6	126327988	126328001	TRMT11
chr6	127634724	127634736	ECHDC1
chr6	127636280	127636283	ECHDC1
chr6	127647641	127647719	ECHDC1
chr6	127650996	127651027	ECHDC1
chr6	127772427	127772462	KIAA0408
chr6	127772427	127772462	SOGA3
chr6	127772427	127772462	SOGA3
chr6	128730458	128730477	PTPRK

chr6	128812877	128812994	PTPRK
chr6	131193511	131193678	EPB41L2
chr6	131485538	131485552	AKAP7
chr6	131959587	131959674	ENPP3
chr6	133074613	133074630	RP1-55C23.7
chr6	133074613	133074630	VNN2
chr6	134536193	134536231	SGK1
chr6	135261319	135261359	ALDH8A1
chr6	135424066	135424153	HBS1L
chr6	135516098	135516202	MYB
chr6	135520664	135520667	MYB
chr6	135623947	135623955	RP3-388E23.2
chr6	135623947	135623955	AHI1
chr6	136568627	136568698	MTFR2
chr6	137539587	137539589	IFNGR1
chr6	138733220	138733293	HEBP2
chr6	139013613	139013708	RP11-390P2.4
chr6	139013613	139013708	NHSL1
chr6	139255450	139255463	REPS1
chr6	142726480	142726528	GPR126
chr6	143381782	143381790	AIG1
chr6	143763306	143763308	AL031320.1
chr6	143763306	143763308	ADAT2
chr6	143764263	143764295	AL031320.1
chr6	143764263	143764295	ADAT2
chr6	145120590	145120609	UTRN
chr6	145822943	145822983	EPM2A
chr6	146272927	146273059	SHPRH
chr6	146921469	146921499	ADGB
chr6	147091715	147091745	ADGB
chr6	148058453	148058584	RP11-307P5.1
chr6	148058453	148058584	SAMD5
chr6	149693519	149693540	TAB2
chr6	149701070	149701077	TAB2
chr6	149903196	149903234	GINM1
chr6	150038985	150039170	LATS1
chr6	150086827	150086888	PCMT1
chr6	150093150	150093178	PCMT1
chr6	150093184	150093224	PCMT1
chr6	150093691	150093749	PCMT1
chr6	151233804	151233841	MTHFD1L
chr6	151343165	151343176	MTHFD1L
chr6	151362912	151362976	MTHFD1L
chr6	152801896	152801916	SYNE1
chr6	153667705	153668592	AL590867.1
chr6	154332144	154332153	OPRM1
chr6	154415345	154415389	OPRM1

chr6	154735857	154735899	CNKSR3
chr6	155538214	155538224	TIAM2
chr6	157507489	157507739	ARID1B
chr6	158438767	158438827	SYNJ2
chr6	158451439	158451441	SYNJ2
chr6	159047471	159047478	AL591025.1
chr6	159047471	159047478	TMEM181
chr6	159047695	159047738	AL591025.1
chr6	159047695	159047738	TMEM181
chr6	160148298	160148389	SOD2
chr6	160148298	160148389	WTAP
chr6	161565161	161565298	AGPAT4
chr6	161586692	161586745	AGPAT4
chr6	163418438	163418494	PACRG
chr6	163587068	163587294	AL078585.1
chr6	163587068	163587294	PACRG
chr6	163611940	163612014	AL078585.1
chr6	163611940	163612014	PACRG
chr6	163612634	163612826	AL078585.1
chr6	163612634	163612826	PACRG
chr6	163878413	163878418	QKI
chr6	163990470	163990516	QKI
chr6	166307713	166308153	SDIM1
chr6	166307713	166308153	PDE10A
chr6	166889272	166889327	RPS6KA2
chr6	166945703	166946107	Z98049.1
chr6	166945703	166946107	RPS6KA2
chr6	167171015	167171065	RPS6KA2
chr6	167741492	167741597	TTLL2
chr6	168188244	168188287	C6orf123
chr6	168191627	168191756	C6orf123
chr6	168196616	168197182	C6orf123
chr6	168196616	168197182	AL009178.1
chr6	168436422	168436431	KIF25
chr6	168467768	168467809	FRMD1
chr6	169056076	169056095	SMOC2
chr7	228396	228860	AC145676.2
chr7	228396	228860	FAM20C
chr7	829090	829190	HEATR2
chr7	855528	855566	SUN1
chr7	855873	855930	SUN1
chr7	856254	856310	SUN1
chr7	889157	889240	SUN1
chr7	923841	924031	RP11-449P15.2
chr7	923841	924031	SUN1
chr7	923841	924031	GET4
chr7	940573	940668	ADAP1

chr7	940573	940668	COX19
chr7	966692	966852	ADAP1
chr7	966692	966852	COX19
chr7	967110	967150	ADAP1
chr7	967110	967150	COX19
chr7	1543959	1544243	INTS1
chr7	1545432	1545489	INTS1
chr7	1732947	1733531	AC074389.6
chr7	1732947	1733531	ELFN1
chr7	1878377	1878504	AC110781.3
chr7	1878377	1878504	MAD1L1
chr7	1886884	1887418	AC110781.3
chr7	1886884	1887418	MAD1L1
chr7	2119333	2119406	MAD1L1
chr7	2278469	2278572	FTSJ2
chr7	2612348	2612455	IQCE
chr7	2621661	2621711	IQCE
chr7	2636592	2636771	IQCE
chr7	2685377	2685520	TTYH3
chr7	3179868	3179930	AC091801.1
chr7	3180554	3180633	AC091801.1
chr7	3180724	3180834	AC091801.1
chr7	3186876	3186905	AC091801.1
chr7	3188615	3188702	AC091801.1
chr7	3197771	3197888	AC091801.1
chr7	3205664	3205728	AC091801.1
chr7	3997672	3997688	SDK1
chr7	4829845	4829881	AP5Z1
chr7	5487468	5487474	FBXL18
chr7	5800060	5800142	RNF216
chr7	6052310	6052321	AIMP2
chr7	6097779	6097873	EIF2AK1
chr7	6151428	6151483	USP42
chr7	6156426	6156508	USP42
chr7	6212362	6212555	CYTH3
chr7	6522195	6522220	DAGLB
chr7	6522195	6522220	KDELR2
chr7	6713376	6713397	AC073343.1
chr7	6713376	6713397	AC073343.13
chr7	6713424	6713456	AC073343.1
chr7	6713424	6713456	AC073343.13
chr7	6713535	6713674	AC073343.1
chr7	6713535	6713674	AC073343.13
chr7	6713781	6713798	AC073343.1
chr7	6713781	6713798	AC073343.13
chr7	6713803	6713855	AC073343.1
chr7	6713803	6713855	AC073343.13

chr7	6713920	6714075	AC073343.1
chr7	6713920	6714075	AC073343.13
chr7	6714482	6714584	AC073343.1
chr7	6714482	6714584	AC073343.13
chr7	6714599	6714653	AC073343.1
chr7	6714599	6714653	AC073343.13
chr7	6714739	6714811	AC073343.1
chr7	6714739	6714811	AC073343.13
chr7	6714865	6714953	AC073343.1
chr7	6714865	6714953	AC073343.13
chr7	6714990	6714994	AC073343.1
chr7	6714990	6714994	AC073343.13
chr7	6715247	6715369	AC073343.1
chr7	6715247	6715369	AC073343.13
chr7	6715404	6715618	AC073343.1
chr7	6715404	6715618	AC073343.13
chr7	6715621	6715991	AC073343.1
chr7	6715621	6715991	AC073343.13
chr7	7551353	7551475	COL28A1
chr7	8132279	8132324	GLCCI1
chr7	8263188	8263189	ICA1
chr7	12255230	12255327	TMEM106B
chr7	12611170	12611250	SCIN
chr7	12617167	12617276	SCIN
chr7	12636013	12636051	SCIN
chr7	13947418	13947481	ETV1
chr7	14789758	14789778	DGKB
chr7	15398035	15398070	AGMO
chr7	16828866	16829246	AC073333.1
chr7	16872880	16872932	AGR2
chr7	16921568	16921611	AGR3
chr7	17928321	17928345	SNX13
chr7	20802879	20802893	ABCB5
chr7	21890933	21890938	DNAH11
chr7	22231181	22231260	RAPGEF5
chr7	23053544	23053749	FAM126A
chr7	23210760	23210807	AC005082.1
chr7	23210760	23210807	KLHL7
chr7	23211775	23211796	AC005082.1
chr7	23211775	23211796	KLHL7
chr7	23561973	23562051	TRA2A
chr7	23725908	23726013	FAM221A
chr7	23727847	23727904	FAM221A
chr7	24706484	24706601	MPP6
chr7	24746931	24747044	DFNA5
chr7	24755110	24755192	DFNA5
chr7	26249560	26249577	CBX3

chr7	26576372	26576639	KIAA0087
chr7	26578025	26578173	KIAA0087
chr7	26933458	26933479	SKAP2
chr7	27284086	27284137	EVX1-AS
chr7	27284086	27284137	EVX1
chr7	27687292	27687375	HIBADH
chr7	27886122	27886166	JAZF1
chr7	27953060	27953072	JAZF1
chr7	27954373	27954427	JAZF1
chr7	28060172	28060331	JAZF1
chr7	28111237	28111252	JAZF1
chr7	29484763	29484773	CHN2
chr7	30059913	30059920	AC007285.6
chr7	30059913	30059920	FKBP14
chr7	30177200	30177310	MTURN
chr7	30666673	30666686	GARS
chr7	33069222	33069335	NT5C3A
chr7	33069222	33069335	AVL9
chr7	33078454	33078516	NT5C3A
chr7	33078454	33078516	AVL9
chr7	33385839	33385892	BBS9
chr7	33961390	33961443	BMPER
chr7	33979564	33979633	BMPER
chr7	34685709	34685726	AC005493.1
chr7	34685709	34685726	NPSR1-AS1
chr7	34687590	34687624	AC005493.1
chr7	34687590	34687624	NPSR1-AS1
chr7	34699742	34699772	AC005493.1
chr7	34699742	34699772	NPSR1
chr7	34699742	34699772	NPSR1-AS1
chr7	35851671	35851678	7-Sep
chr7	35854755	35854756	7-Sep
chr7	35871700	35871723	7-Sep
chr7	35928433	35928455	7-Sep
chr7	36118843	36118925	PP13004
chr7	36124082	36124370	PP13004
chr7	37072983	37073017	ELMO1
chr7	37243714	37243905	ELMO1
chr7	37257317	37257361	ELMO1
chr7	37966282	37966324	SFRP4
chr7	37966282	37966324	EPDR1
chr7	38460599	38460616	AMPH
chr7	38725198	38725605	FAM183B
chr7	38780908	38780951	VPS41
chr7	38836067	38836126	VPS41
chr7	38870816	38870839	VPS41
chr7	39074071	39074091	POU6F2

chr7	39160433	39160455	POU6F2
chr7	39737007	39737021	RALA
chr7	40256996	40257120	SUGCT
chr7	42970736	42970754	PSMA2
chr7	42970736	42970754	PSMA2
chr7	43562648	43562745	HECW1
chr7	43686170	43686180	COA1
chr7	44119964	44120070	POLM
chr7	44145838	44145840	AEBP1
chr7	44149256	44149287	AEBP1
chr7	44185673	44185743	GCK
chr7	44284488	44284614	CAMK2B
chr7	44363410	44363522	CAMK2B
chr7	44840022	44840024	PPIA
chr7	44861593	44861657	PPIA
chr7	44862739	44862781	PPIA
chr7	45961226	45961298	IGFBP3
chr7	46728998	46729144	AC011294.3
chr7	46732409	46732567	AC011294.3
chr7	46736601	46736612	AC011294.3
chr7	48011099	48011113	HUS1
chr7	48015938	48016018	HUS1
chr7	48030607	48030642	SUN3
chr7	48143379	48143458	UPP1
chr7	48643310	48643346	ABCA13
chr7	48887501	48887911	AC004899.3
chr7	48887501	48887911	AC004899.1
chr7	50156849	50156858	C7orf72
chr7	50156849	50156858	ZPBP
chr7	55447340	55447342	LANCL2
chr7	55985735	55985803	MRPS17
chr7	55985735	55985803	ZNF713
chr7	56047453	56047579	GBAS
chr7	56059521	56059524	GBAS
chr7	62810010	62810456	AC006455.1
chr7	62810010	62810456	RP5-905H7.10
chr7	62810010	62810456	RP5-905H7.9
chr7	62858414	62858860	AC073188.1
chr7	62858414	62858860	SLC25A1P2
chr7	62858414	62858860	RP11-340I6.3
chr7	64377408	64377496	ZNF273
chr7	64383764	64383783	ZNF273
chr7	65112081	65112473	AC104057.1
chr7	65112475	65112954	AC104057.1
chr7	65112475	65112954	INTS4L2
chr7	65113248	65113280	AC104057.1
chr7	65113248	65113280	INTS4L2

chr7	65591623	65591630	AC068533.7
chr7	65591623	65591630	CRCP
chr7	65591623	65591630	RP5-1132H15.1
chr7	65595114	65595197	AC068533.7
chr7	65595114	65595197	CRCP
chr7	65595114	65595197	RP5-1132H15.1
chr7	65939543	65939677	AC008267.1
chr7	65939543	65939677	GS1-124K5.2
chr7	70194125	70194207	AUTS2
chr7	70250395	70250458	AUTS2
chr7	70765317	70765488	WBSCR17
chr7	72459478	72459492	AC005488.1
chr7	72459478	72459492	STAG3L3
chr7	72464847	72464894	AC005488.1
chr7	72464847	72464894	STAG3L3
chr7	72464982	72464996	AC005488.1
chr7	72464982	72464996	STAG3L3
chr7	72750761	72750787	FKBP6
chr7	72957169	72957237	BCL7B
chr7	72986278	72986365	TBL2
chr7	72989100	72989134	TBL2
chr7	73275799	73275909	WBSCR28
chr7	73457875	73457882	ELN
chr7	73648150	73648224	RFC2
chr7	73668046	73668048	RFC2
chr7	73795912	73795960	CLIP2
chr7	74192653	74192745	NCF1
chr7	74192653	74192745	STAG3L2
chr7	74194503	74194572	NCF1
chr7	74194503	74194572	STAG3L2
chr7	74489829	74489933	WBSCR16
chr7	74807605	74807670	GATSL2
chr7	74807770	74807864	GATSL2
chr7	74810284	74810366	GATSL2
chr7	74812091	74812201	GATSL2
chr7	74813145	74813268	GATSL2
chr7	74814118	74814250	GATSL2
chr7	74814449	74814642	GATSL2
chr7	74824183	74824253	GATSL2
chr7	74867229	74867341	GATSL2
chr7	74999908	74999922	AC006014.1
chr7	74999908	74999922	STAG3L1
chr7	75000010	75000057	AC006014.1
chr7	75000010	75000057	STAG3L1
chr7	75005411	75005425	AC006014.1
chr7	75005411	75005425	STAG3L1
chr7	75510683	75510744	RHBDD2

chr7	75597400	75597446	POR
chr7	75597569	75597595	POR
chr7	75914572	75914574	SRRM3
chr7	77193253	77193291	PTPN12
chr7	77234196	77234199	PTPN12
chr7	78212738	78212771	MAGI2
chr7	80422795	80422809	SEMA3C
chr7	80535145	80535232	SEMA3C
chr7	80551581	80551596	SEMA3C
chr7	80804829	80804897	AC005008.2
chr7	80805200	80805305	AC005008.2
chr7	80817106	80817246	AC005008.2
chr7	80819221	80819265	AC005008.2
chr7	82478224	82478237	PCLO
chr7	82497456	82497511	PCLO
chr7	83246024	83246028	SEMA3E
chr7	86274089	86274188	GRM3
chr7	86540938	86541011	KIAA1324L
chr7	87471351	87471356	SLC25A40
chr7	87844238	87844251	SRI
chr7	89792288	89792374	STEAP2-AS1
chr7	89792288	89792374	STEAP1
chr7	89969833	89969838	GTPBP10
chr7	89971559	89971636	GTPBP10
chr7	91599787	91599822	AKAP9
chr7	91771777	91771800	CTB-161K23.1
chr7	91771777	91771800	CYP51A1
chr7	91771777	91771800	LRRD1
chr7	91779221	91779239	CTB-161K23.1
chr7	91779221	91779239	LRRD1
chr7	91955242	91955247	ANKIB1
chr7	91977261	91977330	ANKIB1
chr7	93070839	93070861	CALCR
chr7	93605264	93605412	BET1
chr7	93605264	93605412	AC006378.2
chr7	93628970	93629031	BET1
chr7	93628970	93629031	AC006378.2
chr7	94273957	94273979	SGCE
chr7	95171684	95172106	AC002451.1
chr7	97856054	97856081	TECPR1
chr7	98466828	98466855	TMEM130
chr7	98933076	98933107	ARPC1A
chr7	98937605	98937632	ARPC1A
chr7	98986277	98986279	ARPC1B
chr7	99009373	99009392	BUD31
chr7	99012883	99012889	BUD31
chr7	99039067	99039110	PTCD1

chr7	99039067	99039110	ATP5J2-PTCD1
chr7	99039067	99039110	CPSF4
chr7	99040552	99040576	AC073063.10
chr7	99040552	99040576	AC073063.1
chr7	99040552	99040576	PTCD1
chr7	99040552	99040576	ATP5J2-PTCD1
chr7	99040552	99040576	CPSF4
chr7	99040722	99040747	AC073063.10
chr7	99040722	99040747	AC073063.1
chr7	99040722	99040747	PTCD1
chr7	99040722	99040747	ATP5J2-PTCD1
chr7	99040722	99040747	CPSF4
chr7	99045396	99045472	PTCD1
chr7	99045396	99045472	ATP5J2-PTCD1
chr7	99045396	99045472	CPSF4
chr7	99058140	99058152	PTCD1
chr7	99058140	99058152	ATP5J2-PTCD1
chr7	99058140	99058152	ATP5J2
chr7	99160811	99160833	ZNF655
chr7	99160811	99160833	GS1-259H13.10
chr7	99447602	99447722	CYP3A43
chr7	99699869	99699916	AP4M1
chr7	100229122	100229219	TFR2
chr7	100468683	100468799	TRIP6
chr7	100478317	100478347	SRRT
chr7	100480385	100480387	SRRT
chr7	100606196	100606311	MUC3A
chr7	100606738	100606802	MUC3A
chr7	100607746	100607894	RP11-395B7.2
chr7	100607746	100607894	MUC3A
chr7	100608307	100608372	RP11-395B7.2
chr7	100608307	100608372	MUC3A
chr7	100608729	100608891	RP11-395B7.2
chr7	100608729	100608891	MUC3A
chr7	100609539	100609712	RP11-395B7.2
chr7	100609539	100609712	MUC3A
chr7	100609805	100609896	RP11-395B7.2
chr7	100609805	100609896	MUC3A
chr7	100610052	100610104	RP11-395B7.2
chr7	100610052	100610104	MUC3A
chr7	100610274	100610315	RP11-395B7.2
chr7	100610274	100610315	MUC3A
chr7	100734919	100735017	TRIM56
chr7	100861130	100861250	ZNHIT1
chr7	100861130	100861250	PLOD3
chr7	101952400	101952566	SH2B2
chr7	101952825	101952908	SH2B2

chr7	102185864	102185867	POLR2J3
chr7	102185864	102185867	RP11-514P8.7
chr7	102723513	102723579	ARMC10
chr7	102967779	102967825	PMPCB
chr7	102967779	102967825	DNAJC2
chr7	104110492	104110502	LHFPL3
chr7	104263451	104263471	LHFPL3
chr7	104704475	104704493	KMT2E
chr7	104748991	104749029	KMT2E
chr7	104946883	104947018	RP4-778K6.3
chr7	104946883	104947018	SRPK2
chr7	105176019	105176056	RINT1
chr7	105290006	105290008	ATXN7L1
chr7	105668108	105668118	CDHR3
chr7	107208200	107208230	DUS4L
chr7	107332499	107332522	SLC26A4
chr7	107392990	107393012	CBLL1
chr7	107545108	107545115	DLD
chr7	107642572	107642829	LAMB1
chr7	108540389	108540655	FLJ00325
chr7	111424440	111424462	DOCK4
chr7	111480819	111480926	DOCK4
chr7	112120891	112120932	LSMEM1
chr7	112120891	112120932	IFRD1
chr7	112126407	112126459	LSMEM1
chr7	112575242	112575319	C7orf60
chr7	113091127	113091456	TSRM
chr7	114103129	114103209	FOXP2
chr7	114178381	114178386	FOXP2
chr7	114296495	114296602	FOXP2
chr7	114654517	114654524	MDFIC
chr7	116595139	116595207	ST7
chr7	116595139	116595207	ST7-OT4
chr7	116596477	116596785	ST7
chr7	116596477	116596785	ST7-OT4
chr7	116598643	116598739	ST7
chr7	116598643	116598739	ST7-OT4
chr7	116599207	116599346	ST7
chr7	116599207	116599346	ST7-OT4
chr7	116606149	116606176	AC106873.4
chr7	116606149	116606176	ST7
chr7	116606149	116606176	ST7-OT4
chr7	116607345	116607475	AC106873.4
chr7	116607345	116607475	ST7
chr7	116607345	116607475	ST7-OT4
chr7	116608453	116608468	ST7
chr7	116608453	116608468	ST7-OT4

chr7	117355812	117355913	CFTR
chr7	117355812	117355913	CTTNBP2
chr7	117395602	117395628	CTTNBP2
chr7	117426440	117426479	CTTNBP2
chr7	117867986	117868045	ANKRD7
chr7	120380898	120380932	KCND2
chr7	120454269	120454301	TSPAN12
chr7	120633955	120634065	CPED1
chr7	121722861	121722926	AASS
chr7	121722931	121722945	AASS
chr7	122826792	122826803	SLC13A1
chr7	122830644	122830667	SLC13A1
chr7	123102854	123102886	IQUB
chr7	123186842	123186871	NDUFA5
chr7	123516080	123516085	HYAL4
chr7	124462582	124462605	POT1
chr7	124488640	124488713	POT1
chr7	126341811	126341881	GRM8
chr7	126716172	126716290	GRM8
chr7	127234548	127234628	FSCN3
chr7	127234834	127234848	FSCN3
chr7	128118003	128118031	RP11-212P7.3
chr7	128118003	128118031	METTL2B
chr7	128142668	128142775	METTL2B
chr7	128364823	128364874	FAM71F1
chr7	128583113	128583188	IRF5
chr7	128851224	128851246	RP11-286H14.8
chr7	128851224	128851246	SMO
chr7	128923225	128923265	AHCYL2
chr7	129017379	129017464	AHCYL2
chr7	129504898	129504905	UBE2H
chr7	129588799	129588899	UBE2H
chr7	129683691	129683720	ZC3HC1
chr7	129685322	129685328	ZC3HC1
chr7	129846717	129846818	TMEM209
chr7	129913546	129913614	CPA2
chr7	130066488	130066600	CEP41
chr7	130082238	130082261	CEP41
chr7	132409747	132409791	AC009365.3
chr7	132412194	132412601	AC009365.3
chr7	132500278	132500389	CHCHD3
chr7	132707166	132707255	CHCHD3
chr7	132720725	132720747	CHCHD3
chr7	134605386	134605471	CALD1
chr7	134612800	134612841	CALD1
chr7	134647571	134647669	CALD1
chr7	134694793	134695016	AGBL3

chr7	134735773	134735797	AGBL3
chr7	134784711	134784831	C7orf49
chr7	134784711	134784831	AGBL3
chr7	134785908	134786029	C7orf49
chr7	134785908	134786029	AGBL3
chr7	134832797	134832916	AC083862.1
chr7	134832797	134832916	C7orf49
chr7	134832797	134832916	TMEM140
chr7	135298469	135298513	NUP205
chr7	135330589	135330596	NUP205
chr7	137607336	137607513	CREB3L2
chr7	138281905	138281994	SVOPL
chr7	139060255	139060287	C7orf55-LUC7L2
chr7	139060255	139060287	LUC7L2
chr7	139421823	139421841	HIPK2
chr7	139605900	139605906	TBXAS1
chr7	139725348	139725382	PARP12
chr7	139795456	139795511	KDM7A
chr7	140079105	140079107	SLC37A3
chr7	140110773	140110855	RAB19
chr7	140173044	140173081	MKRN1
chr7	140174193	140174289	MKRN1
chr7	140175018	140175135	MKRN1
chr7	140395827	140395985	NDUFB2-AS1
chr7	140395827	140395985	NDUFB2
chr7	140395827	140395985	ADCK2
chr7	140397875	140397881	NDUFB2
chr7	140398001	140398068	NDUFB2
chr7	140447215	140447247	BRAF
chr7	140508095	140508116	BRAF
chr7	141538774	141538804	PRSS37
chr7	142099455	142099752	TRBV7-8
chr7	142099890	142099938	TRBV7-8
chr7	142104121	142104415	TRBV6-9
chr7	142104505	142104553	TRBV6-9
chr7	142960259	142960300	GSTK1
chr7	142960259	142960300	AC073342.12
chr7	143020912	143020931	CLCN1
chr7	143081272	143081338	ZYX
chr7	143084855	143084880	ZYX
chr7	143318048	143318113	FAM115C
chr7	147335901	147335970	CNTNAP2
chr7	148516131	148516151	EZH2
chr7	148724941	148725084	PDIA4
chr7	148948301	148948418	ZNF212
chr7	148982564	148982587	RP4-800G7.2
chr7	148982564	148982587	ZNF783

chr7	148984656	148984867	RP4-800G7.2
chr7	148984656	148984867	ZNF783
chr7	148985712	148985782	RP4-800G7.2
chr7	148985712	148985782	ZNF783
chr7	148987029	148987174	RP4-800G7.2
chr7	148987029	148987174	ZNF783
chr7	148989302	148989399	RP4-800G7.2
chr7	148989302	148989399	ZNF783
chr7	148990468	148990673	RP4-800G7.2
chr7	148990468	148990673	ZNF783
chr7	148990842	148991046	RP4-800G7.2
chr7	148990842	148991046	ZNF783
chr7	149573062	149573149	ATP6V0E2
chr7	149573062	149573149	ATP6V0E2-AS1
chr7	150708876	150708930	NOS3
chr7	150717375	150717434	ATG9B
chr7	150729931	150729982	ABCB8
chr7	150733948	150733951	ABCB8
chr7	150934042	150934068	CHPF2
chr7	151049179	151049279	NUB1
chr7	151263659	151263699	PRKAG2
chr7	154720359	154720477	PAXIP1-AS2
chr7	154720666	154720734	PAXIP1-AS2
chr7	154860926	154860955	HTR5A-AS1
chr7	154861986	154862163	HTR5A
chr7	154861986	154862163	HTR5A-AS1
chr7	154988630	154988697	AC099552.4
chr7	154988972	154989103	AC099552.4
chr7	154989979	154990010	AC099552.4
chr7	155150335	155150874	BLACE
chr7	155174819	155175010	AC008060.7
chr7	155187741	155187869	AC008060.7
chr7	155403994	155404111	AC009403.2
chr7	155436162	155436262	AC009403.2
chr7	155499988	155500029	RBM33
chr7	155593221	155593361	SHH
chr7	156432698	156433348	C7orf13
chr7	156432698	156433348	RNF32
chr7	156432698	156433348	LINC01006
chr7	156439148	156439161	RNF32
chr7	156572394	156572426	LMBR1
chr7	156903160	156903573	AC006967.1
chr7	156957113	156957169	UBE3C
chr7	157162054	157162097	DNAJB6
chr7	157318547	157319038	AC006372.1
chr7	157406721	157406793	AC005481.5
chr7	157406721	157406793	PTPRN2

chr7	157408081	157408358	AC005481.5
chr7	157408081	157408358	PTPRN2
chr7	158545472	158545534	ESYT2
chr7	158662137	158662181	WDR60
chr8	28905	29093	AC144568.2
chr8	2986334	2986422	CSMD1
chr8	7300397	7300554	SPAG11B
chr8	7306748	7306758	SPAG11B
chr8	7719831	7719841	SPAG11A
chr8	7726041	7726198	SPAG11A
chr8	8046158	8046331	LRLE1
chr8	8046158	8046331	ENPP7P1
chr8	8046158	8046331	FAM85B
chr8	8861584	8861610	ERI1
chr8	8866542	8866554	ERI1
chr8	9009359	9009426	RP11-10A14.4
chr8	9011716	9011853	RP11-10A14.4
chr8	9012476	9012572	RP11-10A14.4
chr8	9600974	9600976	TNKS
chr8	10544590	10544648	C8orf74
chr8	10544590	10544648	RP1L1
chr8	10548380	10548441	C8orf74
chr8	10548380	10548441	RP1L1
chr8	10645325	10645335	PINX1
chr8	10645325	10645335	SOX7
chr8	10645325	10645335	SOX7
chr8	10859898	10859991	XKR6
chr8	10971584	10971602	XKR6
chr8	10986311	10986553	AF131215.5
chr8	10986311	10986553	XKR6
chr8	11155409	11155423	MTMR9
chr8	11292575	11292611	FAM167A
chr8	11292575	11292611	C8orf12
chr8	11295549	11295719	FAM167A
chr8	11295549	11295719	C8orf12
chr8	11295945	11296051	FAM167A
chr8	11295945	11296051	C8orf12
chr8	11618923	11619615	C8orf49
chr8	11659318	11659529	RP11-297N6.4
chr8	11659318	11659529	FDFT1
chr8	11659930	11659996	RP11-297N6.4
chr8	11659930	11659996	FDFT1
chr8	11660831	11660869	FDFT1
chr8	11870658	11870660	RP11-481A20.11
chr8	11872340	11872558	RP11-481A20.11
chr8	12051120	12051193	FAM86B1
chr8	15978643	15978689	MSR1

chr8	16122682	16122710	MSR1
chr8	17105819	17105926	VPS37A
chr8	17129502	17129572	VPS37A
chr8	17581646	17581671	MTUS1
chr8	18659841	18659862	PSD3
chr8	18660684	18660789	PSD3
chr8	19315285	19315327	CSGALNACT1
chr8	19699338	19699345	INTS10
chr8	19804053	19804084	LPL
chr8	20006016	20006027	SLC18A1
chr8	20061470	20061519	ATP6V1B2
chr8	20082793	20082839	ATP6V1B2
chr8	21947272	21947365	FAM160B2
chr8	22014509	22014597	LGI3
chr8	22014509	22014597	SFTPC
chr8	22085161	22085181	PHYHIP
chr8	22381156	22381190	PPP3CC
chr8	22430850	22430935	SORBS3
chr8	22432487	22432499	SORBS3
chr8	22438475	22438498	PDLIM2
chr8	22492007	22492038	BIN3
chr8	23105500	23105710	CHMP7
chr8	23109881	23109883	CHMP7
chr8	23152972	23153021	R3HCC1
chr8	23261467	23261586	ENTPD4
chr8	23261467	23261586	LOXL2
chr8	23282564	23282603	ENTPD4
chr8	23282564	23282603	LOXL2
chr8	23412287	23412318	SLC25A37
chr8	23412287	23412318	AC051642.1
chr8	23420639	23420651	SLC25A37
chr8	23420639	23420651	AC051642.1
chr8	23431128	23431469	FP15737
chr8	23431128	23431469	SLC25A37
chr8	24166154	24166180	ADAM28
chr8	24166154	24166180	RP11-624C23.1
chr8	24247642	24247679	RP11-624C23.1
chr8	24247642	24247679	ADAMDEC1
chr8	26191002	26191021	PPP2R2A
chr8	26362823	26362835	PNMA2
chr8	26362823	26362835	BNIP3L
chr8	26434629	26434883	DPYSL2
chr8	26943765	26943802	RP11-521M14.2
chr8	26944696	26944766	RP11-521M14.2
chr8	27143643	27143654	TRIM35
chr8	27469118	27469121	CLU
chr8	27622046	27622050	CCDC25

chr8	28567979	28568020	EXTL3
chr8	28867201	28867238	HMBOX1
chr8	29107502	29107601	KIF13B
chr8	29962250	29962438	LEPROTL1
chr8	30023232	30023257	LEPROTL1
chr8	30023232	30023257	DCTN6
chr8	30395095	30395129	RBPMS
chr8	30494452	30494477	GTF2E2
chr8	30494762	30494791	GTF2E2
chr8	30670302	30670369	PPP2CB
chr8	32606199	32606212	NRG1
chr8	33251736	33251773	FUT10
chr8	35650399	35650815	UNC5D
chr8	35650399	35650815	AC012215.1
chr8	36780501	36780562	KCNU1
chr8	37593398	37594015	RP11-863K10.7
chr8	37641709	37641848	GPR124
chr8	37963549	37963657	ASH2L
chr8	38028511	38028530	LSM1
chr8	38286814	38286908	FGFR1
chr8	38964568	38964718	ADAM32
chr8	39463247	39463280	ADAM18
chr8	40644218	40644301	ZMAT4
chr8	40666052	40666090	ZMAT4
chr8	42013497	42013553	AP3M2
chr8	42025715	42025767	AP3M2
chr8	42328691	42328719	SLC20A2
chr8	42849577	42849592	HOOK3
chr8	42855414	42855419	HOOK3
chr8	42882572	42882575	RP11-598P20.5
chr8	42882572	42882575	HOOK3
chr8	43023087	43023192	HGSNAT
chr8	48243369	48243388	SPIDR
chr8	48956059	48956067	UBE2V2
chr8	48962820	48962978	UBE2V2
chr8	50449023	50449057	RP11-738G5.2
chr8	50449716	50449811	RP11-738G5.2
chr8	50457010	50457076	RP11-738G5.2
chr8	52730402	52730497	AC090186.1
chr8	52730402	52730497	PCMTD1
chr8	53122638	53122677	ST18
chr8	53599458	53599527	RB1CC1
chr8	53626635	53626693	RB1CC1
chr8	54155379	54155472	OPRK1
chr8	54719404	54719418	ATP6V1H
chr8	54915483	54915500	TCEA1
chr8	54915635	54915646	TCEA1

chr8	56048154	56048212	RP11-386G21.1
chr8	56048154	56048212	XKR4
chr8	56052650	56052776	RP11-386G21.1
chr8	56052650	56052776	XKR4
chr8	56054683	56054688	RP11-386G21.1
chr8	56054683	56054688	XKR4
chr8	56074186	56074204	RP11-386G21.2
chr8	56074186	56074204	XKR4
chr8	56076678	56076853	RP11-386G21.2
chr8	56076678	56076853	XKR4
chr8	56076973	56077004	RP11-386G21.2
chr8	56076973	56077004	XKR4
chr8	56637786	56637818	TMEM68
chr8	56664089	56664105	TMEM68
chr8	57349498	57349635	PENK
chr8	57876930	57876977	IMPAD1
chr8	57879704	57879777	IMPAD1
chr8	59549900	59549948	NSMAF
chr8	59552646	59552661	NSMAF
chr8	61482514	61482616	RAB2A
chr8	61488793	61488856	RAB2A
chr8	62200764	62200793	RP11-787D18.2
chr8	62200764	62200793	CLVS1
chr8	62204877	62204975	CLVS1
chr8	64086210	64086240	YTHDF3
chr8	64101293	64101376	YTHDF3
chr8	67365945	67365969	ADHFE1
chr8	67411060	67411080	C8orf46
chr8	67972687	67972780	COPS5
chr8	67973627	67973657	COPS5
chr8	67987041	67987142	COPS5
chr8	67987041	67987142	CSPP1
chr8	69217005	69217036	RP11-664D7.4
chr8	69218753	69218833	RP11-664D7.4
chr8	69442828	69442843	C8orf34
chr8	70850403	70850618	AC090574.1
chr8	71486420	71486566	AC120194.1
chr8	71486420	71486566	TRAM1
chr8	71572797	71573429	RP11-382J12.1
chr8	71572797	71573429	LACTB2
chr8	71595748	71595853	XKR9
chr8	72137472	72137540	EYA1
chr8	72317040	72317149	RP11-1102P16.1
chr8	72383093	72383166	RP11-1102P16.1
chr8	72875158	72875209	RP11-383H13.1
chr8	72875248	72875283	RP11-383H13.1
chr8	72877644	72877769	RP11-383H13.1

chr8	72914457	72914504	RP11-383H13.1
chr8	72932213	72932258	TRPA1
chr8	72932213	72932258	RP11-383H13.1
chr8	74473764	74473774	STAU2
chr8	74891314	74891333	TMEM70
chr8	75525103	75525175	RP11-730G20.1
chr8	75525103	75525175	RP11-758M4.1
chr8	75614614	75614691	RP11-758M4.1
chr8	75615841	75615917	RP11-758M4.1
chr8	75664676	75664753	RP11-758M4.1
chr8	77694379	77694454	ZFHX4
chr8	77746357	77746376	ZFHX4
chr8	80964859	80964910	TPD52
chr8	81032050	81032119	TPD52
chr8	82587034	82587066	IMPA1
chr8	82590818	82590924	IMPA1
chr8	82621047	82621064	ZFAND1
chr8	86126395	86126540	C8orf59
chr8	86126395	86126540	E2F5
chr8	86134884	86135082	CA13
chr8	86134884	86135082	RP11-219B4.5
chr8	86138738	86138805	CA13
chr8	86138738	86138805	RP11-219B4.5
chr8	86158554	86158783	RP11-219B4.6
chr8	86158554	86158783	CA13
chr8	86162053	86162077	RP11-219B4.6
chr8	86162053	86162077	CA13
chr8	86385618	86385691	CA2
chr8	86573699	86575726	REXO1L1P
chr8	87452855	87452962	WWP1
chr8	87480549	87480652	RMDN1
chr8	87480549	87480652	WWP1
chr8	87490303	87490305	RMDN1
chr8	87490303	87490305	WWP1
chr8	87507116	87507195	RMDN1
chr8	87507116	87507195	CPNE3
chr8	87517595	87517608	RMDN1
chr8	87517595	87517608	CPNE3
chr8	87581556	87581615	CNGB3
chr8	88483476	88483535	CNBD1
chr8	88499816	88499932	CNBD1
chr8	88622037	88622082	AF121898.3
chr8	88622037	88622082	CNBD1
chr8	88622767	88622806	AF121898.3
chr8	88622767	88622806	CNBD1
chr8	88624234	88624291	AF121898.3
chr8	88624234	88624291	CNBD1

chr8	89340139	89340209	RP11-586K2.1
chr8	89340139	89340209	MMP16
chr8	90771550	90771589	RIPK2
chr8	90994225	90994230	NBN
chr8	91017595	91017597	DECR1
chr8	92130452	92130478	LRRC69
chr8	93015558	93015608	RUNX1T1
chr8	93897043	93897258	CTD-3239E11.2
chr8	93897043	93897258	AC117834.1
chr8	93897043	93897258	TRIQK
chr8	94147604	94147664	C8orf87
chr8	94241867	94242146	AC016885.1
chr8	94241867	94242146	RP11-388K12.1
chr8	94241867	94242146	LINC00535
chr8	94242198	94242388	AC016885.1
chr8	94242198	94242388	RP11-388K12.1
chr8	94242198	94242388	LINC00535
chr8	94714393	94714530	FAM92A1
chr8	94714748	94714771	FAM92A1
chr8	94719430	94719489	FAM92A1
chr8	94736738	94736823	FAM92A1
chr8	94739359	94739397	FAM92A1
chr8	94752481	94752789	RBM12B-AS1
chr8	94752481	94752789	RBM12B
chr8	94775178	94775215	TMEM67
chr8	94804110	94804232	TMEM67
chr8	95559047	95559292	AC023632.1
chr8	95559047	95559292	KIAA1429
chr8	95756571	95756613	DPY19L4
chr8	95756713	95756761	DPY19L4
chr8	95825660	95825666	INTS8
chr8	95835088	95835409	INTS8
chr8	96046236	96046300	NDUFAF6
chr8	96048599	96048664	NDUFAF6
chr8	97246703	97246764	UQCRB
chr8	99046402	99046428	RPL30
chr8	99046402	99046428	MATN2
chr8	100973414	100973424	RGS22
chr8	101066650	101066652	RGS22
chr8	101137979	101138026	RGS22
chr8	101143360	101143392	RGS22
chr8	101669952	101670095	SNX31
chr8	102213206	102213232	ZNF706
chr8	102700871	102700948	KB-1107E3.1
chr8	102700871	102700948	NCALD
chr8	103246953	103247073	RRM2B
chr8	103541076	103541083	KB-1980E6.3

chr8	103541539	103541656	KB-1980E6.3
chr8	103876579	103876839	AZIN1
chr8	103876579	103876839	KB-1507C5.2
chr8	104145255	104145572	C8orf56
chr8	104319979	104319996	FZD6
chr8	105391982	105392071	DPYS
chr8	105727198	105727203	RP11-127H5.1
chr8	105839585	105839706	RP11-127H5.1
chr8	105845553	105845673	RP11-127H5.1
chr8	105907635	105907679	RP11-127H5.1
chr8	106003852	106003897	RP11-127H5.1
chr8	106258521	106258588	RP11-127H5.1
chr8	109009140	109009149	RSPO2
chr8	109245854	109245901	EIF3E
chr8	109254405	109254485	EIF3E
chr8	109254528	109254575	EIF3E
chr8	109458488	109458530	EMC2
chr8	110492780	110492890	PKHD1L1
chr8	110569960	110569992	EBAG9
chr8	114612622	114612667	RP11-67H2.1
chr8	114627733	114627773	RP11-67H2.1
chr8	116504415	116504448	TRPS1
chr8	117701308	117701379	EIF3H
chr8	117709292	117709476	EIF3H
chr8	117746516	117746563	EIF3H
chr8	117746585	117746593	EIF3H
chr8	117749488	117749526	EIF3H
chr8	117791722	117791825	UTP23
chr8	117798742	117798816	UTP23
chr8	118843838	118843892	EXT1
chr8	119294558	119294569	AC023590.1
chr8	119294558	119294569	SAMD12
chr8	119296511	119296609	AC023590.1
chr8	119296511	119296609	SAMD12
chr8	119942970	119943007	TNFRSF11B
chr8	120630554	120630563	RP11-99I9.2
chr8	120630554	120630563	ENPP2
chr8	120773931	120774012	TAF2
chr8	121382976	121383073	COL14A1
chr8	124114487	124114510	TBC1D31
chr8	124798244	124798265	FAM91A1
chr8	124824296	124824298	FAM91A1
chr8	125739802	125739857	MTSS1
chr8	126168242	126168289	NSMCE2
chr8	128959864	128960280	TMEM75
chr8	128959864	128960280	PVT1
chr8	131353088	131353183	ASAP1

chr8	132947755	132947764	EFR3A
chr8	133620683	133620799	LRRC6
chr8	133655919	133655932	LRRC6
chr8	133699865	133699901	TMEM71
chr8	133819968	133819993	PHF20L1
chr8	134071331	134071437	TG
chr8	134071331	134071437	SLA
chr8	134267530	134267643	NDRG1
chr8	134297000	134297139	NDRG1
chr8	134308747	134308750	NDRG1
chr8	134310621	134310635	NDRG1
chr8	134314231	134314263	NDRG1
chr8	135522305	135522425	ZFAT
chr8	135612043	135612105	ZFAT-AS1
chr8	135612043	135612105	ZFAT
chr8	135686974	135687131	ZFAT
chr8	139321227	139321275	FAM135B
chr8	139379599	139379653	FAM135B
chr8	140944530	140944829	C8orf17
chr8	140944530	140944829	TRAPPC9
chr8	141522017	141522124	CHRAC1
chr8	141682252	141682256	PTK2
chr8	141762002	141762086	PTK2
chr8	142152399	142152585	DENND3
chr8	142160385	142160447	DENND3
chr8	143995379	143995461	CYP11B2
chr8	143995379	143995461	GML
chr8	143997832	143997922	CYP11B2
chr8	143997832	143997922	GML
chr8	144373011	144373028	ZNF696
chr8	144409895	144409960	TOP1MT
chr8	144410459	144410485	TOP1MT
chr8	144414657	144414827	TOP1MT
chr8	144425390	144425418	TOP1MT
chr8	144641041	144641084	GSDMD
chr8	144691003	144691046	PYCRL
chr8	144774171	144774226	ZNF707
chr8	145011917	145011952	PLEC
chr8	145032763	145032925	PLEC
chr8	145331100	145331153	KM-PA-2
chr8	145437947	145438539	FAM203B
chr8	145438618	145438718	FAM203B
chr8	145438826	145438924	FAM203B
chr8	145439120	145439211	FAM203B
chr8	145439300	145439422	FAM203B
chr8	145439499	145439663	FAM203B
chr8	145486120	145486273	BOP1

chr8	145486352	145486459	BOP1
chr8	145486544	145486628	BOP1
chr8	145486842	145487130	BOP1
chr8	145487210	145487390	BOP1
chr8	145487467	145487599	BOP1
chr8	145487681	145487750	BOP1
chr8	145487820	145487900	BOP1
chr8	145487963	145488131	BOP1
chr8	145488214	145488426	BOP1
chr8	145488504	145488605	BOP1
chr8	145488703	145488820	BOP1
chr8	145488835	145489060	BOP1
chr8	145490603	145491169	SCXA
chr8	145490603	145491169	BOP1
chr8	145492093	145492131	SCXA
chr8	145492093	145492131	BOP1
chr8	145601082	145601179	ADCK5
chr8	145614463	145614536	ADCK5
chr8	145980790	145980826	ZNF251
chr8	146016144	146016148	RPL8
chr8	146056316	146056414	ZNF7
chr8	146105821	146105843	ZNF250
chr9	160120	160140	CBWD1
chr9	220748	220847	DOCK8
chr9	276994	277038	DOCK8
chr9	365591	365624	DOCK8
chr9	735712	735780	KANK1
chr9	3323982	3324129	RFX3
chr9	3452305	3452406	AL365202.1
chr9	3452305	3452406	RFX3
chr9	3453526	3453708	AL365202.1
chr9	3453526	3453708	RFX3
chr9	3469155	3469181	AL365202.1
chr9	3469155	3469181	RFX3
chr9	4627830	4627856	SPATA6L
chr9	4859120	4859260	AL158147.2
chr9	4859120	4859260	RCL1
chr9	5077163	5077181	AL161450.1
chr9	5077163	5077181	JAK2
chr9	5084568	5084580	AL161450.1
chr9	5084568	5084580	JAK2
chr9	5791234	5791282	ERMP1
chr9	6468385	6468741	C9orf38
chr9	6468385	6468741	UHRF2
chr9	6492303	6492317	UHRF2
chr9	6981842	6981848	KDM4C
chr9	14307907	14307921	NFIB

chr9	14774063	14774158	FREM1
chr9	15249960	15250008	TTC39B
chr9	15883072	15883161	CCDC171
chr9	16429925	16429997	BNC2
chr9	18890552	18890555	ADAMTSL1
chr9	20927832	20927890	FOCAD
chr9	21967199	21967438	C9orf53
chr9	21967199	21967438	RP11-145E5.5
chr9	26976775	26976780	IFT74
chr9	26982284	26982397	IFT74
chr9	32498310	32498337	DDX58
chr9	32567063	32567650	RP11-205M20.7
chr9	32567063	32567650	GVQW1
chr9	32567063	32567650	NDUFB6
chr9	33262103	33262244	BAG1
chr9	33391454	33391529	AQP7
chr9	33629119	33629189	TRBV21OR9-2
chr9	33629289	33629584	TRBV21OR9-2
chr9	33638033	33638083	TRBV23OR9-2
chr9	33638209	33638492	TRBV23OR9-2
chr9	34125603	34125617	DCAF12
chr9	34127252	34127275	DCAF12
chr9	34487944	34488189	DNAI1
chr9	34991340	34991413	DNAJB5
chr9	35104619	35104643	FAM214B
chr9	35361056	35361262	AL160274.1
chr9	35361056	35361262	UNC13B
chr9	35669581	35669610	ARHGEF39
chr9	35682705	35682901	TPM2
chr9	35749203	35749266	GBA2
chr9	35749203	35749266	RGP1
chr9	35749369	35749405	GBA2
chr9	35749369	35749405	RGP1
chr9	35821435	35821625	FAM221B
chr9	35821435	35821625	TMEM8B
chr9	36141843	36141868	GLIPR2
chr9	36263195	36263439	CLTA
chr9	36263195	36263439	GNE
chr9	36265418	36265470	CLTA
chr9	36265418	36265470	GNE
chr9	39885004	39885192	SPATA31A2
chr9	39886380	39886437	SPATA31A2
chr9	39887006	39887066	SPATA31A2
chr9	39887322	39891057	SPATA31A2
chr9	40028620	40028741	AL353791.1
chr9	40768558	40768619	ZNF658
chr9	41321260	41324995	SPATA31A4

chr9	41321260	41324995	RP11-95K23.3
chr9	41325251	41325311	SPATA31A4
chr9	41325880	41325937	SPATA31A4
chr9	41327147	41327364	SPATA31A4
chr9	42704004	42704049	CBWD7
chr9	43082880	43082885	ANKRD20A3
chr9	43089717	43089866	ANKRD20A3
chr9	43091352	43092326	ANKRD20A3
chr9	43093497	43093677	ANKRD20A3
chr9	43095105	43095268	ANKRD20A3
chr9	43099327	43099397	ANKRD20A3
chr9	43105373	43105445	RP11-327I22.4
chr9	43105373	43105445	ANKRD20A3
chr9	43105537	43105565	RP11-327I22.4
chr9	43105537	43105565	ANKRD20A3
chr9	43108274	43108358	RP11-327I22.4
chr9	43108274	43108358	ANKRD20A3
chr9	43112306	43112375	RP11-327I22.4
chr9	43112306	43112375	ANKRD20A3
chr9	43115062	43115092	ANKRD20A3
chr9	43121640	43121695	ANKRD20A3
chr9	43124293	43124430	ANKRD20A3
chr9	43125477	43125583	ANKRD20A3
chr9	43129467	43129640	ANKRD20A3
chr9	43129803	43129917	ANKRD20A3
chr9	43133230	43133432	ANKRD20A3
chr9	43135095	43135514	AL513478.1
chr9	43629263	43629320	SPATA31A6
chr9	43685295	43685379	CNTNAP3B
chr9	43709650	43709760	CNTNAP3B
chr9	43737327	43737520	CNTNAP3B
chr9	43800864	43801011	CNTNAP3B
chr9	43818041	43818184	CNTNAP3B
chr9	43819610	43819681	CNTNAP3B
chr9	43828078	43828221	CNTNAP3B
chr9	43905604	43905828	CNTNAP3B
chr9	43907404	43907537	CNTNAP3B
chr9	43908436	43908523	CNTNAP3B
chr9	43915360	43915590	CNTNAP3B
chr9	43915826	43915897	CNTNAP3B
chr9	43920249	43920370	CNTNAP3B
chr9	44325916	44326077	BX088651.2
chr9	44401890	44402255	BX088651.1
chr9	44401890	44402255	RP11-475I24.3
chr9	44869083	44869140	RP11-160N1.10
chr9	44869923	44870104	RP11-160N1.10
chr9	44990451	44990528	FAM27C

chr9	44991083	44991208	FAM27C
chr9	45441273	45441542	AL354718.1
chr9	45727244	45727321	FAM27A
chr9	45727874	45727999	FAM27A
chr9	45733588	45733965	RP11-7G23.8
chr9	45733588	45733965	FAM27E2
chr9	46386622	46387002	FAM27E1
chr9	46390277	46390900	FAM27D1
chr9	47299530	47299651	AL953854.2
chr9	47304003	47304074	AL953854.2
chr9	47304310	47304540	AL953854.2
chr9	47311409	47311496	AL953854.2
chr9	47312365	47312528	AL953854.2
chr9	47314098	47314322	AL953854.2
chr9	65507507	65507567	SPATA31A7
chr9	67785913	67786254	FAM27E3
chr9	67785913	67786254	RP11-12A20.2
chr9	67793213	67793338	RP11-12A20.7
chr9	67793213	67793338	FAM27B
chr9	67793897	67793974	RP11-12A20.7
chr9	67793897	67793974	FAM27B
chr9	67924791	67925210	BX649567.1
chr9	67945206	67945236	ANKRD20A1
chr9	67954722	67954750	ANKRD20A1
chr9	67954842	67954914	ANKRD20A1
chr9	67960882	67960952	ANKRD20A1
chr9	67965005	67965168	ANKRD20A1
chr9	67966593	67966773	ANKRD20A1
chr9	67984833	67985114	RP11-195B21.3
chr9	67987826	67987998	RP11-195B21.3
chr9	69200359	69201612	FOXD4L6
chr9	69204963	69205069	CBWD6
chr9	69205457	69205586	CBWD6
chr9	69206680	69206799	CBWD6
chr9	69206903	69206971	CBWD6
chr9	69218510	69218561	CBWD6
chr9	69229612	69229668	CBWD6
chr9	69235562	69235606	CBWD6
chr9	69238230	69238316	CBWD6
chr9	69240013	69240059	CBWD6
chr9	69245941	69245978	CBWD6
chr9	69247522	69247581	CBWD6
chr9	69252845	69253282	BX255923.1
chr9	69252845	69253282	CBWD6
chr9	69261239	69261284	CBWD6
chr9	69380011	69380430	CR769776.1
chr9	69653111	69653179	AL445665.1

chr9	69655521	69655673	AL445665.1
chr9	70427649	70428899	FOXD4L4
chr9	70432429	70432535	CBWD5
chr9	70432923	70433052	CBWD5
chr9	70434178	70434243	CBWD5
chr9	70434347	70434415	CBWD5
chr9	70445953	70446004	CBWD5
chr9	70457087	70457143	CBWD5
chr9	70463031	70463075	CBWD5
chr9	70465708	70465794	CBWD5
chr9	70467484	70467530	CBWD5
chr9	70471120	70471140	CBWD5
chr9	70472645	70472659	CBWD5
chr9	70473437	70473474	CBWD5
chr9	70475019	70475078	CBWD5
chr9	70483188	70483279	CBWD5
chr9	70484371	70484471	CBWD5
chr9	70486793	70486879	CBWD5
chr9	70488820	70488865	CBWD5
chr9	70488895	70488917	CBWD5
chr9	70489915	70490068	CBWD5
chr9	70646864	70647145	AL591479.1
chr9	70866135	70866572	AL353608.1
chr9	70866135	70866572	CBWD3
chr9	71439012	71439131	RP11-203L2.4
chr9	71439012	71439131	PIP5K1B
chr9	71578099	71578126	PIP5K1B
chr9	74314293	74314365	TMEM2
chr9	74829675	74829792	GDA
chr9	74979378	74979524	ZFAND5
chr9	77352311	77352366	TRPM6
chr9	77641969	77642015	C9orf41
chr9	78809657	78809679	PCSK5
chr9	79003138	79003202	RFK
chr9	79920473	79920538	VPS13A
chr9	80645431	80645479	GNAQ
chr9	82216330	82216350	TLE4
chr9	82286124	82286253	TLE4
chr9	82308549	82308575	TLE4
chr9	85912686	85912731	FRMD3
chr9	86241357	86241644	IDNK
chr9	86407912	86407932	GKAP1
chr9	90316751	90316825	DAPK1
chr9	95055723	95055727	IARS
chr9	95063010	95063092	NOL8
chr9	95082395	95082419	NOL8
chr9	96210733	96210771	FAM120AOS

chr9	97059097	97059131	ZNF169
chr9	97080300	97080338	NUTM2F
chr9	97525409	97525412	C9orf3
chr9	97766506	97766568	C9orf3
chr9	97768282	97768404	C9orf3
chr9	98236330	98236425	PTCH1
chr9	98534606	98534956	DKFZP434H0512
chr9	98534606	98534956	LINC00476
chr9	98668933	98668992	ERCC6L2
chr9	98717010	98717181	ERCC6L2
chr9	99623884	99623885	ZNF782
chr9	99637750	99637882	ZNF782
chr9	100053684	100053769	RP11-23J9.7
chr9	100053684	100053769	RP11-23J9.5
chr9	100053684	100053769	CCDC180
chr9	100053684	100053769	RP11-23J9.4
chr9	100054838	100054957	RP11-23J9.7
chr9	100054838	100054957	RP11-23J9.5
chr9	100054838	100054957	CCDC180
chr9	100054838	100054957	RP11-23J9.4
chr9	100056229	100056392	RP11-23J9.7
chr9	100056229	100056392	RP11-23J9.5
chr9	100056229	100056392	CCDC180
chr9	100056229	100056392	RP11-23J9.4
chr9	100057127	100057273	RP11-23J9.7
chr9	100057127	100057273	RP11-23J9.5
chr9	100057127	100057273	CCDC180
chr9	100057127	100057273	RP11-23J9.4
chr9	100057902	100058047	RP11-23J9.7
chr9	100057902	100058047	RP11-23J9.5
chr9	100057902	100058047	CCDC180
chr9	100057902	100058047	RP11-23J9.4
chr9	100107888	100107978	CCDC180
chr9	100107888	100107978	RP11-23J9.4
chr9	100444591	100444712	XPA
chr9	100675184	100675257	C9orf156
chr9	100854780	100854822	TRIM14
chr9	101890216	101890284	TGFBR1
chr9	102678331	102678345	STX17
chr9	102678331	102678345	RP11-60I3.4
chr9	102708001	102708018	STX17
chr9	104151728	104151769	MRPL50
chr9	108398954	108399075	FKTN
chr9	110093950	110094003	RAD23B
chr9	110540006	110540380	AL162389.1
chr9	111637971	111638015	IKBKAP
chr9	112629352	112629354	PALM2

chr9	112629352	112629354	PALM2-AKAP2
chr9	112629352	112629354	AKAP2
chr9	112852661	112852696	PALM2-AKAP2
chr9	112852661	112852696	AKAP2
chr9	113220395	113220399	SVEP1
chr9	113468356	113468432	MUSK
chr9	113761673	113761720	LPAR1
chr9	113800932	113800979	LPAR1
chr9	114326519	114326542	PTGR1
chr9	114326519	114326542	ZNF483
chr9	114351650	114351662	PTGR1
chr9	114405119	114405208	DNAJC25
chr9	114405119	114405208	DNAJC25-GNG10
chr9	115246826	115246906	C9orf147
chr9	115248670	115248821	C9orf147
chr9	115249259	115249484	C9orf147
chr9	115249259	115249484	KIAA1958
chr9	115628765	115628785	SNX30
chr9	115920324	115920376	SLC31A2
chr9	116028516	116028674	CDC26
chr9	116028516	116028674	SLC31A1
chr9	116279550	116279586	RGS3
chr9	116980050	116980139	COL27A1
chr9	119160757	119161065	PAPPA-AS1
chr9	119160757	119161065	PAPPA
chr9	119199601	119199677	ASTN2
chr9	119199779	119199870	ASTN2
chr9	122257779	122257835	RP11-295D22.1
chr9	122275203	122275261	RP11-295D22.1
chr9	122285183	122285246	RP11-295D22.1
chr9	123280090	123280164	CDK5RAP2
chr9	123452204	123452273	MEGF9
chr9	123453688	123453752	MEGF9
chr9	125048059	125048106	MRRF
chr9	125048318	125048389	MRRF
chr9	125148344	125148406	AL162424.1
chr9	125148344	125148406	PTGS1
chr9	125560798	125560818	PDCL
chr9	125878292	125878377	STRBP
chr9	126763949	126763976	LHX2
chr9	127159039	127159126	PSMB7
chr9	127160720	127160751	PSMB7
chr9	127263227	127263265	NR5A1
chr9	127736530	127736555	SCAI
chr9	127737592	127737668	SCAI
chr9	127962884	127963185	RABEPK
chr9	128072208	128072288	GAPVD1

chr9	128209690	128209766	MAPKAP1
chr9	128627550	128627584	PBX3
chr9	129138725	129138907	MVB12B
chr9	130517115	130517120	SH2D3C
chr9	130524671	130524695	SH2D3C
chr9	130861554	130861622	SLC25A25
chr9	130890612	130890624	PTGES2
chr9	130890612	130890624	AL590708.2
chr9	130890799	130891481	AL590708.2
chr9	131040913	131040960	SWI5
chr9	131063193	131063618	AL359091.2
chr9	131602890	131602951	CCBL1
chr9	131604346	131604463	CCBL1
chr9	131778081	131778143	SH3GLB2
chr9	131813579	131813699	FAM73B
chr9	131881339	131881420	PPP2R4
chr9	132672446	132672456	FNBP1
chr9	132903812	132904381	AL360004.1
chr9	134105050	134105212	NUP214
chr9	134401879	134401890	UCK1
chr9	136253541	136253630	C9orf96
chr9	137978452	137978568	OLFM1
chr9	138150512	138150990	AL390778.1
chr9	138151020	138151275	AL390778.1
chr9	138644225	138644259	KCNT1
chr9	139219089	139219640	DKFZP434A062
chr9	139980807	139981121	AL807752.1
chr9	140275696	140275698	EXD3
chr9	140510121	140510651	C9orf37
chr9	140686507	140686572	EHMT1
chr9	140741012	140741026	EHMT1
chr9	140911591	140911653	CACNA1B
chr9	140927134	140927222	CACNA1B
chr9	140934999	140935124	CACNA1B
chr9	140963060	140963065	CACNA1B
chr9	140995556	140995652	CACNA1B
chr10	264395	264439	ZMYND11
chr10	370834	370959	DIP2C
chr10	410938	410961	DIP2C
chr10	1018440	1018871	AL359878.1
chr10	1120410	1120448	WDR37
chr10	3179735	3179762	PFKP
chr10	3183797	3183929	PITRM1
chr10	3183797	3183929	PITRM1-AS1
chr10	3185896	3185967	PITRM1
chr10	3185896	3185967	PITRM1-AS1
chr10	5196335	5196377	AKR1CL1

chr10	5197879	5197952	AKR1CL1
chr10	5199836	5200001	AKR1CL1
chr10	5200798	5200907	AKR1CL1
chr10	5202082	5202135	AKR1CL1
chr10	5202775	5202852	AKR1CL1
chr10	5203719	5203730	AKR1CL1
chr10	5203819	5203935	AKR1CL1
chr10	5204816	5204983	AKR1CL1
chr10	5226975	5227067	AKR1CL1
chr10	5244427	5244449	AKR1CL1
chr10	5244427	5244449	AKR1C4
chr10	5684512	5684516	ASB13
chr10	5853837	5853893	GDI2
chr10	6131130	6131156	RBM17
chr10	6266469	6266586	PFKFB3
chr10	6271072	6271105	PFKFB3
chr10	6392280	6392831	DKFZP667F0711
chr10	7601756	7601845	ITIH5
chr10	7642517	7642528	ITIH5
chr10	13164864	13164914	OPTN
chr10	13350145	13350447	AL138764.1
chr10	13364055	13364065	SEPHS1
chr10	13674560	13674563	RP11-295P9.3
chr10	14570011	14570038	FAM107B
chr10	14595373	14595386	FAM107B
chr10	14891432	14891475	HSPA14
chr10	15146436	15146454	RPP38
chr10	15146436	15146454	NMT2
chr10	15148039	15148058	RPP38
chr10	15148039	15148058	NMT2
chr10	17007971	17007976	CUBN
chr10	17164521	17164584	CUBN
chr10	17211503	17211538	TRDMT1
chr10	17360694	17360732	ST8SIA6
chr10	17714974	17714977	STAM
chr10	17882692	17882805	MRC1L1
chr10	17887289	17887435	MRC1L1
chr10	17891583	17891768	MRC1L1
chr10	17894976	17895133	MRC1L1
chr10	17896743	17896832	MRC1L1
chr10	17898252	17898362	MRC1L1
chr10	17903397	17903512	MRC1L1
chr10	17905544	17905692	MRC1L1
chr10	17908571	17908770	MRC1L1
chr10	17912258	17912385	MRC1L1
chr10	17912860	17912947	MRC1L1
chr10	17913994	17914138	MRC1L1

chr10	17915796	17915837	MRC1L1
chr10	17917102	17917265	MRC1L1
chr10	17919912	17919979	MRC1L1
chr10	17921733	17921833	MRC1L1
chr10	17922537	17922682	MRC1L1
chr10	17923079	17923193	MRC1L1
chr10	17927279	17927445	MRC1L1
chr10	17936221	17936323	MRC1L1
chr10	17940046	17940278	MRC1L1
chr10	17942802	17942967	MRC1L1
chr10	17943987	17944136	MRC1L1
chr10	17948902	17949015	MRC1L1
chr10	17949550	17949714	MRC1L1
chr10	17951322	17951363	MRC1L1
chr10	17952231	17952481	MRC1L1
chr10	18041262	18041518	TMEM236
chr10	18060292	18060364	TMEM236
chr10	18065018	18065159	TMEM236
chr10	18084907	18085490	TMEM236
chr10	18098455	18098515	MRC1
chr10	18112044	18112445	MRC1
chr10	18116495	18116668	MRC1
chr10	18122628	18122792	MRC1
chr10	19777889	19778102	HMGN1P20
chr10	19777889	19778102	MALRD1
chr10	21358883	21358944	NEBL
chr10	23632835	23632893	RP11-371A19.2
chr10	23632835	23632893	C10orf67
chr10	24770273	24770632	AL353583.1
chr10	24770273	24770632	KIAA1217
chr10	27029574	27029615	PDSS1
chr10	27442156	27442164	YME1L1
chr10	27793908	27794119	RAB18
chr10	28089761	28089781	ARMC4
chr10	28247800	28247901	ARMC4
chr10	28346686	28346778	MPP7
chr10	28823163	28823189	WAC
chr10	28900184	28900341	WAC
chr10	29606238	29606240	LYZL1
chr10	29785326	29785365	SVIL
chr10	30653635	30653996	MTPAP
chr10	30653635	30653996	GOLGA2P6
chr10	30654078	30654262	MTPAP
chr10	30654078	30654262	GOLGA2P6
chr10	31123111	31123141	ZNF438
chr10	31124496	31124569	ZNF438
chr10	31609055	31609136	ZEB1-AS1

chr10	31609055	31609136	ZEB1
chr10	31609535	31609627	ZEB1
chr10	31661947	31662050	RP11-192P3.5
chr10	31661947	31662050	ZEB1
chr10	31676053	31676155	RP11-192P3.5
chr10	31676053	31676155	ZEB1
chr10	32964675	32964741	C10ORF68
chr10	33195393	33195508	ITGB1
chr10	33484422	33484504	NRP1
chr10	33538349	33538477	NRP1
chr10	36811744	36813162	NAMPTL
chr10	38717129	38717224	LINC00999
chr10	38724058	38724181	LINC00999
chr10	38726018	38726134	LINC00999
chr10	38726472	38726512	LINC00999
chr10	43657648	43657669	CSGALNACT2
chr10	43698106	43698130	RASGEF1A
chr10	43932282	43932383	ZNF487
chr10	43977832	43978115	ZNF487
chr10	43978329	43978591	ZNF487
chr10	43991464	43991517	ZNF487
chr10	44793339	44793345	CXCL12
chr10	44873687	44873737	AL137026.1
chr10	44873687	44873737	CXCL12
chr10	44878713	44878739	AL137026.1
chr10	44878713	44878739	CXCL12
chr10	45476340	45476351	RASSF4
chr10	45478358	45478469	RASSF4
chr10	46135803	46135869	ZFAND4
chr10	46147054	46147073	ZFAND4
chr10	46551702	46551767	PTPN20A
chr10	46552469	46552531	PTPN20A
chr10	46564914	46565129	PTPN20A
chr10	46567905	46568239	PTPN20A
chr10	46584432	46584522	PTPN20A
chr10	46587344	46587495	PTPN20A
chr10	46604510	46604601	PTPN20A
chr10	46605763	46605875	PTPN20A
chr10	46608423	46608520	PTPN20A
chr10	46611717	46611811	PTPN20A
chr10	46619902	46619946	PTPN20A
chr10	46640599	46640605	PTPN20A
chr10	47158837	47158896	ANXA8L1
chr10	47158837	47158896	LINC00842
chr10	47161251	47161373	ANXA8L1
chr10	47161251	47161373	LINC00842
chr10	47162122	47162180	ANXA8L1

chr10	47162122	47162180	LINC00842
chr10	47163959	47164054	ANXA8L1
chr10	47163959	47164054	LINC00842
chr10	47164294	47164387	ANXA8L1
chr10	47164294	47164387	LINC00842
chr10	47164934	47164993	ANXA8L1
chr10	47164934	47164993	LINC00842
chr10	47165482	47165561	ANXA8L1
chr10	47165482	47165561	LINC00842
chr10	47166221	47166311	ANXA8L1
chr10	47166221	47166311	LINC00842
chr10	47167070	47167183	ANXA8L1
chr10	47167070	47167183	LINC00842
chr10	47168522	47168730	ANXA8L1
chr10	47168522	47168730	LINC00842
chr10	47169787	47169877	ANXA8L1
chr10	47169787	47169877	LINC00842
chr10	47173898	47173918	ANXA8L1
chr10	47173898	47173918	LINC00842
chr10	47177260	47177393	FAM25B
chr10	47179574	47179636	FAM25B
chr10	47181590	47181662	FAM25B
chr10	47192142	47193533	RP11-144G6.4
chr10	47192142	47193533	AGAP10
chr10	47194179	47194230	AGAP10
chr10	47197538	47197573	AGAP10
chr10	47200161	47200261	AGAP10
chr10	47207812	47207846	AGAP10
chr10	47207812	47207846	RP11-144G6.12
chr10	47210487	47210555	AGAP10
chr10	47210487	47210555	RP11-144G6.12
chr10	47212854	47212922	AGAP10
chr10	47212854	47212922	RP11-144G6.12
chr10	47213292	47213514	AGAP10
chr10	47213292	47213514	RP11-144G6.12
chr10	47219702	47219811	AGAP10
chr10	47219702	47219811	RP11-144G6.12
chr10	47232156	47232295	AGAP10
chr10	47232156	47232295	RP11-144G6.12
chr10	47232156	47232295	BMS1P2
chr10	47235421	47235609	AGAP10
chr10	47235421	47235609	RP11-144G6.12
chr10	47235421	47235609	BMS1P2
chr10	47239293	47239372	AGAP10
chr10	47239293	47239372	RP11-144G6.12
chr10	47239293	47239372	BMS1P2
chr10	47239687	47239738	AGAP10

chr10	47239687	47239738	RP11-144G6.12
chr10	47239687	47239738	BMS1P2
chr10	47769180	47769665	AL603965.1
chr10	47894023	47894049	FAM21B
chr10	47894572	47894634	FAM21B
chr10	47896656	47896829	FAM21B
chr10	47899951	47900044	FAM21B
chr10	47901381	47901442	FAM21B
chr10	47903159	47903206	FAM21B
chr10	47906678	47906788	FAM21B
chr10	47907333	47907420	FAM21B
chr10	47908064	47908135	FAM21B
chr10	47909170	47909288	FAM21B
chr10	47909762	47909819	FAM21B
chr10	47911049	47911108	FAM21B
chr10	47911500	47911679	FAM21B
chr10	47913576	47913703	FAM21B
chr10	47915878	47915964	FAM21B
chr10	47919942	47920043	FAM21B
chr10	47922235	47922366	FAM21B
chr10	47926003	47926172	FAM21B
chr10	47929795	47929897	FAM21B
chr10	47933807	47933959	FAM21B
chr10	47935488	47935670	FAM21B
chr10	47941018	47941146	FAM21B
chr10	47941274	47941354	FAM21B
chr10	47941962	47942084	FAM21B
chr10	47943065	47943127	FAM21B
chr10	47943488	47943700	FAM21B
chr10	47945324	47945944	FAM21B
chr10	47946809	47946986	FAM21B
chr10	47948714	47948853	FAM21B
chr10	48001784	48001973	ASAH2C
chr10	48003968	48004056	ASAH2C
chr10	48007124	48007183	ASAH2C
chr10	48011243	48011389	ASAH2C
chr10	48013519	48013614	ASAH2C
chr10	48017297	48017392	ASAH2C
chr10	48018108	48018147	ASAH2C
chr10	48019329	48019423	ASAH2C
chr10	48020450	48020565	ASAH2C
chr10	48025300	48025381	ASAH2C
chr10	48025507	48025611	ASAH2C
chr10	48027449	48027535	ASAH2C
chr10	48029220	48029345	ASAH2C
chr10	48032985	48033105	ASAH2C
chr10	48047653	48047730	ASAH2C

chr10	48048894	48049021	ASAH2C
chr10	48050357	48050538	ASAH2C
chr10	48054983	48055018	ASAH2C
chr10	48189612	48189663	BMS1P6
chr10	48189612	48189663	AGAP9
chr10	48189978	48190057	BMS1P6
chr10	48189978	48190057	AGAP9
chr10	48193742	48193930	BMS1P6
chr10	48193742	48193930	AGAP9
chr10	48197056	48197195	BMS1P6
chr10	48197056	48197195	AGAP9
chr10	48215836	48216058	AGAP9
chr10	48221505	48221539	AGAP9
chr10	48784175	48784266	PTPN20B
chr10	48791430	48791521	PTPN20B
chr10	50009550	50009553	WDFY4
chr10	50010783	50010831	WDFY4
chr10	50386233	50386338	C10orf128
chr10	50599882	50599907	DRGX
chr10	51187950	51188558	FAM21D
chr10	51189423	51189600	FAM21D
chr10	51191329	51191468	FAM21D
chr10	51224990	51226465	AGAP8
chr10	51227102	51227153	AGAP8
chr10	51230459	51230494	AGAP8
chr10	51233081	51233181	AGAP8
chr10	51240710	51240744	AGAP8
chr10	51243388	51243456	AGAP8
chr10	51245749	51245817	AGAP8
chr10	51246187	51246409	AGAP8
chr10	51372579	51372702	TIMM23B
chr10	51464464	51465939	AGAP7
chr10	51466576	51466627	AGAP7
chr10	51469923	51469958	AGAP7
chr10	51472552	51472652	AGAP7
chr10	51480493	51480527	AGAP7
chr10	51483174	51483242	AGAP7
chr10	51485979	51486201	AGAP7
chr10	51573300	51573416	NCOA4
chr10	52152783	52153163	AC069547.2
chr10	52152783	52153163	SGMS1
chr10	52384680	52384795	RP11-50E11.3
chr10	52384680	52384795	SGMS1
chr10	53841374	53841417	PRKG1
chr10	56335033	56335041	RP11-257I14.1
chr10	56335033	56335041	PCDH15
chr10	56400831	56400836	RP11-257I14.1

chr10	56400831	56400836	PCDH15
chr10	60966514	60966557	PHYHIPL
chr10	61120786	61120870	FAM13C
chr10	61718043	61718429	C10orf40
chr10	61813435	61813485	ANK3
chr10	61867946	61868044	ANK3
chr10	69587397	69587441	DNAJC12
chr10	69755003	69755049	HERC4
chr10	69755803	69755895	HERC4
chr10	69925240	69925255	MYPN
chr10	70042989	70043047	PBLD
chr10	70230324	70230369	DNA2
chr10	70276874	70277022	SLC25A16
chr10	70481294	70481420	CCAR1
chr10	70665982	70666072	DDX50
chr10	71021814	71021862	HKDC1
chr10	71267310	71267330	TSPAN15
chr10	71444655	71444874	RP11-242G20.1
chr10	71450357	71450485	RP11-242G20.1
chr10	71450826	71450860	RP11-242G20.1
chr10	71620448	71620455	COL13A1
chr10	71690916	71690987	COL13A1
chr10	71914844	71914880	SAR1A
chr10	72207680	72207707	NODAL
chr10	72542145	72542155	TBATA
chr10	72620150	72620194	SGPL1
chr10	73474959	73475017	C10orf105
chr10	73474959	73475017	CDH23
chr10	73517335	73517387	C10orf54
chr10	73517335	73517387	CDH23
chr10	73958395	73958423	ASCC1
chr10	73972024	73972043	ASCC1
chr10	73976579	73976611	ANAPC16
chr10	73976579	73976611	ASCC1
chr10	74139310	74139341	MICU1
chr10	74235620	74235621	MICU1
chr10	74288511	74288516	MICU1
chr10	74472117	74472157	MCU
chr10	74474869	74474984	MCU
chr10	74475612	74475626	MCU
chr10	74890108	74890217	NUDT13
chr10	74890108	74890217	ECD
chr10	75007165	75007262	MRPS16
chr10	75007165	75007262	DNAJC9-AS1
chr10	75007165	75007262	DNAJC9
chr10	75208422	75208448	PPP3CB
chr10	75441093	75441099	AGAP5

chr10	75441093	75441099	RP11-464F9.1
chr10	78755243	78755466	KCNMA1
chr10	78782686	78782694	KCNMA1
chr10	79605190	79605195	DLG5
chr10	79626704	79626866	AL391421.1
chr10	79626704	79626866	DLG5
chr10	79627681	79627895	AL391421.1
chr10	79627681	79627895	DLG5
chr10	81266449	81267541	AL133481.1
chr10	81270285	81270319	AL133481.1
chr10	81270285	81270319	RP11-342M3.2
chr10	81374797	81374798	SFTPA1
chr10	81742331	81742366	SFTPD
chr10	81916165	81916254	ANXA11
chr10	82012483	82013325	AL359195.1
chr10	83648946	83649050	NRG3
chr10	88426551	88426605	OPN4
chr10	88713795	88713880	MMRN2
chr10	89420746	89420784	PAPSS2
chr10	89519512	89519544	ATAD1
chr10	91526024	91526203	KIF20B
chr10	92987094	92987131	PCGF5
chr10	94301055	94301110	IDE
chr10	94318885	94318972	IDE
chr10	94759007	94759095	EXOC6
chr10	95098088	95098099	MYOF
chr10	95098220	95098227	MYOF
chr10	95433514	95433566	FRA10AC1
chr10	96075204	96075221	PLCE1
chr10	96075204	96075221	NOC3L
chr10	96336839	96336850	HELLS
chr10	96367667	96367782	HELLS
chr10	96367667	96367782	RP11-119K6.6
chr10	96961198	96961263	C10orf129
chr10	97712180	97712182	RP11-248J23.6
chr10	97712180	97712182	ENTPD1-AS1
chr10	97744354	97744401	RP11-248J23.6
chr10	97744354	97744401	CC2D2B
chr10	97744354	97744401	RP11-690P14.4
chr10	97744354	97744401	ENTPD1-AS1
chr10	97959239	97959289	BLNK
chr10	97959239	97959289	ZNF518A
chr10	98978996	98979006	ARHGAP19
chr10	98978996	98979006	ARHGAP19-SLIT1
chr10	99018446	99018511	ARHGAP19
chr10	99018446	99018511	ARHGAP19-SLIT1
chr10	99179228	99179252	AL355490.1

chr10	99179228	99179252	RP11-452K12.7
chr10	99628991	99629761	GOLGA7B
chr10	99628991	99629761	CRTAC1
chr10	99628991	99629761	GOLGA7B
chr10	102259884	102259916	SEC31B
chr10	102268218	102268226	SEC31B
chr10	102268218	102268226	NDUFB8
chr10	102268218	102268226	NDUFB8
chr10	102729614	102729661	MRPL43
chr10	102729614	102729661	SEMA4G
chr10	102849294	102849662	TLX1NB
chr10	102882536	102883624	HUG1
chr10	102882536	102883624	TLX1NB
chr10	103590181	103590195	KCNIP2
chr10	104477527	104477537	SFXN2
chr10	104487432	104487438	SFXN2
chr10	104857801	104857845	NT5C2
chr10	105159404	105159418	PDCD11
chr10	105348481	105348696	NEURL1
chr10	105348481	105348696	SH3PXD2A
chr10	106977602	106977603	SORCS3
chr10	111701407	111701427	RP11-451M19.3
chr10	111702418	111702618	RP11-451M19.3
chr10	111706977	111707034	RP11-451M19.3
chr10	111711602	111711723	RP11-451M19.3
chr10	111713514	111713633	RP11-451M19.3
chr10	112660855	112660880	BBIP1
chr10	115332419	115332490	HABP2
chr10	115674684	115675055	AL162407.1
chr10	115674684	115675055	NHLRC2
chr10	116623408	116623456	FAM160B1
chr10	118015146	118015215	GFRA1
chr10	118353828	118353854	PNLIPRP1
chr10	118467552	118467570	HSPA12A
chr10	118643742	118643825	ENO4
chr10	118643742	118643825	KIAA1598
chr10	120081817	120081923	FAM204A
chr10	120453236	120453261	CACUL1
chr10	121340322	121340358	TIAL1
chr10	121417075	121417080	BAG3
chr10	121558822	121558833	INPP5F
chr10	122357823	122358209	C10orf85
chr10	124787050	124787108	ACADSB
chr10	127310182	127310214	TEX36
chr10	127430789	127430808	EDRF1
chr10	127449797	127449800	EDRF1
chr10	128191354	128191403	C10orf90

chr10	128200680	128200685	C10orf90
chr10	131308884	131309525	AL355531.2
chr10	131308884	131309525	MGMT
chr10	133607899	133608375	AL450307.1
chr10	133748398	133748433	PPP2R2D
chr10	134062534	134062612	STK32C
chr10	134646269	134646306	TTC40
chr10	135113965	135114051	TUBGCP2
chr10	135342643	135342661	AL161645.2
chr10	135342643	135342661	CYP2E1
chr10	135342643	135342661	SPRN
chr11	223754	223849	SIRT3
chr11	308755	308802	IFITM2
chr11	320201	320248	RP11-326C3.11
chr11	320201	320248	IFITM3
chr11	409217	409256	SIGIRR
chr11	451351	451353	PTDSS2
chr11	451385	451448	PTDSS2
chr11	499501	499600	RNH1
chr11	651315	651407	DEAF1
chr11	752264	752319	TALDO1
chr11	783680	784063	AP006621.5
chr11	932693	932777	AP2A2
chr11	933561	933597	AP2A2
chr11	940846	940894	AP2A2
chr11	1283248	1283273	MUC5B
chr11	1295812	1296033	AC136297.1
chr11	1295812	1296033	TOLLIP
chr11	1304748	1304827	TOLLIP
chr11	1770245	1770356	IFITM10
chr11	1770245	1770356	RP11-295K3.1
chr11	1890934	1890959	LSP1
chr11	1891437	1891438	LSP1
chr11	1891689	1891795	LSP1
chr11	2330551	2330603	TSPAN32
chr11	2400386	2400436	CD81
chr11	2405880	2405912	CD81
chr11	2406995	2407036	CD81
chr11	2465916	2465938	KCNQ1
chr11	2542709	2542800	KCNQ1
chr11	3012266	3012445	AC131971.1
chr11	3012266	3012445	NAP1L4
chr11	3061787	3061811	CARS-AS1
chr11	3061787	3061811	CARS
chr11	3388074	3388287	ZNF195
chr11	3390051	3390085	ZNF195
chr11	3390716	3390732	ZNF195

chr11	4105903	4105995	STIM1
chr11	4126836	4126921	RRM1
chr11	4880886	4881794	OR51H1P
chr11	4880886	4881794	MMP26
chr11	5423827	5424777	OR51J1
chr11	5423827	5424777	HBG2
chr11	5423827	5424777	HBE1
chr11	5423827	5424777	AC104389.28
chr11	5526581	5526642	HBG2
chr11	5526581	5526642	HBE1
chr11	5526581	5526642	AC104389.28
chr11	5691164	5691177	TRIM5
chr11	5735917	5735957	TRIM22
chr11	5735917	5735957	TRIM5
chr11	5755409	5755431	TRIM22
chr11	5755409	5755431	TRIM5
chr11	6149840	6150706	OR56B3P
chr11	6149840	6150706	RP11-290F24.3
chr11	6173006	6173818	OR52B1P
chr11	6173006	6173818	RP11-290F24.3
chr11	6416355	6416509	APBB1
chr11	7619478	7619534	PPFIBP2
chr11	7674733	7674873	PPFIBP2
chr11	7710895	7710919	OVCH2
chr11	8149766	8149803	RIC3
chr11	8174963	8174970	RIC3
chr11	8716094	8716202	RPL27A
chr11	8716094	8716202	RP11-152H18.3
chr11	8716094	8716202	ST5
chr11	8719471	8719511	RPL27A
chr11	8719471	8719511	ST5
chr11	8778593	8778618	ST5
chr11	8949118	8949255	C11orf16
chr11	9004506	9004545	NRIP3
chr11	9228727	9228755	DENND5A
chr11	9481747	9482010	AC132192.1
chr11	9481747	9482010	ZNF143
chr11	9599418	9599442	WEE1
chr11	9614772	9614839	WEE1
chr11	9724943	9724995	SWAP70
chr11	9748442	9748502	SWAP70
chr11	10553547	10553562	RNF141
chr11	10709290	10709324	MRVI1
chr11	10711812	10711916	MRVI1
chr11	11747060	11747257	AC131935.1
chr11	12474551	12474578	PARVA
chr11	14329813	14329863	RRAS2

chr11	14363356	14363406	RRAS2
chr11	14378280	14378315	RRAS2
chr11	16305430	16305464	SOX6
chr11	17097990	17098094	RPS13
chr11	17339131	17339145	NUCB2
chr11	17410388	17410419	KCNJ11
chr11	17555266	17555334	USH1C
chr11	17660296	17660300	OTOG
chr11	17868241	17868308	SERGEF
chr11	17940273	17940326	SERGEF
chr11	17948305	17948393	SERGEF
chr11	18021109	18021119	SERGEF
chr11	18022314	18022351	RP1-59M18.2
chr11	18022314	18022351	SERGEF
chr11	18023495	18023605	RP1-59M18.2
chr11	18023495	18023605	SERGEF
chr11	18031624	18031686	RP1-59M18.2
chr11	18031624	18031686	SERGEF
chr11	18041196	18041285	RP1-59M18.2
chr11	18041196	18041285	TPH1
chr11	18063911	18063914	TPH1
chr11	18162157	18162159	RP11-113D6.6
chr11	18210442	18210917	AC090099.2
chr11	18210442	18210917	GLTPP1
chr11	18210442	18210917	RP11-113D6.6
chr11	18231215	18231679	RP11-113D6.10
chr11	18420345	18420347	LDHA
chr11	19799895	19799897	NAV2
chr11	27681811	27681819	BDNF
chr11	27681811	27681819	BDNF-AS
chr11	28143689	28143718	METTL15
chr11	28218988	28219096	METTL15
chr11	30526337	30526380	MPPED2
chr11	30904988	30905065	DCDC1
chr11	31395650	31395673	DNAJC24
chr11	31417830	31417853	DNAJC24
chr11	31429657	31429731	DNAJC24
chr11	31443519	31443526	DNAJC24
chr11	31458690	31458701	IMMP1L
chr11	33078134	33078269	TCP11L1
chr11	33102661	33102770	TCP11L1
chr11	33102661	33102770	CSTF3
chr11	33166416	33166519	CSTF3
chr11	33721108	33721166	C11orf91
chr11	33721108	33721166	CD59
chr11	33724264	33724283	CD59
chr11	33784801	33784832	FBXO3

chr11	33902649	33903017	AC132216.1
chr11	33902649	33903017	LMO2
chr11	34999331	34999357	PDHX
chr11	35150091	35150095	AL356215.1
chr11	35150191	35150215	AL356215.1
chr11	35276299	35276355	SLC1A2
chr11	36611934	36611940	RAG2
chr11	36611934	36611940	RAG1
chr11	36692826	36692831	C11orf74
chr11	43391629	43391634	TTC17
chr11	43507966	43508001	TTC17
chr11	43918745	43918890	ALKBH3
chr11	43946892	43947022	RP11-613D13.4
chr11	43946892	43947022	C11orf96
chr11	43947983	43948097	RP11-613D13.4
chr11	43947983	43948097	C11orf96
chr11	43954643	43954696	RP11-613D13.4
chr11	43954643	43954696	C11orf96
chr11	44586032	44586052	CD82
chr11	44606753	44606842	CD82
chr11	44609025	44609106	CD82
chr11	44618514	44618519	CD82
chr11	44924945	44925210	TSPAN18
chr11	44924945	44925210	TP53I11
chr11	44925331	44925441	TSPAN18
chr11	44925331	44925441	TP53I11
chr11	44952413	44952517	TSPAN18
chr11	44952413	44952517	TP53I11
chr11	44968590	44968642	TP53I11
chr11	45291042	45291059	SYT13
chr11	45793136	45793265	CTD-2210P24.4
chr11	45793544	45793776	CTD-2210P24.4
chr11	46385924	46385928	DGKZ
chr11	46781902	46781925	CKAP5
chr11	46969577	46969715	C11orf49
chr11	47013093	47013095	C11orf49
chr11	47025915	47025917	C11orf49
chr11	47044889	47044891	C11orf49
chr11	47158867	47158895	C11orf49
chr11	47732366	47732454	AGBL2
chr11	49079305	49079334	TRIM64C
chr11	49229425	49229453	FOLH1
chr11	57308979	57309030	SMTNL1
chr11	57334483	57334530	UBE2L6
chr11	58321616	58321618	LPXN
chr11	58410604	58410728	GLYAT
chr11	58672918	58673134	AP001652.1

chr11	58672918	58673134	GLYATL1
chr11	58674714	58674868	AP001652.1
chr11	58674714	58674868	GLYATL1
chr11	59946267	59946301	MS4A6A
chr11	60050067	60050212	MS4A4A
chr11	60057522	60057597	MS4A4A
chr11	60103003	60103116	MS4A6E
chr11	60172019	60172063	MS4A14
chr11	60202880	60202885	MS4A5
chr11	60468757	60468804	MS4A8
chr11	60479303	60479314	MS4A8
chr11	60658239	60658262	PRPF19
chr11	60750097	60750164	CD6
chr11	61204233	61204394	RP11-286N22.8
chr11	61204233	61204394	SDHAF2
chr11	61236136	61236186	RP11-286N22.8
chr11	61314110	61314131	SYT7
chr11	61546281	61546354	MYRF
chr11	61546281	61546354	TMEM258
chr11	61564394	61564399	FEN1
chr11	61564394	61564399	FADS2
chr11	61579292	61579450	FADS1
chr11	61579292	61579450	FADS2
chr11	61735576	61736070	AP003733.1
chr11	62350035	62350064	MIR3654
chr11	62350035	62350064	TUT1
chr11	62438712	62438802	C11orf48
chr11	62438712	62438802	C11orf83
chr11	62474151	62474183	HNRNPUL2-BSCL2
chr11	62474151	62474183	BSCL2
chr11	62500590	62500600	TTC9C
chr11	62530014	62530086	POLR2G
chr11	62539578	62539604	TAF6L
chr11	62539578	62539604	TMEM223
chr11	62542067	62542137	TAF6L
chr11	62542067	62542137	TMEM223
chr11	62597981	62597992	STX5
chr11	62599426	62599523	STX5
chr11	62651461	62651496	SLC3A2
chr11	62935400	62935532	SLC22A25
chr11	62935400	62935532	SLC22A10
chr11	63129852	63129855	SLC22A10
chr11	63439108	63439187	ATL3
chr11	63636226	63636381	MARK2
chr11	64217017	64217124	AP003774.4
chr11	64217369	64217644	AP003774.4
chr11	64480175	64480217	NRXN2

chr11	64507912	64507921	RASGRP2
chr11	64810497	64810594	SAC3D1
chr11	64857948	64858008	VPS51
chr11	64874659	64874792	VPS51
chr11	64948239	64948543	AP003068.23
chr11	64948239	64948543	CAPN1
chr11	64948696	64949149	AP003068.23
chr11	64948696	64949149	CAPN1
chr11	65148018	65148103	SLC25A45
chr11	65358665	65358730	AP001362.1
chr11	65358665	65358730	EHBP1L1
chr11	65381481	65381483	MAP3K11
chr11	65624895	65624902	CFL1
chr11	65624895	65624902	MUS81
chr11	65627696	65627803	CFL1
chr11	65627696	65627803	MUS81
chr11	65834877	65835010	RP11-1167A19.2
chr11	65834877	65835010	SF3B2
chr11	65836852	65836957	RP11-1167A19.2
chr11	65888492	65888538	PACS1
chr11	65984977	65985088	PACS1
chr11	66048888	66048926	CNIH2
chr11	66290643	66290646	CTD-3074O7.11
chr11	66290643	66290646	BBS1
chr11	66290643	66290646	ZDHHC24
chr11	66606603	66606678	C11orf80
chr11	66963718	66963993	AP001885.1
chr11	66963718	66963993	KDM2A
chr11	67199824	67199831	RPS6KB2
chr11	67199824	67199831	AP003419.16
chr11	67371785	67372540	C11orf72
chr11	67374770	67374795	NDUFV1
chr11	67374943	67374967	NDUFV1
chr11	67798374	67798428	NDUFS8
chr11	67803095	67803232	NDUFS8
chr11	67814404	67814462	TCIRG1
chr11	67946937	67947004	SUV420H1
chr11	68475256	68475322	MTL5
chr11	69489474	69489534	ORAOV1
chr11	69587254	69587718	AP001888.1
chr11	70187083	70187203	AP000487.6
chr11	70187083	70187203	PPFIA1
chr11	70192021	70192115	AP000487.6
chr11	70192021	70192115	PPFIA1
chr11	70341954	70341959	SHANK2
chr11	70343094	70343113	SHANK2
chr11	70719113	70719227	SHANK2

chr11	70753927	70754197	SHANK2
chr11	70757122	70757134	SHANK2
chr11	70757234	70757255	SHANK2
chr11	70779278	70779316	SHANK2
chr11	70779371	70779383	SHANK2
chr11	70788699	70788710	SHANK2
chr11	70788943	70788948	SHANK2
chr11	70789169	70789189	SHANK2
chr11	70790046	70790056	SHANK2
chr11	70794410	70794462	SHANK2
chr11	70796204	70796208	SHANK2
chr11	70798846	70798972	SHANK2
chr11	71139869	71139898	DHCR7
chr11	71187079	71187159	NADSYN1
chr11	71188393	71188484	NADSYN1
chr11	71279909	71279980	KRTAP5-10
chr11	71279909	71279980	AP000867.14
chr11	71316199	71316636	UNC93B6
chr11	71316199	71316636	AP000867.1
chr11	71576883	71576900	RP11-849H4.2
chr11	71615061	71615111	RP11-849H4.2
chr11	71615061	71615111	OR7E126P
chr11	71673198	71673200	RNF121
chr11	71689132	71689230	RNF121
chr11	71800463	71800497	LRTOMT
chr11	71800463	71800497	LAMTOR1
chr11	71820373	71820387	ANAPC15
chr11	71820373	71820387	LRTOMT
chr11	72368895	72369099	RP11-31L22.3
chr11	72368895	72369099	PDE2A
chr11	72491178	72491279	ARAP1
chr11	72491178	72491279	STARD10
chr11	72491411	72491495	ARAP1
chr11	72491411	72491495	STARD10
chr11	72532244	72532246	ATG16L2
chr11	72534589	72534631	ATG16L2
chr11	72534811	72534842	ATG16L2
chr11	72694874	72694897	FCHSD2
chr11	73362468	73362482	PLEKHB1
chr11	73364415	73364506	PLEKHB1
chr11	73415209	73415260	RAB6A
chr11	73471093	73471140	RAB6A
chr11	73747522	73747529	C2CD3
chr11	73933716	73933778	PPME1
chr11	73998332	73998336	P4HA3
chr11	74349659	74349723	POLD3
chr11	74408646	74408739	CHRDL2

chr11	74674640	74674732	SPCS2
chr11	74980231	74980239	ARRB1
chr11	75014643	75014714	ARRB1
chr11	75150385	75150399	GDPD5
chr11	75848238	75848394	CTD-2011F17.2
chr11	75848238	75848394	UVRAG
chr11	75848432	75848496	CTD-2011F17.2
chr11	75848432	75848496	UVRAG
chr11	76075447	76075476	PRKRIR
chr11	76258072	76258144	C11orf30
chr11	76579164	76579276	ACER3
chr11	76668509	76668537	ACER3
chr11	76668509	76668537	CTD-2547H18.1
chr11	77040340	77040357	PAK1
chr11	77058080	77058117	PAK1
chr11	77184416	77184868	DKFZP434E1119
chr11	77184416	77184868	PAK1
chr11	77333052	77333089	CLNS1A
chr11	77595826	77595879	AAMDC
chr11	77595826	77595879	INTS4
chr11	77818763	77818810	ALG8
chr11	77825750	77825844	ALG8
chr11	78614851	78614878	TENM4
chr11	82611693	82611698	C11orf82
chr11	82611693	82611698	PRCP
chr11	82611824	82611868	C11orf82
chr11	82611824	82611868	PRCP
chr11	82624697	82624788	C11orf82
chr11	82624697	82624788	PRCP
chr11	82624999	82625073	C11orf82
chr11	82624999	82625073	PRCP
chr11	82703357	82703425	RP11-659G9.3
chr11	82703357	82703425	RAB30
chr11	82882325	82882339	PCF11
chr11	82996238	82996254	CCDC90B
chr11	83983335	83983343	DLG2
chr11	85342189	85342230	TMEM126B
chr11	85419097	85419193	SYTL2
chr11	85563928	85564524	AP000974.1
chr11	86037555	86037565	C11orf73
chr11	86168692	86168801	RP11-317J19.1
chr11	86168692	86168801	ME3
chr11	86650062	86650104	PRSS23
chr11	86889499	86889585	TMEM135
chr11	86988195	86988490	TMEM135
chr11	88658960	88658999	GRM5
chr11	89939916	89939978	CHORDC1

chr11	89949716	89949721	CHORDC1
chr11	92292576	92292733	FAT3
chr11	92705627	92705643	MTNR1B
chr11	92717973	92718147	MTNR1B
chr11	93466528	93466563	TAF1D
chr11	93467791	93467826	TAF1D
chr11	93474488	93474661	TAF1D
chr11	93523426	93523509	MED17
chr11	94915322	94915338	SESN3
chr11	94915322	94915338	RP11-712B9.2
chr11	95527385	95527498	CEP57
chr11	99428944	99429065	CNTN5
chr11	100856805	100856808	ARHGAP42
chr11	101826523	101827672	KIAA1377
chr11	102197460	102197582	BIRC3
chr11	102219434	102219450	BIRC2
chr11	102318967	102318997	TMEM123
chr11	102369248	102369478	RP11-315O6.2
chr11	102552612	102552619	RP11-817J15.3
chr11	102554056	102554308	RP11-817J15.3
chr11	102594647	102594676	MMP8
chr11	102707228	102707259	MMP3
chr11	102707228	102707259	WTAPP1
chr11	102950690	102950726	DCUN1D5
chr11	104763624	104763718	CASP12
chr11	104813596	104813700	CASP4
chr11	104896280	104896327	CASP1
chr11	105668452	105668639	GRIA4
chr11	107216190	107216216	CWF19L2
chr11	107462484	107462727	AP000889.3
chr11	107462484	107462727	ELMOD1
chr11	107463047	107463186	AP000889.3
chr11	107463047	107463186	ELMOD1
chr11	107582998	107583019	SLN
chr11	107582998	107583019	AP002353.1
chr11	107595119	107595135	AP002353.1
chr11	107643129	107643398	AP001024.2
chr11	107646957	107646962	AP001024.2
chr11	107647303	107647344	AP001024.2
chr11	107650219	107650267	AP001024.1
chr11	107650292	107650492	AP001024.1
chr11	107652270	107652316	AP001024.1
chr11	107711475	107711477	SLC35F2
chr11	108079166	108079311	NPAT
chr11	108130255	108130282	ATM
chr11	108130255	108130282	AP001925.1
chr11	108136458	108136480	ATM

chr11	108136458	108136480	AP001925.1
chr11	108348763	108348867	KDELC2
chr11	110143636	110143776	RDX
chr11	111322132	111322134	RP11-794P6.2
chr11	111322132	111322134	POU2AF1
chr11	111324134	111324308	RP11-794P6.2
chr11	111324134	111324308	POU2AF1
chr11	111324961	111325082	RP11-794P6.2
chr11	111324961	111325082	POU2AF1
chr11	111325688	111325813	RP11-794P6.2
chr11	111325688	111325813	POU2AF1
chr11	111326482	111326603	RP11-794P6.2
chr11	111327240	111327264	RP11-794P6.2
chr11	111389734	111389754	RP11-794P6.6
chr11	111389734	111389754	C11orf88
chr11	111422058	111422141	LAYN
chr11	111636522	111636590	PPP2R1B
chr11	111668895	111668919	ALG9
chr11	111668895	111668919	ALG9
chr11	111676000	111676039	ALG9
chr11	111676000	111676039	ALG9
chr11	111751483	111751544	FDXACB1
chr11	111751483	111751544	C11orf1
chr11	111957020	111957034	TIMM8B
chr11	111961802	111961804	SDHD
chr11	111961802	111961804	SDHD
chr11	111978269	111978290	SDHD
chr11	111978269	111978290	SDHD
chr11	113067915	113067938	NCAM1
chr11	113114274	113114341	NCAM1
chr11	113200364	113200372	TTC12
chr11	113705999	113706049	USP28
chr11	113992540	113992549	ZBTB16
chr11	114310843	114310868	REXO2
chr11	114310843	114310868	RP11-212D19.4
chr11	114314874	114314934	REXO2
chr11	114578110	114578285	NXPE2
chr11	115316480	115316555	CADM1
chr11	115375601	115375675	CADM1
chr11	116820849	116820972	SIK3
chr11	117015009	117015134	PAFAH1B2
chr11	117078447	117078451	PCSK7
chr11	117857522	117857528	IL10RA
chr11	118211585	118211652	CD3D
chr11	118269753	118269776	RP11-770J1.5
chr11	118269753	118269776	UBE4A
chr11	118305377	118305820	RP11-770J1.4

chr11	118318340	118318402	KMT2A
chr11	118398637	118398665	TTC36
chr11	118398637	118398665	RP11-770J1.3
chr11	118401917	118401949	TMEM25
chr11	118872323	118872386	CCDC84
chr11	118972950	118973081	DPAGT1
chr11	118972950	118973081	C2CD2L
chr11	119984355	119984363	TRIM29
chr11	119992642	119992688	TRIM29
chr11	119994041	119994150	TRIM29
chr11	120171752	120171771	POU2F3
chr11	120382513	120382596	AP002348.1
chr11	120382513	120382596	GRIK4
chr11	120385694	120385732	AP002348.1
chr11	120385694	120385732	GRIK4
chr11	120928311	120928323	TBCEL
chr11	124622477	124622582	ESAM
chr11	125338945	125339052	FEZ1
chr11	125445699	125445827	EI24
chr11	125445699	125445827	STT3A-AS1
chr11	125791995	125792005	RP11-680F20.9
chr11	125791995	125792005	DDX25
chr11	126161320	126161402	TIRAP
chr11	126164364	126164497	TIRAP
chr11	126164364	126164497	RP11-712L6.5
chr11	126228320	126228406	ST3GAL4
chr11	129817951	129818002	PRDM10
chr11	129936836	129936978	AP003041.2
chr11	129936996	129937066	AP003041.2
chr11	129942608	129942692	AP003041.2
chr11	129942608	129942692	APLP2
chr11	129942743	129942759	AP003041.2
chr11	129942743	129942759	APLP2
chr11	129943981	129944012	AP003041.2
chr11	129943981	129944012	APLP2
chr11	129970165	129970167	APLP2
chr11	129971821	129971895	APLP2
chr11	133940067	133940107	JAM3
chr11	134053001	134053048	RP11-700F16.3
chr11	134053001	134053048	NCAPD3
chr11	134225909	134225948	GLB1L2
chr11	134855393	134856643	AP003062.1
chr12	147052	147059	AC026369.1
chr12	147257	147377	AC026369.1
chr12	147257	147377	FAM138D
chr12	347421	347509	SLC6A13
chr12	395329	395373	KDM5A

chr12	496414	496440	KDM5A
chr12	497399	497506	KDM5A
chr12	652315	652371	B4GALNT3
chr12	664456	664596	B4GALNT3
chr12	2910779	2910853	FKBP4
chr12	2910779	2910853	RP4-816N1.7
chr12	2910779	2910853	RP4-816N1.6
chr12	2914334	2914360	FKBP4
chr12	2914334	2914360	RP4-816N1.6
chr12	2996144	2996155	RHNO1
chr12	2996144	2996155	TULP3
chr12	3049188	3049247	TULP3
chr12	3049361	3049383	TULP3
chr12	3126229	3126261	TEAD4
chr12	3315446	3315562	TSPAN9
chr12	3749828	3749945	EFCAB4B
chr12	3916250	3916269	PARP11
chr12	3924210	3924219	PARP11
chr12	4406923	4407116	CCND2
chr12	4537561	4537569	FGF6
chr12	4654385	4654400	RAD51AP1
chr12	4672223	4672316	DYRK4
chr12	4682496	4682522	DYRK4
chr12	4689990	4690037	DYRK4
chr12	4762730	4762844	NDUFA9
chr12	4762730	4762844	RP11-500M8.7
chr12	4877608	4877625	GALNT8
chr12	4959904	4960077	KCNA6
chr12	4959904	4960077	GALNT8
chr12	5849074	5849104	ANO2
chr12	6459125	6459190	SCNN1A
chr12	6486833	6486841	SCNN1A
chr12	6486833	6486841	LTBR
chr12	6660648	6660669	IFFO1
chr12	6765045	6765079	ING4
chr12	6834814	6834834	COPS7A
chr12	6835031	6835039	COPS7A
chr12	6836837	6836890	COPS7A
chr12	7108748	7108791	LPCAT3
chr12	7108748	7108791	C1S
chr12	7284422	7284434	CLSTN3
chr12	7992418	7992516	SLC2A14
chr12	8341068	8341099	ZNF705A
chr12	8341068	8341099	FAM66C
chr12	8801144	8801151	MFAP5
chr12	8850328	8850374	RIMKLB
chr12	8850814	8850893	RIMKLB

chr12	9092467	9092483	PHC1
chr12	9093319	9093499	PHC1
chr12	9093319	9093499	M6PR
chr12	9832786	9832847	CLEC2D
chr12	9846421	9846532	CLEC2D
chr12	10245033	10245118	CLEC1A
chr12	10279639	10279688	CLEC7A
chr12	10572148	10572192	NKG2-E
chr12	10572148	10572192	KLRC3
chr12	10761974	10761982	MAGOHB
chr12	10856122	10856218	YBX3
chr12	11993395	11993417	ETV6
chr12	12044475	12044535	ETV6
chr12	12230911	12230912	BCL2L14
chr12	12268229	12268261	BCL2L14
chr12	12291770	12291889	BCL2L14
chr12	12291770	12291889	LRP6
chr12	12509717	12509739	LOH12CR2
chr12	12879231	12879257	APOLD1
chr12	13136666	13136669	HEBP1
chr12	13136666	13136669	RP11-392P7.6
chr12	13294415	13294419	KIAA1467
chr12	13526138	13526372	C12orf36
chr12	13527769	13527940	C12orf36
chr12	13529154	13529339	C12orf36
chr12	14634542	14634567	ATF7IP
chr12	15290764	15290863	RERG
chr12	15818039	15818040	EPS8
chr12	16064625	16064687	DERA
chr12	16751829	16751901	LMO3
chr12	16751829	16751901	MGST1
chr12	18836531	18836565	PLCZ1
chr12	18838561	18838592	PLCZ1
chr12	18889627	18889682	PLCZ1
chr12	18889627	18889682	RP11-361I14.2
chr12	19459333	19459440	PLEKHA5
chr12	19705787	19705907	AEBP2
chr12	19873567	19873621	AEBP2
chr12	21054834	21054936	SLCO1B3
chr12	21054834	21054936	LST3
chr12	21054834	21054936	SLCO1B7
chr12	21245447	21245473	RP11-125O5.2
chr12	21245447	21245473	SLCO1B7
chr12	21272538	21272544	RP11-125O5.2
chr12	21529257	21529298	IAPP
chr12	21529257	21529298	SLCO1A2
chr12	21594141	21594143	PYROXD1

chr12	22376674	22376707	ST8SIA1
chr12	22489046	22489134	ST8SIA1
chr12	22778748	22778765	ETNK1
chr12	22835215	22835273	ETNK1
chr12	25243750	25243800	LRMP
chr12	25254872	25254903	LRMP
chr12	25381515	25381572	AC087239.1
chr12	25381515	25381572	KRAS
chr12	25384952	25384978	AC087239.1
chr12	25384952	25384978	KRAS
chr12	25390815	25390840	AC087239.1
chr12	25390815	25390840	KRAS
chr12	27071974	27072002	ASUN
chr12	27130987	27131027	RP11-421F16.3
chr12	27130987	27131027	TM7SF3
chr12	27165643	27165670	TM7SF3
chr12	27453487	27453523	STK38L
chr12	27752409	27752412	PPFIBP1
chr12	28122942	28122943	PTHLH
chr12	28336731	28336772	RP11-967K21.1
chr12	28336731	28336772	CCDC91
chr12	28565687	28565728	CCDC91
chr12	28724150	28724174	CCDC91
chr12	28726173	28726245	CCDC91
chr12	28732785	28732800	CCDC91
chr12	29444349	29444363	RP11-996F15.2
chr12	29444349	29444363	FAR2
chr12	29493741	29493746	ERGIC2
chr12	29493741	29493746	FAR2
chr12	29542439	29542786	OVCH1-AS1
chr12	29576131	29576231	OVCH1-AS1
chr12	29576131	29576231	OVCH1
chr12	29639819	29639899	OVCH1-AS1
chr12	29639819	29639899	OVCH1
chr12	29721854	29721917	TMTC1
chr12	30841294	30841390	IPO8
chr12	31079886	31079939	TSPAN11
chr12	31238959	31239146	DDX11
chr12	31449900	31449978	FAM60A
chr12	31477778	31478257	AC024940.1
chr12	31477778	31478257	FAM60A
chr12	31693901	31693965	DENND5B
chr12	31861852	31861944	AMN1
chr12	32777593	32777637	FGD4
chr12	32859624	32859667	DNM1L
chr12	32885472	32885501	DNM1L
chr12	32885472	32885501	YARS2

chr12	33576869	33576944	SYT10
chr12	40077923	40078052	C12orf40
chr12	40694615	40694697	LRRK2
chr12	40805409	40805429	RP11-115F18.1
chr12	40805409	40805429	MUC19
chr12	40951620	40951673	MUC19
chr12	42599677	42599679	YAF2
chr12	42624050	42624093	AC020629.1
chr12	42624050	42624093	YAF2
chr12	42627985	42628075	AC020629.1
chr12	42627985	42628075	YAF2
chr12	42737671	42737706	PPHLN1
chr12	42768440	42768464	PPHLN1
chr12	44197979	44198043	TWF1
chr12	44199327	44199440	TWF1
chr12	45621951	45622018	ANO6
chr12	48413844	48413952	RP1-228P16.4
chr12	48413844	48413952	RP1-228P16.5
chr12	48418603	48418892	RP1-228P16.4
chr12	48418603	48418892	RP1-228P16.5
chr12	48482946	48483076	SENP1
chr12	48519108	48519244	PFKM
chr12	48592180	48592575	DKFZP779L1853
chr12	48759940	48760392	AC024257.1
chr12	48759940	48760392	RP11-370I10.6
chr12	49056367	49056437	KANSL2
chr12	49129871	49129932	LINC00935
chr12	49213419	49213487	CACNB3
chr12	49222445	49222555	CACNB3
chr12	49228903	49228997	DDX23
chr12	49362893	49362927	WNT10B
chr12	49410940	49410963	RP11-386G11.5
chr12	49410940	49410963	PRKAG1
chr12	49503557	49503664	LMBR1L
chr12	49503791	49503877	LMBR1L
chr12	49524039	49524185	TUBA1B
chr12	49524039	49524185	RP11-386G11.10
chr12	49650180	49650233	TUBA1C
chr12	49650180	49650233	RP11-977B10.2
chr12	49651667	49651784	TUBA1C
chr12	49651667	49651784	RP11-977B10.2
chr12	49880033	49880119	SPATS2
chr12	49906645	49906688	SPATS2
chr12	50082228	50082260	FMNL3
chr12	50538808	50538844	RP4-605O3.4
chr12	50538808	50538844	CERS5
chr12	50542335	50542371	RP4-605O3.4

chr12	50542335	50542371	CERS5
chr12	50547650	50547734	RP4-605O3.4
chr12	50547650	50547734	CERS5
chr12	50548019	50548103	RP4-605O3.4
chr12	50548019	50548103	CERS5
chr12	50559723	50559726	CERS5
chr12	50579205	50579231	LIMA1
chr12	50690489	50690512	AC140061.12
chr12	50691374	50691718	AC140061.12
chr12	51181991	51182108	ATF1
chr12	51399436	51399460	SLC11A2
chr12	51399995	51400054	SLC11A2
chr12	51619860	51619875	AC139768.1
chr12	51620045	51620085	AC139768.1
chr12	51645288	51645367	DAZAP2
chr12	51645288	51645367	SMAGP
chr12	51698032	51698049	BIN2
chr12	52203959	52204336	RP11-923I11.3
chr12	52203959	52204336	AC068987.1
chr12	52203959	52204336	SCN8A
chr12	52709412	52709452	KRT83
chr12	52709412	52709452	AC121757.1
chr12	52912237	52912277	KRT5
chr12	52922915	52922980	AC055736.1
chr12	53091960	53092044	KRT77
chr12	53300432	53300619	KRT8
chr12	53416038	53416063	EIF4B
chr12	53416038	53416063	RP11-983P16.4
chr12	53419011	53419027	EIF4B
chr12	53419011	53419027	RP11-983P16.4
chr12	53574351	53574380	CSAD
chr12	53853584	53853727	RP11-793H13.8
chr12	53853584	53853727	PCBP2
chr12	53854115	53854209	RP11-793H13.8
chr12	53854115	53854209	PCBP2
chr12	53868653	53869001	PCBP2
chr12	54067542	54067596	ATP5G2
chr12	54674807	54674878	HNRNPA1
chr12	54674807	54674878	RP11-968A15.8
chr12	54766678	54766815	ZNF385A
chr12	54766678	54766815	RP11-753H16.3
chr12	54766678	54766815	RP11-753H16.5
chr12	54853194	54853237	GTSF1
chr12	54853194	54853237	RP11-753H16.3
chr12	54853194	54853237	RP11-753H16.5
chr12	55039798	55039934	DCD
chr12	55343992	55344174	TESPA1

chr12	56211826	56211912	RP11-762I7.5
chr12	56211826	56211912	ORMDL2
chr12	56379495	56379507	RAB5B
chr12	56498828	56498882	RP11-603J24.9
chr12	56498828	56498882	PA2G4
chr12	56498828	56498882	RP11-603J24.17
chr12	56601091	56601094	RNF41
chr12	56606780	56606861	RNF41
chr12	56665666	56665675	CS
chr12	56679002	56679028	CS
chr12	56933470	56933548	RBMS2
chr12	57615090	57615143	NXPH4
chr12	57644957	57644976	STAC3
chr12	57644957	57644976	R3HDM2
chr12	57644957	57644976	RP11-123K3.4
chr12	57810198	57810536	AC126614.1
chr12	57810198	57810536	R3HDM2
chr12	57848720	57848761	INHBE
chr12	57936644	57936649	DCTN2
chr12	57936729	57936758	DCTN2
chr12	57937658	57937663	DCTN2
chr12	57937919	57937927	DCTN2
chr12	58013319	58013661	AC025165.8
chr12	58013319	58013661	SLC26A10
chr12	58018283	58018389	B4GALNT1
chr12	58018283	58018389	SLC26A10
chr12	58209361	58209475	AVIL
chr12	59197114	59197131	RP11-362K2.2
chr12	59199723	59199841	RP11-362K2.2
chr12	59205704	59205815	RP11-362K2.2
chr12	59206196	59206430	RP11-362K2.2
chr12	59917185	59917236	RP11-272B17.2
chr12	59917484	59917692	RP11-272B17.2
chr12	60127497	60127602	SLC16A7
chr12	60129711	60129748	SLC16A7
chr12	60161907	60162037	SLC16A7
chr12	62114694	62114705	FAM19A2
chr12	62271859	62271860	FAM19A2
chr12	62498525	62498544	FAM19A2
chr12	62652544	62652545	FAM19A2
chr12	62706067	62706090	USP15
chr12	62721442	62721493	USP15
chr12	62722074	62722125	USP15
chr12	62810878	62810982	USP15
chr12	62864481	62864501	MON2
chr12	62883816	62883820	MON2
chr12	62996723	62997118	RP11-631N16.2

chr12	62996723	62997118	C12orf61
chr12	63539144	63539166	AVPR1A
chr12	63984820	63984907	DPY19L2
chr12	64013723	64013773	DPY19L2
chr12	65090189	65090329	AC025262.1
chr12	65090189	65090329	RASSF3
chr12	65174589	65175066	TBC1D30
chr12	65179661	65179825	TBC1D30
chr12	65881784	65881870	MSRB3
chr12	65881784	65881870	RP11-230G5.2
chr12	66235914	66235976	HMGA2
chr12	66261269	66261280	RP11-366L20.2
chr12	66261269	66261280	HMGA2
chr12	66291518	66291555	AC090673.2
chr12	66291518	66291555	HMGA2
chr12	66298638	66298794	AC090673.2
chr12	66298638	66298794	HMGA2
chr12	66317959	66317967	AC090673.2
chr12	66317959	66317967	HMGA2
chr12	68045307	68045438	DYRK2
chr12	68674503	68674535	MDM1
chr12	68725781	68725783	MDM1
chr12	69053060	69053063	RAP1B
chr12	69160402	69160677	SLC35E3
chr12	69186336	69186590	AC124890.1
chr12	69186336	69186590	SLC35E3
chr12	69208383	69208439	MDM2
chr12	69209408	69209500	MDM2
chr12	69236047	69236109	RP11-611O2.5
chr12	69236047	69236109	RP11-611O2.3
chr12	69236047	69236109	CPM
chr12	69236047	69236109	MDM2
chr12	69645866	69645919	CPSF6
chr12	69757957	69758079	YEATS4
chr12	70691539	70691659	CNOT2
chr12	70816487	70816493	KCNMB4
chr12	71066625	71066756	FAHD2P1
chr12	71066625	71066756	PTPRR
chr12	71441430	71441459	CTD-2021H9.3
chr12	71441430	71441459	CTD-2021H9.2
chr12	71509630	71509738	CTD-2021H9.3
chr12	71511850	71511962	CTD-2021H9.3
chr12	71532931	71532942	TSPAN8
chr12	72097757	72097831	TMEM19
chr12	75765230	75765262	CAPS2
chr12	75784102	75784161	CAPS2
chr12	76450354	76450452	NAP1L1

chr12	76454790	76454867	NAP1L1
chr12	76879815	76879817	OSBPL8
chr12	77158569	77158592	ZDHHC17
chr12	77162853	77162888	ZDHHC17
chr12	77207093	77207194	ZDHHC17
chr12	77438039	77438049	E2F7
chr12	77949297	77949313	AC073528.1
chr12	77949297	77949313	RP1-34H18.1
chr12	77950325	77950336	AC073528.1
chr12	77950325	77950336	RP1-34H18.1
chr12	77950556	77950568	AC073528.1
chr12	77950556	77950568	RP1-34H18.1
chr12	78537174	78537197	NAV3
chr12	79978922	79978928	PAWR
chr12	80176351	80176375	PPP1R12A
chr12	80198742	80198758	AC073569.1
chr12	80198742	80198758	PPP1R12A
chr12	80760048	80760133	OTOGL
chr12	80816527	80816554	PTPRQ
chr12	80829905	80830162	PTPRQ
chr12	80855627	80855704	PTPRQ
chr12	80855763	80855833	PTPRQ
chr12	80856197	80856261	PTPRQ
chr12	80856304	80856345	PTPRQ
chr12	80856358	80856439	PTPRQ
chr12	80856508	80856558	PTPRQ
chr12	80858971	80859015	PTPRQ
chr12	80859253	80859315	PTPRQ
chr12	81235830	81235907	LIN7A
chr12	81695964	81695972	RP11-121G22.3
chr12	81695964	81695972	PPFIA2
chr12	81761883	81761945	PPFIA2
chr12	82617460	82617473	CCDC59
chr12	82702030	82702094	CCDC59
chr12	85418430	85418550	TSPAN19
chr12	88429819	88429850	C12orf29
chr12	88566743	88566764	TMTC3
chr12	89853793	89853815	POC1B
chr12	91539502	91539511	DCN
chr12	92380402	92380473	C12orf79
chr12	92381309	92381570	C12orf79
chr12	92382873	92382969	C12orf79
chr12	92386968	92387052	C12orf79
chr12	92387146	92387179	C12orf79
chr12	93143327	93143392	PLEKHG7
chr12	93273125	93273139	EEA1
chr12	93864262	93864347	MRPL42

chr12	94648313	94648316	PLXNC1
chr12	94671640	94671913	RP11-1105G2.3
chr12	94671640	94671913	PLXNC1
chr12	94721986	94722084	CCDC41
chr12	95322026	95322039	NDUFA12
chr12	95366223	95366265	NDUFA12
chr12	95390706	95390752	NDUFA12
chr12	95510614	95510683	FGD6
chr12	95637717	95637785	VEZT
chr12	95652021	95652062	VEZT
chr12	95770990	95771145	RP11-167N24.6
chr12	95774634	95774672	RP11-167N24.6
chr12	95875903	95875986	METAP2
chr12	95878619	95878649	METAP2
chr12	96197041	96197048	RP11-536G4.2
chr12	96197041	96197048	RP11-536G4.1
chr12	96216867	96217047	RP11-536G4.2
chr12	96216867	96217047	RP11-536G4.1
chr12	96313295	96313401	CCDC38
chr12	97038936	97038945	C12orf55
chr12	98880881	98880966	RP11-181C3.1
chr12	98887209	98887262	RP11-181C3.1
chr12	98896361	98896390	RP11-181C3.1
chr12	98897538	98897610	RP11-181C3.1
chr12	99175268	99175276	ANKS1B
chr12	99189417	99189423	ANKS1B
chr12	100522410	100522503	UHRF1BP1L
chr12	100592905	100593008	RP11-175P13.3
chr12	100592905	100593008	ACTR6
chr12	100602550	100602605	ACTR6
chr12	100602550	100602605	DEPDC4
chr12	100713002	100713046	SCYL2
chr12	100720963	100720970	SCYL2
chr12	100893631	100893725	NR1H4
chr12	101796981	101797033	ARL1
chr12	102111015	102111095	CHPT1
chr12	102113268	102113276	CHPT1
chr12	102124299	102124331	SYCP3
chr12	102124299	102124331	CHPT1
chr12	102283866	102283881	DRAM1
chr12	102405591	102405641	DRAM1
chr12	102411559	102411583	CCDC53
chr12	102442173	102442175	CCDC53
chr12	102444034	102444069	CCDC53
chr12	102444334	102444417	CCDC53
chr12	102472989	102472994	NUP37
chr12	102544042	102544089	PARPBP

chr12	103558189	103558215	RP11-328J6.1
chr12	103558189	103558215	RP11-552I14.1
chr12	103558469	103558541	RP11-328J6.1
chr12	103558469	103558541	RP11-552I14.1
chr12	103561816	103561913	RP11-328J6.1
chr12	103561816	103561913	RP11-552I14.1
chr12	103667658	103667661	C12orf42
chr12	103761871	103761884	C12orf42
chr12	104098659	104098661	STAB2
chr12	104179454	104179525	NT5DC3
chr12	104235268	104235412	RP11-650K20.3
chr12	104237953	104237984	RP11-650K20.3
chr12	104237953	104237984	RP11-642P15.1
chr12	104739797	104739814	TXNRD1
chr12	105764412	105764458	C12orf75
chr12	106647856	106647906	CKAP4
chr12	106706071	106706096	TCP11L2
chr12	106893854	106893960	POLR3B
chr12	106893854	106893960	RP11-144F15.1
chr12	106901540	106901588	POLR3B
chr12	106901540	106901588	RP11-144F15.1
chr12	107039689	107039714	RFX4
chr12	107039689	107039714	RP11-144F15.1
chr12	107055959	107055964	RFX4
chr12	107055959	107055964	RP11-144F15.1
chr12	107086844	107086937	RFX4
chr12	107086844	107086937	RP11-144F15.1
chr12	107169102	107169143	RIC8B
chr12	107217205	107217225	RIC8B
chr12	107262079	107262124	RIC8B
chr12	107266810	107266828	RIC8B
chr12	109491843	109491847	USP30
chr12	109542901	109542903	UNG
chr12	109717124	109717234	FOXN4
chr12	109908991	109909182	KCTD10
chr12	109955722	109955772	UBE3B
chr12	110007802	110007899	MMAB
chr12	110390345	110390359	GIT2
chr12	110390345	110390359	TCHP
chr12	110458913	110458964	ANKRD13A
chr12	110654342	110654410	IFT81
chr12	110654513	110654560	IFT81
chr12	110949233	110949324	RAD9B
chr12	111054526	111054533	TCTN1
chr12	111054553	111054653	TCTN1
chr12	111086848	111086933	TCTN1
chr12	111086848	111086933	HVCN1

chr12	111537386	111537440	CUX2
chr12	111920592	111920635	ATXN2
chr12	112035553	112035609	ATXN2
chr12	112213409	112213524	ALDH2
chr12	112213409	112213524	RP11-162P23.2
chr12	112320594	112320605	MAPKAPK5
chr12	112403516	112403563	TMEM116
chr12	112416559	112416591	TMEM116
chr12	112535190	112535245	NAA25
chr12	112577923	112577970	TRAFD1
chr12	112895095	112895130	PTPN11
chr12	113269550	113269658	RPH3A
chr12	113275607	113275614	RPH3A
chr12	113279775	113279778	RPH3A
chr12	113659002	113659041	TPCN1
chr12	113683717	113683868	TPCN1
chr12	113740458	113740505	SLC8B1
chr12	113863469	113863625	SDS
chr12	113863469	113863625	SDSL
chr12	113863955	113864077	SDS
chr12	113863955	113864077	SDSL
chr12	115800959	115800978	RP11-116D17.1
chr12	115801870	115802107	RP11-116D17.1
chr12	117186467	117186509	RNFT2
chr12	117486557	117486615	TESC
chr12	117739821	117739876	NOS1
chr12	118455519	118455620	RFC5
chr12	119651482	119651546	HSPB8
chr12	119658519	119658757	HSPB8
chr12	120137621	120137707	RP1-127H14.3
chr12	120137621	120137707	CIT
chr12	120637849	120637860	RPLP0
chr12	120662446	120662530	PXN
chr12	120892736	120892833	AL021546.6
chr12	120892736	120892833	GATC
chr12	120902615	120902625	SRSF9
chr12	120961688	120961805	COQ5
chr12	120964260	120964265	COQ5
chr12	121409319	121409723	HNF1A-AS1
chr12	121409319	121409723	AC079602.1
chr12	121593039	121593128	P2RX7
chr12	121593154	121593177	P2RX7
chr12	121788863	121789006	ANAPC5
chr12	121954272	121954321	KDM2B
chr12	121962479	121962515	KDM2B
chr12	122348848	122348916	PSMD9
chr12	122348848	122348916	RP11-87C12.2

chr12	122750246	122750272	RP11-512M8.5
chr12	122750246	122750272	VPS33A
chr12	122999263	122999275	RSRC2
chr12	123101954	123101980	KNTC1
chr12	123373067	123373171	VPS37B
chr12	123432023	123432046	ABCB9
chr12	123491972	123491975	PITPNM2
chr12	123637080	123637172	MPHOSPH9
chr12	123710887	123710898	MPHOSPH9
chr12	123874593	123874697	SETD8
chr12	123969675	123969770	RILPL1
chr12	123997905	123997908	RILPL1
chr12	124124504	124124548	GTF2H3
chr12	124392860	124393103	DNAH10
chr12	124813630	124813703	NCOR2
chr12	124873231	124873276	NCOR2
chr12	125323790	125323792	SCARB1
chr12	125324519	125324533	SCARB1
chr12	131356582	131356671	RAN
chr12	131451577	131451610	GPR133
chr12	131487299	131487382	GPR133
chr12	131514221	131514373	AC078925.1
chr12	131514221	131514373	GPR133
chr12	131514416	131514769	AC078925.1
chr12	131514416	131514769	GPR133
chr12	131555398	131555435	GPR133
chr12	131780941	131781585	AC092850.1
chr12	131780941	131781585	RP11-495K9.3
chr12	132588566	132589849	EP400NL
chr12	132593117	132593299	EP400NL
chr12	132594313	132594377	EP400NL
chr12	132599003	132599074	EP400NL
chr12	132630785	132630910	NOC4L
chr12	133279015	133279150	PXMP2
chr12	133279015	133279150	RP13-672B3.2
chr12	133658118	133658199	ZNF140
chr12	133660701	133660792	ZNF140
chr12	133677573	133677580	ZNF140
chr12	133721490	133721552	ZNF10
chr12	133721490	133721552	ZNF268
chr12	133721490	133721552	CTD-2140B24.4
chr12	133761250	133761333	ZNF268
chr12	133761250	133761333	CTD-2140B24.4
chr12	133787351	133787680	AC226150.4
chr13	19759450	19759482	RP11-408E5.4
chr13	19761260	19761466	RP11-408E5.4
chr13	19999869	19999973	TPTE2

chr13	20268779	20268961	AL354808.2
chr13	20268779	20268961	PSPC1
chr13	21978459	21978481	ZDHHC20
chr13	22089673	22089699	MICU2
chr13	24322243	24322294	AL139080.1
chr13	24322243	24322294	MIPEP
chr13	24323858	24323901	AL139080.1
chr13	24323858	24323901	MIPEP
chr13	24334672	24334712	MIPEP
chr13	25591541	25591675	LSP1
chr13	25802678	25802719	MTMR6
chr13	25877065	25877146	NUPL1
chr13	25877240	25877243	NUPL1
chr13	25884098	25884153	NUPL1
chr13	25910351	25910466	NUPL1
chr13	26442061	26442083	AL138815.1
chr13	26442061	26442083	ATP8A2
chr13	26442265	26442273	AL138815.1
chr13	26442265	26442273	ATP8A2
chr13	26445330	26445338	AL138815.1
chr13	26445330	26445338	ATP8A2
chr13	26447960	26447973	AL138815.1
chr13	26447960	26447973	ATP8A2
chr13	26452422	26452679	AL138815.2
chr13	26452422	26452679	AL138815.1
chr13	26452422	26452679	ATP8A2
chr13	26455046	26455095	AL138815.1
chr13	26455046	26455095	ATP8A2
chr13	28519397	28519552	ATP5EP2
chr13	28599540	28599552	FLT3
chr13	32949480	32949543	BRCA2
chr13	32970128	32970229	BRCA2
chr13	36814767	36814803	SOHLH2
chr13	36814767	36814803	CCDC169-SOHLH2
chr13	36814767	36814803	CCDC169
chr13	36939724	36939741	SPG20
chr13	36939724	36939741	SPG20OS
chr13	36939867	36939980	SPG20
chr13	36939867	36939980	SPG20OS
chr13	36942335	36942361	SPG20
chr13	36942335	36942361	SPG20OS
chr13	37541873	37541944	ALG5
chr13	37597168	37597181	SUPT20H
chr13	40230963	40231027	COG6
chr13	40252229	40252281	COG6
chr13	41111138	41111323	AL133318.1
chr13	43895228	43895235	ENOX1

chr13	44430576	44430758	CCDC122
chr13	44964493	44964590	SERP2
chr13	44969920	44970002	SERP2
chr13	46716861	46716935	LCP1
chr13	46844469	46844506	FAM206BP
chr13	46844469	46844506	LRRC63
chr13	46844600	46844757	FAM206BP
chr13	46844600	46844757	LRRC63
chr13	47349747	47349827	ESD
chr13	48829036	48829106	ITM2B
chr13	49840015	49840062	CDADC1
chr13	50007495	50007529	AL136218.1
chr13	50007495	50007529	CAB39L
chr13	50008222	50008359	AL136218.1
chr13	50008222	50008359	CAB39L
chr13	50018122	50018167	AL136218.1
chr13	50018122	50018167	CAB39L
chr13	50020549	50020554	AL136218.1
chr13	50020549	50020554	SETDB2
chr13	50071342	50071353	PHF11
chr13	50099909	50099937	PHF11
chr13	50121285	50121307	RCBTB1
chr13	50275659	50275663	KPNA3
chr13	50656573	50656693	DLEU2
chr13	50656573	50656693	DLEU1
chr13	50678840	50678955	DLEU2
chr13	50678840	50678955	DLEU1
chr13	51958773	51958823	INTS6
chr13	51997200	51997226	INTS6
chr13	61058827	61058889	TDRD3
chr13	64320934	64321323	AL445989.1
chr13	74988682	74989077	AL355390.1
chr13	74992909	74992947	AL355390.1
chr13	88325735	88325829	SLITRK5
chr13	95096671	95096690	DCT
chr13	95921675	95921677	ABCC4
chr13	96283057	96283067	DZIP1
chr13	98094022	98094034	RAP2A
chr13	98606150	98606181	IPO5
chr13	98632165	98632197	IPO5
chr13	98640244	98640260	IPO5
chr13	99041738	99041794	FARP1
chr13	99108718	99108814	STK24
chr13	99498774	99498810	DOCK9
chr13	100529852	100529935	CLYBL
chr13	100537231	100537359	CLYBL
chr13	100945478	100945508	PCCA

chr13	100958030	100958088	PCCA
chr13	101179551	101179598	PCCA
chr13	101318223	101318361	TMTC4
chr13	101833446	101833688	NALCN
chr13	103501468	103501544	BIVM-ERCC5
chr13	103501468	103501544	ERCC5
chr13	106119959	106120017	DAOA-AS1
chr13	106119959	106120017	DAOA
chr13	109538788	109538810	MYO16
chr13	111296497	111296529	CARS2
chr13	111315374	111315397	CARS2
chr13	111341180	111341251	CARS2
chr13	111521643	111522137	LINC00346
chr13	111552978	111553008	ANKRD10
chr13	111917831	111917998	ARHGEF7
chr13	111917999	111918057	ARHGEF7
chr13	111919174	111919234	ARHGEF7
chr13	112278343	112278457	RP11-65D24.2
chr13	112278343	112278457	RP11-65D24.1
chr13	112297493	112297546	RP11-65D24.2
chr13	112324745	112324773	RP11-65D24.2
chr13	113571428	113571459	MCF2L
chr13	113688186	113688372	MCF2L
chr13	113706821	113706877	MCF2L
chr13	113749283	113749384	MCF2L
chr13	113755563	113755589	AL137002.1
chr13	113756399	113756422	AL137002.1
chr13	113756600	113756608	AL137002.1
chr13	113794881	113794903	F10
chr13	114117166	114117206	DCUN1D2
chr13	114549939	114550056	GAS6
chr14	19560718	19560876	POTEG
chr14	19578325	19578373	POTEG
chr14	19995263	19995311	POTEM
chr14	20012787	20012945	POTEM
chr14	20783766	20783777	CCNB1IP1
chr14	20783955	20784009	CCNB1IP1
chr14	20788447	20788502	CCNB1IP1
chr14	20919470	20919611	RP11-203M5.7
chr14	20919470	20919611	OSGEP
chr14	20926491	20926505	TMEM55B
chr14	21485038	21485073	NDRG2
chr14	21501079	21501313	RP11-998D10.1
chr14	21501079	21501313	RNASE13
chr14	21501079	21501313	NDRG2
chr14	21501079	21501313	TPPP2
chr14	21503953	21503982	NDRG2

chr14	21503953	21503982	TPPP2
chr14	21699524	21699527	HNRNPC
chr14	21779073	21779076	RPGRIP1
chr14	21786286	21786360	RPGRIP1
chr14	21840790	21840876	SUPT16H
chr14	21905060	21905155	CHD8
chr14	21950711	21950767	TOX4
chr14	22564327	22564375	TRDV1
chr14	22689792	22689837	TRAV35
chr14	22690088	22690371	TRAV35
chr14	23005092	23005151	TRAJ8
chr14	23069080	23069102	ABHD4
chr14	23286463	23286497	AL135998.1
chr14	23286463	23286497	SLC7A7
chr14	23291720	23291750	AL135998.1
chr14	23291720	23291750	SLC7A7
chr14	23306390	23306495	MMP14
chr14	23306521	23306646	MMP14
chr14	23747269	23747309	HOMEZ
chr14	23780182	23780307	BCL2L2
chr14	23780182	23780307	BCL2L2-PABPN1
chr14	23788944	23789025	BCL2L2-PABPN1
chr14	23978549	23978571	NGDN
chr14	24408734	24408931	DHRS4-AS1
chr14	24408734	24408931	DHRS4-AS1
chr14	24564352	24564431	NRL
chr14	24564352	24564431	PCK2
chr14	24579503	24579627	NRL
chr14	24579503	24579627	PCK2
chr14	24681757	24681817	CHMP4A
chr14	24681757	24681817	AL136419.6
chr14	24681757	24681817	TM9SF1
chr14	24681757	24681817	TM9SF1
chr14	24970802	24970942	RP11-80A15.1
chr14	25287292	25287315	STXBP6
chr14	25347976	25348019	STXBP6
chr14	27018902	27018987	NOVA1
chr14	27065142	27065154	NOVA1
chr14	29242014	29242061	C14orf23
chr14	29242566	29242639	C14orf23
chr14	29247064	29247197	C14orf23
chr14	29247312	29247349	C14orf23
chr14	29261086	29261467	C14orf23
chr14	29282179	29282284	C14orf23
chr14	29282179	29282284	RP11-966I7.3
chr14	30063311	30063375	PRKD1
chr14	30516916	30516948	CTD-2251F13.1

chr14	30516916	30516948	PRKD1
chr14	31059860	31059966	G2E3
chr14	31104001	31104057	SCFD1
chr14	31104128	31104169	SCFD1
chr14	31127289	31127303	SCFD1
chr14	31592775	31592830	HECTD1
chr14	31803607	31803662	HEATR5A
chr14	31803607	31803662	RP11-176H8.1
chr14	31809656	31809682	HEATR5A
chr14	31809656	31809682	RP11-176H8.1
chr14	32020101	32020133	NUBPL
chr14	32031998	32032053	NUBPL
chr14	32123276	32123322	NUBPL
chr14	32414132	32414314	RP11-187E13.1
chr14	32419295	32419351	RP11-187E13.1
chr14	32476113	32476205	RP11-187E13.2
chr14	32487714	32487785	RP11-187E13.2
chr14	34047445	34047459	NPAS3
chr14	34081611	34081700	NPAS3
chr14	34249837	34249841	NPAS3
chr14	34561527	34561529	EGLN3
chr14	35073341	35073474	SNX6
chr14	35098664	35098807	SNX6
chr14	35237907	35237986	BAZ1A
chr14	35286038	35286062	BAZ1A
chr14	35519990	35520074	FAM177A1
chr14	35586280	35586382	PPP2R3C
chr14	36010946	36011010	RALGAPA1
chr14	36299001	36299005	BRMS1L
chr14	36400929	36400977	BRMS1L
chr14	36400929	36400977	RP11-116N8.1
chr14	36788791	36788793	MBIP
chr14	36944052	36944078	RP11-896J10.3
chr14	36944052	36944078	SFTA3
chr14	36946986	36947083	RP11-896J10.3
chr14	36946986	36947083	SFTA3
chr14	36988792	36988817	NKX2-1
chr14	36988792	36988817	NKX2-1-AS1
chr14	38063343	38063381	FOXA1
chr14	38306527	38306697	TTC6
chr14	39591111	39591155	GEMIN2
chr14	45464704	45464706	KLHL28
chr14	45464704	45464706	FAM179B
chr14	45565799	45565819	PRPF39
chr14	45602553	45602642	FKBP3
chr14	45664684	45664788	FANCM
chr14	47528462	47528583	MDGA2

chr14	47528462	47528583	MDGA2
chr14	50068260	50068271	LRR1
chr14	50070270	50070336	LRR1
chr14	50166768	50166875	KLHDC1
chr14	50262951	50263015	NEMF
chr14	50300810	50300836	AL627171.1
chr14	50300810	50300836	NEMF
chr14	50311520	50311552	AL627171.1
chr14	50311520	50311552	NEMF
chr14	50459022	50459043	C14orf182
chr14	50459496	50459591	C14orf182
chr14	50472312	50472517	C14orf182
chr14	50550369	50550727	C14orf183
chr14	50550369	50550727	RP11-58E21.5
chr14	50550369	50550727	RP11-58E21.7
chr14	50551848	50552016	C14orf183
chr14	50551848	50552016	RP11-58E21.5
chr14	50551848	50552016	RP11-58E21.7
chr14	50555839	50555913	C14orf183
chr14	50555839	50555913	RP11-58E21.5
chr14	50555839	50555913	RP11-58E21.7
chr14	50558196	50558493	C14orf183
chr14	50559288	50559361	C14orf183
chr14	50682092	50682150	SOS2
chr14	50781309	50781313	ATP5S
chr14	51097650	51097655	ATL1
chr14	51100871	51100892	SAV1
chr14	51134100	51134218	SAV1
chr14	51197641	51197701	NIN
chr14	52344335	52344375	GNG2
chr14	52383155	52383225	RP11-463J10.2
chr14	52383155	52383225	GNG2
chr14	52384052	52384166	RP11-463J10.2
chr14	52384052	52384166	GNG2
chr14	52436014	52436247	RP11-463J10.3
chr14	52436014	52436247	AL358333.1
chr14	52436014	52436247	GNG2
chr14	52445992	52446060	GNG2
chr14	52929666	52929688	TXNDC16
chr14	53063635	53063700	GPR137C
chr14	53080389	53080436	GPR137C
chr14	53394738	53394762	FERMT2
chr14	53415280	53415298	FERMT2
chr14	54903483	54903503	CNIH1
chr14	54989868	54989963	CGRRF1
chr14	56118005	56118061	KTN1
chr14	56129623	56129667	KTN1

chr14	56168062	56168096	KTN1
chr14	56958251	56958299	TMEM260
chr14	57192620	57192705	RP11-1085N6.3
chr14	57196954	57196990	RP11-1085N6.3
chr14	57672580	57672750	AL391152.1
chr14	57672580	57672750	EXOC5
chr14	58047240	58047257	RP11-409I10.2
chr14	58047240	58047257	SLC35F4
chr14	58673767	58673889	ACTR10
chr14	58673767	58673889	C14orf37
chr14	58697798	58697836	ACTR10
chr14	58697798	58697836	C14orf37
chr14	58755636	58755797	AL132989.1
chr14	58755636	58755797	RP11-349A22.5
chr14	58755636	58755797	C14orf37
chr14	58770256	58770261	ARID4A
chr14	58906788	58906878	KIAA0586
chr14	60520593	60520673	LRRC9
chr14	60520593	60520673	RP11-16B13.1
chr14	60600374	60600382	PCNXL4
chr14	60864549	60864555	RBM8B
chr14	60864549	60864555	C14orf39
chr14	60900029	60900131	C14orf39
chr14	61119519	61119849	SIX1
chr14	61187841	61187862	SIX4
chr14	61448914	61448961	RP11-193F5.1
chr14	61448914	61448961	SLC38A6
chr14	61453770	61453786	SLC38A6
chr14	61793711	61793899	PRKCH
chr14	62037416	62037478	RP11-47I22.3
chr14	62037416	62037478	RP11-47I22.4
chr14	62117680	62117735	RP11-47I22.3
chr14	62117680	62117735	RP11-47I22.4
chr14	62120341	62120497	RP11-47I22.3
chr14	62120341	62120497	RP11-47I22.4
chr14	62546058	62546146	RP11-355I22.5
chr14	62546058	62546146	SYT16
chr14	64423148	64423164	SYNE2
chr14	64674676	64674910	SYNE2
chr14	64674676	64674910	ESR2
chr14	65018352	65018355	PPP1R36
chr14	65028506	65028548	PPP1R36
chr14	65028506	65028548	RP11-973N13.3
chr14	65218929	65219033	SPTB
chr14	65397518	65397616	CHURC1
chr14	65397518	65397616	FNTB
chr14	65397518	65397616	CHURC1-FNTB

chr14	65406902	65406916	GPX2
chr14	65406902	65406916	CHURC1
chr14	65406902	65406916	FNTB
chr14	65406902	65406916	CHURC1-FNTB
chr14	65408225	65408239	GPX2
chr14	65408225	65408239	CHURC1
chr14	65408225	65408239	FNTB
chr14	65408225	65408239	CHURC1-FNTB
chr14	65410017	65410130	CHURC1
chr14	65410017	65410130	FNTB
chr14	65410017	65410130	CHURC1-FNTB
chr14	66424458	66424573	CTD-2014B16.3
chr14	66455288	66455368	CTD-2014B16.3
chr14	66456398	66456461	CTD-2014B16.3
chr14	67017759	67017814	GPHN
chr14	67176075	67176081	GPHN
chr14	67648137	67648287	GPHN
chr14	67665692	67665703	FAM71D
chr14	67668148	67668249	FAM71D
chr14	67691358	67691410	FAM71D
chr14	67803448	67803455	ATP6V1D
chr14	68053085	68053173	PIGH
chr14	68053085	68053173	PLEKHH1
chr14	68331505	68331516	RAD51B
chr14	70037483	70037749	CCDC177
chr14	70826009	70826017	COX16
chr14	70826009	70826017	SYNJ2BP-COX16
chr14	71520014	71520129	PCNX
chr14	72457259	72457642	AC005477.1
chr14	72457259	72457642	RGS6
chr14	73555078	73555087	RBM25
chr14	73626717	73626815	PSEN1
chr14	73675532	73675623	PSEN1
chr14	74341287	74341330	PTGR2
chr14	74341287	74341330	RP5-1021I20.4
chr14	75138718	75138723	AREL1
chr14	75144887	75145032	AREL1
chr14	75158879	75159031	AREL1
chr14	75158879	75159031	AC007956.1
chr14	75165467	75165559	AREL1
chr14	75165467	75165559	AC007956.1
chr14	75473764	75473784	EIF2B2
chr14	75473764	75473784	RP11-950C14.3
chr14	75503372	75503445	MLH3
chr14	75605824	75605874	TMED10
chr14	75705632	75705892	RP11-293M10.1
chr14	75735883	75735972	RP11-293M10.1

chr14	75735883	75735972	RP11-293M10.2
chr14	76098117	76098168	FLVCR2
chr14	76452469	76452513	IFT43
chr14	76720557	76720670	RP11-361H10.3
chr14	76720557	76720670	GPATCH2L
chr14	77276560	77276597	ANGEL1
chr14	77295434	77295473	C14orf166B
chr14	77607313	77607753	ZDHHC22
chr14	77607313	77607753	AC007375.1
chr14	77607313	77607753	RP11-463C8.4
chr14	77607313	77607753	TMEM63C
chr14	77882741	77883115	FKSG61
chr14	77882741	77883115	NOXRED1
chr14	77964582	77964671	ISM2
chr14	78176898	78176923	SLIRP
chr14	78200280	78200294	SLIRP
chr14	78200280	78200294	SNW1
chr14	78227711	78227719	AC008372.1
chr14	78227711	78227719	C14orf178
chr14	78228010	78228033	AC008372.1
chr14	78228010	78228033	C14orf178
chr14	78294197	78294314	ADCK1
chr14	80319882	80319991	NRXN3
chr14	80993600	80993605	CEP128
chr14	81360923	81361021	CEP128
chr14	81676233	81676280	GTF2A1
chr14	85995027	85995202	RP11-497E19.1
chr14	85995027	85995202	RP11-497E19.2
chr14	85995348	85995468	RP11-497E19.1
chr14	85995348	85995468	RP11-497E19.2
chr14	87379867	87379903	RP11-322L20.1
chr14	87380986	87381027	RP11-322L20.1
chr14	87386605	87386678	RP11-322L20.1
chr14	87387809	87387952	RP11-322L20.1
chr14	88447777	88447791	GALC
chr14	88453390	88453400	GALC
chr14	88789990	88789992	KCNK10
chr14	88873027	88873080	SPATA7
chr14	88881018	88881025	SPATA7
chr14	89013990	89013995	PTPN21
chr14	89172174	89172217	EML5
chr14	89297220	89297231	TTC8
chr14	89303252	89303410	TTC8
chr14	89591488	89591570	FOXN3
chr14	89591672	89591784	FOXN3
chr14	89697204	89697211	FOXN3
chr14	89750857	89750866	FOXN3

chr14	89799567	89799603	FOXN3
chr14	89867914	89868009	RP11-33N16.2
chr14	89867914	89868009	FOXN3
chr14	90095459	90095480	RP11-944C7.1
chr14	90095459	90095480	RP11-33N16.3
chr14	90097711	90097910	RP11-944C7.1
chr14	90097711	90097910	RP11-33N16.3
chr14	90302907	90303005	EFCAB11
chr14	90302907	90303005	RP11-33N16.3
chr14	90303360	90303489	EFCAB11
chr14	90303360	90303489	RP11-33N16.3
chr14	90431195	90431211	TDP1
chr14	90447807	90447834	TDP1
chr14	90449458	90449470	TDP1
chr14	91068480	91068544	TTC7B
chr14	91144168	91144189	TTC7B
chr14	91165502	91165567	RP11-661G16.2
chr14	91165502	91165567	TTC7B
chr14	91518667	91518806	RPS6KA5
chr14	91631591	91631601	C14orf159
chr14	91686787	91687110	C14orf159
chr14	91690023	91690046	C14orf159
chr14	91710702	91710843	CTD-2547L24.3
chr14	91710702	91710843	GPR68
chr14	91717109	91717422	CTD-2547L24.3
chr14	91717109	91717422	GPR68
chr14	91932759	91932760	SMEK1
chr14	92040510	92040956	AL133373.1
chr14	92344272	92344386	FBLN5
chr14	92406284	92406294	FBLN5
chr14	92408489	92408524	FBLN5
chr14	92443570	92443592	TRIP11
chr14	92549837	92549847	ATXN3
chr14	93532530	93532703	ITPK1
chr14	93655238	93655282	RP11-371E8.4
chr14	94407974	94408038	RP11-131H24.4
chr14	94407974	94408038	ASB2
chr14	94408546	94408605	RP11-131H24.4
chr14	94408546	94408605	ASB2
chr14	94410235	94410388	RP11-131H24.4
chr14	94410235	94410388	ASB2
chr14	94581664	94581672	IFI27
chr14	95078727	95078784	SERPINA3
chr14	95078727	95078784	RP11-986E7.7
chr14	95688883	95688936	CLMN
chr14	95983344	95983634	RP11-1070N10.3
chr14	96009310	96009610	GLRX5

chr14	96179017	96179059	TCL1A
chr14	96179017	96179059	RP11-164H13.1
chr14	96179091	96179111	TCL1A
chr14	96179091	96179111	RP11-164H13.1
chr14	96556882	96557403	C14orf132
chr14	96742336	96742719	DKFZP434O1614
chr14	97397668	97397727	AL133168.3
chr14	97397668	97397727	VRK1
chr14	98217971	98218219	RP11-204N11.1
chr14	98435619	98435884	C14orf64
chr14	98444314	98444461	C14orf64
chr14	100039057	100039105	CCDC85C
chr14	100046358	100046463	CCDC85C
chr14	100189223	100189282	CYP46A1
chr14	100276012	100276021	EML1
chr14	100352210	100352237	EML1
chr14	100568641	100568657	EVL
chr14	100600990	100600992	EVL
chr14	100831812	100831913	WARS
chr14	100832393	100832404	WARS
chr14	101295066	101295152	AL117190.2
chr14	101295066	101295152	MEG3
chr14	101295370	101295537	AL117190.2
chr14	101295370	101295537	MEG3
chr14	101359265	101359615	AL117190.3
chr14	102196792	102196824	RP11-796G6.2
chr14	102196792	102196824	RP11-1029J19.5
chr14	102197694	102197805	RP11-796G6.2
chr14	102198125	102198313	RP11-796G6.2
chr14	102198573	102198583	RP11-796G6.2
chr14	102263548	102263564	CTD-2017C7.2
chr14	102263548	102263564	PPP2R5C
chr14	102356319	102356349	PPP2R5C
chr14	102380512	102380532	PPP2R5C
chr14	102387430	102387567	PPP2R5C
chr14	102745019	102745031	MOK
chr14	102802972	102802986	ZNF839
chr14	102818857	102818892	CINP
chr14	103411511	103411668	CDC42BPB
chr14	103852711	103852725	MARK3
chr14	103957289	103957444	MARK3
chr14	103995563	103995598	TRMT61A
chr14	104045708	104045812	KLC1
chr14	104045708	104045812	RP11-73M18.2
chr14	104045708	104045812	APOPT1
chr14	104172666	104172712	XRCC3
chr14	104200884	104201000	PPP1R13B

chr14	104230799	104230816	PPP1R13B
chr14	104260914	104261000	PPP1R13B
chr14	104313112	104313117	PPP1R13B
chr14	104710618	104710782	C14orf144
chr14	104710618	104710782	RP11-260M19.2
chr14	104941015	104941062	TMEM179
chr14	105220055	105220225	SIVA1
chr14	105223612	105223649	SIVA1
chr14	105233127	105233166	SIVA1
chr14	105235923	105235963	RP11-982M15.2
chr14	105235923	105235963	AKT1
chr14	105452112	105452215	C14orf79
chr14	105455969	105455993	C14orf79
chr14	105520330	105520502	GPR132
chr14	105935406	105935497	RP11-521B24.5
chr14	105935406	105935497	MTA1
chr14	106303485	106303493	IGHD
chr14	106303778	106303935	IGHD
chr14	106386952	106387117	KIAA0125
chr14	106388058	106388203	KIAA0125
chr14	106388400	106388506	KIAA0125
chr14	106539079	106539383	IGHV1-8
chr14	106539470	106539515	IGHV1-8
chr14	106552285	106552592	IGHV3-9
chr14	106552684	106552729	IGHV3-9
chr14	106805209	106805516	IGHV4-31
chr14	106805599	106805644	IGHV4-31
chr14	107082712	107082728	IGHV4-59
chr15	21004687	21005367	AC012414.1
chr15	22011370	22012050	DKFZP547L112
chr15	22456895	22456919	AC010760.1
chr15	22460344	22460351	AC010760.1
chr15	22744255	22744278	GOLGA6L1
chr15	23058970	23058985	NIPA1
chr15	23059372	23059435	NIPA1
chr15	23255340	23255387	GOLGA8I
chr15	23257058	23257177	GOLGA8I
chr15	23258081	23258140	GOLGA8I
chr15	23258322	23258402	GOLGA8I
chr15	23259256	23259294	GOLGA8I
chr15	23259496	23259543	GOLGA8I
chr15	23259632	23259716	GOLGA8I
chr15	23259812	23259921	GOLGA8I
chr15	23260811	23260897	GOLGA8I
chr15	23261004	23261111	GOLGA8I
chr15	23261307	23261394	GOLGA8I
chr15	23261763	23262019	GOLGA8I

chr15	23262275	23262343	GOLGA8I
chr15	23263592	23263667	GOLGA8I
chr15	23264099	23264190	GOLGA8I
chr15	23264765	23264865	GOLGA8I
chr15	23264948	23265045	GOLGA8I
chr15	23265141	23265296	GOLGA8I
chr15	23265381	23265556	GOLGA8I
chr15	26861168	26861207	GABRB3
chr15	26887652	26887675	GABRB3
chr15	27516689	27516784	GABRG3
chr15	27595273	27595316	GABRG3
chr15	27775760	27775858	RP11-100M12.3
chr15	27775760	27775858	GABRG3
chr15	28543720	28543775	HERC2
chr15	29995564	29995638	TJP1
chr15	31233442	31233456	FAN1
chr15	31233442	31233456	MTMR10
chr15	31258236	31258243	MTMR10
chr15	31941882	31942032	OTUD7A
chr15	32746935	32746943	GOLGA8O
chr15	34435091	34435225	KATNBL1
chr15	35271774	35271977	AC114546.1
chr15	35271774	35271977	ZNF770
chr15	37048305	37048307	C15orf41
chr15	37101105	37101136	C15orf41
chr15	37101105	37101136	CSNK1A1P1
chr15	37358623	37358738	MEIS2
chr15	37387208	37387222	MEIS2
chr15	38248873	38248891	TMCO5A
chr15	40458560	40458671	BUB1B
chr15	40532800	40532882	PAK6
chr15	40532800	40532882	RP11-133K1.2
chr15	40545379	40545490	PAK6
chr15	40545379	40545490	RP11-133K1.2
chr15	40556108	40556120	PAK6
chr15	40556108	40556120	RP11-133K1.2
chr15	40556644	40556655	PAK6
chr15	40556644	40556655	RP11-133K1.2
chr15	40576712	40576732	PLCB2
chr15	40662813	40662848	DISP2
chr15	40676658	40676695	KNSTRN
chr15	40681367	40681492	KNSTRN
chr15	40726053	40726113	IVD
chr15	41136265	41136384	RP11-532F12.5
chr15	41136265	41136384	SPINT1
chr15	41315467	41315544	RP11-540O11.4
chr15	41315467	41315544	INO80

chr15	41564378	41564380	CHP1
chr15	41637684	41637752	NUSAP1
chr15	41658014	41658023	NUSAP1
chr15	41785663	41785692	ITPKA
chr15	41866854	41866910	TYRO3
chr15	41976592	41976607	MGA
chr15	42073346	42073408	MAPKBP1
chr15	42073346	42073408	AC073657.1
chr15	42076110	42076229	MAPKBP1
chr15	42076110	42076229	AC073657.1
chr15	42302277	42302445	CTD-2382E5.2
chr15	42302277	42302445	PLA2G4E
chr15	42570223	42570244	GANC
chr15	42707627	42707674	ZNF106
chr15	42966123	42966161	STARD9
chr15	43486279	43486287	EPB42
chr15	43486279	43486287	CCNDBP1
chr15	43677566	43677583	TUBGCP4
chr15	43691310	43691325	TUBGCP4
chr15	43698799	43698814	TUBGCP4
chr15	43704870	43704965	TP53BP1
chr15	43739096	43739112	TP53BP1
chr15	43852611	43852793	PPIP5K1
chr15	43923699	43923736	CATSPER2
chr15	43923699	43923736	STRC
chr15	43927346	43927352	CATSPER2
chr15	43927346	43927352	STRC
chr15	44043782	44043830	PDIA3
chr15	44043850	44043956	PDIA3
chr15	44045000	44045060	PDIA3
chr15	44069314	44069340	ELL3
chr15	44069314	44069340	RP11-296A16.1
chr15	44069314	44069340	SERF2
chr15	44075041	44075058	AC018512.1
chr15	44075041	44075058	RP11-296A16.1
chr15	44075041	44075058	SERF2
chr15	44075202	44075231	AC018512.1
chr15	44075202	44075231	RP11-296A16.1
chr15	44075202	44075231	SERF2
chr15	44880823	44880836	SPG11
chr15	44896322	44896395	SPG11
chr15	45407564	45407782	DUOXA2
chr15	45492925	45493069	SHF
chr15	45544432	45544476	CTD-2651B20.3
chr15	45544432	45544476	SLC28A2
chr15	45665527	45665529	GATM
chr15	45699227	45699238	SPATA5L1

chr15	47425850	47426224	FKSG62
chr15	48483861	48484050	RP11-605F22.2
chr15	48483861	48484050	CTXN2
chr15	48483861	48484050	SLC12A1
chr15	48624909	48624934	DUT
chr15	49009314	49009366	CEP152
chr15	49280784	49280818	SECISBP2L
chr15	49411335	49411364	COPS2
chr15	49453937	49454058	GALK2
chr15	49487282	49487399	GALK2
chr15	49517402	49517441	GALK2
chr15	49527822	49527828	GALK2
chr15	49611169	49611212	GALK2
chr15	49688172	49688294	FAM227B
chr15	49688441	49688639	FAM227B
chr15	49918941	49919006	DTWD1
chr15	49925717	49925809	DTWD1
chr15	50200242	50200290	ATP8B4
chr15	50214582	50214602	ATP8B4
chr15	50301859	50301873	ATP8B4
chr15	50311167	50311173	ATP8B4
chr15	50574490	50574588	GABPB1
chr15	50824295	50824424	USP50
chr15	50844797	50844894	TRPM7
chr15	52420889	52420892	GNB5
chr15	52420889	52420892	CTD-2184D3.7
chr15	52570041	52570224	MYO5C
chr15	52727841	52727867	MYO5A
chr15	53080683	53080711	ONECUT1
chr15	54336622	54336644	UNC13C
chr15	54804546	54804575	UNC13C
chr15	55488551	55488580	RSL24D1
chr15	55666353	55666448	CCPG1
chr15	55666353	55666448	DYX1C1-CCPG1
chr15	56197453	56197479	NEDD4
chr15	56379666	56379707	RFX7
chr15	56380162	56380188	RFX7
chr15	56946944	56947040	ZNF280D
chr15	57891872	57891901	GCOM1
chr15	57891872	57891901	MYZAP
chr15	57891872	57891901	POLR2M
chr15	57938335	57938388	GCOM1
chr15	57938335	57938388	MYZAP
chr15	57938335	57938388	POLR2M
chr15	57984097	57984138	GCOM1
chr15	57984097	57984138	POLR2M
chr15	57998132	57998252	GCOM1

chr15	57998132	57998252	POLR2M
chr15	58027665	58027793	POLR2M
chr15	58968476	58968492	ADAM10
chr15	58990479	58990603	ADAM10
chr15	59088276	59088365	FAM63B
chr15	59213764	59213783	SLTM
chr15	59213764	59213783	RNF111
chr15	59428884	59428976	MYO1E
chr15	59439899	59440054	C15ORF31
chr15	59439899	59440054	MYO1E
chr15	59971005	59971033	BNIP2
chr15	60666651	60666704	ANXA2
chr15	60666820	60666881	ANXA2
chr15	60685237	60685309	ANXA2
chr15	60771202	60771206	NARG2
chr15	60969355	60969431	RORA
chr15	62930726	62930817	RP11-625H11.1
chr15	62930726	62930817	TLN2
chr15	62932359	62932561	RP11-625H11.1
chr15	62932359	62932561	TLN2
chr15	62936716	62936765	RP11-625H11.1
chr15	62936716	62936765	TLN2
chr15	63335668	63335676	TPM1
chr15	63515241	63515260	RAB8B
chr15	63516047	63516069	RAB8B
chr15	63570490	63570553	APH1B
chr15	63822697	63822789	USP3
chr15	63826002	63826017	USP3
chr15	63834253	63834255	USP3
chr15	63929299	63929333	HERC1
chr15	64283427	64283550	DAPK2
chr15	64382429	64382514	FAM96A
chr15	64407788	64407867	SNX1
chr15	64434867	64434870	SNX1
chr15	64437825	64437902	SNX1
chr15	64481605	64481679	CSNK1G1
chr15	64481605	64481679	CTD-2116N17.1
chr15	64557965	64558064	CSNK1G1
chr15	64557965	64558064	CTD-2116N17.1
chr15	64742864	64742961	TRIP4
chr15	64983107	64983208	OAZ2
chr15	65540552	65540566	PARP16
chr15	65748156	65748179	DPP8
chr15	66874554	66874586	RP11-321F6.1
chr15	66956700	66956790	RP11-321F6.1
chr15	66960679	66960792	RP11-321F6.1
chr15	66976337	66976380	RP11-321F6.1

chr15	66977608	66977667	RP11-321F6.1
chr15	67009347	67009411	SMAD6
chr15	67435106	67435294	RP11-342M21.2
chr15	67435106	67435294	SMAD3
chr15	67479358	67479468	SMAD3
chr15	67933783	67933785	MAP2K5
chr15	68380180	68380193	PIAS1
chr15	69719709	69719813	KIF23
chr15	70359222	70359278	TLE3
chr15	70995480	70995542	UACA
chr15	71144557	71144671	LARP6
chr15	71186911	71186994	LRRC49
chr15	71192796	71192921	LRRC49
chr15	72546385	72546411	PARP6
chr15	72648259	72648317	HEXA
chr15	72648259	72648317	RP11-106M3.2
chr15	72654794	72654807	HEXA
chr15	72654794	72654807	RP11-106M3.2
chr15	72990333	72990358	BBS4
chr15	72996765	72996822	BBS4
chr15	73858111	73858118	NPTN
chr15	73983055	73983117	CD276
chr15	74283968	74283974	STOML1
chr15	74420766	74421077	RP11-247C2.2
chr15	74420766	74421077	ISLR2
chr15	74421306	74421432	RP11-247C2.2
chr15	74421306	74421432	ISLR2
chr15	74536075	74536084	CCDC33
chr15	74919330	74919391	CLK3
chr15	74927439	74927468	CLK3
chr15	74927439	74927468	EDC3
chr15	75105778	75105820	LMAN1L
chr15	75183320	75183376	MPI
chr15	75655552	75655631	MAN2C1
chr15	75657942	75658039	MAN2C1
chr15	75658303	75658350	MAN2C1
chr15	75805552	75805779	PTPN9
chr15	75917056	75917176	CTD-2026K11.3
chr15	75917056	75917176	SNUPN
chr15	75970841	75971218	CSPG4
chr15	75970841	75971218	AC105020.1
chr15	76030377	76030994	AC019294.1
chr15	76030377	76030994	RP11-24M17.4
chr15	76030377	76030994	DNM1P35
chr15	76226083	76226092	FBXO22
chr15	76251460	76251481	NRG4
chr15	76261559	76261565	NRG4

chr15	76263586	76263589	NRG4
chr15	76298084	76298150	NRG4
chr15	76509929	76510018	C15orf27
chr15	76509929	76510018	ETFA
chr15	76520164	76520238	C15orf27
chr15	76520164	76520238	ETFA
chr15	76520242	76520259	C15orf27
chr15	76520242	76520259	ETFA
chr15	76576706	76576801	ETFA
chr15	76577146	76577268	ETFA
chr15	76602307	76602405	ETFA
chr15	76859648	76859746	SCAPER
chr15	77066709	77066750	SCAPER
chr15	77148185	77148217	SCAPER
chr15	77231067	77231114	RCN2
chr15	77308328	77308336	PSTPIP1
chr15	77926618	77926638	LINGO1
chr15	78276557	78276858	ADAMTS7P3
chr15	78276557	78276858	TBC1D2B
chr15	78358368	78358391	TBC1D2B
chr15	78403031	78403075	CIB2
chr15	78570421	78570432	DNAJA4
chr15	78570421	78570432	WDR61
chr15	78570550	78570643	DNAJA4
chr15	78570550	78570643	WDR61
chr15	78581252	78581259	WDR61
chr15	78831032	78831283	AC027228.1
chr15	78927076	78927081	CHRNB4
chr15	79168859	79168928	MORF4L1
chr15	79756037	79756048	KIAA1024
chr15	80215116	80215508	ST20
chr15	80215116	80215508	ST20-MTHFS
chr15	80215116	80215508	C15orf37
chr15	80215116	80215508	C15ORF37
chr15	80215519	80216118	ST20
chr15	80215519	80216118	ST20-MTHFS
chr15	80215519	80216118	C15orf37
chr15	80215519	80216118	C15ORF37
chr15	80365642	80365672	ZFAND6
chr15	80367630	80367657	ZFAND6
chr15	80403842	80403901	ZFAND6
chr15	80405118	80405196	ZFAND6
chr15	80672443	80672495	RP11-210M15.2
chr15	80690286	80690348	RP11-210M15.2
chr15	80695844	80695898	RP11-210M15.2
chr15	81188634	81188702	RP11-351M8.2
chr15	81188634	81188702	RP11-351M8.1

chr15	81188634	81188702	KIAA1199
chr15	81189595	81189680	RP11-351M8.2
chr15	81189595	81189680	RP11-351M8.1
chr15	81189595	81189680	KIAA1199
chr15	81193296	81193306	RP11-351M8.2
chr15	81193296	81193306	RP11-351M8.1
chr15	81193296	81193306	KIAA1199
chr15	81244434	81244637	MESDC2
chr15	81245348	81245377	MESDC2
chr15	81582245	81582277	IL16
chr15	81584265	81584299	IL16
chr15	82384075	82384281	RP11-597K23.2
chr15	82387823	82387869	RP11-597K23.2
chr15	82635473	82635575	GOLGA6L10
chr15	82636616	82636676	GOLGA6L10
chr15	82636943	82637652	GOLGA6L10
chr15	82638040	82638127	GOLGA6L10
chr15	82638792	82638872	GOLGA6L10
chr15	82639058	82639117	GOLGA6L10
chr15	82639740	82639859	GOLGA6L10
chr15	82641523	82641606	GOLGA6L10
chr15	82722285	82722368	RP13-608F4.8
chr15	82722285	82722368	GOLGA6L9
chr15	82722285	82722368	UBE2Q2P6
chr15	82724031	82724150	RP13-608F4.8
chr15	82724031	82724150	GOLGA6L9
chr15	82724031	82724150	UBE2Q2P6
chr15	82724773	82724832	RP13-608F4.8
chr15	82724773	82724832	GOLGA6L9
chr15	82724773	82724832	UBE2Q2P6
chr15	82725018	82725098	RP13-608F4.8
chr15	82725018	82725098	GOLGA6L9
chr15	82725018	82725098	UBE2Q2P6
chr15	82725763	82725850	RP13-608F4.8
chr15	82725763	82725850	GOLGA6L9
chr15	82725763	82725850	UBE2Q2P6
chr15	82726234	82726802	RP13-608F4.8
chr15	82726234	82726802	GOLGA6L9
chr15	82726234	82726802	UBE2Q2P6
chr15	82727069	82727129	GOLGA6L9
chr15	82727069	82727129	UBE2Q2P6
chr15	82728167	82728269	GOLGA6L9
chr15	82728167	82728269	UBE2Q2P6
chr15	82728479	82728611	GOLGA6L9
chr15	82728479	82728611	UBE2Q2P6
chr15	82821164	82821289	RPS17
chr15	82822330	82822450	RPS17

chr15	82822630	82822779	RPS17
chr15	82823288	82823393	RPS17
chr15	82823756	82823783	RPS17
chr15	82824389	82824540	RPS17
chr15	82824834	82824836	RPS17
chr15	82932454	82932586	GOLGA6L18
chr15	82932796	82932898	GOLGA6L18
chr15	82933939	82933999	GOLGA6L18
chr15	82934266	82935146	GOLGA6L18
chr15	82935531	82935618	GOLGA6L18
chr15	82936283	82936363	GOLGA6L18
chr15	82936549	82936608	GOLGA6L18
chr15	82937231	82937350	GOLGA6L18
chr15	82939014	82939097	GOLGA6L18
chr15	83011415	83011547	GOLGA6L19
chr15	83208099	83208126	RPS17L
chr15	83209693	83209729	RP11-152F13.10
chr15	83209693	83209729	RP11-379H8.1
chr15	83217406	83217521	RP11-152F13.10
chr15	83217406	83217521	RP11-379H8.1
chr15	83217406	83217521	CPEB1
chr15	83230930	83230980	RP11-379H8.1
chr15	83230930	83230980	CPEB1
chr15	83837974	83837983	HDGFRP3
chr15	83861010	83861060	HDGFRP3
chr15	83861010	83861060	RP11-382A20.4
chr15	84278022	84278050	SH3GL3
chr15	85190196	85190254	WDR73
chr15	85222458	85222518	SEC11A
chr15	85633949	85634053	PDE8A
chr15	86098677	86098722	AKAP13
chr15	89174333	89174407	AEN
chr15	89423774	89423841	HAPLN3
chr15	89861244	89861268	POLG
chr15	89999051	89999077	RHCG
chr15	90029721	90029760	RHCG
chr15	90208074	90208172	PLIN1
chr15	90420555	90420656	AP3S2
chr15	90420555	90420656	C15orf38-AP3S2
chr15	90758169	90758196	SEMA4B
chr15	90772486	90772579	SEMA4B
chr15	90892076	90892429	GABARAPL3
chr15	90898583	90898613	ZNF774
chr15	92397526	92397531	SLCO3A1
chr15	93541368	93541373	CHD2
chr15	93541379	93541435	CHD2
chr15	93609381	93609484	RP11-368J22.2

chr15	93609381	93609484	RGMA
chr15	94857956	94858027	MCTP2
chr15	96831280	96831660	AC016251.1
chr15	96831280	96831660	NR2F2-AS1
chr15	96904487	96904900	AC087477.1
chr15	96904487	96904900	RP11-522B15.3
chr15	98286684	98286713	LINC00923
chr15	98301880	98301891	LINC00923
chr15	98417183	98417515	LINC00923
chr15	98462784	98462829	ARRDC4
chr15	99825973	99825988	LRRC28
chr15	99859300	99859335	AC022819.2
chr15	99859300	99859335	LRRC28
chr15	99859629	99859630	AC022819.2
chr15	99859629	99859630	LRRC28
chr15	99859786	99859828	AC022819.2
chr15	99859786	99859828	LRRC28
chr15	100038447	100038578	AC015660.1
chr15	100038447	100038578	MEF2A
chr15	100230850	100230861	MEF2A
chr15	100348376	100348507	CTD-2054N24.2
chr15	100353178	100353279	CTD-2054N24.2
chr15	100846685	100846782	ADAMTS17
chr15	101105454	101105470	LINS
chr15	101418056	101418070	RP11-66B24.8
chr15	101418056	101418070	ALDH1A3
chr15	101607445	101607526	RP11-505E24.2
chr15	101607445	101607526	LRRK1
chr15	101840983	101841074	RP11-299G20.2
chr15	101840983	101841074	PCSK6
chr15	102177062	102177137	TM2D3
chr15	102239618	102239638	TARSL2
chr16	64381	64511	WASH4P
chr16	64652	64720	WASH4P
chr16	65481	65633	WASH4P
chr16	66290	66448	WASH4P
chr16	66537	66738	WASH4P
chr16	66916	67051	WASH4P
chr16	67291	67427	WASH4P
chr16	67604	67750	WASH4P
chr16	67604	67750	Z84812.4
chr16	67957	68068	WASH4P
chr16	67957	68068	Z84812.4
chr16	68186	68243	WASH4P
chr16	68186	68243	Z84812.4
chr16	68602	68789	WASH4P
chr16	68602	68789	Z84812.4

chr16	114331	114435	RHBDF1
chr16	173506	173544	NPRL3
chr16	175000	175072	NPRL3
chr16	310412	310752	ITFG3
chr16	310412	310752	ITFG3
chr16	448208	448251	NME4
chr16	455513	455665	NME4
chr16	455513	455665	DECR2
chr16	604554	604572	CAPN15
chr16	622569	622601	PIGQ
chr16	631199	631341	PIGQ
chr16	646317	646327	RAB40C
chr16	675037	675165	RAB40C
chr16	723934	724102	RHOT2
chr16	787657	787695	NARFL
chr16	790022	790103	NARFL
chr16	831954	832058	MSLNL
chr16	832863	832926	MSLNL
chr16	905631	906055	LMF1
chr16	981693	981793	LA16c-366D3.1
chr16	981693	981793	LMF1
chr16	997400	997401	LMF1
chr16	1014047	1014122	LMF1
chr16	1031252	1031269	RP11-161M6.2
chr16	1031252	1031269	AC009041.2
chr16	1031252	1031269	LMF1
chr16	1031643	1031663	AC009041.2
chr16	1130832	1130833	SSTR5
chr16	1131019	1131037	SSTR5
chr16	1369703	1369704	LA16c-358B7.3
chr16	1369703	1369704	UBE2I
chr16	1407701	1407753	GNPTG
chr16	1433524	1433933	UNKL
chr16	1445645	1445813	UNKL
chr16	1455907	1456081	UNKL
chr16	1458464	1458549	UNKL
chr16	1476120	1476384	C16orf91
chr16	1478413	1478506	C16orf91
chr16	1479202	1479345	C16orf91
chr16	1524154	1524189	CLCN7
chr16	1670639	1670692	CRAMP1L
chr16	1750075	1750226	HN1L
chr16	1752137	1752174	HN1L
chr16	2013742	2013840	RPS2
chr16	2013742	2013840	AC005363.9
chr16	2031634	2031762	AC005606.1
chr16	2031634	2031762	TBL3

chr16	2031634	2031762	NOXO1
chr16	2039802	2039832	AC005606.14
chr16	2039802	2039832	SYNGR3
chr16	2040500	2040673	SYNGR3
chr16	2075482	2075667	SLC9A3R2
chr16	2092098	2092147	NTHL1
chr16	2133191	2133255	TSC2
chr16	2201131	2201217	RAB26
chr16	2588503	2588586	PDPK1
chr16	2588620	2588763	PDPK1
chr16	2818506	2818518	SRRM2
chr16	2888377	2888404	ZG16B
chr16	2993400	2993421	FLYWCH1
chr16	2993400	2993421	LA16c-321D4.2
chr16	3131634	3131908	IL32
chr16	3131634	3131908	RP11-473M20.9
chr16	3197340	3197481	CASP16
chr16	3197804	3197920	CASP16
chr16	3198325	3198399	CASP16
chr16	3198937	3199040	CASP16
chr16	3199696	3199809	CASP16
chr16	3298465	3298562	MEFV
chr16	3615320	3615346	NLRC3
chr16	3739799	3739812	TRAP1
chr16	3760429	3760454	TRAP1
chr16	4318049	4318082	TFAP4
chr16	4400936	4400948	PAM16
chr16	4400936	4400948	CORO7-PAM16
chr16	4447416	4447451	CORO7-PAM16
chr16	4447416	4447451	CORO7
chr16	4465773	4465853	CORO7-PAM16
chr16	4465773	4465853	CORO7
chr16	4466786	4466884	CORO7-PAM16
chr16	4466786	4466884	CORO7
chr16	4481600	4481673	DNAJA3
chr16	4521317	4521450	NMRAL1
chr16	4658885	4659053	UBALD1
chr16	4798301	4798411	C16orf71
chr16	4798301	4798411	RP11-127I20.7
chr16	4798301	4798411	ZNF500
chr16	4893600	4893603	GLYR1
chr16	5078390	5078399	RP11-165E7.1
chr16	5078390	5078399	NAGPA
chr16	5147281	5147354	FAM86A
chr16	8735260	8735302	METTL22
chr16	8816687	8816699	ABAT
chr16	8832300	8832311	ABAT

chr16	8851052	8851089	ABAT
chr16	8896118	8896215	PMM2
chr16	8955499	8955517	CARHSP1
chr16	9023393	9023400	USP7
chr16	9056333	9056369	USP7
chr16	9761094	9761231	RP11-297M9.1
chr16	9770531	9770692	RP11-297M9.1
chr16	10869380	10869404	TVP23A
chr16	10902394	10902463	TVP23A
chr16	10995655	10995692	CIITA
chr16	11454111	11454187	CTD-3088G3.8
chr16	11537275	11537316	CTD-3088G3.8
chr16	11561008	11561010	CTD-3088G3.6
chr16	11561008	11561010	CTD-3088G3.8
chr16	11943203	11943242	RSL1D1
chr16	11944865	11944867	RSL1D1
chr16	12008846	12008876	GSPT1
chr16	12060877	12061000	TNFRSF17
chr16	12060877	12061000	RP11-166B2.1
chr16	12062000	12062021	AC007216.2
chr16	12062000	12062021	RP11-166B2.1
chr16	12062209	12062333	AC007216.2
chr16	12062209	12062333	RP11-166B2.1
chr16	12123470	12123513	SNX29
chr16	12181628	12182042	RP11-276H1.3
chr16	12181628	12182042	SNX29
chr16	12183626	12183795	RP11-276H1.3
chr16	12183626	12183795	SNX29
chr16	12536852	12536896	SNX29
chr16	14037831	14037843	ERCC4
chr16	14263998	14264020	MKL2
chr16	14564842	14565054	AC092291.2
chr16	14564842	14565054	PARN
chr16	14726247	14726337	PARN
chr16	14762890	14762944	BFAR
chr16	15541792	15541796	RP11-1021N1.1
chr16	15541792	15541796	C16orf45
chr16	15626844	15626880	C16orf45
chr16	15681353	15681483	C16orf45
chr16	15694834	15694909	C16orf45
chr16	15694834	15694909	KIAA0430
chr16	15696357	15696599	C16orf45
chr16	15696357	15696599	KIAA0430
chr16	15766547	15766628	NDE1
chr16	16199257	16199290	ABCC1
chr16	16314216	16314245	ABCC6
chr16	16329700	16329720	NOMO3

chr16	16360454	16360512	NOMO3
chr16	18570156	18570176	NOMO2
chr16	18794963	18795036	RPS15A
chr16	19164410	19164427	CTD-2349B8.1
chr16	19486080	19486095	TMC5
chr16	19589626	19589680	C16orf62
chr16	19598715	19598720	C16orf62
chr16	19716438	19716623	KNOP1
chr16	19716438	19716623	C16orf62
chr16	19747434	19747481	IQCK
chr16	19868777	19868804	GPRC5B
chr16	19868777	19868804	IQCK
chr16	20082319	20082344	GPR139
chr16	20400109	20400231	PDILT
chr16	20400109	20400231	RP11-429K17.1
chr16	20482138	20482168	ACSM2A
chr16	20499542	20499949	AC137056.1
chr16	20565469	20565499	ACSM2B
chr16	20685419	20685422	ACSM3
chr16	20685419	20685422	ACSM1
chr16	20749560	20749591	THUMPD1
chr16	20749560	20749591	ACSM3
chr16	21255361	21255389	ANKS4B
chr16	21255361	21255389	CRYM
chr16	21279997	21280005	CRYM
chr16	21430811	21430896	NPIPB3
chr16	21430956	21431018	NPIPB3
chr16	21863174	21863176	NPIPB4
chr16	21863236	21863298	NPIPB4
chr16	21863338	21863367	NPIPB4
chr16	21972649	21972667	UQCRC2
chr16	22075659	22075669	C16orf52
chr16	22075723	22075759	C16orf52
chr16	22152524	22152570	VWA3A
chr16	22310304	22310357	POLR3E
chr16	22530459	22530488	NPIPB5
chr16	22530650	22530652	NPIPB5
chr16	22844597	22844600	HS3ST2
chr16	23530908	23530960	GGA2
chr16	23533114	23533121	GGA2
chr16	23564344	23564444	EARS2
chr16	23606903	23606959	NDUFAB1
chr16	23649878	23649913	PALB2
chr16	23668807	23668907	DCTN5
chr16	23720508	23720591	ERN2
chr16	23723317	23723399	ERN2
chr16	23723317	23723399	CTD-2385L22.1

chr16	23880434	23880436	PRKCB
chr16	24163171	24163176	PRKCB
chr16	24789665	24789675	TNRC6A
chr16	24838822	24838849	TNRC6A
chr16	24870508	24870561	SLC5A11
chr16	24983937	24983954	ARHGAP17
chr16	27253134	27253210	NSMCE1
chr16	27300131	27300274	CTD-3203P2.2
chr16	27301566	27301667	CTD-3203P2.2
chr16	27366341	27366354	IL4R
chr16	27375424	27375596	IL4R
chr16	27493832	27493906	GTF3C1
chr16	27861800	27861944	GSG1L
chr16	28491051	28491086	CLN3
chr16	28491051	28491086	CLN3
chr16	28510168	28510242	APOBR
chr16	28599931	28600012	CCDC101
chr16	28903636	28903704	ATP2A1
chr16	28932506	28932534	RABEP2
chr16	29049976	29050041	CTB-134H23.2
chr16	29053523	29053705	CTB-134H23.2
chr16	29057331	29057430	CTB-134H23.2
chr16	29057543	29057738	CTB-134H23.2
chr16	29061823	29061883	CTB-134H23.2
chr16	29061993	29062028	CTB-134H23.2
chr16	29063393	29064047	CTB-134H23.2
chr16	29474014	29474028	SLX1B-SULT1A4
chr16	29474014	29474028	SULT1A4
chr16	29474014	29474028	SNX29P2
chr16	29499373	29499412	RP11-231C14.4
chr16	29499373	29499412	SNX29P2
chr16	29511402	29511404	RP11-231C14.4
chr16	29511402	29511404	SNX29P2
chr16	29511566	29511595	RP11-231C14.4
chr16	29511566	29511595	SNX29P2
chr16	29516794	29516913	RP11-231C14.4
chr16	29516794	29516913	SNX29P2
chr16	29822327	29822474	AC009133.14
chr16	29822327	29822474	AC009133.20
chr16	29822327	29822474	MAZ
chr16	29823290	29823329	AC009133.14
chr16	29823290	29823329	AC009133.20
chr16	29823290	29823329	PRRT2
chr16	29823290	29823329	MAZ
chr16	29917852	29917887	ASPHD1
chr16	29917852	29917887	KCTD13
chr16	30013757	30013810	INO80E

chr16	30038409	30038494	C16orf92
chr16	30038409	30038494	FAM57B
chr16	30038924	30038940	C16orf92
chr16	30038924	30038940	FAM57B
chr16	30105794	30105796	YPEL3
chr16	30194348	30194454	CORO1A
chr16	30213359	30213373	SLX1A-SULT1A3
chr16	30213359	30213373	SULT1A3
chr16	30406451	30406776	1-Sep
chr16	30406451	30406776	ZNF48
chr16	30681440	30681622	FBRS
chr16	30766196	30766206	PHKG2
chr16	30959169	30959203	FBXL19
chr16	31044345	31044397	STX4
chr16	31725688	31725738	ZNF720
chr16	31744832	31744854	ZNF720
chr16	31744921	31744948	ZNF720
chr16	31886473	31886484	ZNF267
chr16	31886524	31886538	ZNF267
chr16	32063311	32063601	RP11-1166P10.6
chr16	32063311	32063601	AC142381.1
chr16	46841922	46842046	C16orf87
chr16	47484331	47484364	RNA5SP424
chr16	47484331	47484364	ITFG1
chr16	47596309	47596327	RP11-177N22.2
chr16	47596309	47596327	PHKB
chr16	47653041	47653070	PHKB
chr16	47709497	47709578	PHKB
chr16	50323132	50323224	ADCY7
chr16	50834731	50834753	CYLD
chr16	50834731	50834753	RP11-327F22.4
chr16	52039536	52039546	C16orf97
chr16	52082667	52082719	C16orf97
chr16	52085725	52085739	C16orf97
chr16	52509457	52509552	TOX3
chr16	53353725	53353730	CHD9
chr16	53355075	53355107	CHD9
chr16	54018850	54018866	FTO
chr16	54279597	54279713	RP11-324D17.1
chr16	54280735	54280873	RP11-324D17.1
chr16	55846316	55846342	CES1
chr16	56488686	56488691	OGFOD1
chr16	56662971	56662987	AC026461.1
chr16	56666114	56666150	AC026461.1
chr16	56666114	56666150	MT1M
chr16	56967207	56967227	HERPUD1
chr16	57109853	57109968	NLRC5

chr16	57159189	57159275	CPNE2
chr16	57495084	57495089	AC009052.12
chr16	57495084	57495089	COQ9
chr16	57497568	57497572	POLR2C
chr16	57708501	57708504	GPR97
chr16	57723713	57723975	GPR97
chr16	57723713	57723975	RP11-405F3.4
chr16	58509516	58509561	NDRG4
chr16	58535877	58535957	NDRG4
chr16	58561995	58562009	CNOT1
chr16	58709218	58709246	SLC38A7
chr16	59772016	59772068	RP11-105C20.2
chr16	59772685	59772756	RP11-105C20.2
chr16	59773754	59773837	RP11-105C20.2
chr16	59785865	59785871	RP11-105C20.2
chr16	66542660	66542674	RP11-403P17.5
chr16	66542660	66542674	TK2
chr16	66605040	66605089	CKLF-CMTM1
chr16	66605040	66605089	CMTM1
chr16	66757278	66757330	DYNC1LI2
chr16	66757278	66757330	RP11-63M22.2
chr16	66861363	66861467	NAE1
chr16	66920450	66920453	PDP2
chr16	67198344	67198473	FBXL8
chr16	67198344	67198473	HSF4
chr16	67198344	67198473	RP11-5A19.5
chr16	67370544	67370633	LRRC36
chr16	67396087	67396208	LRRC36
chr16	67479823	67479868	ATP6V0D1
chr16	67491507	67491554	ATP6V0D1
chr16	67492869	67493128	ATP6V0D1
chr16	67797353	67797402	RANBP10
chr16	67922570	67922627	NRN1L
chr16	67974709	67974734	LCAT
chr16	67975830	67975855	LCAT
chr16	67975856	67975945	LCAT
chr16	68172414	68172662	NFATC3
chr16	68282472	68282595	PLA2G15
chr16	68321226	68321338	SLC7A6
chr16	68321226	68321338	SLC7A6OS
chr16	68588976	68589103	ZFP90
chr16	69165227	69165311	CHTF8
chr16	69165227	69165311	CIRH1A
chr16	69166387	69166476	CHTF8
chr16	69166387	69166476	CIRH1A
chr16	69166499	69166592	CIRH1A
chr16	69269651	69269658	SNTB2

chr16	69357486	69357606	VPS4A
chr16	69357486	69357606	COG8
chr16	69678700	69678759	NFAT5
chr16	69680420	69680466	NFAT5
chr16	69861797	69861807	WWP2
chr16	70179636	70179651	PDPR
chr16	70239303	70239683	AC009060.1
chr16	70258261	70258641	FKSG63
chr16	70258261	70258641	RP11-296I10.6
chr16	70323591	70323608	DDX19B
chr16	70381778	70381876	RP11-529K1.3
chr16	70381778	70381876	DDX19A
chr16	70510090	70510145	FUK
chr16	70542704	70542727	COG4
chr16	70549920	70549943	COG4
chr16	70584553	70584563	SF3B3
chr16	70899248	70899266	HYDIN
chr16	71100734	71100836	HYDIN
chr16	71109847	71109891	HYDIN
chr16	71167132	71167186	HYDIN
chr16	71678705	71678758	PHLPP2
chr16	71840589	71840631	AP1G1
chr16	71841704	71841726	AP1G1
chr16	71957994	71958104	IST1
chr16	71957994	71958104	RP11-498D10.5
chr16	72047483	72047491	DHODH
chr16	72124035	72124076	TXNL4B
chr16	72198270	72198385	PMFBP1
chr16	74333474	74333531	PSMD7
chr16	74710306	74710323	MLKL
chr16	74775095	74775166	FA2H
chr16	74993487	74993490	WDR59
chr16	75141631	75141760	ZNRF1
chr16	75452916	75453003	CFDP1
chr16	75452916	75453003	RP11-77K12.1
chr16	75565474	75565538	RP11-77K12.7
chr16	75565474	75565538	CHST5
chr16	75631281	75631287	ADAT1
chr16	75631281	75631287	RP11-77K12.8
chr16	75632169	75632229	ADAT1
chr16	75632169	75632229	RP11-77K12.8
chr16	75793948	75794038	TERF2IP
chr16	75794731	75794873	TERF2IP
chr16	77281871	77281962	RP11-538I12.2
chr16	77281871	77281962	RP11-538I12.3
chr16	77281871	77281962	ADAMTS18
chr16	78085855	78085865	RP11-281J9.2

chr16	78085855	78085865	CLEC3A
chr16	79015647	79015739	PIH1
chr16	79015647	79015739	WWOX
chr16	80581655	80581740	RP11-109P11.1
chr16	80581655	80581740	DYNLRB2
chr16	80581655	80581740	RP11-525K10.3
chr16	80582840	80582895	RP11-109P11.1
chr16	80582840	80582895	DYNLRB2
chr16	80582840	80582895	RP11-525K10.3
chr16	80673567	80673633	CDYL2
chr16	81012280	81012312	CMC2
chr16	81025533	81025562	CMC2
chr16	81030551	81030625	CMC2
chr16	81931481	81931514	PLCG2
chr16	82738667	82738825	CDH13
chr16	82752967	82753035	CDH13
chr16	82917790	82917803	CDH13
chr16	83981816	83981854	RP11-505K9.4
chr16	83986867	83986975	OSGIN1
chr16	84192754	84192756	DNAAF1
chr16	84211996	84212012	DNAAF1
chr16	84211996	84212012	TAF1C
chr16	84766074	84766195	USP10
chr16	84766682	84766687	USP10
chr16	84766696	84766711	USP10
chr16	84953246	84953255	CRISPLD2
chr16	85218725	85218969	CTC-786C10.1
chr16	85392948	85393055	RP11-680G10.1
chr16	85838146	85838201	COX4I1
chr16	86581641	86581717	MTHFSD
chr16	87433204	87433251	RP11-178L8.3
chr16	87433204	87433251	MAP1LC3B
chr16	87728869	87729747	AC010536.1
chr16	87728869	87729747	JPH3
chr16	87798672	87798812	KLHDC4
chr16	87798911	87798946	KLHDC4
chr16	88012196	88012298	BANP
chr16	88698080	88698096	ZC3H18
chr16	88716661	88716707	CYBA
chr16	88727194	88727249	MVD
chr16	88762962	88763046	RP5-1142A6.5
chr16	88762962	88763046	RNF166
chr16	88763081	88763117	RP5-1142A6.5
chr16	88763081	88763117	RNF166
chr16	88894366	88894395	AC092384.1
chr16	88894366	88894395	GALNS
chr16	88894528	88894545	AC092384.1

chr16	88894528	88894545	GALNS
chr16	88928034	88928106	TRAPPC2L
chr16	88928034	88928106	PABPN1L
chr16	89188782	89188850	ACSF3
chr16	89379977	89379997	ANKRD11
chr16	89612280	89612366	SPG7
chr16	89615489	89615547	SPG7
chr16	89653964	89653990	CPNE7
chr16	89749194	89749250	CDK10
chr16	89749194	89749250	RP11-368I7.4
chr16	89751709	89752044	CDK10
chr16	89751709	89752044	RP11-368I7.4
chr16	89753857	89753956	CDK10
chr16	89753997	89754119	CDK10
chr16	89829154	89829201	FANCA
chr16	89844765	89844870	FANCA
chr16	89902623	89902648	SPIRE2
chr16	89944869	89944907	TCF25
chr16	89998209	89998307	TUBB3
chr16	89998209	89998307	TUBB3
chr16	89999523	89999524	TUBB3
chr16	89999523	89999524	TUBB3
chr16	89999547	89999644	TUBB3
chr16	89999547	89999644	TUBB3
chr16	90095397	90095750	GAS8
chr16	90095397	90095750	C16orf3
chr16	90136416	90136460	PRDM7
chr16	90160431	90160487	TUBB8P7
chr16	90160750	90160858	TUBB8P7
chr16	90160937	90161047	TUBB8P7
chr16	90161542	90162597	TUBB8P7
chr17	504135	504317	VPS53
chr17	813049	813172	RP11-676J12.7
chr17	813049	813172	NXN
chr17	813934	813989	RP11-676J12.7
chr17	813934	813989	NXN
chr17	914385	914475	ABR
chr17	1387884	1387892	MYO1C
chr17	1418768	1418879	INPP5K
chr17	1439079	1439135	PITPNA
chr17	1462802	1462887	PITPNA
chr17	1506421	1506501	SLC43A2
chr17	1718005	1718089	SMYD4
chr17	2076392	2076444	SMG6
chr17	2310298	2310524	AC006435.1
chr17	2310298	2310524	METTL16
chr17	2317689	2317765	AC006435.1

chr17	2317689	2317765	METTL16
chr17	2318482	2318651	AC006435.1
chr17	2318482	2318651	METTL16
chr17	2378535	2378593	METTL16
chr17	2614856	2614955	CLUH
chr17	3372129	3372180	SPATA22
chr17	3727358	3727386	C17orf85
chr17	3828081	3828095	ATP2A3
chr17	3920003	3920068	ZZEF1
chr17	3974729	3974740	ZZEF1
chr17	4440748	4441093	SPNS2
chr17	4545518	4545558	ALOX15
chr17	4726554	4726595	PLD2
chr17	4762508	4762623	MINK1
chr17	4852239	4852314	ENO3
chr17	4852239	4852314	PFN1
chr17	5014385	5014514	ZNF232
chr17	5042266	5042318	USP6
chr17	5187179	5187364	RABEP1
chr17	5321347	5321436	NUP88
chr17	6297013	6297170	AIPL1
chr17	6527283	6527326	KIAA0753
chr17	6593281	6593352	SLC13A5
chr17	6692641	6692649	TEKT1
chr17	6692641	6692649	FBXO39
chr17	6693020	6693028	TEKT1
chr17	6693020	6693028	FBXO39
chr17	6698095	6698118	TEKT1
chr17	6698095	6698118	FBXO39
chr17	6780107	6780206	ALOX12P2
chr17	6780107	6780206	AC027763.2
chr17	6784368	6784381	ALOX12P2
chr17	6784368	6784381	AC027763.2
chr17	6829480	6829602	ALOX12P2
chr17	6829480	6829602	AC027763.2
chr17	6888780	6888859	RP11-589P10.7
chr17	6888780	6888859	AC027763.2
chr17	6905803	6905939	RP11-589P10.7
chr17	6905803	6905939	ALOX12
chr17	6905803	6905939	AC027763.2
chr17	6915515	6915517	RP11-589P10.7
chr17	6915515	6915517	AC027763.2
chr17	6933135	6933152	BCL6B
chr17	7117120	7117149	DLG4
chr17	7142905	7142950	PHF23
chr17	7147269	7147271	CTD-2545G14.7
chr17	7147269	7147271	CTDNEP1

chr17	7166478	7166495	CLDN7
chr17	7253872	7253918	ACAP1
chr17	7349717	7349791	CHRNB1
chr17	7456904	7456935	TNFSF12
chr17	7456904	7456935	TNFSF12-TNFSF13
chr17	7476718	7476865	EIF4A1
chr17	7476718	7476865	SNORA67
chr17	7476718	7476865	SENP3-EIF4A1
chr17	7477040	7477094	EIF4A1
chr17	7477040	7477094	SNORA67
chr17	7477040	7477094	SENP3-EIF4A1
chr17	7491413	7491418	MPDU1
chr17	7517269	7517283	AC007421.1
chr17	7517269	7517283	FXR2
chr17	7558554	7558574	ATP1B2
chr17	7831628	7831665	KCNAB3
chr17	7831628	7831665	RP11-1099M24.7
chr17	8055655	8055750	PER1
chr17	8055655	8055750	RP11-599B13.6
chr17	8262810	8263223	AC135178.1
chr17	8283998	8284021	RPL26
chr17	8283998	8284021	RP11-849F2.5
chr17	8283998	8284021	RP11-849F2.7
chr17	8347942	8348060	NDEL1
chr17	8364455	8364542	NDEL1
chr17	9086565	9086755	NTN1
chr17	9487499	9487557	WDR16
chr17	9487589	9487688	WDR16
chr17	9737855	9737859	GLP2R
chr17	10049175	10049546	AC000003.2
chr17	10049175	10049546	GAS7
chr17	10602509	10602584	ADPRM
chr17	10602509	10602584	TMEM220
chr17	10627772	10627819	TMEM220
chr17	11515860	11515986	DNAH9
chr17	12613812	12613816	MYOCD
chr17	15419923	15419973	TVP23C-CDRT4
chr17	15419923	15419973	TVP23C
chr17	15531372	15531375	RP11-385D13.1
chr17	15531372	15531375	TRIM16
chr17	15562116	15562190	TRIM16
chr17	15580922	15581035	TRIM16
chr17	15943505	15943520	AC002553.1
chr17	15943505	15943520	TTC19
chr17	15943505	15943520	NCOR1
chr17	16182971	16183073	PIGL
chr17	16227667	16227705	PIGL

chr17	16252629	16252647	CENPV
chr17	16322651	16322722	TRPV2
chr17	17082872	17082891	RP11-45M22.3
chr17	17082872	17082891	MPRIP
chr17	17104772	17104930	PLD6
chr17	17104772	17104930	MPRIP
chr17	17164914	17165006	COPS3
chr17	17207678	17207692	NT5M
chr17	17468565	17468620	PEMT
chr17	17712481	17712483	RAI1
chr17	17714069	17714194	SREBF1
chr17	17714069	17714194	RAI1
chr17	17715069	17715098	SREBF1
chr17	17715287	17715370	SREBF1
chr17	17765672	17765741	TOM1L2
chr17	17898921	17898961	LRRC48
chr17	17898921	17898961	ATPAF2
chr17	17995232	17995236	DRG2
chr17	17998735	17998803	DRG2
chr17	18210849	18210884	TOP3A
chr17	18429608	18430117	FAM106A
chr17	18429608	18430117	CTD-2303H24.2
chr17	18639330	18639333	TRIM16L
chr17	18820745	18820849	PRPSAP2
chr17	18832880	18832993	PRPSAP2
chr17	18933744	18933756	GRAP
chr17	19041448	19041452	AC007952.1
chr17	19041448	19041452	GRAPL
chr17	19260903	19260947	B9D1
chr17	19482057	19482101	SLC47A1
chr17	19482057	19482101	RP11-1113L8.1
chr17	19751605	19751657	ULK2
chr17	19764255	19764368	ULK2
chr17	19871292	19871347	AKAP10
chr17	20360799	20360876	LGALS9B
chr17	21191303	21191403	MAP2K3
chr17	21199398	21199489	MAP2K3
chr17	21730699	21731373	UBBP4
chr17	21731430	21731615	UBBP4
chr17	26638667	26638677	AC061975.10
chr17	26638667	26638677	KRT18P55
chr17	26642194	26642323	AC061975.10
chr17	26642194	26642323	KRT18P55
chr17	26687174	26687176	TMEM199
chr17	26687174	26687176	CTB-96E2.3
chr17	26713627	26713640	SARM1
chr17	26814428	26814433	SLC13A2

chr17	26814428	26814433	RP11-192H23.4
chr17	26879083	26879110	UNC119
chr17	26879083	26879110	RP11-192H23.4
chr17	26909772	26909837	SPAG5
chr17	26909772	26909837	RP11-192H23.4
chr17	27071719	27071725	TRAF4
chr17	27278485	27278622	PIPOX
chr17	27278485	27278622	PHF12
chr17	27451671	27451706	MYO18A
chr17	27464061	27464102	MYO18A
chr17	27467358	27467436	MYO18A
chr17	27895535	27895566	TP53I13
chr17	27895535	27895566	RP11-68I3.2
chr17	27920960	27921047	RP11-68I3.2
chr17	27920960	27921047	GIT1
chr17	27920960	27921047	ANKRD13B
chr17	28165945	28165947	SSH2
chr17	28483558	28483680	NSRP1
chr17	28495832	28495846	NSRP1
chr17	28774580	28774582	CPD
chr17	28793265	28793316	CPD
chr17	28809918	28810011	GOSR1
chr17	28822444	28822459	GOSR1
chr17	29099403	29099455	SUZ12P
chr17	29099403	29099455	CRLF3
chr17	29100425	29100515	SUZ12P
chr17	29100425	29100515	CRLF3
chr17	29584705	29584737	NF1
chr17	29626528	29626624	NF1
chr17	29641389	29641483	CTD-2370N5.3
chr17	29641389	29641483	NF1
chr17	29705906	29705949	NF1
chr17	29836095	29836217	RAB11FIP4
chr17	29898165	29898380	AC003101.1
chr17	29899104	29899193	AC003101.1
chr17	29902530	29902658	MIR365B
chr17	29902530	29902658	AC003101.1
chr17	30334960	30335151	LRRC37B
chr17	30470044	30470048	AC090616.2
chr17	30470044	30470048	RHOT1
chr17	30499956	30500015	RHOT1
chr17	30506379	30506885	AC116407.2
chr17	30506379	30506885	RHOT1
chr17	30534531	30534573	RHOT1
chr17	30615251	30615271	RHBDL3
chr17	30651744	30651793	RP11-227G15.3
chr17	30651744	30651793	C17orf75

chr17	31072671	31072707	MYO1D
chr17	32581900	32581926	AC005549.3
chr17	33352357	33352359	RFFL
chr17	33352357	33352359	RAD51L3-RFFL
chr17	34307444	34307529	CCL16
chr17	34538887	34538895	CCL4L1
chr17	34624259	34624349	CCL3L1
chr17	34624259	34624349	TBC1D3C
chr17	34624259	34624349	TBC1D3H
chr17	34624771	34624885	CCL3L1
chr17	34624771	34624885	TBC1D3C
chr17	34624771	34624885	TBC1D3H
chr17	34625521	34625646	CCL3L1
chr17	34625521	34625646	TBC1D3C
chr17	34625521	34625646	TBC1D3H
chr17	34640110	34640185	CCL4L2
chr17	34640110	34640185	TBC1D3C
chr17	34640110	34640185	TBC1D3H
chr17	34640744	34640858	CCL4L2
chr17	34640744	34640858	TBC1D3C
chr17	34640744	34640858	TBC1D3H
chr17	34641261	34641401	CCL4L2
chr17	34641261	34641401	TBC1D3C
chr17	34641261	34641401	TBC1D3H
chr17	34641450	34641537	CCL4L2
chr17	34641450	34641537	TBC1D3C
chr17	34641450	34641537	TBC1D3H
chr17	35646424	35646430	ACACA
chr17	35995627	35995634	DDX52
chr17	35999545	35999597	DDX52
chr17	36351925	36351996	TBC1D3
chr17	36351925	36351996	RP11-1407O15.2
chr17	36352422	36352526	TBC1D3
chr17	36352422	36352526	RP11-1407O15.2
chr17	36353601	36353765	TBC1D3
chr17	36353601	36353765	RP11-1407O15.2
chr17	36357158	36357272	TBC1D3
chr17	36357158	36357272	RP11-1407O15.2
chr17	36367407	36367522	RP11-1407O15.2
chr17	36412905	36413159	RP11-1407O15.2
chr17	36421735	36421811	RP11-1407O15.2
chr17	36458712	36458833	MRPL45
chr17	36645999	36646015	ARHGAP23
chr17	36884086	36884451	AC006449.1
chr17	36884086	36884451	MLLT6
chr17	36890412	36890506	CISD3
chr17	36890412	36890506	PCGF2

chr17	36905801	36906391	CTB-58E17.5
chr17	36905801	36906391	PCGF2
chr17	36971878	36971879	CWC25
chr17	36971979	36971994	CWC25
chr17	37053155	37053181	LASP1
chr17	37235747	37235781	CTD-2206N4.4
chr17	37235747	37235781	PLXDC1
chr17	37237678	37237702	CTD-2206N4.4
chr17	37237678	37237702	PLXDC1
chr17	37283158	37283257	PLXDC1
chr17	37601715	37601822	MED1
chr17	37869406	37869522	ERBB2
chr17	38152954	38152962	PSMD3
chr17	38154047	38154103	PSMD3
chr17	38212802	38212880	MED24
chr17	38281007	38281114	MSL1
chr17	38287473	38287516	MSL1
chr17	38517235	38517393	CTD-2267D19.3
chr17	38517235	38517393	GJD3
chr17	38796319	38796330	SMARCE1
chr17	38796319	38796330	KRT222
chr17	38800637	38800639	SMARCE1
chr17	38800637	38800639	KRT222
chr17	39120421	39120424	KRT39
chr17	39120421	39120424	AC004231.2
chr17	39344697	39344713	KRTAP9-1
chr17	39673798	39673861	KRT15
chr17	39874197	39874278	HAP1
chr17	39874197	39874278	JUP
chr17	40155533	40155575	DNAJC7
chr17	40170456	40170493	DNAJC7
chr17	40170456	40170493	NKIRAS2
chr17	40173892	40173893	NKIRAS2
chr17	40526077	40526081	STAT3
chr17	40630945	40631067	ATP6V0A1
chr17	40654812	40654886	ATP6V0A1
chr17	40662077	40662106	RP11-400F19.18
chr17	40662077	40662106	ATP6V0A1
chr17	40757303	40757309	FAM134C
chr17	40926266	40926268	VPS25
chr17	41114192	41114312	PTGES3L-AARSD1
chr17	41114192	41114312	AARSD1
chr17	41262552	41262597	BRCA1
chr17	41851342	41851477	DUSP3
chr17	41854897	41854898	DUSP3
chr17	41855169	41855181	DUSP3
chr17	41896440	41896449	MPP3

chr17	41994679	41995023	FAM215A
chr17	41994679	41995023	RP11-527L4.5
chr17	42015731	42015765	RP11-527L4.2
chr17	42015929	42016082	RP11-527L4.2
chr17	42148995	42148997	G6PC3
chr17	42286331	42286369	CTB-175E5.7
chr17	42286331	42286369	UBTF
chr17	42355793	42355825	AC003043.1
chr17	42436471	42436511	FAM171A2
chr17	42544471	42544482	GPATCH8
chr17	42551107	42551127	GPATCH8
chr17	43161026	43161055	NMT1
chr17	43210618	43210711	PLCD3
chr17	43210618	43210711	ACBD4
chr17	43338248	43338357	MAP3K14-AS1
chr17	43338248	43338357	SPATA32
chr17	43902852	43902902	CRHR1
chr17	44046531	44046577	MAPT
chr17	45217322	45217339	CDC27
chr17	45636978	45637053	NPEPPS
chr17	45654411	45654526	NPEPPS
chr17	45664027	45664120	NPEPPS
chr17	45749076	45749228	KPNB1
chr17	45912184	45912255	LRRC46
chr17	46105102	46105155	COPZ2
chr17	46137280	46137333	NFE2L1
chr17	46309233	46309264	SKAP1
chr17	46326336	46326391	SKAP1
chr17	46688855	46688966	HOXB8
chr17	46688855	46688966	HOXB7
chr17	46881657	46881885	TTLL6
chr17	46892717	46892760	TTLL6
chr17	46921265	46921388	CALCOCO2
chr17	46926163	46926189	CALCOCO2
chr17	46933941	46933978	CALCOCO2
chr17	47011857	47011938	AC091133.1
chr17	47011857	47011938	SNF8
chr17	47448194	47448297	RP11-81K2.1
chr17	47450375	47450448	RP11-81K2.1
chr17	47554239	47554297	RP11-81K2.1
chr17	47695908	47695925	SPOP
chr17	47795374	47795406	RP11-613C6.2
chr17	47795374	47795406	FAM117A
chr17	47801756	47801791	FAM117A
chr17	48049541	48049548	DLX4
chr17	48167554	48167682	ITGA3
chr17	48537563	48537599	ACSF2

chr17	48592206	48592310	MYCBPAP
chr17	48593220	48593454	MYCBPAP
chr17	48610236	48610346	EPN3
chr17	48720910	48720996	ABCC3
chr17	48801180	48801326	LUC7L3
chr17	48815502	48815524	LUC7L3
chr17	48826580	48826649	LUC7L3
chr17	48828658	48828682	LUC7L3
chr17	49085198	49085212	SPAG9
chr17	49086492	49086503	SPAG9
chr17	49286768	49286791	MBTD1
chr17	49375143	49375175	UTP18
chr17	49739192	49739206	CA10
chr17	50212239	50212317	CA10
chr17	50212342	50212409	CA10
chr17	50939750	50939831	AC102948.2
chr17	50975794	50975866	AC102948.2
chr17	50976691	50976703	AC102948.2
chr17	52978515	52978542	TOM1L1
chr17	52984140	52984147	TOM1L1
chr17	53019650	53019731	TOM1L1
chr17	53036641	53036680	TOM1L1
chr17	53036641	53036680	COX11
chr17	53864927	53865015	PCTP
chr17	54251682	54251762	ANKFN1
chr17	54989476	54989526	TRIM25
chr17	55028783	55028811	COIL
chr17	55198640	55198679	AKAP1
chr17	55337338	55337367	MSI2
chr17	55509947	55509975	MSI2
chr17	56282912	56283065	MKS1
chr17	56331914	56331942	LPO
chr17	56645262	56645272	TEX14
chr17	56725409	56725473	TEX14
chr17	56783850	56783969	RAD51C
chr17	57150455	57150502	TRIM37
chr17	57201724	57201789	SKA2
chr17	57229130	57229132	SKA2
chr17	57238162	57238237	PRR11
chr17	57736023	57736028	CLTC
chr17	57911373	57911411	VMP1
chr17	57962060	57962117	TUBD1
chr17	57969175	57969177	TUBD1
chr17	57971157	57971174	RPS6KB1
chr17	58036009	58036062	RP11-178C3.1
chr17	58036009	58036062	RNFT1
chr17	58151503	58151520	HEATR6

chr17	58153066	58153194	HEATR6
chr17	58573774	58573802	APPBP2
chr17	58642747	58642800	RP11-15E18.2
chr17	58642747	58642800	RP11-15E18.4
chr17	58654558	58654699	RP11-15E18.4
chr17	58663669	58663748	RP11-15E18.4
chr17	58682067	58682109	PPM1D
chr17	58766293	58766325	BCAS3
chr17	59861247	59861268	BRIP1
chr17	60351451	60351477	TBC1D3P2
chr17	60351451	60351477	RP11-51L5.7
chr17	60360063	60360245	RP11-51L5.5
chr17	60360063	60360245	RP11-51L5.7
chr17	60584742	60584810	TLK2
chr17	60585769	60585777	TLK2
chr17	60778723	60778739	RP11-156L14.1
chr17	60778723	60778739	10-Mar
chr17	60827560	60827897	10-Mar
chr17	61516473	61516567	CYB561
chr17	61745506	61745525	MAP3K3
chr17	62511841	62511905	CEP95
chr17	62516604	62516694	CEP95
chr17	62538192	62538281	CEP95
chr17	62590216	62590222	SMURF2
chr17	62915549	62915571	LRRC37A3
chr17	64550637	64550697	PRKCA
chr17	64692545	64692553	PRKCA
chr17	65027509	65027514	AC005544.1
chr17	65027509	65027514	CACNG4
chr17	65027554	65028198	AC005544.1
chr17	65027554	65028198	CACNG4
chr17	65175712	65175852	HELZ
chr17	65357369	65357377	PSMD12
chr17	65358988	65359008	PSMD12
chr17	65715227	65715283	NOL11
chr17	66244206	66244227	AMZ2
chr17	66244781	66244846	AMZ2
chr17	66524772	66524785	PRKAR1A
chr17	66586442	66586490	FAM20A
chr17	67143579	67143581	ABCA10
chr17	67143662	67143754	ABCA10
chr17	67192500	67192624	ABCA10
chr17	67211681	67211683	ABCA10
chr17	67281551	67281561	ABCA5
chr17	70036465	70036617	AC007461.1
chr17	70036465	70036617	RP11-84E24.2
chr17	70036465	70036617	SOX9-AS1

chr17	70814013	70814074	SLC39A11
chr17	70989030	70989062	SLC39A11
chr17	71166793	71166870	SSTR2
chr17	71166793	71166870	RP11-143K11.5
chr17	72248008	72248153	TTYH2
chr17	72447026	72447304	GPRC5C
chr17	72615236	72615281	CD300E
chr17	72866073	72866114	FDXR
chr17	73014681	73014721	ICT1
chr17	73086386	73086427	SLC16A5
chr17	73138852	73138868	HN1
chr17	73145202	73145280	HN1
chr17	73225093	73225102	NUP85
chr17	73244921	73245089	GGA3
chr17	73314845	73314877	AC011933.1
chr17	73314845	73314877	GRB2
chr17	73483359	73483408	KIAA0195
chr17	73488320	73488337	KIAA0195
chr17	73542327	73542404	LLGL2
chr17	73664129	73664133	SAP30BP
chr17	73759741	73759748	GALK1
chr17	73780681	73780755	MIR4738
chr17	73780681	73780755	H3F3B
chr17	73780681	73780755	UNK
chr17	73787841	73787856	UNK
chr17	73805029	73805050	UNK
chr17	73841977	73842115	WBP2
chr17	73848853	73848901	WBP2
chr17	73938922	73938993	ACOX1
chr17	73980992	73981093	TEN1
chr17	73980992	73981093	TEN1-CDK3
chr17	74076821	74076979	ZACN
chr17	74116412	74116483	EXOC7
chr17	74297524	74297621	QRICH2
chr17	74379927	74379970	SPHK1
chr17	74379927	74379970	PRPSAP1
chr17	74577053	74577134	ST6GALNAC2
chr17	74637974	74637986	ST6GALNAC1
chr17	74741475	74741521	MFSD11
chr17	75276858	75276888	RP11-285E9.6
chr17	75276858	75276888	9-Sep
chr17	75282433	75282504	9-Sep
chr17	75290856	75290971	9-Sep
chr17	75315850	75315885	9-Sep
chr17	75318934	75319030	9-Sep
chr17	75878008	75878472	FLJ45079
chr17	76179835	76179858	TK1

chr17	76188718	76188746	AFMID
chr17	76219983	76220062	BIRC5
chr17	76219983	76220062	AC087645.1
chr17	76220514	76220634	BIRC5
chr17	76220514	76220634	AC087645.1
chr17	76220639	76220740	BIRC5
chr17	76220639	76220740	AC087645.1
chr17	76230315	76230425	TMEM235
chr17	76468744	76468856	DNAH17
chr17	76723740	76723794	CYTH1
chr17	76988696	76988843	CANT1
chr17	77015620	77015698	C1QTNF1-AS1
chr17	77016084	77016227	C1QTNF1-AS1
chr17	77179346	77179450	RBFOX3
chr17	77772041	77772079	CBX8
chr17	77925785	77925994	TBC1D16
chr17	78040673	78040745	CCDC40
chr17	78978059	78978529	AC127496.1
chr17	78978059	78978529	CHMP6
chr17	78983190	78983287	CHMP6
chr17	79084077	79084097	BAIAP2
chr17	79253951	79253960	SLC38A10
chr17	79380724	79380736	RP11-1055B8.7
chr17	79383860	79383878	RP11-1055B8.7
chr17	79385351	79385408	RP11-1055B8.7
chr17	79386905	79386959	RP11-1055B8.7
chr17	79395735	79395765	RP11-1055B8.7
chr17	79396630	79396633	RP11-1055B8.7
chr17	79513529	79513566	C17orf70
chr17	79520471	79520580	C17orf70
chr17	79527588	79527655	NPLOC4
chr17	79530569	79530594	NPLOC4
chr17	79534980	79535101	NPLOC4
chr17	79649673	79649705	ARL16
chr17	79680292	79680318	SLC25A10
chr17	79680292	79680318	SLC25A10
chr17	79783419	79783946	AC174470.1
chr17	79783419	79783946	FAM195B
chr17	79868846	79868891	PCYT2
chr17	79934763	79934855	ASPSCR1
chr17	79940490	79940508	ASPSCR1
chr17	79967808	79967928	ASPSCR1
chr17	79993054	79993121	DCXR
chr17	80065278	80065497	CCDC57
chr17	80197239	80197357	SLC16A3
chr17	80197239	80197357	CSNK1D
chr17	80208031	80208055	AC132872.1

chr17	80208031	80208055	SLC16A3
chr17	80208031	80208055	CSNK1D
chr17	80214341	80214727	SLC16A3
chr17	80214341	80214727	CSNK1D
chr17	80214341	80214727	AC132872.2
chr17	80218775	80218971	SLC16A3
chr17	80218775	80218971	CSNK1D
chr17	80224715	80224770	CSNK1D
chr17	80224859	80224908	CSNK1D
chr17	80281133	80281192	SECTM1
chr17	80354421	80354727	OGFOD3
chr17	80398349	80398383	HEXDC
chr17	80517265	80517323	FOXK2
chr17	80553658	80553716	FOXK2
chr17	80587491	80587504	WDR45B
chr17	80587491	80587504	FOXK2
chr17	80676320	80676349	FN3KRP
chr17	80678637	80678689	FN3KRP
chr17	80687200	80687299	FN3KRP
chr17	80687200	80687299	RP11-388C12.5
chr17	80866173	80866283	TBCD
chr18	260755	260759	THOC1
chr18	693170	693175	ENOSF1
chr18	2577235	2577356	NDC80
chr18	2582101	2582310	NDC80
chr18	2614953	2614970	NDC80
chr18	2784843	2784877	SMCHD1
chr18	2784843	2784877	RP11-703M24.5
chr18	5400496	5400594	EPB41L3
chr18	5539620	5539655	EPB41L3
chr18	5577329	5577383	EPB41L3
chr18	6511501	6511609	C18orf64
chr18	6513638	6513725	C18orf64
chr18	6575989	6576103	C18orf64
chr18	6588810	6588866	C18orf64
chr18	6589446	6589474	C18orf64
chr18	6590534	6590543	C18orf64
chr18	6754798	6754814	ARHGAP28
chr18	8336437	8336454	AP001094.1
chr18	8336437	8336454	PTPRM
chr18	8336459	8336555	AP001094.1
chr18	8336459	8336555	PTPRM
chr18	8336980	8337038	AP001094.1
chr18	8336980	8337038	PTPRM
chr18	8785688	8785724	SOGA2
chr18	9200512	9200525	RP11-21J18.1
chr18	9200512	9200525	ANKRD12

chr18	9388038	9388042	TWSG1
chr18	9594177	9594232	PPP4R1
chr18	9612687	9612688	PPP4R1
chr18	9723555	9723573	RAB31
chr18	9924900	9924910	VAPA
chr18	10474103	10474244	APCDD1
chr18	10666590	10666810	RP11-856M7.1
chr18	10666590	10666810	PIEZO2
chr18	11874050	11874115	GNAL
chr18	11895184	11895252	MPPE1
chr18	12021701	12021821	IMPA2
chr18	12410717	12410857	SLMO1
chr18	12422247	12422340	SLMO1
chr18	12653383	12653413	SPIRE1
chr18	12658242	12658342	AP005482.1
chr18	12658550	12658562	AP005482.1
chr18	12658573	12658630	AP005482.1
chr18	12686671	12686751	CEP76
chr18	12686671	12686751	PSMG2
chr18	12788083	12788149	PTPN2
chr18	13000311	13000359	CEP192
chr18	13058293	13058295	CEP192
chr18	13103874	13103918	CEP192
chr18	13670870	13670967	FAM210A
chr18	14085053	14085075	ZNF519
chr18	19048687	19048748	GREB1L
chr18	19120538	19120661	ESCO1
chr18	19262964	19262966	ABHD3
chr18	19678334	19678606	RP11-595B24.2
chr18	20557753	20557849	RBBP8
chr18	20799218	20799220	CABLES1
chr18	20799218	20799220	TMEM241
chr18	21066072	21066152	RIOK3
chr18	21088619	21088740	C18orf8
chr18	21088619	21088740	NPC1
chr18	21089569	21089641	C18orf8
chr18	21089569	21089641	NPC1
chr18	21189238	21189255	ANKRD29
chr18	21229466	21229486	ANKRD29
chr18	21375987	21376112	LAMA3
chr18	21414248	21414389	LAMA3
chr18	21511384	21511471	LAMA3
chr18	21730502	21730524	CABYR
chr18	21881257	21881288	OSBPL1A
chr18	22033114	22033218	IMPACT
chr18	22716682	22716703	ZNF521
chr18	22811435	22811494	ZNF521

chr18	23648516	23648521	SS18
chr18	23659029	23659089	SS18
chr18	23660279	23660387	SS18
chr18	23661191	23661329	SS18
chr18	23662462	23662492	SS18
chr18	23664103	23664139	SS18
chr18	25625375	25625393	CDH2
chr18	29519344	29519425	TRAPPC8
chr18	29716379	29716423	GAREM
chr18	30350917	30351071	AC012123.1
chr18	30350917	30351071	KLHL14
chr18	30352010	30352250	AC012123.1
chr18	30352010	30352250	KLHL14
chr18	30353278	30353297	AC012123.1
chr18	31196643	31196690	ASXL3
chr18	31205729	31205732	ASXL3
chr18	31206826	31206957	ASXL3
chr18	31221785	31221787	ASXL3
chr18	31270206	31270280	ASXL3
chr18	31701263	31701280	NOL4
chr18	31708836	31708853	NOL4
chr18	31763558	31763590	NOL4
chr18	32337180	32337252	RP11-138H11.1
chr18	32337180	32337252	DTNA
chr18	32401437	32401439	DTNA
chr18	33067728	33067787	INO80C
chr18	33067728	33067787	RP11-322E11.6
chr18	33239822	33239835	GALNT1
chr18	33642968	33643038	RPRD1A
chr18	34370109	34370114	TPGS2
chr18	34385955	34386059	TPGS2
chr18	34397672	34397734	TPGS2
chr18	34401028	34401092	TPGS2
chr18	34403827	34403834	TPGS2
chr18	34809532	34809584	KIAA1328
chr18	39564844	39564852	PIK3C3
chr18	39639833	39639858	PIK3C3
chr18	43214787	43214844	SLC14A2
chr18	43420851	43420954	SIGLEC15
chr18	43487198	43487254	EPG5
chr18	43673209	43673328	ATP5A1
chr18	43679269	43679306	ATP5A1
chr18	43686671	43686681	HAUS1
chr18	43689613	43689701	HAUS1
chr18	44113720	44113722	LOXHD1
chr18	44135088	44135143	LOXHD1
chr18	44311790	44311806	ST8SIA5

chr18	44338958	44339063	RP11-742D12.2
chr18	44338958	44339063	ST8SIA5
chr18	44484064	44484066	PIAS2
chr18	44486190	44486208	PIAS2
chr18	46944343	46944427	DYM
chr18	47807534	47807586	MBD1
chr18	48577724	48577785	RP11-729L2.2
chr18	48577724	48577785	SMAD4
chr18	48579005	48579022	RP11-729L2.2
chr18	48579005	48579022	SMAD4
chr18	50285157	50285386	DCC
chr18	50335669	50335682	DCC
chr18	50343004	50343056	DCC
chr18	50418778	50418782	DCC
chr18	51714151	51714207	MBD2
chr18	53263337	53263457	TCF4
chr18	53331906	53331989	TCF4
chr18	54318248	54318306	TXNL1
chr18	54388778	54388814	WDR7
chr18	54558064	54558179	WDR7
chr18	54603412	54603460	WDR7
chr18	55038640	55038697	ST8SIA3
chr18	55111608	55111659	ONECUT2
chr18	55134392	55134452	ONECUT2
chr18	55235077	55235108	FECH
chr18	56024689	56024691	NEDD4L
chr18	56598878	56598928	ZNF532
chr18	56621466	56621525	ZNF532
chr18	59771011	59771032	PIGN
chr18	59785780	59785890	PIGN
chr18	59790740	59790777	PIGN
chr18	59830764	59830782	PIGN
chr18	60491423	60491432	AC015989.1
chr18	60491423	60491432	PHLPP1
chr18	60492654	60492724	AC015989.1
chr18	60492654	60492724	PHLPP1
chr18	61254328	61254361	SERPINB13
chr18	61254646	61254684	SERPINB13
chr18	61271174	61271230	SERPINB13
chr18	61575194	61575274	SERPINB10
chr18	61609566	61609601	AC009802.1
chr18	66373517	66373645	TMX3
chr18	66567321	66567474	RP11-861L17.3
chr18	66567321	66567474	CCDC102B
chr18	66569425	66569477	RP11-861L17.3
chr18	66569425	66569477	CCDC102B
chr18	67509627	67509631	CD226

chr18	67509627	67509631	DOK6
chr18	67728602	67728636	RTTN
chr18	68003616	68003740	RP11-484N16.1
chr18	68003616	68003740	RP11-41O4.1
chr18	68019215	68019287	RP11-41O4.1
chr18	69446812	69447012	RP11-723G8.2
chr18	70829180	70829208	RP11-169F17.1
chr18	70840184	70840197	RP11-169F17.1
chr18	70867888	70868024	RP11-169F17.1
chr18	70923048	70923151	RP11-169F17.1
chr18	71807983	71808008	FBXO15
chr18	71814303	71814354	FBXO15
chr18	72163517	72163666	CNDP2
chr18	73119952	73120028	RP11-321M21.3
chr18	73971141	73971284	RP11-94B19.4
chr18	73971772	73971798	RP11-94B19.4
chr18	74240909	74240920	LINC00908
chr18	74242341	74242421	LINC00908
chr18	74269932	74270021	LINC00908
chr18	74273820	74273834	LINC00908
chr18	74314695	74314745	LINC00908
chr18	74322802	74322861	LINC00908
chr18	74507493	74507614	RP11-162A12.2
chr18	74528711	74528765	RP11-162A12.2
chr18	74702847	74702870	MBP
chr18	76762289	76762291	SALL3
chr18	77275812	77276057	AC018445.1
chr18	77275812	77276057	NFATC1
chr18	77709594	77709636	PQLC1
chr18	77709654	77709656	PQLC1
chr18	77709709	77709838	PQLC1
chr18	77736769	77736787	TXNL4A
chr18	77739533	77739640	TXNL4A
chr18	77891575	77891688	ADNP2
chr18	77905318	77905362	ADNP2
chr18	77906042	77906137	AC139100.2
chr18	77906305	77906427	AC139100.2
chr18	77920399	77920486	AC139100.2
chr18	77920399	77920486	PARD6G
chr18	77926702	77926820	AC139100.2
chr18	77926702	77926820	PARD6G
chr18	77926901	77927019	AC139100.2
chr18	77926901	77927019	PARD6G
chr18	77933763	77933818	AC139100.2
chr18	77933763	77933818	PARD6G
chr18	77935969	77936011	AC139100.2
chr18	77935969	77936011	PARD6G

chr18	77959888	77959901	AC139100.3
chr18	77959888	77959901	PARD6G
chr19	435946	435961	SHC2
chr19	439398	439491	SHC2
chr19	641178	641268	FGF22
chr19	661356	661402	RNF126
chr19	872923	873009	MED16
chr19	999601	999621	AC004528.1
chr19	999914	999952	AC004528.1
chr19	999914	999952	AC004528.4
chr19	1036878	1036999	CNN2
chr19	1066536	1066640	HMHA1
chr19	1071703	1071765	HMHA1
chr19	1272968	1272992	CIRBP
chr19	1386517	1386626	NDUFS7
chr19	1416456	1416540	DAZAP1
chr19	1440838	1441275	AC027307.3
chr19	1508856	1508907	CTB-25B13.9
chr19	1508856	1508907	ADAMTSL5
chr19	1974687	1974751	CSNK1G2
chr19	1991876	1992055	BTBD2
chr19	2013834	2013879	BTBD2
chr19	2215457	2215570	AC004490.1
chr19	2215457	2215570	DOT1L
chr19	2227362	2227466	DOT1L
chr19	2270444	2270675	OAZ1
chr19	2326047	2326138	LSM7
chr19	2517051	2517080	GNG7
chr19	2813494	2813571	THOP1
chr19	2826248	2826328	ZNF554
chr19	3146401	3146414	GNA15
chr19	3146401	3146414	AC005264.2
chr19	3567143	3567240	MFSD12
chr19	3572887	3573019	HMG20B
chr19	3572887	3573019	MFSD12
chr19	3610665	3611074	CACTIN-AS1
chr19	3610665	3611074	CACTIN
chr19	3611227	3611351	CACTIN-AS1
chr19	3611227	3611351	CACTIN
chr19	3768197	3768312	MRPL54
chr19	3895125	3895195	ATCAY
chr19	3976150	3976298	EEF2
chr19	4042083	4042496	AC016586.1
chr19	4338212	4338261	STAP2
chr19	4455411	4455421	UBXN6
chr19	4486380	4486382	HDGFRP2
chr19	4496916	4497153	HDGFRP2

chr19	4641595	4641662	TNFAIP8L1
chr19	4641595	4641662	C19orf10
chr19	4717627	4717694	DPP9
chr19	4719215	4719336	DPP9
chr19	4866892	4866937	PLIN3
chr19	5140263	5140346	KDM4B
chr19	5690832	5690942	RPL36
chr19	5712474	5712479	LONP1
chr19	5772104	5772446	CATSPERD
chr19	5870582	5870634	FUT5
chr19	5870582	5870634	AC024592.12
chr19	5901455	5901465	AC104532.3
chr19	5901455	5901465	FUT5
chr19	5901455	5901465	AC024592.12
chr19	5901455	5901465	NDUFA11
chr19	5935828	5935850	RANBP3
chr19	5950839	5950883	RANBP3
chr19	5965744	5965828	RANBP3
chr19	5974361	5974438	RANBP3
chr19	5976491	5976585	RANBP3
chr19	6392214	6392421	GTF2F1
chr19	6430532	6430629	SLC25A41
chr19	6441353	6441495	SLC25A23
chr19	6500719	6500753	TUBB4A
chr19	7011817	7012062	AC025278.1
chr19	7159628	7159656	INSR
chr19	7696118	7696123	PET100
chr19	7696118	7696123	CTD-3214H19.4
chr19	7797678	7797775	CLEC4G
chr19	7798724	7798731	CLEC4G
chr19	8026279	8026496	ELAVL1
chr19	8066936	8067000	ELAVL1
chr19	8370586	8370689	CD320
chr19	8384185	8384278	NDUFA7
chr19	8384185	8384278	NDUFA7
chr19	8439077	8439112	ANGPTL4
chr19	8552893	8552939	HNRNPM
chr19	9273731	9273778	ZNF317
chr19	9443691	9443773	ZNF559
chr19	9443691	9443773	ZNF559-ZNF177
chr19	9443691	9443773	ZNF177
chr19	9453977	9453988	ZNF559
chr19	9453977	9453988	ZNF559-ZNF177
chr19	9453977	9453988	ZNF177
chr19	9475538	9475624	ZNF559-ZNF177
chr19	9475538	9475624	ZNF177
chr19	9486992	9487084	ZNF559-ZNF177

chr19	9486992	9487084	ZNF177
chr19	9732229	9732321	C19orf82
chr19	9737460	9737536	C19orf82
chr19	9738392	9738482	C19orf82
chr19	9742904	9743048	C19orf82
chr19	9800814	9801937	ZNF812
chr19	9804370	9804496	ZNF812
chr19	9805455	9805543	ZNF812
chr19	9806770	9806794	ZNF812
chr19	9925326	9925345	FBXL12
chr19	10217679	10217681	PPAN-P2RY11
chr19	10217679	10217681	PPAN
chr19	10290557	10290658	DNMT1
chr19	10311550	10311559	DNMT1
chr19	10471727	10471749	TYK2
chr19	10480433	10480553	TYK2
chr19	10511289	10511369	CDC37
chr19	10613917	10614234	KEAP1
chr19	10627717	10627764	S1PR5
chr19	10695646	10695720	AP1M2
chr19	10751715	10751757	AC011475.1
chr19	10751715	10751757	SLC44A2
chr19	10752056	10752093	AC011475.1
chr19	10752056	10752093	SLC44A2
chr19	10817386	10817501	QTRT1
chr19	10819957	10820040	QTRT1
chr19	10914315	10914344	DNM2
chr19	10922429	10922440	DNM2
chr19	11019226	11019258	CARM1
chr19	11175869	11175877	SMARCA4
chr19	11201275	11201595	LDLR
chr19	11247988	11248091	SPC24
chr19	11418538	11418621	TSPAN16
chr19	11418538	11418621	CTC-510F12.4
chr19	11545283	11545351	CCDC151
chr19	11611540	11611630	CTC-398G3.6
chr19	11611540	11611630	ZNF653
chr19	11621432	11621475	ECSIT
chr19	11650567	11650569	CNN1
chr19	11660848	11661122	CNN1
chr19	11888931	11888935	ZNF441
chr19	12072476	12072573	ZNF763
chr19	12072476	12072573	ZNF763
chr19	12132745	12132873	CTD-2006C1.2
chr19	12132745	12132873	ZNF433
chr19	12132745	12132873	CTD-2006C1.10
chr19	12140955	12141013	CTD-2006C1.2

chr19	12140955	12141013	ZNF433
chr19	12140955	12141013	CTD-2006C1.10
chr19	12145643	12145689	CTD-2006C1.2
chr19	12145643	12145689	ZNF433
chr19	12145643	12145689	CTD-2006C1.10
chr19	12163976	12164074	ZNF878
chr19	12167083	12167127	ZNF878
chr19	12247666	12247691	ZNF788
chr19	12247666	12247691	ZNF20
chr19	12247666	12247691	ZNF625-ZNF20
chr19	12353623	12353659	ZNF44
chr19	12355396	12355488	ZNF44
chr19	12358092	12359493	ZNF44
chr19	12360793	12360853	ZNF44
chr19	12361057	12361183	ZNF44
chr19	12361586	12361618	ZNF44
chr19	12402035	12402065	ZNF44
chr19	12624558	12624647	ZNF709
chr19	12624558	12624647	ZNF709
chr19	12661277	12661466	ZNF564
chr19	12661277	12661466	CTD-2192J16.20
chr19	12661277	12661466	ZNF709
chr19	12707963	12707965	ZNF490
chr19	12720723	12720788	ZNF490
chr19	12723040	12723171	ZNF791
chr19	12723040	12723171	ZNF490
chr19	12783277	12783363	WDR83
chr19	12882631	12882692	HOOK2
chr19	13008940	13008998	GCDH
chr19	13254895	13254953	STX10
chr19	13347394	13347432	CACNA1A
chr19	13886285	13886320	C19orf53
chr19	13890884	13890900	AC008686.1
chr19	13891002	13891017	AC008686.1
chr19	13892140	13892311	AC008686.1
chr19	13899021	13899062	AC008686.1
chr19	13900051	13900105	AC008686.1
chr19	13900149	13900157	AC008686.1
chr19	13900900	13900972	AC008686.1
chr19	13999176	13999193	C19orf57
chr19	14502203	14502304	CD97
chr19	14590630	14590669	GIPC1
chr19	14628291	14628300	DNAJB1
chr19	14628291	14628300	TECR
chr19	14656682	14656700	TECR
chr19	14812396	14812410	ZNF333
chr19	14825550	14825626	ZNF333

chr19	14834747	14834836	AC090427.1
chr19	14834747	14834836	ZNF333
chr19	14834839	14834892	AC090427.1
chr19	14834839	14834892	ZNF333
chr19	14834895	14834918	AC090427.1
chr19	14834895	14834918	ZNF333
chr19	14835005	14835121	AC090427.1
chr19	14835005	14835121	ZNF333
chr19	15480013	15480035	AKAP8
chr19	15509441	15509577	AKAP8L
chr19	15591010	15591053	PGLYRP2
chr19	15784035	15784091	CYP4F12
chr19	16254543	16254584	HSH2D
chr19	16262257	16262341	HSH2D
chr19	16262361	16262388	HSH2D
chr19	16297264	16297308	CTD-2562J15.4
chr19	16297264	16297308	FAM32A
chr19	16300199	16300306	CTD-2562J15.4
chr19	16300199	16300306	FAM32A
chr19	16345958	16346044	AP1M1
chr19	16667734	16667742	SLC35E1
chr19	16667734	16667742	CTD-3222D19.2
chr19	16675917	16675943	SLC35E1
chr19	16675917	16675943	CTD-3222D19.2
chr19	16739333	16739770	MED26
chr19	16739333	16739770	CTC-429P9.4
chr19	16739333	16739770	CTC-429P9.2
chr19	16766157	16766179	CTC-429P9.4
chr19	16766157	16766179	SMIM7
chr19	16799734	16799754	TMEM38A
chr19	16849037	16849069	NWD1
chr19	17288606	17288695	CTD-3032J10.4
chr19	17288606	17288695	MYO9B
chr19	17325269	17325327	MYO9B
chr19	17388193	17388195	USHBP1
chr19	17388193	17388195	BABAM1
chr19	17388193	17388195	CTD-2278I10.6
chr19	17412992	17413041	ABHD8
chr19	17412992	17413041	MRPL34
chr19	17414051	17414055	ABHD8
chr19	17414051	17414055	MRPL34
chr19	17420976	17421015	ABHD8
chr19	17420976	17421015	DDA1
chr19	17462371	17462451	PLVAP
chr19	17531770	17531905	MVB12A
chr19	17531770	17531905	CTD-2521M24.6
chr19	17656032	17656166	FAM129C

chr19	17832060	17832121	MAP1S
chr19	18390829	18390854	JUND
chr19	18420959	18421024	LSM4
chr19	18459758	18459878	PGPEP1
chr19	18581804	18581845	ELL
chr19	18653698	18653759	FKBP8
chr19	18770497	18770554	KLHL26
chr19	18988826	18988907	CERS1
chr19	18988826	18988907	GDF1
chr19	19109733	19109794	SUGP2
chr19	19114323	19114417	SUGP2
chr19	19118181	19118256	SUGP2
chr19	19148361	19148475	ARMC6
chr19	19150131	19150223	ARMC6
chr19	19151499	19151601	ARMC6
chr19	19311628	19311672	RFXANK
chr19	19439879	19439896	MAU2
chr19	19901781	19901852	CTC-559E9.4
chr19	19901781	19901852	CTC-559E9.6
chr19	19901781	19901852	ZNF506
chr19	19902427	19902540	CTC-559E9.4
chr19	19902427	19902540	CTC-559E9.6
chr19	19902427	19902540	ZNF506
chr19	19903220	19903293	CTC-559E9.4
chr19	19903220	19903293	CTC-559E9.6
chr19	19903220	19903293	ZNF506
chr19	20036910	20036938	ZNF93
chr19	20036910	20036938	AC007204.2
chr19	20047769	20047782	AC007204.1
chr19	20047769	20047782	AC007204.2
chr19	20047924	20048018	AC007204.1
chr19	20047924	20048018	AC007204.2
chr19	20048512	20048639	AC007204.1
chr19	20048512	20048639	AC007204.2
chr19	20115546	20115556	ZNF682
chr19	20217757	20217761	ZNF90
chr19	20232031	20232143	ZNF90
chr19	20746847	20746944	ZNF737
chr19	20746847	20746944	CTC-513N18.7
chr19	20973438	20973578	ZNF66
chr19	20991839	20991922	ZNF66
chr19	21128305	21128515	ZNF85
chr19	21232311	21232373	ZNF430
chr19	21265152	21265154	ZNF714
chr19	21510830	21510943	ZNF708
chr19	21543277	21543398	ZNF738
chr19	21737712	21737869	ZNF429

chr19	21737712	21737869	RP11-678G14.2
chr19	21925880	21926025	ZNF100
chr19	22174241	22174270	ZNF208
chr19	22243310	22243414	ZNF257
chr19	22257174	22257355	ZNF257
chr19	23168464	23168633	ZNF728
chr19	23326376	23326500	ZNF730
chr19	23839584	23839624	ZNF675
chr19	24009964	24010851	RP11-255H23.4
chr19	24009964	24010851	RP11-255H23.2
chr19	24009964	24010851	RPSAP58
chr19	24274703	24274817	ZNF254
chr19	24286549	24286631	ZNF254
chr19	30196255	30196301	C19orf12
chr19	30416441	30416473	URI1
chr19	30471065	30471067	URI1
chr19	30505088	30505099	URI1
chr19	31640362	31640757	AC020952.1
chr19	31796563	31796669	TSHZ3
chr19	32896383	32896451	AC007773.3
chr19	32896383	32896451	AC007773.2
chr19	32896383	32896451	DPY19L3
chr19	32958380	32958394	DPY19L3
chr19	33427920	33428026	CEP89
chr19	33886996	33887061	PEPD
chr19	33968193	33968205	PEPD
chr19	34686124	34686147	LSM14A
chr19	34971874	34971931	WTIP
chr19	34972121	34972626	WTIP
chr19	35597290	35597736	AC020907.1
chr19	35647747	35647877	FXYD5
chr19	35988401	35988440	DMKN
chr19	36279607	36279684	ARHGAP33
chr19	36572696	36572776	WDR62
chr19	36602535	36602612	OVOL3
chr19	36602668	36602706	OVOL3
chr19	36608693	36608830	TBCB
chr19	36640944	36641012	CAPNS1
chr19	36641216	36641255	CAPNS1
chr19	36936941	36937017	ZNF566
chr19	37148555	37148652	ZNF461
chr19	37583948	37584054	ZNF420
chr19	37583948	37584054	CTC-454I21.3
chr19	37586933	37586940	ZNF420
chr19	37586933	37586940	CTC-454I21.3
chr19	37602502	37602521	ZNF420
chr19	37602502	37602521	CTC-454I21.3

chr19	37602502	37602521	ZNF585A
chr19	37606063	37606067	ZNF420
chr19	37606063	37606067	CTC-454I21.3
chr19	37606063	37606067	ZNF585A
chr19	37641737	37641781	CTC-454I21.3
chr19	37641737	37641781	ZNF585A
chr19	37686811	37686921	ZNF585B
chr19	37686811	37686921	CTC-454I21.3
chr19	37690364	37690399	ZNF585B
chr19	37690364	37690399	CTC-454I21.3
chr19	37837790	37837882	HKR1
chr19	38036986	38037068	CTD-3064H18.4
chr19	38036986	38037068	ZNF793
chr19	38197408	38197484	CTD-2528L19.4
chr19	38197408	38197484	ZNF607
chr19	38249742	38249779	ZNF573
chr19	38261125	38261237	ZNF573
chr19	38578396	38578427	SIPA1L3
chr19	38622294	38622375	SIPA1L3
chr19	38664266	38664312	SIPA1L3
chr19	38826783	38826890	CATSPERG
chr19	38885001	38885036	SPRED3
chr19	39029199	39029313	RYR1
chr19	39119143	39119208	EIF3K
chr19	39121342	39121460	EIF3K
chr19	39220629	39220660	ACTN4
chr19	39332264	39332322	HNRNPL
chr19	39341708	39341795	HNRNPL
chr19	39381298	39381302	SIRT2
chr19	39382563	39382606	SIRT2
chr19	39418985	39418990	SARS2
chr19	39418985	39418990	CTC-360G5.8
chr19	39438037	39438058	SARS2
chr19	39438037	39438058	CTC-360G5.8
chr19	39438037	39438058	FBXO17
chr19	39481354	39481401	FBXO27
chr19	39505027	39505187	FBXO27
chr19	39616428	39616519	PAK4
chr19	39877828	39877867	PAF1
chr19	39898079	39898121	ZFP36
chr19	39930212	39930481	AC011500.1
chr19	39930212	39930481	SUPT5H
chr19	39930580	39930624	AC011500.1
chr19	39930580	39930624	SUPT5H
chr19	39931930	39932082	AC011500.1
chr19	39931930	39932082	SUPT5H
chr19	39975792	39975841	TIMM50

chr19	40006879	40006890	SELV
chr19	40397967	40398510	FCGBP
chr19	40399239	40399855	FCGBP
chr19	40400250	40400823	FCGBP
chr19	40402134	40402471	FCGBP
chr19	40405919	40406060	FCGBP
chr19	40407936	40408135	FCGBP
chr19	40408254	40408848	FCGBP
chr19	40411638	40412230	FCGBP
chr19	40517158	40517255	ZNF546
chr19	40740387	40740478	AKT2
chr19	40750318	40750321	AKT2
chr19	40838441	40838550	C19orf47
chr19	40840742	40840811	C19orf47
chr19	41264689	41264706	SNRPA
chr19	41868888	41868982	CTC-435M10.3
chr19	41868888	41868982	TMEM91
chr19	41868888	41868982	B9D2
chr19	41924630	41924722	CTC-435M10.3
chr19	41924630	41924722	BCKDHA
chr19	41937785	41937793	ATP5SL
chr19	42311149	42311258	CEACAM3
chr19	42365549	42365629	RPS19
chr19	42367941	42367987	RPS19
chr19	42427244	42427385	ARHGEF1
chr19	42432983	42433138	ARHGEF1
chr19	42433807	42433895	ARHGEF1
chr19	42747003	42747458	AC006486.1
chr19	42747003	42747458	AC006486.9
chr19	42825727	42825826	TMEM145
chr19	42882852	42882902	MEGF8
chr19	43237820	43237878	PSG3
chr19	43519919	43520197	PSG11
chr19	43523806	43523864	PSG11
chr19	43697811	43697968	PSG4
chr19	43700969	43700979	PSG4
chr19	43703031	43703089	PSG4
chr19	43863300	43863418	CD177
chr19	44084517	44084588	PINLYP
chr19	44084517	44084588	XRCC1
chr19	44084517	44084588	L34079.2
chr19	44112657	44112755	SRRM5
chr19	44112657	44112755	ZNF428
chr19	44165509	44165594	PLAUR
chr19	44567418	44567461	ZNF223
chr19	44567418	44567461	ZNF223
chr19	44653924	44653943	ZNF234

chr19	44754318	44754400	ZNF235
chr19	44754318	44754400	ZNF233
chr19	44808854	44809060	ZNF235
chr19	44871241	44871257	ZNF112
chr19	44871241	44871257	CTC-512J12.6
chr19	45000625	45000726	ZNF180
chr19	45422098	45422155	APOC1
chr19	45453408	45453457	CTB-129P6.11
chr19	45454193	45454283	CTB-129P6.11
chr19	45457152	45457241	CTB-129P6.11
chr19	45511716	45511738	RELB
chr19	45705699	45705765	AC005779.2
chr19	45705699	45705765	AC006126.3
chr19	45705699	45705765	MARK4
chr19	45719934	45720151	AC006126.3
chr19	45719934	45720151	EXOC3L2
chr19	45719934	45720151	MARK4
chr19	45842445	45842639	L47234.1
chr19	45842445	45842639	KLC3
chr19	45882978	45883184	PPP1R13L
chr19	45922669	45922747	ERCC1
chr19	46028136	46028333	VASP
chr19	46118188	46118294	EML2
chr19	46131985	46132053	EML2
chr19	46147967	46148009	EML2
chr19	46292578	46292745	DMWD
chr19	46498339	46498418	CCDC61
chr19	46498683	46498762	CCDC61
chr19	46813799	46813940	HIF3A
chr19	46894408	46894431	PPP5C
chr19	46995339	46995444	PNMAL2
chr19	46995339	46995444	PPP5D1
chr19	47050313	47050398	PPP5D1
chr19	47050313	47050398	AC011551.3
chr19	47079856	47079974	PPP5D1
chr19	47079856	47079974	AC011551.3
chr19	47092403	47092492	PPP5D1
chr19	47092403	47092492	AC011551.3
chr19	47092821	47092903	PPP5D1
chr19	47092821	47092903	AC011551.3
chr19	47188713	47188778	PRKD2
chr19	47237105	47237132	STRN4
chr19	47645867	47645868	SAE1
chr19	48008716	48008800	NAPA
chr19	48363370	48363539	TPRX2P
chr19	48364059	48364769	TPRX2P
chr19	48564664	48564699	CTD-2265M8.2

chr19	48564664	48564699	PLA2G4C
chr19	48600380	48600384	PLA2G4C
chr19	48612077	48612179	PLA2G4C
chr19	48709695	48709777	CTC-453G23.8
chr19	48709695	48709777	ZNF114
chr19	48709695	48709777	CARD8
chr19	48727114	48727125	ZNF114
chr19	48727114	48727125	CARD8
chr19	48736595	48736661	ZNF114
chr19	48736595	48736661	CARD8
chr19	48863808	48863825	TMEM143
chr19	48891012	48891078	KDELR1
chr19	49463496	49463593	BAX
chr19	49497433	49497555	RUVBL2
chr19	49559433	49559852	CGB7
chr19	49559433	49559852	NTF4
chr19	49560378	49560453	CGB7
chr19	49560378	49560453	NTF4
chr19	49605371	49605396	SNRNP70
chr19	49619082	49619255	LIN7B
chr19	49809342	49809389	SLC6A16
chr19	49894943	49895028	CCDC155
chr19	49945535	49945560	SLC17A7
chr19	49982947	49983002	CTD-3148I10.9
chr19	49982947	49983002	FLT3LG
chr19	49982947	49983002	CTD-3148I10.15
chr19	50003781	50004614	MIR150
chr19	50003781	50004614	hsa-mir-150
chr19	50018434	50018510	FCGRT
chr19	50023926	50023963	FCGRT
chr19	50050078	50050122	RCN3
chr19	50199739	50199804	CPT1C
chr19	50382580	50382612	TBC1D17
chr19	50489753	50489801	VRK3
chr19	50518989	50519050	VRK3
chr19	50543791	50543885	ZNF473
chr19	50664009	50664037	IZUMO2
chr19	50728081	50728085	MYH14
chr19	50980396	50980478	EMC10
chr19	51166417	51166426	SYT3
chr19	51166417	51166426	SHANK1
chr19	51325983	51326031	KLK1
chr19	51326133	51326208	KLK1
chr19	51377552	51377581	AC037199.1
chr19	51377552	51377581	KLK2
chr19	51380960	51381012	KLK2
chr19	51566889	51567118	KLK13

chr19	51567855	51567910	KLK13
chr19	51767677	51767693	SIGLECL1
chr19	51767677	51767693	CTD-3187F8.2
chr19	51842918	51842920	VSIG10L
chr19	51979838	51979853	CEACAM18
chr19	52095889	52096304	AC018755.1
chr19	52097202	52097574	AC018755.1
chr19	52364179	52364231	ZNF577
chr19	52403051	52403053	CTC-429C10.2
chr19	52403051	52403053	ZNF649
chr19	52520946	52520950	ZNF614
chr19	52820669	52820766	ZNF480
chr19	52820669	52820766	CTD-2525I3.6
chr19	53075903	53075931	ZNF701
chr19	53076345	53076463	ZNF701
chr19	53120563	53120588	ZNF83
chr19	53211527	53211579	ZNF611
chr19	53214553	53214557	ZNF611
chr19	53349811	53349864	ZNF28
chr19	53349811	53349864	ZNF468
chr19	53367282	53367412	ZNF320
chr19	53377245	53377325	ZNF320
chr19	53456537	53456642	ZNF816
chr19	53456537	53456642	ZNF321P
chr19	53991462	53991554	ZNF813
chr19	54006910	54006950	CTD-2224J9.8
chr19	54006910	54006950	ZNF813
chr19	54103573	54103688	CTB-167G5.5
chr19	54104519	54104626	CTB-167G5.5
chr19	54105251	54105417	CTB-167G5.5
chr19	54106606	54106643	CTB-167G5.5
chr19	54306985	54306990	NLRP12
chr19	54608904	54609010	NDUFA3
chr19	54735617	54735630	RPS9
chr19	54735617	54735630	LILRB3
chr19	54735617	54735630	LILRA6
chr19	54746567	54746600	RPS9
chr19	54746567	54746600	LILRB3
chr19	54746567	54746600	LILRA6
chr19	54800046	54800104	LILRA3
chr19	54801927	54802229	LILRA3
chr19	54802483	54802779	LILRA3
chr19	54803016	54803318	LILRA3
chr19	54803466	54803753	LILRA3
chr19	54803943	54803978	LILRA3
chr19	54804140	54804221	LILRA3
chr19	54804604	54804606	LILRA3

chr19	54926427	54926468	TTYH1
chr19	54927230	54927240	TTYH1
chr19	54927963	54928098	TTYH1
chr19	54971319	54971327	LENG8
chr19	55009463	55009478	LAIR2
chr19	55053732	55053791	KIR3DX1
chr19	55147372	55147527	LILRB1
chr19	55147372	55147527	AC009892.10
chr19	55150145	55150189	AC009892.10
chr19	55275343	55275393	KIR2DP1
chr19	55275343	55275393	CTB-61M7.1
chr19	55275343	55275393	KIR2DL3
chr19	55275343	55275393	KIR3DL1
chr19	55275343	55275393	KIR2DL4
chr19	55485417	55485492	CTC-550B14.1
chr19	55485417	55485492	NLRP2
chr19	55488228	55488232	NLRP2
chr19	55500925	55500928	NLRP2
chr19	55644937	55645042	TNNT1
chr19	55685378	55685395	CTD-2587H24.5
chr19	55685378	55685395	SYT5
chr19	55856095	55856484	SUV420H2
chr19	55856095	55856484	AC020922.1
chr19	55949928	55949977	SHISA7
chr19	56170228	56170337	U2AF2
chr19	56173137	56173139	U2AF2
chr19	56221089	56221156	EPN1
chr19	56221089	56221156	NLRP9
chr19	56222799	56222913	NLRP9
chr19	56309595	56309670	NLRP11
chr19	56312458	56312542	NLRP11
chr19	56529978	56530030	NLRP5
chr19	56662314	56662350	AC024580.1
chr19	56662314	56662350	ZNF444
chr19	56662571	56663250	AC024580.1
chr19	56662571	56663250	ZNF444
chr19	56751773	56751906	ZSCAN5A
chr19	56751773	56751906	ZSCAN5D
chr19	56755555	56755795	ZSCAN5A
chr19	56755555	56755795	ZSCAN5D
chr19	56756591	56756794	ZSCAN5A
chr19	56756591	56756794	ZSCAN5D
chr19	56757685	56757835	ZSCAN5A
chr19	56757685	56757835	ZSCAN5D
chr19	56758103	56758863	ZSCAN5A
chr19	56758103	56758863	ZSCAN5D
chr19	56784204	56784288	AC006116.20

chr19	56784204	56784288	ZSCAN5A
chr19	56794364	56794389	AC006116.20
chr19	56794364	56794389	ZSCAN5A
chr19	56795536	56795575	AC006116.20
chr19	56795536	56795575	ZSCAN5A
chr19	56796367	56796393	AC006116.20
chr19	56796367	56796393	ZSCAN5A
chr19	56797751	56797884	AC006116.20
chr19	56797751	56797884	ZSCAN5A
chr19	56806436	56806522	AC006116.20
chr19	56806436	56806522	ZSCAN5A
chr19	56807328	56807336	AC006116.20
chr19	56807328	56807336	ZSCAN5A
chr19	56807705	56807731	AC006116.20
chr19	56807705	56807731	ZSCAN5A
chr19	56808874	56808900	AC006116.20
chr19	56808874	56808900	ZSCAN5A
chr19	56816143	56816199	AC006116.20
chr19	56816143	56816199	ZSCAN5A
chr19	56821493	56821575	AC006116.20
chr19	56821493	56821575	ZSCAN5A
chr19	56887413	56887490	ZNF582
chr19	56887413	56887490	ZNF542
chr19	56960602	56960706	ZNF667
chr19	56978358	56978435	ZNF667
chr19	56981283	56981424	ZNF667
chr19	57059747	57059775	ZFP28
chr19	57059747	57059775	AC007228.11
chr19	57093918	57093970	ZNF470
chr19	57293828	57293839	ZIM2
chr19	57293828	57293839	AC006115.3
chr19	57882341	57882454	ZNF547
chr19	57882341	57882454	AC003002.4
chr19	57902090	57902213	ZNF548
chr19	57902090	57902213	AC003002.4
chr19	57902090	57902213	AC003002.6
chr19	57904169	57904297	ZNF548
chr19	57904169	57904297	AC003002.4
chr19	57904169	57904297	AC003002.6
chr19	58141761	58141862	ZNF211
chr19	58145081	58145128	ZNF211
chr19	58145154	58145180	ZNF211
chr19	58203868	58204128	AC004017.1
chr19	58203868	58204128	ZNF551
chr19	58203868	58204128	AC003006.7
chr19	58268240	58268247	ZNF776
chr19	58315356	58315432	ZNF552

chr19	58315356	58315432	ZNF586
chr19	58376238	58376287	ZNF814
chr19	58376238	58376287	ZNF587
chr19	58411778	58411882	CTD-2583A14.9
chr19	58411778	58411882	ZNF417
chr19	58513479	58514414	CTD-2368P22.1
chr19	58513479	58514414	ZNF606
chr19	58514579	58514704	CTD-2368P22.1
chr19	58514579	58514704	ZNF606
chr19	58515751	58515789	CTD-2368P22.1
chr19	58517275	58517367	CTD-2368P22.1
chr19	58520699	58520818	CTD-2368P22.1
chr19	58593060	58593121	ZNF135
chr19	58869318	58869432	CTD-2619J13.9
chr19	58869318	58869432	CTD-2619J13.8
chr19	58869318	58869432	ZNF497
chr19	59055503	59055578	TRIM28
chr20	334756	334760	NRSN2
chr20	1316935	1317330	AL136531.1
chr20	1316935	1317330	SDCBP2-AS1
chr20	1454505	1454515	SIRPB2
chr20	1520931	1520981	RP4-576H24.2
chr20	1520931	1520981	SIRPD
chr20	1520931	1520981	RP4-576H24.4
chr20	2447904	2447961	SNRPB
chr20	2447904	2447961	RP4-734P14.4
chr20	2740449	2740615	EBF4
chr20	2903905	2903931	PTPRA
chr20	4053172	4053311	RP11-352D3.2
chr20	4055394	4055595	RP11-352D3.2
chr20	4713038	4713322	PRNT
chr20	7999623	7999686	TMX4
chr20	8731451	8731561	PLCB1
chr20	10401194	10401385	MKKS
chr20	10616944	10617012	SLX4IP
chr20	11008541	11008554	C20orf187
chr20	11008810	11008936	C20orf187
chr20	11009839	11009854	C20orf187
chr20	13595408	13595524	TASP1
chr20	13597663	13597770	TASP1
chr20	13795064	13795089	NDUFAF5
chr20	13895767	13895887	SEL1L2
chr20	17585755	17585774	DSTN
chr20	18375278	18375291	DZANK1
chr20	18375389	18375502	DZANK1
chr20	20050389	20050413	C20orf26
chr20	20088290	20088357	C20orf26

chr20	23330015	23330039	RP3-322G13.7
chr20	23330015	23330039	AL096677.1
chr20	23330259	23330262	RP3-322G13.7
chr20	23330259	23330262	AL096677.1
chr20	23334039	23334066	NXT1
chr20	23334039	23334066	AL096677.1
chr20	24945011	24945034	APMAP
chr20	25062077	25062084	VSX1
chr20	25207122	25207370	AL035252.1
chr20	25207122	25207370	ENTPD6
chr20	25270484	25270566	PYGB
chr20	25319600	25319640	ABHD12
chr20	25320258	25320313	ABHD12
chr20	25400320	25400441	GINS1
chr20	25745605	25745637	FAM182B
chr20	29623185	29623254	FRG1B
chr20	29624032	29624092	FRG1B
chr20	29625873	29625984	FRG1B
chr20	29628227	29628331	FRG1B
chr20	29628396	29628413	FRG1B
chr20	29630699	29630707	FRG1B
chr20	29631538	29631629	FRG1B
chr20	29632611	29632721	FRG1B
chr20	29633898	29633910	FRG1B
chr20	30309001	30309041	AL160175.1
chr20	30309001	30309041	BCL2L1
chr20	30309125	30309134	AL160175.1
chr20	30309125	30309134	BCL2L1
chr20	30657491	30657554	HCK
chr20	30724680	30724708	TM9SF4
chr20	30959581	30959586	ASXL1
chr20	31073563	31073567	C20orf112
chr20	31074499	31074604	C20orf112
chr20	31097669	31097743	C20orf112
chr20	31189469	31189498	RP11-410N8.4
chr20	31196112	31196504	RP11-410N8.4
chr20	31330282	31330371	COMMD7
chr20	31621109	31621175	BPIFB6
chr20	31823091	31823387	AL121901.1
chr20	33150292	33150369	PIGU
chr20	33481372	33481425	ACSS2
chr20	33721908	33721946	EDEM2
chr20	33972427	33972447	UQCC1
chr20	33974187	33974192	UQCC1
chr20	33997226	33997270	UQCC1
chr20	34131407	34131414	ERGIC3
chr20	34136917	34136961	ERGIC3

chr20	34280257	34280292	NFS1
chr20	34295862	34295914	RBM39
chr20	34322137	34322179	RBM39
chr20	34324471	34324489	RBM39
chr20	34327332	34327469	RBM39
chr20	34328448	34328519	RBM39
chr20	34433307	34433363	PHF20
chr20	34437387	34437405	PHF20
chr20	34756461	34756487	AL121895.1
chr20	34756461	34756487	EPB41L1
chr20	34756535	34756540	AL121895.1
chr20	34756535	34756540	EPB41L1
chr20	34758148	34758180	AL121895.1
chr20	34758148	34758180	EPB41L1
chr20	35710381	35710432	RBL1
chr20	36678155	36678215	RPRD1B
chr20	36917429	36917559	CTD-2308N23.2
chr20	36917429	36917559	BPI
chr20	36919751	36919836	CTD-2308N23.2
chr20	36919751	36919836	BPI
chr20	37216744	37217098	ADIG
chr20	37278985	37279103	ARHGAP40
chr20	37279325	37279362	ARHGAP40
chr20	39809144	39809158	PLCG1
chr20	39809144	39809158	RP3-511B24.6
chr20	39809144	39809158	ZHX3
chr20	40111177	40111191	CHD6
chr20	42087793	42087944	SRSF6
chr20	42176751	42176885	L3MBTL1
chr20	42908223	42908261	GDAP1L1
chr20	42908360	42908407	GDAP1L1
chr20	43080751	43080813	C20orf62
chr20	43090525	43090922	C20orf62
chr20	43093805	43093919	C20orf62
chr20	43234346	43234385	PKIG
chr20	43234346	43234385	Z97053.1
chr20	43248095	43248126	PKIG
chr20	43248095	43248126	Z97053.1
chr20	44028330	44028391	SYS1-DBNDD2
chr20	44028330	44028391	TP53TG5
chr20	44075127	44075525	AL031663.2
chr20	44095910	44095954	AL031663.1
chr20	44099793	44099809	AL031663.1
chr20	44099793	44099809	WFDC2
chr20	44107438	44107452	AL031663.1
chr20	44107438	44107452	WFDC2
chr20	44163796	44163968	WFDC6

chr20	44419569	44419579	WFDC3
chr20	44425097	44425192	DNTTIP1
chr20	44482544	44482623	ACOT8
chr20	44501328	44501464	ZSWIM3
chr20	44600761	44600815	ZNF335
chr20	45005343	45005345	ELMO2
chr20	45010845	45010880	ELMO2
chr20	45214337	45214382	SLC13A3
chr20	45229227	45229255	SLC13A3
chr20	48767836	48767852	TMEM189-UBE2V1
chr20	48767836	48767852	TMEM189
chr20	49457074	49457079	BCAS4
chr20	50713635	50713645	ZFP64
chr20	52557997	52558063	AC005220.3
chr20	52557997	52558063	BCAS1
chr20	52840766	52840776	PFDN4
chr20	54935238	54935312	FAM210B
chr20	55045656	55045715	RTFDC1
chr20	55205417	55205428	TFAP2C
chr20	55800911	55801018	BMP7
chr20	55974984	55975008	RBM38
chr20	56088903	56088928	CTCFL
chr20	56182109	56182356	ZBP1
chr20	56275072	56275242	PMEPA1
chr20	56807967	56807978	PPP4R1L
chr20	56810296	56810430	PPP4R1L
chr20	56811210	56811330	PPP4R1L
chr20	56813267	56813367	PPP4R1L
chr20	56814297	56814458	PPP4R1L
chr20	56814716	56814901	PPP4R1L
chr20	56815571	56815666	PPP4R1L
chr20	56818568	56818761	PPP4R1L
chr20	56820839	56820860	PPP4R1L
chr20	56821014	56821308	PPP4R1L
chr20	56822332	56822562	PPP4R1L
chr20	56823171	56823329	PPP4R1L
chr20	56825943	56826008	PPP4R1L
chr20	56826813	56826920	PPP4R1L
chr20	56846419	56846561	PPP4R1L
chr20	56847825	56847931	PPP4R1L
chr20	56861370	56861502	PPP4R1L
chr20	56884282	56884326	PPP4R1L
chr20	56884475	56884481	PPP4R1L
chr20	57210133	57210420	MGC4294
chr20	57280554	57280607	STX16-NPEPL1
chr20	57280554	57280607	NPEPL1
chr20	60293804	60294226	CDH4

chr20	60293804	60294226	RP11-429E11.3
chr20	60528819	60528884	TAF4
chr20	60917246	60917355	LAMA5
chr20	60920616	60920641	LAMA5
chr20	61273320	61273712	RP11-93B14.6
chr20	61274366	61274407	RP11-93B14.6
chr20	61274366	61274407	SLCO4A1
chr20	61875822	61875895	NKAIN4
chr20	61910572	61910658	ARFGAP1
chr20	61919662	61919883	ARFGAP1
chr20	62042370	62042375	KCNQ2
chr20	62332542	62332740	ARFRP1
chr20	62474779	62475273	AL158091.1
chr20	62585007	62585495	AL118506.1
chr20	62585007	62585495	UCKL1
chr20	62893881	62893906	PCMTD2
chr21	14741931	14741956	AL050302.1
chr21	14743755	14743756	AL050302.1
chr21	14743816	14743916	AL050302.1
chr21	14745372	14745386	AL050302.1
chr21	15051911	15052330	AL050303.1
chr21	18814099	18814161	C21orf37
chr21	18816401	18816518	C21orf37
chr21	18821183	18821226	C21orf37
chr21	19858117	19858126	AL109763.1
chr21	19858117	19858126	TMPRSS15
chr21	27083679	27083775	JAM2
chr21	27318229	27318290	APP
chr21	27937126	27937144	AP001597.1
chr21	27937126	27937144	CYYR1
chr21	28215785	28215859	ADAMTS1
chr21	28216061	28216076	ADAMTS1
chr21	30340293	30340295	LTN1
chr21	30397064	30397098	RP1-100J12.1
chr21	30397064	30397098	USP16
chr21	30954632	30954671	BACH1
chr21	30954632	30954671	GRIK1
chr21	30969890	30970014	GRIK1-AS2
chr21	30969890	30970014	BACH1
chr21	30969890	30970014	GRIK1
chr21	30973548	30973549	GRIK1-AS2
chr21	30973548	30973549	BACH1
chr21	30973548	30973549	GRIK1
chr21	31002859	31002876	GRIK1-AS2
chr21	31002859	31002876	BACH1
chr21	31002859	31002876	GRIK1
chr21	32490861	32490988	TIAM1

chr21	33416415	33416425	HUNK
chr21	33765532	33765717	C21orf119
chr21	33949900	33949986	TCP10L
chr21	33964567	33964728	AP000275.65
chr21	33964567	33964728	C21orf59
chr21	34157189	34157230	C21orf49
chr21	34160923	34161012	C21orf49
chr21	34169249	34169356	C21orf62
chr21	34169249	34169356	C21orf49
chr21	34224059	34224091	C21orf49
chr21	34258240	34258293	C21orf49
chr21	34537977	34537997	C21orf54
chr21	34540748	34540886	C21orf54
chr21	34542001	34542138	C21orf54
chr21	34542446	34542474	C21orf54
chr21	34602828	34602862	IFNAR2
chr21	34624443	34624496	AP000295.9
chr21	34624443	34624496	IFNAR2
chr21	34666317	34666352	IL10RB
chr21	34828322	34828404	IFNGR2
chr21	34828322	34828404	TMEM50B
chr21	34851105	34851110	IFNGR2
chr21	34851105	34851110	TMEM50B
chr21	34901997	34902074	GART
chr21	34941882	34941935	SON
chr21	34941882	34941935	DONSON
chr21	34944857	34944936	SON
chr21	34944857	34944936	DONSON
chr21	34947431	34947451	AP000304.1
chr21	34947431	34947451	SON
chr21	34947431	34947451	DONSON
chr21	34947631	34947645	AP000304.1
chr21	34947631	34947645	SON
chr21	34947631	34947645	DONSON
chr21	34968482	34968519	AP000304.12
chr21	34968482	34968519	CRYZL1
chr21	35201272	35201284	AP000304.12
chr21	35201272	35201284	ITSN1
chr21	35279048	35279098	ATP5O
chr21	35279048	35279098	AP000304.12
chr21	35279123	35279125	ATP5O
chr21	35279123	35279125	AP000304.12
chr21	36228710	36228744	RUNX1
chr21	37270353	37270727	FKSG68
chr21	37270353	37270727	RUNX1
chr21	37402368	37402428	AP000688.1
chr21	37749708	37749805	MORC3

chr21	37752529	37752610	MORC3
chr21	37858193	37858849	AP000695.1
chr21	37858193	37858849	PSMD4P1
chr21	37858193	37858849	AP000695.4
chr21	37858193	37858849	CLDN14
chr21	38118096	38118148	SIM2
chr21	38888740	38888973	AP001421.1
chr21	38888740	38888973	DYRK1A
chr21	39325155	39325308	DSCR4
chr21	39471423	39471432	DSCR4
chr21	39494426	39494505	DSCR8
chr21	39526509	39526623	DSCR8
chr21	39528398	39528496	DSCR8
chr21	40719393	40719409	HMGN1
chr21	40887015	40887086	SH3BGR
chr21	40969631	40969916	C21orf88
chr21	40969631	40969916	B3GALT5
chr21	40977887	40977929	C21orf88
chr21	40977887	40977929	B3GALT5
chr21	40978157	40978274	C21orf88
chr21	40978157	40978274	B3GALT5
chr21	40981510	40981592	C21orf88
chr21	40981510	40981592	B3GALT5
chr21	40984224	40984292	C21orf88
chr21	40984224	40984292	B3GALT5
chr21	41297972	41298006	PCP4
chr21	43506640	43506736	UMODL1
chr21	43513981	43514066	UMODL1
chr21	43521818	43521852	UMODL1
chr21	43528406	43528492	C21orf128
chr21	43528406	43528492	UMODL1
chr21	43816051	43816251	TMPRSS3
chr21	43830225	43830239	UBASH3A
chr21	44579355	44579877	AP001631.10
chr21	44581199	44581362	AP001631.10
chr21	45210897	45210901	RRP1
chr21	45482934	45482941	TRAPPC10
chr21	45565453	45565506	C21orf33
chr21	45587818	45588263	AP001055.1
chr21	45590937	45591047	AP001055.1
chr21	45593517	45593580	AP001055.1
chr21	45879823	45880557	LRRC3DN
chr21	45928752	45928794	TSPEAR-AS1
chr21	45928752	45928794	TSPEAR
chr21	45937677	45937718	C21orf90
chr21	45937677	45937718	TSPEAR
chr21	45938142	45938215	C21orf90

chr21	45938142	45938215	TSPEAR
chr21	45938511	45938592	C21orf90
chr21	45938511	45938592	TSPEAR
chr21	46194601	46194656	UBE2G2
chr21	46199110	46199131	UBE2G2
chr21	46354129	46354283	C21orf67
chr21	46354727	46355011	C21orf67
chr21	46355542	46355805	C21orf67
chr21	46357454	46357519	C21orf67
chr21	46359508	46359582	C21orf67
chr21	46389025	46389179	FAM207A
chr21	46492796	46492927	SSR4P1
chr21	46492796	46492927	AP001579.1
chr21	46511596	46511693	PRED57
chr21	46511596	46511693	ADARB1
chr21	46512040	46512202	PRED57
chr21	46512040	46512202	ADARB1
chr21	46515410	46515493	PRED57
chr21	46515410	46515493	ADARB1
chr21	46520728	46521105	ADARB1
chr21	46520728	46521105	PRED58
chr21	46525754	46525899	ADARB1
chr21	46525754	46525899	PRED58
chr21	46528690	46528753	ADARB1
chr21	46528690	46528753	PRED58
chr21	46534727	46534873	ADARB1
chr21	46534727	46534873	PRED58
chr21	47183565	47183690	PCBP3
chr21	47183565	47183690	PRED60
chr21	47185994	47186037	PCBP3
chr21	47185994	47186037	PRED60
chr21	47187934	47188060	PCBP3
chr21	47187934	47188060	PRED60
chr21	47189949	47190005	PCBP3
chr21	47189949	47190005	PRED60
chr21	47347679	47347697	PRED62
chr21	47347679	47347697	PCBP3
chr21	47351505	47351619	PRED62
chr21	47351505	47351619	PCBP3
chr21	47352373	47352477	PRED62
chr21	47352373	47352477	PCBP3
chr21	47608496	47608735	AP001468.58
chr21	47608496	47608735	LSS
chr21	47612391	47612516	AP001468.1
chr21	47612391	47612516	LSS
chr21	47612518	47613088	AP001468.1
chr21	47612518	47613088	LSS

chr21	47613567	47613673	AP001468.1
chr21	47613567	47613673	LSS
chr22	16258186	16258303	POTEH
chr22	16266929	16267095	POTEH
chr22	16268137	16268181	POTEH
chr22	16269873	16269943	POTEH
chr22	16275207	16275277	POTEH-AS1
chr22	16275207	16275277	POTEH
chr22	16277748	16277885	POTEH-AS1
chr22	16277748	16277885	POTEH
chr22	16279195	16279301	POTEH
chr22	16280431	16280589	POTEH
chr22	16282145	16282318	POTEH
chr22	16282478	16282592	POTEH
chr22	16448824	16449804	OR11H1
chr22	17602819	17602929	AC006946.15
chr22	17603459	17603545	AC006946.15
chr22	17605545	17605661	AC006946.15
chr22	17611252	17611344	AC006946.15
chr22	18029928	18030018	CECR2
chr22	18101800	18101893	ATP6V1E1
chr22	18167375	18167460	BCL2L13
chr22	18276495	18276560	XXbac-B461K10.4
chr22	18276495	18276560	MICAL3
chr22	18628423	18628756	TUBA8
chr22	18721540	18721567	AC008132.1
chr22	18723545	18723603	AC008132.1
chr22	18724072	18724096	AC008132.1
chr22	18727043	18727129	AC008132.1
chr22	18732023	18732088	AC008132.1
chr22	18734087	18734148	AC008132.1
chr22	18734614	18734647	AC008132.1
chr22	18739382	18739404	AC008132.1
chr22	19048275	19048468	DGCR2
chr22	19172017	19172049	CLTCL1
chr22	19245328	19245390	KRT18P62
chr22	19245328	19245390	CLTCL1
chr22	19704801	19704968	5-Sep
chr22	20116995	20117065	ZDHHC8
chr22	20230929	20231207	RTN4R
chr22	20255132	20255212	RTN4R
chr22	20692622	20692649	FAM230A
chr22	20695279	20695337	FAM230A
chr22	20695806	20695830	FAM230A
chr22	20698777	20698863	FAM230A
chr22	20703776	20703841	FAM230A
chr22	20705845	20705906	FAM230A

chr22	20705845	20705906	USP41
chr22	20706372	20706545	FAM230A
chr22	20706372	20706545	USP41
chr22	20708630	20710986	FAM230A
chr22	20708630	20710986	USP41
chr22	20739003	20739029	USP41
chr22	20753885	20753974	ZNF74
chr22	20862560	20862731	MED15
chr22	20873165	20873282	MED15
chr22	20906826	20906869	MED15
chr22	21349657	21349766	LZTR1
chr22	21358030	21358052	THAP7-AS1
chr22	21358030	21358052	AC002472.1
chr22	21358030	21358052	TUBA3FP
chr22	21360568	21360736	THAP7-AS1
chr22	21360568	21360736	AC002472.1
chr22	21360568	21360736	TUBA3FP
chr22	21480537	21481925	POM121L7
chr22	22023623	22023715	PPIL2
chr22	22024588	22024631	PPIL2
chr22	22292576	22292618	LL22NC03-86G7.1
chr22	22292576	22292618	PPM1F
chr22	22325428	22325433	TOP3B
chr22	22569197	22569242	IGLV10-54
chr22	22895230	22895372	PRAME
chr22	22898257	22898347	PRAME
chr22	22901775	22901814	LL22NC03-63E9.3
chr22	22905268	22905377	LL22NC03-63E9.3
chr22	22906072	22906221	LL22NC03-63E9.3
chr22	23466515	23466687	RTDR1
chr22	23466515	23466687	GNAZ
chr22	23473857	23473864	RTDR1
chr22	23585005	23585129	BCR
chr22	24123975	24124048	MMP11
chr22	24209922	24209927	SLC2A11
chr22	24209922	24209927	AP000350.10
chr22	24219572	24219704	SLC2A11
chr22	24219572	24219704	AP000350.10
chr22	24238016	24238100	AP000350.4
chr22	24376423	24376617	GSTT1
chr22	24376822	24376998	GSTT1
chr22	24379361	24379511	GSTT1
chr22	24381700	24381787	GSTT1
chr22	24382973	24383081	GSTT1
chr22	24384120	24384231	GSTT1
chr22	24988343	24988390	SNRPD3
chr22	24988343	24988390	GGT1

chr22	24988343	24988390	FAM211B
chr22	29125309	29125364	CHEK2
chr22	29657293	29657414	RHBDD3
chr22	29657293	29657414	CTA-984G1.5
chr22	29941050	29941133	THOC5
chr22	29960341	29960360	NIPSNAP1
chr22	29976088	29976144	NIPSNAP1
chr22	30217881	30218064	ASCC2
chr22	30223557	30223589	ASCC2
chr22	30229587	30229603	ASCC2
chr22	30230478	30230539	ASCC2
chr22	30234167	30234250	ASCC2
chr22	30683811	30683851	GATSL3
chr22	30683811	30683851	RP1-130H16.18
chr22	30689267	30689308	RP1-130H16.18
chr22	30689267	30689308	TBC1D10A
chr22	30714648	30714655	TBC1D10A
chr22	30793838	30793859	RNF215
chr22	30793838	30793859	SEC14L2
chr22	30794676	30794807	RNF215
chr22	30794676	30794807	SEC14L2
chr22	30814212	30814469	KIAA1658
chr22	30814212	30814469	RP4-539M6.19
chr22	30814212	30814469	RNF215
chr22	30814212	30814469	SEC14L2
chr22	30867163	30867264	SEC14L3
chr22	31006222	31006247	TCN2
chr22	31277668	31277789	OSBP2
chr22	31478471	31478553	RP3-412A9.16
chr22	31478471	31478553	SMTN
chr22	31488454	31488516	SMTN
chr22	31743328	31743723	AC005003.1
chr22	31842967	31842994	DRG1
chr22	31842967	31842994	EIF4ENIF1
chr22	31983314	31983352	SFI1
chr22	32072734	32073004	PRR14L
chr22	32341486	32341518	C22orf24
chr22	32341486	32341518	YWHAH
chr22	32343425	32343472	YWHAH
chr22	32758939	32759045	RFPL3S
chr22	32763677	32763701	RFPL3S
chr22	32764484	32764554	RFPL3S
chr22	32766886	32766913	RFPL3S
chr22	33562749	33562818	LARGE
chr22	35727289	35727388	TOM1
chr22	35797364	35797459	MCM5
chr22	35797659	35797665	MCM5

chr22	36023489	36023706	MB
chr22	36023489	36023706	AL049747.1
chr22	36031002	36031026	MB
chr22	36031002	36031026	AL049747.1
chr22	36545394	36545419	APOL3
chr22	36545840	36546012	APOL3
chr22	36549440	36549471	APOL3
chr22	36550669	36550676	APOL3
chr22	36553041	36553054	APOL3
chr22	36655715	36655956	APOL1
chr22	36864711	36864764	TXN2
chr22	37156961	37156963	IFT27
chr22	37207549	37207568	PVALB
chr22	37319837	37319859	CSF2RB
chr22	37448580	37448672	KCTD17
chr22	37479120	37479126	TMPRSS6
chr22	37706009	37706073	CYTH4
chr22	38044756	38044853	SH3BP1
chr22	38044756	38044853	Z83844.1
chr22	38146649	38146737	NOL12
chr22	38146649	38146737	TRIOBP
chr22	38147148	38147207	NOL12
chr22	38147148	38147207	TRIOBP
chr22	38246531	38246581	EIF3L
chr22	38367249	38367305	SOX10
chr22	38367249	38367305	POLR2F
chr22	38421876	38422154	POLR2F
chr22	38422281	38422317	POLR2F
chr22	38527997	38528118	PLA2G6
chr22	38642865	38642891	TMEM184B
chr22	38694096	38694209	CSNK1E
chr22	39141103	39141150	SUN2
chr22	39141103	39141150	RP3-508I15.14
chr22	39656865	39656884	AL031590.1
chr22	39657045	39657063	AL031590.1
chr22	39715033	39715116	SNORD43
chr22	39715033	39715116	RPL3
chr22	39926531	39926559	RPS19BP1
chr22	39983748	39983756	CACNA1I
chr22	40052423	40052424	CACNA1I
chr22	40052713	40052714	CACNA1I
chr22	40297243	40297334	GRAP2
chr22	41172286	41172312	SLC25A17
chr22	41180517	41180608	SLC25A17
chr22	41195565	41195567	SLC25A17
chr22	41228200	41228220	ST13
chr22	41266052	41266080	XPNPEP3

chr22	41284433	41284477	XPNPEP3
chr22	41303130	41303194	XPNPEP3
chr22	41604319	41604417	L3MBTL2
chr22	41623579	41623601	L3MBTL2
chr22	41673479	41673501	RANGAP1
chr22	41685388	41685407	AL035681.1
chr22	41685668	41685686	AL035681.1
chr22	41778939	41778992	TEF
chr22	41929687	41929691	POLR3H
chr22	42806454	42806497	NFAM1
chr22	42914646	42914734	RRP7A
chr22	43253885	43253887	ARFGAP3
chr22	43253885	43253887	PACSIN2
chr22	43254422	43254528	PACSIN2
chr22	43305527	43305571	PACSIN2
chr22	43466182	43466248	TTLL1
chr22	43814102	43814122	MPPED1
chr22	44223480	44223520	SULT4A1
chr22	44435732	44435837	PARVB
chr22	44761495	44761596	RP1-32I10.10
chr22	44761725	44761850	RP1-32I10.10
chr22	45126684	45126884	PRR5-ARHGAP8
chr22	45126684	45126884	ARHGAP8
chr22	45126684	45126884	PRR5
chr22	45204552	45204612	PRR5-ARHGAP8
chr22	45204552	45204612	ARHGAP8
chr22	45566869	45566892	NUP50
chr22	45603567	45603653	KIAA0930
chr22	45622074	45622264	KIAA0930
chr22	45898118	45898220	FBLN1
chr22	45913473	45913578	FBLN1
chr22	45961846	45961853	FBLN1
chr22	46207578	46207674	ATXN10
chr22	46218998	46219098	ATXN10
chr22	46493807	46493890	FLJ27365
chr22	46494374	46494438	FLJ27365
chr22	46499072	46499129	FLJ27365
chr22	46501289	46501741	FLJ27365
chr22	46505147	46505233	FLJ27365
chr22	46505644	46505890	FLJ27365
chr22	46677868	46677909	TTC38
chr22	46688100	46688145	TTC38
chr22	46844455	46844465	CELSR1
chr22	46858805	46858815	CELSR1
chr22	47066947	47067064	GRAMD4
chr22	47185209	47185323	TBC1D22A
chr22	47857442	47857578	LL22NC03-75H12.2

chr22	47859067	47859137	LL22NC03-75H12.2
chr22	47882479	47882528	LL22NC03-75H12.2
chr22	48935196	48935260	CTA-299D3.8
chr22	48935196	48935260	FAM19A5
chr22	48940484	48940737	CTA-299D3.8
chr22	48940484	48940737	FAM19A5
chr22	48942325	48942395	CTA-299D3.8
chr22	48942325	48942395	FAM19A5
chr22	49246570	49246724	FAM19A5
chr22	49834737	49834861	C22orf34
chr22	50051053	50051107	C22orf34
chr22	50613861	50613898	PANX2
chr22	51048142	51048250	MAPK8IP2
chr22	51062295	51062323	ARSA
chr22	51162555	51162581	SHANK3
chr22	51219134	51219146	RPL23AP82
chr22	51219134	51219146	RABL2B
chrX	334267	334506	PPP2R3B
chrX	1317790	1318008	CRLF2
chrX	1719031	1719036	AKAP17A
chrX	2310508	2310515	DHRSX
chrX	2418352	2418630	ZBED1
chrX	2418352	2418630	DHRSX
chrX	2420802	2420808	DHRSX
chrX	2823059	2823103	ARSD-AS1
chrX	2823059	2823103	ARSD
chrX	2841293	2841347	ARSD
chrX	2976652	2976662	ARSF
chrX	3189874	3189959	CXorf28
chrX	3190338	3190413	CXorf28
chrX	3195027	3195134	CXorf28
chrX	3202196	3202222	CXorf28
chrX	3735816	3735819	RP11-706O15.1
chrX	3736483	3736541	RP11-706O15.1
chrX	3747405	3747433	RP11-706O15.1
chrX	3761382	3761523	RP11-706O15.1
chrX	9050620	9050706	FAM9B
chrX	9754282	9754337	GPR143
chrX	11138115	11138165	HCCS
chrX	11138115	11138165	ARHGAP6
chrX	11166770	11166839	ARHGAP6
chrX	11283990	11284052	ARHGAP6
chrX	11308503	11308598	ARHGAP6
chrX	11369506	11369520	ARHGAP6
chrX	13684435	13684437	TCEANC
chrX	13799034	13799124	GPM6B
chrX	13801898	13802040	GPM6B

chrX	14933556	14933579	MOSPD2
chrX	15253410	15253462	ASB9
chrX	15254650	15254722	ASB9
chrX	15310032	15310149	ASB11
chrX	15785035	15785066	CA5B
chrX	15858670	15858714	AP1S2
chrX	19057380	19057398	GPR64
chrX	19363854	19363937	PDHA1
chrX	19758877	19758886	SH3KBP1
chrX	19765782	19765784	SH3KBP1
chrX	19941517	19941525	CXorf23
chrX	19948668	19948772	CXorf23
chrX	22025264	22025318	SMS
chrX	22116112	22116153	PHEX
chrX	23704119	23704164	PRDX4
chrX	30692419	30692468	GK
chrX	30707589	30707714	GK
chrX	38080579	38080696	RP13-43E11.1
chrX	38080579	38080696	SRPX
chrX	38080579	38080696	TM4SF2
chrX	38138423	38138458	RPGR
chrX	38138423	38138458	TM4SF2
chrX	38157623	38157700	RPGR
chrX	38157623	38157700	TM4SF2
chrX	38422209	38422392	TSPAN7
chrX	38422209	38422392	TM4SF2
chrX	38425575	38425608	TSPAN7
chrX	38425575	38425608	TM4SF2
chrX	38482156	38482212	TSPAN7
chrX	38482156	38482212	TM4SF2
chrX	38482284	38482286	TSPAN7
chrX	38482284	38482286	TM4SF2
chrX	38515249	38515299	TSPAN7
chrX	38515249	38515299	TM4SF2
chrX	40499456	40499572	CXorf38
chrX	40508884	40508924	MED14
chrX	41055158	41055258	USP9X
chrX	41483517	41483534	CASK
chrX	46384813	46384873	ZNF674
chrX	46747025	46747127	CXorf31
chrX	46749728	46749814	CXorf31
chrX	46753959	46754008	CXorf31
chrX	47088770	47088814	CDK16
chrX	47343004	47343288	CXorf24
chrX	47441962	47441989	TIMP1
chrX	47441962	47441989	SYN1
chrX	47916708	47916736	ZNF630

chrX	47916708	47916736	ZNF630-AS1
chrX	48432859	48433248	AC115618.1
chrX	48432859	48433248	RBM3
chrX	48435853	48435947	RBM3
chrX	48753211	48753255	TIMM17B
chrX	48758796	48758820	PQBP1
chrX	49019212	49019276	MAGIX
chrX	49157830	49157922	PPP1R3F
chrX	49189765	49189885	GAGE13
chrX	49192693	49192818	GAGE13
chrX	49195253	49195275	GAGE13
chrX	49204807	49204829	GAGE2E
chrX	49208272	49208352	GAGE2D
chrX	49208817	49208937	GAGE2D
chrX	49211738	49211863	GAGE2D
chrX	49223827	49223849	GAGE12I
chrX	49227294	49227374	GAGE2C
chrX	49227840	49227960	GAGE2C
chrX	49230799	49230924	GAGE2C
chrX	49233365	49233387	GAGE2C
chrX	49236831	49236911	GAGE2B
chrX	49237377	49237497	GAGE2B
chrX	49240334	49240459	GAGE2B
chrX	49242900	49242922	GAGE2B
chrX	49369601	49369689	GAGE1
chrX	51795944	51796063	RP11-114H20.1
chrX	51797091	51797342	RP11-114H20.1
chrX	51942972	51943223	RP11-363G10.2
chrX	51944251	51944370	RP11-363G10.2
chrX	52255307	52255329	XAGE1A
chrX	52257920	52258046	XAGE1A
chrX	52258558	52258715	XAGE1A
chrX	52260122	52260270	XAGE1A
chrX	52380440	52380462	XAGE2
chrX	52383362	52383487	XAGE2
chrX	52385359	52385464	XAGE2
chrX	52385936	52386016	XAGE2
chrX	52511854	52512002	XAGE1C
chrX	52513409	52513566	XAGE1C
chrX	52514078	52514204	XAGE1C
chrX	52516795	52516817	XAGE1C
chrX	52528247	52528269	XAGE1D
chrX	52530860	52530986	XAGE1D
chrX	52531498	52531655	XAGE1D
chrX	52533062	52533210	XAGE1D
chrX	52843213	52843262	XAGE5
chrX	53247823	53247894	KDM5C

chrX	53250901	53250975	KDM5C
chrX	54021573	54021638	PHF8
chrX	56101008	56101235	AL353698.1
chrX	57010903	57010911	SPIN3
chrX	64185333	64185370	ZC4H2
chrX	65390969	65390984	HEPH
chrX	69635184	69635312	KIF4A
chrX	70636468	70636541	TAF1
chrX	70748392	70748401	AL590763.5
chrX	70748392	70748401	TAF1
chrX	70756864	70756960	OGT
chrX	71494277	71494285	RPS4X
chrX	71494277	71494285	PIN4
chrX	71518832	71518849	PIN4
chrX	71700299	71700301	HDAC8
chrX	71709112	71709113	HDAC8
chrX	72347398	72347721	NAP1L6
chrX	74376497	74376532	ABCB7
chrX	74511228	74511270	UPRT
chrX	85439237	85439276	DACH2
chrX	85997646	85997728	DACH2
chrX	91675526	91675538	PCDH11X
chrX	91824799	91824801	PCDH11X
chrX	96048615	96048629	DIAPH2
chrX	100654759	100654788	GLA
chrX	100654759	100654788	RPL36A-HNRNPH2
chrX	100742605	100742678	ARMCX4
chrX	100742995	100743086	ARMCX4
chrX	100753132	100753176	ARMCX4
chrX	101476904	101476921	TCP11X1
chrX	101476904	101476921	NXF2
chrX	101720103	101720120	NXF2B
chrX	101720103	101720120	TCP11X2
chrX	102981543	102981606	GLRA4
chrX	103227464	103227606	TMSB15B
chrX	103274127	103274222	H2BFM
chrX	106059013	106059016	TBC1D8B
chrX	106059013	106059016	MORC4
chrX	106313420	106313655	RBM41
chrX	106367471	106367562	NUP62CL
chrX	106394086	106394197	NUP62CL
chrX	107370023	107370045	ATG4A
chrX	107386937	107387062	ATG4A
chrX	107386937	107387062	COL4A6
chrX	109589931	109590137	GNG5P2
chrX	109589931	109590137	AMMECR1
chrX	114858653	114858692	PLS3

chrX	114874030	114874081	PLS3
chrX	114953262	114953549	RP1-241P17.4
chrX	114953262	114953549	AC005000.1
chrX	117581023	117581077	WDR44
chrX	118820523	118820612	6-Sep
chrX	122318792	122318852	GRIA3
chrX	123160415	123160468	STAG2
chrX	128781653	128781894	APLN
chrX	129144484	129144535	BCORL1
chrX	130212727	130212729	ARHGAP36
chrX	135056059	135056106	MMGT1
chrX	135519163	135519215	GPR112
chrX	138067180	138067246	FGF13
chrX	138814595	138814684	ATP11C
chrX	139791962	139791970	LINC00632
chrX	139793786	139793827	LINC00632
chrX	139795682	139795948	LINC00632
chrX	140096864	140096953	SPANXB1
chrX	140097601	140097822	SPANXB1
chrX	140968647	140968650	MAGEC3
chrX	140982426	140982586	MAGEC3
chrX	148573139	148573198	IDS
chrX	148573139	148573198	IDS
chrX	148583628	148583707	IDS
chrX	148583628	148583707	IDS
chrX	148675471	148675569	HSFX2
chrX	150873740	150873872	PRRG3
chrX	151125930	151125958	GABRE
chrX	151127436	151127621	GABRE
chrX	151139714	151139789	GABRE
chrX	152244152	152245351	PNMA6D
chrX	152244152	152245351	PNMA6A
chrX	152338908	152340107	PNMA6A
chrX	152341614	152342813	PNMA6B
chrX	152609656	152609664	ZNF275
chrX	152610129	152610237	ZNF275
chrX	152720335	152720511	TREX2
chrX	152720335	152720511	HAUS7
chrX	152935272	152935321	PNCK
chrX	153000798	153000881	ABCD1
chrX	153054569	153054655	IDH3G
chrX	153146127	153146173	LCA10
chrX	153146127	153146173	L1CAM
chrX	153149166	153149351	LCA10
chrX	153149166	153149351	L1CAM
chrX	153149403	153149727	LCA10
chrX	153149403	153149727	L1CAM

chrX	153150817	153150890	LCA10
chrX	153150817	153150890	L1CAM
chrX	153151262	153151431	LCA10
chrX	153151262	153151431	L1CAM
chrX	153152417	153152511	LCA10
chrX	153152417	153152511	L1CAM
chrX	153153951	153154051	LCA10
chrX	153153951	153154051	L1CAM
chrX	153201457	153201558	RENBP
chrX	153202621	153202641	RENBP
chrX	153276391	153276567	IRAK1
chrX	153424832	153425188	TEX28P2
chrX	153425808	153425943	TEX28P2
chrX	153441110	153441837	TEX28P2
chrX	153445535	153445546	TEX28P2
chrX	153461962	153462318	TEX28P1
chrX	153462938	153463073	TEX28P1
chrX	153482656	153482667	TEX28P1
chrX	153516035	153516762	TEX28
chrX	153518086	153518106	TEX28
chrX	153637435	153637488	RPL10
chrX	153637435	153637488	DNASE1L1
chrX	153666562	153666657	GDI1
chrX	153907255	153907616	GAB3
chrX	154032998	154033102	MPP1
chrX	154275644	154275666	FUNDC2

Chromosome	Start Position	End Position	Gene	Agilent V7
chr1	138530	139309	AL627309.1	
chr1	738532	738618	AL669831.1	
chr1	738532	738618	RP11-206L10.9	
chr1	738532	738618	RP11-206L10.8	
chr1	738788	738812	AL669831.1	
chr1	738788	738812	RP11-206L10.9	
chr1	738788	738812	RP11-206L10.8	
chr1	739121	739137	AL669831.1	
chr1	739121	739137	RP11-206L10.9	
chr1	739121	739137	RP11-206L10.8	
chr1	818043	818058	AL645608.2	
chr1	819496	819513	AL645608.2	
chr1	819961	819983	AL645608.2	
chr1	863255	863261	AL645608.1	
chr1	863255	863261	SAMD11	
chr1	865990	865996	AL645608.1	
chr1	865990	865996	SAMD11	
chr1	1020631	1020690	C1orf159	
chr1	1156079	1156110	SDF4	
chr1	1334932	1335069	RP4-758J18.2	
chr1	1335361	1335509	RP4-758J18.2	
chr1	1335538	1335687	RP4-758J18.2	
chr1	1335985	1336114	RP4-758J18.2	
chr1	1418427	1418486	ATAD3B	
chr1	1510357	1510659	AL645728.1	
chr1	2121490	2122032	AL590822.2	
chr1	2121490	2122032	C1orf86	
chr1	2144366	2145019	AL590822.1	
chr1	2144366	2145019	RP11-181G12.4	
chr1	2258581	2259042	AL589739.1	
chr1	2258581	2259042	MORN1	
chr1	3311056	3311058	PRDM16	
chr1	6101389	6101399	AL035406.1	
chr1	6101389	6101399	KCNAB2	
chr1	6156181	6156186	KCNAB2	
chr1	6366923	6366942	ACOT7	
chr1	9109159	9109274	SLC2A5	
chr1	9713837	9714101	C1orf200	
chr1	9713837	9714101	PIK3CD	
chr1	9714256	9714494	C1orf200	
chr1	9714256	9714494	PIK3CD	
chr1	9778196	9778216	PIK3CD	
chr1	12469924	12469947	VPS13D	
chr1	12538855	12538928	VPS13D	
chr1	16555368	16555558	C1orf134	
chr1	16555978	16556038	C1orf134	

chr1	17664217	17664284	PADI4
chr1	17664217	17664284	AC004824.2
chr1	17669346	17669388	PADI4
chr1	17669346	17669388	AC004824.2
chr1	17669394	17669415	PADI4
chr1	17669394	17669415	AC004824.2
chr1	17675982	17676070	PADI4
chr1	17675982	17676070	AC004824.2
chr1	17966327	17966348	ARHGEF10L
chr1	18605339	18605378	IGSF21
chr1	22215161	22215214	HSPG2
chr1	22263648	22263710	HSPG2
chr1	23695791	23696171	C1orf213
chr1	23695791	23696171	ZNF436
chr1	23697632	23697706	C1orf213
chr1	23697967	23698000	C1orf213
chr1	23698006	23698015	C1orf213
chr1	24980797	24980834	SRRM1
chr1	25255531	25255533	RUNX3
chr1	26146744	26147250	AL020996.1
chr1	26146744	26147250	RP1-317E23.6
chr1	26146744	26147250	MTFR1L
chr1	28527013	28527030	AL353354.1
chr1	28527013	28527030	DNAJC8
chr1	28527105	28527152	AL353354.1
chr1	28527105	28527152	AL353354.2
chr1	28527105	28527152	DNAJC8
chr1	28527229	28527375	AL353354.2
chr1	28527229	28527375	DNAJC8
chr1	31733818	31734023	SNRNP40
chr1	32045889	32046339	TINAGL1
chr1	32100447	32100544	PEF1
chr1	32608607	32608625	KPNA6
chr1	33299635	33299639	S100PBP
chr1	33439268	33439642	FKSG48
chr1	33791426	33791562	RP11-415J8.3
chr1	33791426	33791562	PHC2
chr1	36643159	36643175	MAP7D1
chr1	36793435	36793758	RP11-268J15.5
chr1	38292232	38292262	AL929472.1
chr1	38292232	38292262	MTF1
chr1	38293873	38293886	AL929472.1
chr1	38293873	38293886	MTF1
chr1	39391626	39391701	RHBDL2
chr1	41447659	41447666	CTPS1
chr1	43008460	43008501	CCDC30
chr1	43262231	43262445	C1orf50

chr1	45406301	45406438	EIF2B3
chr1	45879506	45879542	TESK2
chr1	48226979	48227290	RP11-543D5.2
chr1	48226979	48227290	FLJ00388
chr1	48226979	48227290	TRABD2B
chr1	50460708	50461031	AL645730.2
chr1	50460708	50461031	AGBL4
chr1	51078003	51078020	FAF1
chr1	51888880	51888950	EPS15
chr1	52260845	52260880	RP4-657D16.3
chr1	52260845	52260880	NRD1
chr1	54356224	54356249	YIPF1
chr1	54636764	54637144	AL357673.1
chr1	54636764	54637144	RP11-446E24.4
chr1	54703740	54704042	SSBP3-AS1
chr1	54703740	54704042	SSBP3
chr1	55516865	55516959	PCSK9
chr1	60474320	60474413	C1orf87
chr1	63073438	63073806	AL138847.1
chr1	63073438	63073806	DOCK7
chr1	64014979	64015044	DLEU2L
chr1	64014979	64015044	EFCAB7
chr1	64014979	64015044	ITGB3BP
chr1	64015160	64015261	DLEU2L
chr1	64015160	64015261	EFCAB7
chr1	64015160	64015261	ITGB3BP
chr1	67131894	67132439	AL139147.1
chr1	67131894	67132439	SGIP1
chr1	67474070	67474130	SLC35D1
chr1	70696240	70696298	SRSF11
chr1	71349632	71349766	PTGER3
chr1	71351961	71352011	PTGER3
chr1	78389895	78389927	NEXN
chr1	84810519	84810653	RP11-376N17.4
chr1	84810519	84810653	SAMD13
chr1	85096297	85096570	C1orf180
chr1	85097293	85097390	C1orf180
chr1	85930179	85930202	DDAH1
chr1	89246838	89246844	PKN2
chr1	92632542	92632697	KIAA1107
chr1	94484130	94484135	ABCA4
chr1	94511278	94511521	ABCA4
chr1	94921289	94921360	ABCD3
chr1	96457908	96458065	RP11-147C23.1
chr1	96461677	96461750	RP11-147C23.1
chr1	96488089	96488171	RP11-147C23.1
chr1	98165760	98165858	DPYD

chr1	107946262	107946267	NTNG1
chr1	108996669	108996675	NBPF6
chr1	113229588	113229621	MOV10
chr1	144825783	144825788	NBPF9
chr1	144989641	144990021	AL590452.1
chr1	144989641	144990021	PDE4DIP
chr1	145017977	145018002	PDE4DIP
chr1	145074964	145075060	PDE4DIP
chr1	145313334	145313506	RP11-458D21.5
chr1	145313334	145313506	NBPF10
chr1	145318054	145318226	RP11-458D21.5
chr1	145318054	145318226	NBPF10
chr1	145322772	145322944	NBPF10
chr1	145327493	145327665	NBPF10
chr1	145328375	145328432	NBPF10
chr1	145332223	145332395	NBPF10
chr1	145334684	145334792	NBPF10
chr1	145336929	145337101	NBPF10
chr1	145341649	145341821	NBPF10
chr1	145344128	145344236	NBPF10
chr1	145351103	145351275	NBPF10
chr1	145355837	145356009	NBPF10
chr1	145357417	145357589	NBPF10
chr1	145358304	145358412	NBPF10
chr1	145362097	145362269	NBPF10
chr1	145363679	145363851	NBPF10
chr1	145364566	145364674	NBPF10
chr1	145366179	145366230	NBPF10
chr1	145366827	145366999	NBPF10
chr1	145648016	145648022	RNF115
chr1	147631967	147632428	BX842679.1
chr1	147631967	147632428	NBPF24
chr1	148591201	148591373	NBPF15
chr1	149765520	149765643	HIST2H2BF
chr1	149765520	149765643	RP11-196G18.21
chr1	150070952	150070970	VPS45
chr1	150522734	150522752	RP11-54A4.2
chr1	150522734	150522752	ADAMTSL4
chr1	150522734	150522752	AL356356.1
chr1	150523091	150523536	RP11-54A4.2
chr1	150523091	150523536	ADAMTSL4
chr1	150523091	150523536	AL356356.1
chr1	150533557	150533946	ADAMTSL4-AS1
chr1	151252672	151252734	RP11-126K1.2
chr1	151254019	151254135	RP11-126K1.2
chr1	151254019	151254135	ZNF687
chr1	151682969	151683382	CELF3

chr1	151682969	151683382	RIIAD1
chr1	151682969	151683382	AL589765.1
chr1	151803404	151803605	RORC
chr1	153513861	153513900	S100A5
chr1	154301464	154301551	ATP8B2
chr1	154585067	154585080	AL606500.1
chr1	154585067	154585080	ADAR
chr1	154585184	154585190	AL606500.1
chr1	154585184	154585190	ADAR
chr1	154600331	154600474	ADAR
chr1	155942046	155942111	ARHGEF2
chr1	156219529	156219745	SMG5
chr1	157516003	157516070	FCRL5
chr1	158323265	158323316	CD1E
chr1	159879884	159880159	AL590560.1
chr1	160585526	160585558	SLAMF1
chr1	164853779	164853799	PBX1
chr1	166028072	166028467	AL626787.1
chr1	166028072	166028467	FAM78B
chr1	167356418	167356438	POU2F1
chr1	169079869	169079941	ATP1B1
chr1	169795234	169795258	C1orf112
chr1	171107351	171107545	FMO6P
chr1	171112310	171112498	FMO6P
chr1	171115496	171115658	FMO6P
chr1	171116765	171116907	FMO6P
chr1	171118699	171118898	FMO6P
chr1	171121049	171121404	FMO6P
chr1	171123281	171123353	FMO6P
chr1	171130202	171130204	FMO6P
chr1	171558508	171558744	PRRC2C
chr1	178494506	178494603	TEX35
chr1	178499256	178499312	TEX35
chr1	178500667	178500693	TEX35
chr1	178514056	178514163	C1orf220
chr1	178514056	178514163	C1ORF220
chr1	178514056	178514163	TEX35
chr1	178514348	178514560	C1orf220
chr1	178514348	178514560	C1ORF220
chr1	178514348	178514560	TEX35
chr1	178514615	178515019	C1orf220
chr1	178514615	178515019	C1ORF220
chr1	178514615	178515019	TEX35
chr1	179418206	179418348	HNRNPA1P54
chr1	179418206	179418348	AXDND1
chr1	179443716	179443776	AL160286.1
chr1	179443716	179443776	AXDND1

chr1	179457738	179457805	AL160286.1
chr1	179457738	179457805	AXDND1
chr1	180941695	180941705	RP11-46A10.5
chr1	180941695	180941705	AL162431.1
chr1	180949712	180949868	AL162431.1
chr1	180949712	180949868	STX6
chr1	181714484	181714495	CACNA1E
chr1	181748579	181748675	CACNA1E
chr1	182778320	182778419	NPL
chr1	185275617	185275619	IVNS1ABP
chr1	197886489	197886672	LHX9
chr1	202794635	202795252	RP11-480I12.4
chr1	204527769	204527809	MDM4
chr1	204915440	204915457	NFASC
chr1	204915838	204915852	RP11-494K3.2
chr1	204915838	204915852	NFASC
chr1	204962889	204962899	NFASC
chr1	205325661	205325697	KLHDC8A
chr1	205683471	205683719	AC119673.1
chr1	205683471	205683719	NUCKS1
chr1	206670927	206671050	C1orf147
chr1	207268636	207268913	C4BPB
chr1	207520477	207520529	CD55
chr1	207523483	207523496	CD55
chr1	210330224	210330225	SYT14
chr1	210804341	210804507	HHAT
chr1	213146766	213146783	VASH2
chr1	215803531	215803602	USH2A
chr1	218683438	218683529	C1orf143
chr1	218696695	218696792	C1orf143
chr1	218698749	218698870	C1orf143
chr1	220603286	220603370	AC096644.1
chr1	220607683	220607710	AC096644.1
chr1	220607984	220608023	AC096644.1
chr1	222821281	222821296	MIA3
chr1	222886086	222886123	AIDA
chr1	222886086	222886123	BROX
chr1	223152964	223153009	DISP1
chr1	223532400	223532463	SUSD4
chr1	223533545	223533653	SUSD4
chr1	224215760	224215846	AC138393.1
chr1	224216016	224216040	AC138393.1
chr1	224216349	224216371	AC138393.1
chr1	224490642	224490723	NVL
chr1	225601576	225601923	AC092811.1
chr1	225601576	225601923	LBR
chr1	228247903	228248005	WNT3A

chr1	228351818	228352150	IBA57-AS1
chr1	229463283	229463305	CCSAP
chr1	229634941	229634967	NUP133
chr1	230498019	230498123	PGBD5
chr1	230503699	230503890	PGBD5
chr1	231353591	231353594	TRIM67
chr1	231355670	231355698	TRIM67
chr1	233337896	233337996	PCNXL2
chr1	233767027	233767033	KCNK1
chr1	236974945	236974964	MTR
chr1	237430900	237430933	RYR2
chr1	237496916	237496926	RYR2
chr1	237516141	237516155	RYR2
chr1	237906900	237906923	RYR2
chr1	238107552	238107626	MTRNR2L11
chr1	240629183	240629225	AL646016.1
chr1	240629183	240629225	FMN2
chr1	240643479	240643504	AL646016.1
chr1	241779156	241779238	OPN3
chr1	243388058	243388102	AC092782.1
chr1	243388058	243388102	CEP170
chr1	243395894	243395911	AC092782.1
chr1	243395894	243395911	CEP170
chr1	243481339	243481353	SDCCAG8
chr1	244227916	244228404	AL590483.1
chr1	247690258	247690356	GCSAML
chr1	247701978	247702093	GCSAML
chr1	249208751	249208758	PGBD2
chr2	3393457	3393550	TRAPPC12
chr2	6112057	6112536	DKFZP761K2322
chr2	6112057	6112536	LINC01105
chr2	7019148	7019172	RSAD2
chr2	10085341	10085353	GRHL1
chr2	10223908	10224072	AC104794.4
chr2	10225272	10225336	AC104794.4
chr2	10226147	10226228	AC104794.4
chr2	10994493	10994726	AC092687.4
chr2	10994989	10995036	AC092687.4
chr2	11603927	11603937	AC099344.1
chr2	11603927	11603937	E2F6
chr2	11606250	11606274	AC099344.1
chr2	11606250	11606274	E2F6
chr2	14775327	14775768	FAM84A
chr2	14775327	14775768	AC011897.1
chr2	14789440	14789447	FAM84A
chr2	14789440	14789447	AC011897.1
chr2	15840525	15840659	AC008271.1

chr2	15841353	15841499	AC008271.1
chr2	15883613	15883647	AC008271.1
chr2	15884350	15884396	AC008271.1
chr2	23729913	23730102	AC011239.1
chr2	23729913	23730102	KLHL29
chr2	23746821	23746961	AC011239.1
chr2	23746821	23746961	KLHL29
chr2	24079279	24079312	ATAD2B
chr2	25622315	25622335	DTNB
chr2	26251074	26251481	AC013449.1
chr2	26671229	26671292	DRC1
chr2	27930448	27931032	AC074091.13
chr2	27998851	27998862	AC110084.1
chr2	27998851	27998862	MRPL33
chr2	28009122	28009148	AC110084.1
chr2	28009122	28009148	MRPL33
chr2	28009122	28009148	RBKS
chr2	28009163	28009186	AC110084.1
chr2	28009163	28009186	MRPL33
chr2	28009163	28009186	RBKS
chr2	29152924	29153015	WDR43
chr2	29430910	29430912	ALK
chr2	36759001	36759164	AC007401.2
chr2	36759001	36759164	CRIM1
chr2	37068327	37068530	AC007382.1
chr2	38907202	38907291	GALM
chr2	39261958	39261999	SOS1
chr2	40403117	40403126	SLC8A1-AS1
chr2	40403117	40403126	SLC8A1
chr2	40488147	40488178	AC007377.1
chr2	40488147	40488178	SLC8A1
chr2	40490099	40490117	AC007377.1
chr2	40490099	40490117	SLC8A1
chr2	40564465	40564468	SLC8A1
chr2	43393800	43393905	THADA
chr2	43657785	43657789	THADA
chr2	47043964	47044051	LINC01118
chr2	47043964	47044051	LINC01119
chr2	47044387	47044572	LINC01118
chr2	47044387	47044572	LINC01119
chr2	47045319	47045370	LINC01118
chr2	47045319	47045370	LINC01119
chr2	47049240	47049297	LINC01118
chr2	47049240	47049297	LINC01119
chr2	47083062	47083129	LINC01119
chr2	47085225	47085349	LINC01119
chr2	47799674	47800126	AC138655.1

chr2	48064127	48064175	FBXO11
chr2	50246375	50246411	NRXN1
chr2	50966402	50966408	NRXN1
chr2	51125736	51125740	NRXN1
chr2	51150542	51150553	NRXN1
chr2	51206409	51206413	NRXN1
chr2	54144396	54144413	PSME4
chr2	62282575	62282592	COMMD1
chr2	65332530	65332537	RAB1A
chr2	65333510	65333524	RAB1A
chr2	66672325	66672428	MEIS1
chr2	66681841	66681920	MEIS1
chr2	66723158	66723304	MEIS1
chr2	66736059	66736255	MEIS1
chr2	66789214	66789251	MEIS1
chr2	70129482	70129603	AC019206.1
chr2	70129482	70129603	MXD1
chr2	70129482	70129603	SNRNP27
chr2	70129772	70129841	AC019206.1
chr2	70129772	70129841	MXD1
chr2	70129772	70129841	SNRNP27
chr2	71602440	71602465	ZNF638
chr2	71635829	71635909	ZNF638
chr2	73497951	73498043	FBXO41
chr2	73689923	73689937	ALMS1
chr2	74159725	74159771	DGUOK
chr2	74361811	74361906	MGC10955
chr2	74418517	74418627	CATX-2
chr2	86078714	86078826	ST3GAL5
chr2	86079983	86080214	ST3GAL5
chr2	86507144	86507185	REEP1
chr2	87092511	87092525	AC111200.1
chr2	87092795	87092827	AC111200.1
chr2	90458201	90458249	CH17-132F21.1
chr2	90458374	90458671	CH17-132F21.1
chr2	95538562	95538741	TEKT4
chr2	95538562	95538741	AC097374.2
chr2	96514587	96514602	ANKRD36C
chr2	96541715	96541725	ANKRD36C
chr2	97259000	97259027	KANSL3
chr2	97281258	97281281	KANSL3
chr2	97643554	97643669	FAM178B
chr2	97654025	97654061	FAM178B
chr2	97674734	97674921	FAM178B
chr2	97684092	97684175	AC079395.1
chr2	97684092	97684175	FAM178B
chr2	98341333	98341356	ZAP70

chr2	98948162	98948212	AC092675.3
chr2	98949677	98949767	AC092675.3
chr2	98949904	98949966	AC092675.3
chr2	98950386	98950414	AC092675.3
chr2	100986705	100987007	AC012493.2
chr2	102090664	102090737	RFX8
chr2	102413726	102413845	MAP4K4
chr2	102509223	102509804	FLJ20373
chr2	102509223	102509804	MAP4K4
chr2	105867759	105867773	AC012360.1
chr2	105869587	105869601	AC012360.1
chr2	105882490	105882585	AC012360.2
chr2	105882490	105882585	TGFBRAP1
chr2	107029217	107029251	RGPD3
chr2	107099728	107099749	AC108868.1
chr2	107103826	107103848	AC108868.1
chr2	107103826	107103848	CD8BP
chr2	108499618	108499652	RGPD4
chr2	114204992	114205429	AC016745.1
chr2	114204992	114205429	CBWD2
chr2	121223153	121223686	LINC01101
chr2	122227842	122227853	CLASP1
chr2	122247849	122247864	CLASP1
chr2	127659445	127659593	AC114783.1
chr2	128176023	128176068	PROC
chr2	130915757	130915838	SMPD4
chr2	131328413	131328553	AC140481.2
chr2	132044682	132045038	CYP4F31P
chr2	132044682	132045038	PLEKHB2
chr2	133875457	133875834	AC011755.1
chr2	133875457	133875834	NCKAP5
chr2	138237045	138237053	THSD7B
chr2	145188105	145188137	ZEB2
chr2	149791569	149791574	KIF5C
chr2	149804457	149804474	KIF5C
chr2	150016990	150016994	LYPD6B
chr2	151857842	151857904	AC023469.2
chr2	151857842	151857904	AC023469.1
chr2	151890323	151890405	AC023469.1
chr2	151900283	151900387	AC023469.1
chr2	151904193	151904217	AC023469.1
chr2	157188992	157189022	NR4A2
chr2	161138768	161138815	RBMS1
chr2	170821380	170821476	UBR3
chr2	171570141	171570953	LINC01124
chr2	172560844	172560883	AC068039.1
chr2	172560844	172560883	DYNC1I2

chr2	172567289	172567314	AC068039.1
chr2	172567289	172567314	DYNC1I2
chr2	175234715	175234742	CIR1
chr2	176796880	176796890	KIAA1715
chr2	187361840	187361857	AC018867.1
chr2	187361840	187361857	ZC3H15
chr2	187365355	187365393	AC018867.1
chr2	187365355	187365393	ZC3H15
chr2	190642909	190642936	AC013468.1
chr2	190642909	190642936	ORMDL1
chr2	190643138	190643157	AC013468.1
chr2	190643138	190643157	ORMDL1
chr2	198012593	198012628	ANKRD44
chr2	198557835	198557940	AC011997.1
chr2	198563534	198563588	AC011997.1
chr2	198638740	198638781	BOLL
chr2	198638740	198638781	AC011997.1
chr2	202526174	202526187	MPP4
chr2	202526278	202526294	MPP4
chr2	202941655	202941822	AC079354.1
chr2	202990551	202990624	AC079354.1
chr2	203104292	203104435	AC079354.2
chr2	208436458	208436492	CREB1
chr2	214261315	214261416	SPAG16
chr2	215595632	215595647	BARD1
chr2	216212344	216212361	ATIC
chr2	216213407	216213454	ATIC
chr2	217735570	217735703	AC007563.1
chr2	217735570	217735703	AC007557.1
chr2	217735570	217735703	AC007563.5
chr2	217736179	217736338	AC007563.1
chr2	217736179	217736338	AC007557.1
chr2	217736179	217736338	AC007563.5
chr2	223785672	223785786	ACSL3
chr2	231334166	231334167	SP100
chr2	232317248	232317521	AC017104.2
chr2	232317795	232317864	AC017104.2
chr2	232571517	232571621	MGC4771
chr2	232571517	232571621	PTMA
chr2	233755644	233755690	NGEF
chr2	233770355	233770559	NGEF
chr2	233877562	233877679	AC106876.2
chr2	233877562	233877679	NGEF
chr2	233877850	233877957	AC106876.2
chr2	233877850	233877957	NGEF
chr2	233880417	233880451	AC106876.2
chr2	233998690	233998706	INPP5D

chr2	233998840	233998870	INPP5D
chr2	234053919	234053927	INPP5D
chr2	234252276	234252283	SAG
chr2	236682576	236682626	AC064874.1
chr2	236682576	236682626	AGAP1
chr2	236682947	236683066	AC064874.1
chr2	236682947	236683066	AGAP1
chr2	236685601	236685685	AC064874.1
chr2	236685601	236685685	AGAP1
chr2	236691958	236692031	AC064874.1
chr2	236691958	236692031	AGAP1
chr2	236954581	236954675	AGAP1
chr2	238165759	238165836	AC112715.2
chr2	238166073	238166177	AC112715.2
chr2	238330183	238330483	AC112721.1
chr2	238330768	238330787	AC112721.1
chr2	238454100	238454216	MLPH
chr2	238499812	238499910	RAB17
chr2	238499812	238499910	AC104667.3
chr2	238500515	238500674	RAB17
chr2	238500515	238500674	AC104667.3
chr2	238503583	238503588	RAB17
chr2	238503583	238503588	AC104667.3
chr2	239133774	239134140	AC016757.3
chr2	239136325	239136439	AC016757.3
chr2	239139842	239139860	AC016757.3
chr2	239192329	239192351	AC012485.1
chr2	239192329	239192351	PER2
chr2	239192447	239192498	AC012485.1
chr2	239192447	239192498	PER2
chr2	240323442	240323663	AC062017.1
chr2	240323769	240323940	AC062017.1
chr2	240500120	240500618	AC079612.1
chr2	240504713	240504804	AC079612.1
chr2	240684580	240684626	AC093802.1
chr2	240701998	240702119	AC093802.1
chr2	240721535	240721542	AC093802.1
chr2	241053907	241053937	AC013469.1
chr2	241064285	241064304	AC013469.1
chr2	241389247	241389338	AC110619.2
chr2	241389247	241389338	GPC1
chr2	241390028	241390339	AC110619.2
chr2	241390028	241390339	AC110619.1
chr2	241390028	241390339	GPC1
chr2	241390558	241390748	AC110619.2
chr2	241390558	241390748	AC110619.1
chr2	241390558	241390748	GPC1

chr2	241392228	241392511	AC110619.2
chr2	241392228	241392511	GPC1
chr2	241396014	241396080	AC110619.2
chr2	241396014	241396080	GPC1
chr2	241396851	241396886	GPC1
chr2	241397382	241397534	GPC1
chr2	241471493	241471552	ANKMY1
chr2	241624478	241624705	AC011298.1
chr2	241869102	241869113	AC104809.3
chr2	242688714	242688742	D2HGDH
chr2	242836364	242836490	AC131097.4
chr2	242836364	242836490	AC131097.3
chr2	242839337	242839485	AC131097.4
chr2	242839337	242839485	AC131097.3
chr2	242841266	242841289	AC131097.4
chr2	242841266	242841289	AC131097.3
chr2	242842509	242842520	AC131097.4
chr2	242842509	242842520	AC131097.3
chr3	4867430	4867546	AC018816.3
chr3	4867430	4867546	ITPR1
chr3	4872631	4872702	AC018816.3
chr3	4872631	4872702	ITPR1
chr3	4927407	4927450	AC018816.3
chr3	11734606	11734637	VGLL4
chr3	13028471	13028536	IQSEC1
chr3	13573971	13574007	FBLN2
chr3	14486246	14486355	SLC6A6
chr3	15509275	15509416	COLQ
chr3	19490420	19490503	KCNH8
chr3	25900180	25900289	LINC00692
chr3	25902182	25902243	LINC00692
chr3	25904479	25904544	LINC00692
chr3	25904776	25904870	LINC00692
chr3	38583258	38583437	EXOG
chr3	39448319	39448573	RPSA
chr3	46599303	46599818	LUZPP1
chr3	46599303	46599818	LRRC2
chr3	46927699	46927728	AC109583.1
chr3	46927699	46927728	PTH1R
chr3	46930133	46930171	AC109583.1
chr3	46930133	46930171	PTH1R
chr3	48701364	48701567	RP11-148G20.1
chr3	48701364	48701567	NCKIPSD
chr3	49265751	49265776	CCDC36
chr3	49298046	49298486	RP11-3B7.1
chr3	49410673	49410775	RHOA
chr3	49941498	49941523	CTD-2330K9.3

chr3	49953875	49954046	CTD-2330K9.3
chr3	49953875	49954046	MON1A
chr3	50330324	50330349	IFRD2
chr3	50330324	50330349	HYAL3
chr3	52097076	52097567	LINC00696
chr3	53825008	53825028	CACNA1D
chr3	53830368	53830427	CACNA1D
chr3	56603341	56603390	CCDC66
chr3	57176481	57176534	IL17RD
chr3	59957215	59957520	NPCDR1
chr3	59957215	59957520	FHIT
chr3	59957569	59957583	NPCDR1
chr3	59957569	59957583	FHIT
chr3	62536520	62536564	CADPS
chr3	63831862	63831893	C3orf49
chr3	63831862	63831893	THOC7
chr3	69277531	69277542	FRMD4B
chr3	69277739	69277749	FRMD4B
chr3	97651956	97651967	CRYBG3
chr3	101481953	101482030	CEP97
chr3	105468568	105468609	CBLB
chr3	110607766	110607773	RP11-553A10.1
chr3	110611076	110611450	RP11-553A10.1
chr3	110611985	110612209	RP11-553A10.1
chr3	113631920	113632007	GRAMD1C
chr3	117716029	117716095	LSAMP
chr3	117716029	117716095	RP11-384F7.2
chr3	122477619	122477700	HSPBAP1
chr3	123671442	123671457	CCDC14
chr3	124223750	124223797	KALRN
chr3	124396411	124396454	KALRN
chr3	128292123	128292572	C3orf27
chr3	130365223	130365235	COL6A6
chr3	141145005	141145007	ZBTB38
chr3	142217972	142218034	ATR
chr3	145782380	145782562	AC107021.1
chr3	145782380	145782562	RP11-274H2.2
chr3	149481732	149481776	ANKUB1
chr3	149689067	149689528	AC117395.1
chr3	149689067	149689528	PFN2
chr3	150588832	150588974	CLRN1-AS1
chr3	150588832	150588974	FAM188B2
chr3	150590974	150591154	CLRN1-AS1
chr3	150590974	150591154	FAM188B2
chr3	150600684	150600845	CLRN1-AS1
chr3	150600684	150600845	FAM188B2
chr3	150600684	150600845	RP11-166N6.3

chr3	150601487	150601559	CLRN1-AS1
chr3	150601487	150601559	FAM188B2
chr3	150601487	150601559	RP11-166N6.3
chr3	150603155	150603225	CLRN1-AS1
chr3	150603155	150603225	FAM188B2
chr3	150603155	150603225	RP11-166N6.3
chr3	150608107	150608172	CLRN1-AS1
chr3	150608107	150608172	FAM188B2
chr3	150608107	150608172	RP11-166N6.3
chr3	150608725	150608890	CLRN1-AS1
chr3	150608725	150608890	FAM188B2
chr3	150608725	150608890	RP11-166N6.3
chr3	150608725	150608890	RP11-166N6.2
chr3	150611111	150611202	CLRN1-AS1
chr3	150611111	150611202	FAM188B2
chr3	150611111	150611202	RP11-166N6.3
chr3	150611111	150611202	RP11-166N6.2
chr3	155461005	155461391	AC104472.1
chr3	155461005	155461391	PLCH1
chr3	159733853	159733867	LINC01100
chr3	159733853	159733867	IL12A-AS1
chr3	159736924	159736985	LINC01100
chr3	159736924	159736985	IL12A-AS1
chr3	159738433	159738531	LINC01100
chr3	159738433	159738531	IL12A-AS1
chr3	159744397	159744605	LINC01100
chr3	159744397	159744605	IL12A-AS1
chr3	167351197	167351320	WDR49
chr3	169868395	169868497	PHC3
chr3	171852151	171852172	FNDC3B
chr3	172019438	172019686	AC092964.2
chr3	172019438	172019686	FNDC3B
chr3	172034218	172034233	AC092964.1
chr3	172034218	172034233	FNDC3B
chr3	172034972	172035032	AC092964.1
chr3	172034972	172035032	FNDC3B
chr3	172039389	172039416	AC092964.1
chr3	172039389	172039416	FNDC3B
chr3	172039421	172039513	AC092964.1
chr3	172039421	172039513	FNDC3B
chr3	172361483	172361740	AC007919.2
chr3	172361483	172361740	NCEH1
chr3	180397396	180397587	CCDC39
chr3	180426556	180426613	CCDC39
chr3	185431216	185431276	C3orf65
chr3	185431216	185431276	IGF2BP2
chr3	185434229	185434599	C3orf65

chr3	185434229	185434599	IGF2BP2
chr3	185439907	185440046	C3orf65
chr3	185439907	185440046	IGF2BP2
chr3	185826246	185826297	ETV5
chr3	185826246	185826297	DGKG
chr3	187420318	187420327	RTP2
chr3	187420318	187420327	RP11-211G3.3
chr3	187433410	187433528	RP11-211G3.3
chr3	187450159	187450182	BCL6
chr3	187450159	187450182	RP11-211G3.3
chr3	187896972	187897346	AC022498.1
chr3	187896972	187897346	LPP
chr3	191986440	191986538	FGF12-AS1
chr3	191986440	191986538	FGF12
chr3	195076797	195076941	ACAP2
chr4	3344231	3344305	RGS12
chr4	3511523	3511678	AL590235.1
chr4	3511523	3511678	LRPAP1
chr4	3589633	3589777	LINC00955
chr4	3590661	3591100	LINC00955
chr4	4238803	4238805	TMEM128
chr4	4463629	4463664	STX18
chr4	5527058	5527223	C4orf6
chr4	5527922	5528058	C4orf6
chr4	6693782	6694189	AC093323.1
chr4	8000375	8000378	ABLIM2
chr4	8007126	8007129	ABLIM2
chr4	8612946	8612996	GPR78
chr4	8612946	8612996	CPZ
chr4	10525580	10525585	CLNK
chr4	15977813	15977818	PROM1
chr4	28364117	28364214	RP11-180C1.1
chr4	28372322	28372431	RP11-180C1.1
chr4	28383446	28383533	RP11-180C1.1
chr4	28394826	28394926	RP11-180C1.1
chr4	38628340	38628882	RP11-617D20.1
chr4	38628340	38628882	AC021860.1
chr4	54927213	54927377	AC110792.1
chr4	54927213	54927377	CHIC2
chr4	54927213	54927377	FIP1L1
chr4	57276682	57277131	AC068620.1
chr4	57276682	57277131	PPAT
chr4	69056959	69057034	FTLP10
chr4	69056959	69057034	RP11-646E20.6
chr4	69056959	69057034	TMPRSS11BNL
chr4	69057125	69057242	FTLP10
chr4	69057125	69057242	RP11-646E20.6

chr4	69057125	69057242	TMPRSS11BNL
chr4	69078080	69078195	FTLP10
chr4	69078080	69078195	RP11-646E20.6
chr4	69078080	69078195	TMPRSS11BNL
chr4	69083624	69083631	RP11-646E20.6
chr4	69083624	69083631	TMPRSS11BNL
chr4	76655092	76655164	USO1
chr4	76688671	76688682	USO1
chr4	78106250	78106262	CCNG2
chr4	78829479	78829529	MRPL1
chr4	83619200	83619235	SCD5
chr4	89192181	89192341	PPM1K
chr4	92240235	92240284	RP11-763F8.1
chr4	92240235	92240284	CCSER1
chr4	92240796	92240877	RP11-763F8.1
chr4	92240796	92240877	CCSER1
chr4	92246265	92246402	RP11-763F8.1
chr4	92246265	92246402	CCSER1
chr4	93218651	93218780	RP11-9B6.1
chr4	93221950	93221993	RP11-9B6.1
chr4	95498359	95498376	PDLIM5
chr4	95498535	95498574	PDLIM5
chr4	100242048	100242071	ADH1B
chr4	102269538	102269930	AP001816.1
chr4	106326915	106327105	PPA2
chr4	110569770	110569805	CCDC109B
chr4	110602591	110602625	CCDC109B
chr4	113532002	113532175	C4orf21
chr4	117220957	117221031	MTRNR2L13
chr4	120116569	120116723	RP11-455G16.1
chr4	120116866	120116996	RP11-455G16.1
chr4	120133497	120133667	RP11-455G16.1
chr4	122687200	122687499	AC079341.1
chr4	129143504	129143545	LARP1B
chr4	133356957	133357092	RP11-62N21.1
chr4	133367527	133367650	RP11-62N21.1
chr4	133368187	133368279	RP11-62N21.1
chr4	140809739	140809756	MAML3
chr4	147145712	147145760	RP11-6L6.2
chr4	147149362	147149674	RP11-6L6.2
chr4	147150177	147150208	RP11-6L6.2
chr4	155407595	155407672	DCHS2
chr4	157555875	157556023	RP11-171N4.2
chr4	157557720	157557758	RP11-171N4.2
chr4	157563417	157563474	RP11-171N4.2
chr4	157699353	157699382	PDGFC
chr4	178163809	178163857	RP11-487E13.1

chr4	178167182	178167274	RP11-487E13.1
chr4	178169350	178169432	RP11-487E13.1
chr4	178882004	178882059	LINC01098
chr4	178887829	178887932	LINC01098
chr4	178896958	178897097	LINC01098
chr4	178911633	178911662	LINC01098
chr4	185620729	185620756	CENPU
chr4	185734950	185735062	RP11-701P16.2
chr4	185734950	185735062	ACSL1
chr4	185736172	185736252	RP11-701P16.2
chr4	185736172	185736252	ACSL1
chr4	185742198	185742252	RP11-701P16.2
chr4	185742198	185742252	ACSL1
chr4	186393269	186393391	RP11-279O9.4
chr4	187111913	187112626	AC110771.1
chr4	187196336	187196339	F11
chr5	442578	442937	C5orf55
chr5	471908	471938	CTD-2228K2.5
chr5	471908	471938	EXOC3
chr5	472023	472080	CTD-2228K2.5
chr5	472023	472080	EXOC3
chr5	472794	473050	CTD-2228K2.5
chr5	667759	667976	AC026740.1
chr5	667759	667976	TPPP
chr5	668218	668313	AC026740.1
chr5	668218	668313	TPPP
chr5	668410	668839	AC026740.1
chr5	668410	668839	TPPP
chr5	979585	979634	RP11-661C8.3
chr5	981151	981286	RP11-661C8.3
chr5	1036895	1037018	NKD2
chr5	7654130	7654309	ADCY2
chr5	9265546	9265657	SEMA5A
chr5	9280914	9280919	SEMA5A
chr5	10505590	10505636	RP11-1C1.5
chr5	10506581	10506687	RP11-1C1.5
chr5	10509169	10509296	RP11-1C1.5
chr5	14490928	14490929	TRIO
chr5	23951673	23951912	C5orf17
chr5	23976106	23976159	C5orf17
chr5	23977890	23978031	C5orf17
chr5	23980046	23980095	C5orf17
chr5	31548597	31548794	C5orf22
chr5	32789508	32789873	AC026703.1
chr5	32789508	32789873	NPR3
chr5	33972139	33972140	RP11-1084J3.1
chr5	33972139	33972140	SLC45A2

chr5	35051012	35051053	AC010368.2
chr5	35051012	35051053	PRLR
chr5	35051193	35051198	AC010368.2
chr5	35051193	35051198	PRLR
chr5	35789599	35789807	CTD-2113L7.1
chr5	35789599	35789807	SPEF2
chr5	35789903	35790026	CTD-2113L7.1
chr5	35789903	35790026	SPEF2
chr5	35790127	35790311	CTD-2113L7.1
chr5	35790127	35790311	SPEF2
chr5	36612848	36613010	SLC1A3
chr5	39105659	39105865	AC008964.1
chr5	39105659	39105865	FYB
chr5	43384067	43384164	CCL28
chr5	51307412	51307436	CTD-2203A3.1
chr5	51374718	51374824	CTD-2203A3.1
chr5	51376577	51376696	CTD-2203A3.1
chr5	54252147	54252350	RP11-45H22.3
chr5	54253522	54253626	RP11-45H22.3
chr5	55753716	55753770	CTC-236F12.4
chr5	55759836	55759943	CTC-236F12.4
chr5	55760814	55760848	CTC-236F12.4
chr5	57838181	57838214	CTD-2117L12.1
chr5	57841919	57841986	CTD-2117L12.1
chr5	57842962	57843198	CTD-2117L12.1
chr5	57854013	57854036	CTD-2117L12.1
chr5	60411538	60411762	AC008498.1
chr5	60411538	60411762	NDUFAF2
chr5	61808070	61808309	CKS1B
chr5	61808070	61808309	KIF2A
chr5	61808070	61808309	IPO11
chr5	64593035	64593071	ADAMTS6
chr5	64820428	64820504	CENPK
chr5	67492310	67492317	RP11-404L6.2
chr5	67494018	67494171	RP11-404L6.2
chr5	72350896	72350917	FCHO2
chr5	72894203	72894224	AC008387.1
chr5	72896823	72896845	AC008387.1
chr5	78523714	78523911	AC016559.1
chr5	78523714	78523911	DMGDH
chr5	79731581	79731615	ZFYVE16
chr5	86513756	86514167	AC008394.1
chr5	86534632	86534684	AC008394.1
chr5	95192612	95192685	C5orf27
chr5	95194508	95194724	C5orf27
chr5	96149950	96150013	CTD-2260A17.2
chr5	96158763	96158820	CTD-2260A17.2

chr5	96208989	96209081	CTD-2260A17.2
chr5	96209218	96209261	CTD-2260A17.2
chr5	96438767	96438798	CTD-2215E18.1
chr5	96438767	96438798	LIX1
chr5	96519209	96519261	CTD-2215E18.1
chr5	101570447	101570719	AC008948.1
chr5	101570447	101570719	SLCO4C1
chr5	109219598	109219671	AC011366.3
chr5	109220138	109220174	AC011366.3
chr5	111755295	111756116	EPB41L4A-AS2
chr5	133622064	133622250	CDKL3
chr5	133622064	133622250	CTD-2410N18.4
chr5	134690739	134690810	H2AFY
chr5	134690739	134690810	C5orf66
chr5	136988763	136988765	KLHL3
chr5	138852108	138852506	AC138517.1
chr5	140242397	140242975	AC005609.1
chr5	140242397	140242975	PCDHA14
chr5	140242397	140242975	PCDHA1
chr5	140242397	140242975	PCDHA2
chr5	140242397	140242975	PCDHA3
chr5	140242397	140242975	PCDHA4
chr5	140242397	140242975	PCDHA5
chr5	140242397	140242975	PCDHA6
chr5	140242397	140242975	PCDHA7
chr5	140242397	140242975	PCDHA8
chr5	140242397	140242975	PCDHA9
chr5	140242397	140242975	PCDHA10
chr5	140940458	140940469	CTD-2024I7.13
chr5	140940458	140940469	DIAPH1
chr5	147698550	147698623	AC091948.1
chr5	147698550	147698623	RP11-373N22.3
chr5	147701843	147701891	AC091948.1
chr5	147701843	147701891	RP11-373N22.3
chr5	147701843	147701891	SPINK9
chr5	147900485	147900526	HTR4
chr5	150077288	150077300	RBM22
chr5	150158132	150158534	SMIM3
chr5	150158132	150158534	AC010441.1
chr5	150711990	150712004	SLC36A2
chr5	156738045	156738058	CYFIP2
chr5	156822574	156822687	CTB-109A12.1
chr5	156822574	156822687	CYFIP2
chr5	156822574	156822687	ADAM19
chr5	156825169	156825222	CTB-109A12.1
chr5	156825169	156825222	ADAM19
chr5	156999278	156999283	ADAM19

chr5	159696466	159696551	CCNJL
chr5	159742389	159742512	CCNJL
chr5	159766452	159766528	CCNJL
chr5	162873776	162873821	NUDCD2
chr5	162873776	162873821	AC112205.1
chr5	167566511	167566522	CTB-178M22.1
chr5	167566511	167566522	TENM2
chr5	167576433	167576447	CTB-178M22.1
chr5	167576433	167576447	TENM2
chr5	169678652	169678659	C5orf58
chr5	169678652	169678659	LCP2
chr5	169988118	169988119	KCNIP1
chr5	171200827	171200962	CTB-78H18.1
chr5	171201346	171201737	CTB-78H18.1
chr5	172036245	172036436	AC027309.1
chr5	172333063	172333183	ERGIC1
chr5	172342521	172342694	ERGIC1
chr5	175477606	175477735	RP11-826N14.2
chr5	175487235	175487293	RP11-826N14.2
chr5	175731320	175731421	SIMC1
chr5	176292551	176292722	UNC5A
chr5	177210798	177210856	RP11-1026M7.2
chr5	177220343	177220472	RP11-1026M7.2
chr5	177682788	177682890	COL23A1
chr5	177989027	177989239	COL23A1
chr5	179125503	179125604	CANX
chr5	179287310	179287780	CTC-241N9.1
chr5	179287310	179287780	C5orf45
chr5	180480902	180480925	BTNL9
chr5	180682781	180683218	CTC-338M12.4
chr5	180682781	180683218	TRIM52
chr5	180682781	180683218	AC008443.1
chr6	1101508	1101531	AL033381.1
chr6	1102041	1102415	AL033381.1
chr6	2623673	2624056	C6orf195
chr6	3983402	3983710	C6ORF50
chr6	8419047	8419118	SLC35B3
chr6	8435442	8435642	SLC35B3
chr6	10633993	10634929	GCNT6
chr6	10647263	10647501	GCNT6
chr6	10798071	10798106	TMEM14B
chr6	10798071	10798106	MAK
chr6	10798071	10798106	SYCP2L
chr6	10798071	10798106	RP11-637O19.3
chr6	13288633	13288645	RP1-257A7.4
chr6	13288633	13288645	TBC1D7
chr6	13288633	13288645	PHACTR1

chr6	15401182	15401248	JARID2
chr6	15509195	15509345	JARID2
chr6	15546273	15546298	DTNBP1
chr6	17936608	17936616	KIF13A
chr6	18368986	18369049	RNF144B
chr6	20126125	20126136	MBOAT1
chr6	25479336	25479475	LRRC16A
chr6	28911681	28911803	C6orf100
chr6	28911999	28912064	C6orf100
chr6	29704192	29704200	HLA-F
chr6	29704192	29704200	HLA-F-AS1
chr6	30867825	30867859	DDR1
chr6	31466428	31466483	MICB
chr6	33553402	33553498	GGNBP1
chr6	33554447	33554565	GGNBP1
chr6	33554447	33554565	LINC00336
chr6	33556690	33556803	GGNBP1
chr6	33556690	33556803	LINC00336
chr6	35057961	35058013	ANKS1A
chr6	35203223	35203270	SCUBE3
chr6	35283882	35283923	DEF6
chr6	35806483	35806575	SRPK1
chr6	37012636	37012965	COX6A1P2
chr6	39585504	39585529	KIF6
chr6	42601986	42602032	UBR2
chr6	44400472	44400720	CDC5L
chr6	44400472	44400720	AL133262.1
chr6	45296060	45296197	RUNX2
chr6	45296060	45296197	SUPT3H
chr6	46626226	46626265	SLC25A27
chr6	46822454	46822518	GPR116
chr6	52387682	52387756	EFHC1
chr6	52387682	52387756	TRAM2
chr6	56466803	56466804	DST
chr6	57039180	57039193	BAG2
chr6	62284233	62284307	MTRNR2L9
chr6	70769654	70769689	COL19A1
chr6	71469531	71469606	SMAP1
chr6	72885064	72885075	RIMS1
chr6	73835131	73835155	KCNQ5
chr6	73835243	73835247	KCNQ5
chr6	82262307	82262537	AL359693.1
chr6	82262307	82262537	FAM46A
chr6	84321157	84321162	SNAP91
chr6	88107933	88108095	C6orf164
chr6	88107933	88108095	RP1-102H19.8
chr6	88109156	88109223	C6orf164

chr6	88109156	88109223	RP1-102H19.8
chr6	88360891	88361002	ORC3
chr6	89675362	89675796	AL079342.1
chr6	99949815	99949885	USP45
chr6	110774832	110774898	SLC22A16
chr6	110964394	110964477	CDK19
chr6	112381216	112381278	WISP3
chr6	116323848	116323887	FRK
chr6	116579609	116580130	RP3-486I3.4
chr6	116579609	116580130	DSE
chr6	119532053	119532095	MAN1A1
chr6	119653101	119653169	MAN1A1
chr6	124603384	124603392	NKAIN2
chr6	125369358	125369405	RNF217
chr6	126221142	126221167	NCOA7
chr6	137539587	137539589	IFNGR1
chr6	138699210	138699320	RP3-422G23.4
chr6	138703637	138703984	RP3-422G23.4
chr6	143763306	143763308	AL031320.1
chr6	143763306	143763308	ADAT2
chr6	143764263	143764295	AL031320.1
chr6	143764263	143764295	ADAT2
chr6	151233804	151233841	MTHFD1L
chr6	152801896	152801916	SYNE1
chr6	153667705	153668592	AL590867.1
chr6	154735857	154735899	CNKSR3
chr6	158514920	158515042	SYNJ2
chr6	159047471	159047478	AL591025.1
chr6	159047471	159047478	TMEM181
chr6	159047695	159047738	AL591025.1
chr6	159047695	159047738	TMEM181
chr6	159291223	159291342	C6orf99
chr6	159294558	159294653	C6orf99
chr6	163587068	163587294	AL078585.1
chr6	163587068	163587294	PACRG
chr6	163611940	163612014	AL078585.1
chr6	163611940	163612014	PACRG
chr6	163612634	163612826	AL078585.1
chr6	163612634	163612826	PACRG
chr6	166307713	166308153	SDIM1
chr6	166307713	166308153	PDE10A
chr6	166889272	166889327	RPS6KA2
chr6	166945703	166946107	Z98049.1
chr6	166945703	166946107	RPS6KA2
chr6	168188244	168188287	C6orf123
chr6	168191627	168191756	C6orf123
chr6	168196616	168197182	C6orf123

chr6	168196616	168197182	AL009178.1
chr7	228396	228860	AC145676.2
chr7	228396	228860	FAM20C
chr7	302918	303160	AC187652.1
chr7	303163	303255	AC187652.1
chr7	303260	303304	AC187652.1
chr7	303309	303330	AC187652.1
chr7	303597	303622	AC187652.1
chr7	303627	303645	AC187652.1
chr7	304018	304070	AC187652.1
chr7	829090	829190	HEATR2
chr7	855528	855566	SUN1
chr7	855873	855930	SUN1
chr7	856254	856310	SUN1
chr7	1543959	1544243	INTS1
chr7	1545432	1545489	INTS1
chr7	1732947	1733531	AC074389.6
chr7	1732947	1733531	ELFN1
chr7	1878377	1878504	AC110781.3
chr7	1878377	1878504	MAD1L1
chr7	1886884	1887418	AC110781.3
chr7	1886884	1887418	MAD1L1
chr7	3179868	3179930	AC091801.1
chr7	3180554	3180633	AC091801.1
chr7	3180724	3180834	AC091801.1
chr7	3186876	3186905	AC091801.1
chr7	3188615	3188702	AC091801.1
chr7	3197771	3197888	AC091801.1
chr7	3205664	3205728	AC091801.1
chr7	4714886	4714956	FOXK1
chr7	6212362	6212555	CYTH3
chr7	6713376	6713397	AC073343.1
chr7	6713376	6713397	AC073343.13
chr7	6713424	6713456	AC073343.1
chr7	6713424	6713456	AC073343.13
chr7	6713535	6713674	AC073343.1
chr7	6713535	6713674	AC073343.13
chr7	6713781	6713798	AC073343.1
chr7	6713781	6713798	AC073343.13
chr7	6713803	6713855	AC073343.1
chr7	6713803	6713855	AC073343.13
chr7	6713920	6714075	AC073343.1
chr7	6713920	6714075	AC073343.13
chr7	6714482	6714584	AC073343.1
chr7	6714482	6714584	AC073343.13
chr7	6714599	6714653	AC073343.1
chr7	6714599	6714653	AC073343.13

chr7	6714739	6714811	AC073343.1
chr7	6714739	6714811	AC073343.13
chr7	6714865	6714953	AC073343.1
chr7	6714865	6714953	AC073343.13
chr7	6714990	6714994	AC073343.1
chr7	6714990	6714994	AC073343.13
chr7	6715247	6715369	AC073343.1
chr7	6715247	6715369	AC073343.13
chr7	6715404	6715618	AC073343.1
chr7	6715404	6715618	AC073343.13
chr7	6715621	6715991	AC073343.1
chr7	6715621	6715991	AC073343.13
chr7	14789758	14789778	DGKB
chr7	16872880	16872932	AGR2
chr7	21890933	21890938	DNAH11
chr7	23210760	23210807	AC005082.1
chr7	23210760	23210807	KLHL7
chr7	23211775	23211796	AC005082.1
chr7	23211775	23211796	KLHL7
chr7	26576372	26576639	KIAA0087
chr7	26578025	26578173	KIAA0087
chr7	27886122	27886166	JAZF1
chr7	28060172	28060331	JAZF1
chr7	29484763	29484773	CHN2
chr7	33069222	33069335	NT5C3A
chr7	33069222	33069335	AVL9
chr7	33078454	33078516	NT5C3A
chr7	33078454	33078516	AVL9
chr7	33767242	33767877	RP11-89N17.1
chr7	34685709	34685726	AC005493.1
chr7	34685709	34685726	NPSR1-AS1
chr7	34687590	34687624	AC005493.1
chr7	34687590	34687624	NPSR1-AS1
chr7	34699742	34699772	AC005493.1
chr7	34699742	34699772	NPSR1
chr7	34699742	34699772	NPSR1-AS1
chr7	35851671	35851678	7-Sep
chr7	35854755	35854756	7-Sep
chr7	35871700	35871723	7-Sep
chr7	35928433	35928455	7-Sep
chr7	36118843	36118925	PP13004
chr7	36124082	36124370	PP13004
chr7	37243714	37243905	ELMO1
chr7	38725198	38725605	FAM183B
chr7	39160433	39160455	POU6F2
chr7	40256996	40257120	SUGCT
chr7	45902684	45902770	AC096582.1

chr7	45905635	45906045	AC096582.1
chr7	46728998	46729144	AC011294.3
chr7	46732409	46732567	AC011294.3
chr7	46736601	46736612	AC011294.3
chr7	48887501	48887911	AC004899.3
chr7	48887501	48887911	AC004899.1
chr7	62810010	62810456	AC006455.1
chr7	62810010	62810456	RP5-905H7.10
chr7	62810010	62810456	RP5-905H7.9
chr7	62858414	62858860	AC073188.1
chr7	62858414	62858860	SLC25A1P2
chr7	62858414	62858860	RP11-340I6.3
chr7	64383764	64383783	ZNF273
chr7	65591623	65591630	AC068533.7
chr7	65591623	65591630	CRCP
chr7	65591623	65591630	RP5-1132H15.1
chr7	65939543	65939677	AC008267.1
chr7	65939543	65939677	GS1-124K5.2
chr7	72459478	72459492	AC005488.1
chr7	72459478	72459492	STAG3L3
chr7	72464847	72464894	AC005488.1
chr7	72464847	72464894	STAG3L3
chr7	72464982	72464996	AC005488.1
chr7	72464982	72464996	STAG3L3
chr7	72986278	72986365	TBL2
chr7	74999908	74999922	AC006014.1
chr7	74999908	74999922	STAG3L1
chr7	75000010	75000057	AC006014.1
chr7	75000010	75000057	STAG3L1
chr7	75005411	75005425	AC006014.1
chr7	75005411	75005425	STAG3L1
chr7	75914572	75914574	SRRM3
chr7	78212738	78212771	MAGI2
chr7	80422795	80422809	SEMA3C
chr7	80551581	80551596	SEMA3C
chr7	80804829	80804897	AC005008.2
chr7	80805200	80805305	AC005008.2
chr7	80817106	80817246	AC005008.2
chr7	80819221	80819265	AC005008.2
chr7	91599787	91599822	AKAP9
chr7	91779221	91779239	CTB-161K23.1
chr7	91779221	91779239	LRRD1
chr7	95171684	95172106	AC002451.1
chr7	97856054	97856081	TECPR1
chr7	98466828	98466855	TMEM130
chr7	98937605	98937632	ARPC1A
chr7	99040552	99040576	AC073063.10

chr7	99040552	99040576	AC073063.1
chr7	99040552	99040576	PTCD1
chr7	99040552	99040576	ATP5J2-PTCD1
chr7	99040552	99040576	CPSF4
chr7	99040722	99040747	AC073063.10
chr7	99040722	99040747	AC073063.1
chr7	99040722	99040747	PTCD1
chr7	99040722	99040747	ATP5J2-PTCD1
chr7	99040722	99040747	CPSF4
chr7	99058140	99058152	PTCD1
chr7	99058140	99058152	ATP5J2-PTCD1
chr7	99058140	99058152	ATP5J2
chr7	101952825	101952908	SH2B2
chr7	102789726	102789928	NAPEPLD
chr7	102969378	102969499	PMPCB
chr7	102969378	102969499	DNAJC2
chr7	104110492	104110502	LHFPL3
chr7	104263451	104263471	LHFPL3
chr7	105668108	105668118	CDHR3
chr7	107332499	107332522	SLC26A4
chr7	107791165	107791198	NRCAM
chr7	108120017	108120020	PNPLA8
chr7	108540389	108540655	FLJ00325
chr7	112757454	112757633	LINC00998
chr7	112757454	112757633	RP11-736E3.1
chr7	113091127	113091456	TSRM
chr7	114296495	114296602	FOXP2
chr7	116595139	116595207	ST7
chr7	116595139	116595207	ST7-OT4
chr7	116596477	116596785	ST7
chr7	116596477	116596785	ST7-OT4
chr7	116598643	116598739	ST7
chr7	116598643	116598739	ST7-OT4
chr7	116599207	116599346	ST7
chr7	116599207	116599346	ST7-OT4
chr7	116606149	116606176	AC106873.4
chr7	116606149	116606176	ST7
chr7	116606149	116606176	ST7-OT4
chr7	116607345	116607475	AC106873.4
chr7	116607345	116607475	ST7
chr7	116607345	116607475	ST7-OT4
chr7	116608453	116608468	ST7
chr7	116608453	116608468	ST7-OT4
chr7	128923225	128923265	AHCYL2
chr7	132409747	132409791	AC009365.3
chr7	132412194	132412601	AC009365.3
chr7	133261269	133261515	EXOC4

chr7	134250233	134250298	AKR1B15
chr7	134605386	134605471	CALD1
chr7	134694793	134695016	AGBL3
chr7	134784711	134784831	C7orf49
chr7	134784711	134784831	AGBL3
chr7	134785908	134786029	C7orf49
chr7	134785908	134786029	AGBL3
chr7	139421823	139421841	HIPK2
chr7	140110773	140110855	RAB19
chr7	142099455	142099752	TRBV7-8
chr7	142099890	142099938	TRBV7-8
chr7	142104121	142104415	TRBV6-9
chr7	142104505	142104553	TRBV6-9
chr7	142375063	142375137	MTRNR2L6
chr7	144100750	144100786	NOBOX
chr7	144228157	144228262	TPK1
chr7	147335901	147335970	CNTNAP2
chr7	148848336	148848374	ZNF398
chr7	148982564	148982587	RP4-800G7.2
chr7	148982564	148982587	ZNF783
chr7	150717375	150717434	ATG9B
chr7	154720359	154720477	PAXIP1-AS2
chr7	154720666	154720734	PAXIP1-AS2
chr7	154860926	154860955	HTR5A-AS1
chr7	154861986	154862163	HTR5A
chr7	154861986	154862163	HTR5A-AS1
chr7	154988630	154988697	AC099552.4
chr7	154988972	154989103	AC099552.4
chr7	154989979	154990010	AC099552.4
chr7	155174819	155175010	AC008060.7
chr7	155187741	155187869	AC008060.7
chr7	155403994	155404111	AC009403.2
chr7	155436162	155436262	AC009403.2
chr7	156432698	156433348	C7orf13
chr7	156432698	156433348	RNF32
chr7	156432698	156433348	LINC01006
chr7	156903160	156903573	AC006967.1
chr7	157318547	157319038	AC006372.1
chr7	157406721	157406793	AC005481.5
chr7	157406721	157406793	PTPRN2
chr7	157408081	157408358	AC005481.5
chr7	157408081	157408358	PTPRN2
chr7	158662137	158662181	WDR60
chr8	28905	29093	AC144568.2
chr8	142183	142298	RP11-585F1.10
chr8	2986334	2986422	CSMD1
chr8	8046158	8046331	LRLE1

chr8	8046158	8046331	ENPP7P1
chr8	8046158	8046331	FAM85B
chr8	9009359	9009426	RP11-10A14.4
chr8	9011716	9011853	RP11-10A14.4
chr8	9012476	9012572	RP11-10A14.4
chr8	10986311	10986553	AF131215.5
chr8	10986311	10986553	XKR6
chr8	11292575	11292611	FAM167A
chr8	11292575	11292611	C8orf12
chr8	11295549	11295719	FAM167A
chr8	11295549	11295719	C8orf12
chr8	11295945	11296051	FAM167A
chr8	11295945	11296051	C8orf12
chr8	11618923	11619615	C8orf49
chr8	11659318	11659529	RP11-297N6.4
chr8	11659318	11659529	FDFT1
chr8	11659930	11659996	RP11-297N6.4
chr8	11659930	11659996	FDFT1
chr8	11870658	11870660	RP11-481A20.11
chr8	11872340	11872558	RP11-481A20.11
chr8	22430850	22430935	SORBS3
chr8	23412287	23412318	SLC25A37
chr8	23412287	23412318	AC051642.1
chr8	23420639	23420651	SLC25A37
chr8	23420639	23420651	AC051642.1
chr8	23431128	23431469	FP15737
chr8	23431128	23431469	SLC25A37
chr8	26943765	26943802	RP11-521M14.2
chr8	26944696	26944766	RP11-521M14.2
chr8	27472172	27472298	CLU
chr8	28867201	28867238	HMBOX1
chr8	32606199	32606212	NRG1
chr8	33251736	33251773	FUT10
chr8	35650399	35650815	UNC5D
chr8	35650399	35650815	AC012215.1
chr8	37593398	37594015	RP11-863K10.7
chr8	50449023	50449057	RP11-738G5.2
chr8	50449716	50449811	RP11-738G5.2
chr8	50457010	50457076	RP11-738G5.2
chr8	52730402	52730497	AC090186.1
chr8	52730402	52730497	PCMTD1
chr8	56048154	56048212	RP11-386G21.1
chr8	56048154	56048212	XKR4
chr8	56052650	56052776	RP11-386G21.1
chr8	56052650	56052776	XKR4
chr8	56054683	56054688	RP11-386G21.1
chr8	56054683	56054688	XKR4

chr8	56074186	56074204	RP11-386G21.2
chr8	56074186	56074204	XKR4
chr8	56076678	56076853	RP11-386G21.2
chr8	56076678	56076853	XKR4
chr8	56076973	56077004	RP11-386G21.2
chr8	56076973	56077004	XKR4
chr8	62200764	62200793	RP11-787D18.2
chr8	62200764	62200793	CLVS1
chr8	62204877	62204975	CLVS1
chr8	64101293	64101376	YTHDF3
chr8	69217005	69217036	RP11-664D7.4
chr8	69218753	69218833	RP11-664D7.4
chr8	70850403	70850618	AC090574.1
chr8	71486420	71486566	AC120194.1
chr8	71486420	71486566	TRAM1
chr8	71572797	71573429	RP11-382J12.1
chr8	71572797	71573429	LACTB2
chr8	72317040	72317149	RP11-1102P16.1
chr8	72383093	72383166	RP11-1102P16.1
chr8	72447979	72447989	RP11-1102P16.1
chr8	72875158	72875209	RP11-383H13.1
chr8	72875248	72875283	RP11-383H13.1
chr8	72877644	72877769	RP11-383H13.1
chr8	72914457	72914504	RP11-383H13.1
chr8	75525103	75525175	RP11-730G20.1
chr8	75525103	75525175	RP11-758M4.1
chr8	75614614	75614691	RP11-758M4.1
chr8	75615841	75615917	RP11-758M4.1
chr8	75664676	75664753	RP11-758M4.1
chr8	86134884	86135082	CA13
chr8	86134884	86135082	RP11-219B4.5
chr8	86138738	86138805	CA13
chr8	86138738	86138805	RP11-219B4.5
chr8	86158554	86158783	RP11-219B4.6
chr8	86158554	86158783	CA13
chr8	86162053	86162077	RP11-219B4.6
chr8	86162053	86162077	CA13
chr8	86573699	86575726	REXO1L1P
chr8	86747543	86749570	REXO1L11P
chr8	86756718	86758745	REXO1L10P
chr8	93897043	93897258	CTD-3239E11.2
chr8	93897043	93897258	AC117834.1
chr8	93897043	93897258	TRIQK
chr8	94147604	94147664	C8orf87
chr8	94241867	94242146	AC016885.1
chr8	94241867	94242146	RP11-388K12.1
chr8	94241867	94242146	LINC00535

chr8	94242198	94242388	AC016885.1
chr8	94242198	94242388	RP11-388K12.1
chr8	94242198	94242388	LINC00535
chr8	94752481	94752789	RBM12B-AS1
chr8	94752481	94752789	RBM12B
chr8	95559047	95559292	AC023632.1
chr8	95559047	95559292	KIAA1429
chr8	95993161	95993201	NDUFAF6
chr8	96087861	96087923	RP11-320N21.2
chr8	96087861	96087923	NDUFAF6
chr8	103541076	103541083	KB-1980E6.3
chr8	103541539	103541656	KB-1980E6.3
chr8	103876579	103876839	AZIN1
chr8	103876579	103876839	KB-1507C5.2
chr8	104145255	104145572	C8orf56
chr8	105727198	105727203	RP11-127H5.1
chr8	105839585	105839706	RP11-127H5.1
chr8	105845553	105845673	RP11-127H5.1
chr8	105907635	105907679	RP11-127H5.1
chr8	106003852	106003897	RP11-127H5.1
chr8	106258521	106258588	RP11-127H5.1
chr8	114612622	114612667	RP11-67H2.1
chr8	114627733	114627773	RP11-67H2.1
chr8	119294558	119294569	AC023590.1
chr8	119294558	119294569	SAMD12
chr8	119296511	119296609	AC023590.1
chr8	119296511	119296609	SAMD12
chr8	128959864	128960280	TMEM75
chr8	128959864	128960280	PVT1
chr8	131353088	131353183	ASAP1
chr8	132947755	132947764	EFR3A
chr8	140944530	140944829	C8orf17
chr8	140944530	140944829	TRAPPC9
chr8	141682252	141682256	PTK2
chr8	142524797	142524863	AC138647.1
chr8	142528316	142528815	AC138647.1
chr8	144125591	144125608	C8orf31
chr9	3469155	3469181	AL365202.1
chr9	3469155	3469181	RFX3
chr9	4859120	4859260	AL158147.2
chr9	4859120	4859260	RCL1
chr9	5077163	5077181	AL161450.1
chr9	5077163	5077181	JAK2
chr9	5084568	5084580	AL161450.1
chr9	5084568	5084580	JAK2
chr9	6468385	6468741	C9orf38
chr9	6468385	6468741	UHRF2

chr9	17494905	17495003	CNTLN
chr9	21967199	21967438	C9orf53
chr9	21967199	21967438	RP11-145E5.5
chr9	32498310	32498337	DDX58
chr9	33391454	33391529	AQP7
chr9	34487944	34488189	DNAI1
chr9	35104619	35104643	FAM214B
chr9	35361056	35361262	AL160274.1
chr9	35361056	35361262	UNC13B
chr9	35749203	35749266	GBA2
chr9	35749203	35749266	RGP1
chr9	35749369	35749405	GBA2
chr9	35749369	35749405	RGP1
chr9	36141843	36141868	GLIPR2
chr9	37715665	37715679	FRMPD1
chr9	37715665	37715679	RP11-613M10.9
chr9	38543250	38543360	RP11-392E22.5
chr9	38543250	38543360	ANKRD18A
chr9	38543250	38543360	ANKRD18A
chr9	38543250	38543360	RP11-392E22.11
chr9	38543458	38543493	ANKRD18A
chr9	38543458	38543493	ANKRD18A
chr9	38543458	38543493	RP11-392E22.11
chr9	38571119	38571198	ANKRD18A
chr9	38571119	38571198	ANKRD18A
chr9	42411969	42412118	RP11-146D12.2
chr9	42419017	42419022	RP11-146D12.2
chr9	42704004	42704049	CBWD7
chr9	43082880	43082885	ANKRD20A3
chr9	43089717	43089866	ANKRD20A3
chr9	43135095	43135514	AL513478.1
chr9	44325916	44326077	BX088651.2
chr9	44401890	44402255	BX088651.1
chr9	44401890	44402255	RP11-475I24.3
chr9	44869083	44869140	RP11-160N1.10
chr9	44869923	44870104	RP11-160N1.10
chr9	44990451	44990528	FAM27C
chr9	44991083	44991208	FAM27C
chr9	45441273	45441542	AL354718.1
chr9	45727244	45727321	FAM27A
chr9	45727874	45727999	FAM27A
chr9	45733588	45733965	RP11-7G23.8
chr9	45733588	45733965	FAM27E2
chr9	46386622	46387002	FAM27E1
chr9	46390277	46390900	FAM27D1
chr9	67785913	67786254	FAM27E3
chr9	67785913	67786254	RP11-12A20.2

chr9	67793213	67793338	RP11-12A20.7
chr9	67793213	67793338	FAM27B
chr9	67793897	67793974	RP11-12A20.7
chr9	67793897	67793974	FAM27B
chr9	67924791	67925210	BX649567.1
chr9	67977438	67977567	RP11-195B21.3
chr9	67984833	67985114	RP11-195B21.3
chr9	67985711	67985922	RP11-195B21.3
chr9	67987826	67987998	RP11-195B21.3
chr9	69252845	69253282	BX255923.1
chr9	69252845	69253282	CBWD6
chr9	69380011	69380430	CR769776.1
chr9	69653111	69653179	AL445665.1
chr9	69655521	69655673	AL445665.1
chr9	69660302	69660358	AL445665.1
chr9	70646864	70647145	AL591479.1
chr9	70866135	70866572	AL353608.1
chr9	70866135	70866572	CBWD3
chr9	77352311	77352366	TRPM6
chr9	82308549	82308575	TLE4
chr9	88581511	88581587	NAA35
chr9	90316751	90316825	DAPK1
chr9	94056967	94057079	AUH
chr9	96285562	96285645	FAM120A
chr9	97059097	97059131	ZNF169
chr9	97766506	97766568	C9orf3
chr9	98534606	98534956	DKFZP434H0512
chr9	98534606	98534956	LINC00476
chr9	98668933	98668992	ERCC6L2
chr9	100053684	100053769	RP11-23J9.7
chr9	100053684	100053769	RP11-23J9.5
chr9	100053684	100053769	CCDC180
chr9	100053684	100053769	RP11-23J9.4
chr9	100054838	100054957	RP11-23J9.7
chr9	100054838	100054957	RP11-23J9.5
chr9	100054838	100054957	CCDC180
chr9	100054838	100054957	RP11-23J9.4
chr9	100056229	100056392	RP11-23J9.7
chr9	100056229	100056392	RP11-23J9.5
chr9	100056229	100056392	CCDC180
chr9	100056229	100056392	RP11-23J9.4
chr9	100057127	100057273	RP11-23J9.7
chr9	100057127	100057273	RP11-23J9.5
chr9	100057127	100057273	CCDC180
chr9	100057127	100057273	RP11-23J9.4
chr9	100057902	100058047	RP11-23J9.7
chr9	100057902	100058047	RP11-23J9.5

chr9	100057902	100058047	CCDC180
chr9	100057902	100058047	RP11-23J9.4
chr9	100854780	100854822	TRIM14
chr9	101890216	101890284	TGFBR1
chr9	104151728	104151769	MRPL50
chr9	110540006	110540380	AL162389.1
chr9	113220395	113220399	SVEP1
chr9	113761673	113761720	LPAR1
chr9	113800932	113800979	LPAR1
chr9	115246826	115246906	C9orf147
chr9	115248670	115248821	C9orf147
chr9	115249259	115249484	C9orf147
chr9	115249259	115249484	KIAA1958
chr9	116028516	116028674	CDC26
chr9	116028516	116028674	SLC31A1
chr9	119160757	119161065	PAPPA-AS1
chr9	119160757	119161065	PAPPA
chr9	122257779	122257835	RP11-295D22.1
chr9	122275203	122275261	RP11-295D22.1
chr9	122285183	122285246	RP11-295D22.1
chr9	123452204	123452273	MEGF9
chr9	123453688	123453752	MEGF9
chr9	125148344	125148406	AL162424.1
chr9	125148344	125148406	PTGS1
chr9	126150009	126150137	DENND1A
chr9	126763949	126763976	LHX2
chr9	127213423	127213478	GPR144
chr9	127239278	127239379	GPR144
chr9	129138725	129138907	MVB12B
chr9	130524671	130524695	SH2D3C
chr9	130890612	130890624	PTGES2
chr9	130890612	130890624	AL590708.2
chr9	130890799	130891481	AL590708.2
chr9	131063193	131063618	AL359091.2
chr9	132672446	132672456	FNBP1
chr9	132681438	132681491	FNBP1
chr9	132903812	132904381	AL360004.1
chr9	133978268	133978375	AIF1L
chr9	138150512	138150990	AL390778.1
chr9	138151020	138151275	AL390778.1
chr9	139219089	139219640	DKFZP434A062
chr9	139863820	139863925	C9orf141
chr9	139864338	139864511	C9orf141
chr9	139865416	139865495	C9orf141
chr9	139865950	139866282	C9orf141
chr9	139980807	139981121	AL807752.1
chr9	140510121	140510651	C9orf37

chr9	140686507	140686572	EHMT1
chr9	140911591	140911653	CACNA1B
chr9	140927134	140927222	CACNA1B
chr9	140934999	140935124	CACNA1B
chr9	140944527	140944538	CACNA1B
chr9	140963060	140963065	CACNA1B
chr10	1018440	1018871	AL359878.1
chr10	5196335	5196377	AKR1CL1
chr10	5197879	5197952	AKR1CL1
chr10	5199836	5200001	AKR1CL1
chr10	5200798	5200907	AKR1CL1
chr10	5202082	5202135	AKR1CL1
chr10	5202775	5202852	AKR1CL1
chr10	5203719	5203730	AKR1CL1
chr10	5203819	5203935	AKR1CL1
chr10	5204816	5204983	AKR1CL1
chr10	5226975	5227067	AKR1CL1
chr10	5244427	5244449	AKR1CL1
chr10	5244427	5244449	AKR1C4
chr10	6392280	6392831	DKFZP667F0711
chr10	7601756	7601845	ITIH5
chr10	13350145	13350447	AL138764.1
chr10	13364055	13364065	SEPHS1
chr10	13568138	13568221	BEND7
chr10	13570473	13570533	RP11-214D15.2
chr10	13570473	13570533	BEND7
chr10	17896743	17896832	MRC1L1
chr10	21358883	21358944	NEBL
chr10	23493102	23493184	C10orf115
chr10	23512527	23512587	C10orf115
chr10	23512673	23512747	C10orf115
chr10	23528658	23528723	C10orf115
chr10	24544409	24544525	KIAA1217
chr10	24770273	24770632	AL353583.1
chr10	24770273	24770632	KIAA1217
chr10	24879266	24879408	ARHGAP21
chr10	27339988	27340293	ANKRD26
chr10	28089761	28089781	ARMC4
chr10	28346686	28346778	MPP7
chr10	29785326	29785365	SVIL
chr10	30653635	30653996	MTPAP
chr10	30653635	30653996	GOLGA2P6
chr10	30654078	30654262	MTPAP
chr10	30654078	30654262	GOLGA2P6
chr10	31609055	31609136	ZEB1-AS1
chr10	31609055	31609136	ZEB1
chr10	31609535	31609627	ZEB1

chr10	31676053	31676155	RP11-192P3.5
chr10	31676053	31676155	ZEB1
chr10	33195393	33195508	ITGB1
chr10	36811744	36813162	NAMPTL
chr10	37890898	37890972	MTRNR2L7
chr10	38717129	38717224	LINC00999
chr10	38724058	38724181	LINC00999
chr10	38726018	38726134	LINC00999
chr10	38726472	38726512	LINC00999
chr10	43657648	43657669	CSGALNACT2
chr10	43977832	43978115	ZNF487
chr10	43978329	43978591	ZNF487
chr10	43991464	43991517	ZNF487
chr10	44793339	44793345	CXCL12
chr10	44873687	44873737	AL137026.1
chr10	44873687	44873737	CXCL12
chr10	44878713	44878739	AL137026.1
chr10	44878713	44878739	CXCL12
chr10	46147054	46147073	ZFAND4
chr10	47219702	47219811	AGAP10
chr10	47219702	47219811	RP11-144G6.12
chr10	47232156	47232295	AGAP10
chr10	47232156	47232295	RP11-144G6.12
chr10	47232156	47232295	BMS1P2
chr10	47235421	47235609	AGAP10
chr10	47235421	47235609	RP11-144G6.12
chr10	47235421	47235609	BMS1P2
chr10	47239687	47239738	AGAP10
chr10	47239687	47239738	RP11-144G6.12
chr10	47239687	47239738	BMS1P2
chr10	48189612	48189663	BMS1P6
chr10	48189612	48189663	AGAP9
chr10	48193742	48193930	BMS1P6
chr10	48193742	48193930	AGAP9
chr10	48197056	48197195	BMS1P6
chr10	48197056	48197195	AGAP9
chr10	50009550	50009553	WDFY4
chr10	50323476	50323530	VSTM4
chr10	50599882	50599907	DRGX
chr10	51573300	51573416	NCOA4
chr10	52095002	52095223	AC069547.1
chr10	52095002	52095223	SGMS1
chr10	52152783	52153163	AC069547.2
chr10	52152783	52153163	SGMS1
chr10	52384680	52384795	RP11-50E11.3
chr10	52384680	52384795	SGMS1
chr10	53841374	53841417	PRKG1

chr10	56335033	56335041	RP11-257I14.1
chr10	56335033	56335041	PCDH15
chr10	56400831	56400836	RP11-257I14.1
chr10	56400831	56400836	PCDH15
chr10	57359660	57359734	MTRNR2L5
chr10	57359660	57359734	PCDH15
chr10	61718043	61718429	C10orf40
chr10	71021814	71021862	HKDC1
chr10	71444655	71444874	RP11-242G20.1
chr10	71450357	71450485	RP11-242G20.1
chr10	71450826	71450860	RP11-242G20.1
chr10	71620448	71620455	COL13A1
chr10	72542145	72542155	TBATA
chr10	73156946	73157075	CDH23
chr10	73976579	73976611	ANAPC16
chr10	73976579	73976611	ASCC1
chr10	74235620	74235621	MICU1
chr10	74288511	74288516	MICU1
chr10	78782686	78782694	KCNMA1
chr10	79626704	79626866	AL391421.1
chr10	79626704	79626866	DLG5
chr10	79627681	79627895	AL391421.1
chr10	79627681	79627895	DLG5
chr10	81266449	81267541	AL133481.1
chr10	81270285	81270319	AL133481.1
chr10	81270285	81270319	RP11-342M3.2
chr10	81374797	81374798	SFTPA1
chr10	82012483	82013325	AL359195.1
chr10	82394041	82394261	SH2D4B
chr10	89420746	89420784	PAPSS2
chr10	89519512	89519544	ATAD1
chr10	91398906	91398999	PANK1
chr10	91526024	91526203	KIF20B
chr10	92987094	92987131	PCGF5
chr10	95098088	95098099	MYOF
chr10	95098220	95098227	MYOF
chr10	95431811	95431846	FRA10AC1
chr10	95433514	95433566	FRA10AC1
chr10	95435379	95435458	FRA10AC1
chr10	96075204	96075221	PLCE1
chr10	96075204	96075221	NOC3L
chr10	96336839	96336850	HELLS
chr10	97712180	97712182	RP11-248J23.6
chr10	97712180	97712182	ENTPD1-AS1
chr10	97744354	97744401	RP11-248J23.6
chr10	97744354	97744401	CC2D2B
chr10	97744354	97744401	RP11-690P14.4

chr10	97744354	97744401	ENTPD1-AS1
chr10	97959239	97959289	BLNK
chr10	97959239	97959289	ZNF518A
chr10	98978996	98979006	ARHGAP19
chr10	98978996	98979006	ARHGAP19-SLIT1
chr10	99179228	99179252	AL355490.1
chr10	99179228	99179252	RP11-452K12.7
chr10	99184558	99184650	AL355490.1
chr10	99185431	99185496	AL355490.1
chr10	99393104	99393344	MORN4
chr10	99393104	99393344	PI4K2A
chr10	99393104	99393344	PI4K2A
chr10	99514484	99514509	ZFYVE27
chr10	99628991	99629761	GOLGA7B
chr10	99628991	99629761	CRTAC1
chr10	99628991	99629761	GOLGA7B
chr10	102259884	102259916	SEC31B
chr10	102268218	102268226	SEC31B
chr10	102268218	102268226	NDUFB8
chr10	102268218	102268226	NDUFB8
chr10	102849294	102849662	TLX1NB
chr10	102882536	102883624	HUG1
chr10	102882536	102883624	TLX1NB
chr10	104913048	104913061	NT5C2
chr10	105348481	105348696	NEURL1
chr10	105348481	105348696	SH3PXD2A
chr10	105437839	105437911	SH3PXD2A
chr10	106045693	106045725	GSTO2
chr10	106977602	106977603	SORCS3
chr10	111701407	111701427	RP11-451M19.3
chr10	111702418	111702618	RP11-451M19.3
chr10	111706977	111707034	RP11-451M19.3
chr10	111711602	111711723	RP11-451M19.3
chr10	111713514	111713633	RP11-451M19.3
chr10	115332419	115332490	HABP2
chr10	115674684	115675055	AL162407.1
chr10	115674684	115675055	NHLRC2
chr10	118643742	118643825	ENO4
chr10	118643742	118643825	KIAA1598
chr10	121558822	121558833	INPP5F
chr10	122340410	122340495	PPAPDC1A
chr10	122357823	122358209	C10orf85
chr10	124787050	124787108	ACADSB
chr10	128191354	128191403	C10orf90
chr10	128335134	128335206	C10orf90
chr10	128358810	128359049	C10orf90
chr10	131308884	131309525	AL355531.2

chr10	131308884	131309525	MGMT
chr10	133607899	133608375	AL450307.1
chr10	134062534	134062612	STK32C
chr10	135342643	135342661	AL161645.2
chr10	135342643	135342661	CYP2E1
chr10	135342643	135342661	SPRN
chr11	783680	784063	AP006621.5
chr11	914523	914569	CHID1
chr11	1295812	1296033	AC136297.1
chr11	1295812	1296033	TOLLIP
chr11	1852683	1853126	SYT8
chr11	3012266	3012445	AC131971.1
chr11	3012266	3012445	NAP1L4
chr11	5691164	5691177	TRIM5
chr11	6149840	6150706	OR56B3P
chr11	6149840	6150706	RP11-290F24.3
chr11	6173006	6173818	OR52B1P
chr11	6173006	6173818	RP11-290F24.3
chr11	7717221	7717257	OVCH2
chr11	8716094	8716202	RPL27A
chr11	8716094	8716202	RP11-152H18.3
chr11	8716094	8716202	ST5
chr11	8719471	8719511	RPL27A
chr11	8719471	8719511	ST5
chr11	9481747	9482010	AC132192.1
chr11	9481747	9482010	ZNF143
chr11	10529699	10529773	MTRNR2L8
chr11	11747060	11747257	AC131935.1
chr11	18041196	18041285	RP1-59M18.2
chr11	18041196	18041285	TPH1
chr11	18162157	18162159	RP11-113D6.6
chr11	18210442	18210917	AC090099.2
chr11	18210442	18210917	GLTPP1
chr11	18210442	18210917	RP11-113D6.6
chr11	19799013	19799063	NAV2
chr11	19799472	19799528	NAV2
chr11	19799895	19799897	NAV2
chr11	33902649	33903017	AC132216.1
chr11	33902649	33903017	LMO2
chr11	34918308	34918402	APIP
chr11	35150091	35150095	AL356215.1
chr11	35150191	35150215	AL356215.1
chr11	36692826	36692831	C11orf74
chr11	45793136	45793265	CTD-2210P24.4
chr11	45793544	45793776	CTD-2210P24.4
chr11	46385924	46385928	DGKZ
chr11	57308979	57309030	SMTNL1

chr11	58672918	58673134	AP001652.1
chr11	58672918	58673134	GLYATL1
chr11	58674714	58674868	AP001652.1
chr11	58674714	58674868	GLYATL1
chr11	59705928	59706003	OOSP1
chr11	59710320	59710501	OOSP1
chr11	59712642	59712739	OOSP1
chr11	59712795	59712807	OOSP1
chr11	59969016	59969352	MS4A4E
chr11	61546281	61546354	MYRF
chr11	61546281	61546354	TMEM258
chr11	61735576	61736070	AP003733.1
chr11	62985553	62985560	SLC22A25
chr11	62985553	62985560	SLC22A10
chr11	63129852	63129855	SLC22A10
chr11	64217017	64217124	AP003774.4
chr11	64217369	64217644	AP003774.4
chr11	64948239	64948543	AP003068.23
chr11	64948239	64948543	CAPN1
chr11	64948696	64949149	AP003068.23
chr11	64948696	64949149	CAPN1
chr11	65222728	65222802	AP000769.1
chr11	65222908	65223052	AP000769.1
chr11	65233556	65233674	AP000769.1
chr11	65358665	65358730	AP001362.1
chr11	65358665	65358730	EHBP1L1
chr11	65834877	65835010	RP11-1167A19.2
chr11	65834877	65835010	SF3B2
chr11	65836852	65836957	RP11-1167A19.2
chr11	66290643	66290646	CTD-3074O7.11
chr11	66290643	66290646	BBS1
chr11	66290643	66290646	ZDHHC24
chr11	66963718	66963993	AP001885.1
chr11	66963718	66963993	KDM2A
chr11	67371785	67372540	C11orf72
chr11	69587254	69587718	AP001888.1
chr11	70187083	70187203	AP000487.6
chr11	70187083	70187203	PPFIA1
chr11	70213612	70213735	PPFIA1
chr11	70268609	70268698	CTTN
chr11	70341954	70341959	SHANK2
chr11	70343094	70343113	SHANK2
chr11	70753927	70754197	SHANK2
chr11	70757122	70757134	SHANK2
chr11	70757234	70757255	SHANK2
chr11	70779278	70779316	SHANK2
chr11	70779371	70779383	SHANK2

chr11	70788699	70788710	SHANK2
chr11	70788943	70788948	SHANK2
chr11	70789169	70789189	SHANK2
chr11	70790046	70790056	SHANK2
chr11	70794410	70794462	SHANK2
chr11	70796204	70796208	SHANK2
chr11	70798846	70798972	SHANK2
chr11	71615061	71615111	RP11-849H4.2
chr11	71615061	71615111	OR7E126P
chr11	72065345	72065433	CLPB
chr11	73415209	73415260	RAB6A
chr11	73471093	73471140	RAB6A
chr11	73933716	73933778	PPME1
chr11	74980231	74980239	ARRB1
chr11	76705232	76705270	ACER3
chr11	77184416	77184868	DKFZP434E1119
chr11	77184416	77184868	PAK1
chr11	85563928	85564524	AP000974.1
chr11	86889499	86889585	TMEM135
chr11	89804314	89804336	RP11-529A4.10
chr11	89804314	89804336	TRIM49C
chr11	89804864	89804961	RP11-529A4.10
chr11	89804864	89804961	TRIM49C
chr11	89806077	89806558	RP11-529A4.10
chr11	89806077	89806558	TRIM49C
chr11	92292576	92292733	FAT3
chr11	94915322	94915338	SESN3
chr11	94915322	94915338	RP11-712B9.2
chr11	99428944	99429065	CNTN5
chr11	102369248	102369478	RP11-315O6.2
chr11	102552612	102552619	RP11-817J15.3
chr11	102554056	102554308	RP11-817J15.3
chr11	107462484	107462727	AP000889.3
chr11	107462484	107462727	ELMOD1
chr11	107463047	107463186	AP000889.3
chr11	107463047	107463186	ELMOD1
chr11	107582998	107583019	SLN
chr11	107582998	107583019	AP002353.1
chr11	107595119	107595135	AP002353.1
chr11	107643129	107643398	AP001024.2
chr11	107646957	107646962	AP001024.2
chr11	107647303	107647344	AP001024.2
chr11	107650219	107650267	AP001024.1
chr11	107650292	107650492	AP001024.1
chr11	107652270	107652316	AP001024.1
chr11	108130255	108130282	ATM
chr11	108130255	108130282	AP001925.1

chr11	108136458	108136480	ATM
chr11	108136458	108136480	AP001925.1
chr11	111322132	111322134	RP11-794P6.2
chr11	111322132	111322134	POU2AF1
chr11	111324134	111324308	RP11-794P6.2
chr11	111324134	111324308	POU2AF1
chr11	111324961	111325082	RP11-794P6.2
chr11	111324961	111325082	POU2AF1
chr11	111325688	111325813	RP11-794P6.2
chr11	111325688	111325813	POU2AF1
chr11	111326482	111326603	RP11-794P6.2
chr11	111327240	111327264	RP11-794P6.2
chr11	111422058	111422141	LAYN
chr11	112131511	112131624	PLET1
chr11	112131511	112131624	PTS
chr11	112131511	112131624	AP002884.2
chr11	113067915	113067938	NCAM1
chr11	114310843	114310868	REXO2
chr11	114310843	114310868	RP11-212D19.4
chr11	114578110	114578285	NXPE2
chr11	118269753	118269776	RP11-770J1.5
chr11	118269753	118269776	UBE4A
chr11	118305377	118305820	RP11-770J1.4
chr11	118512338	118512502	PHLDB1
chr11	118515365	118515409	PHLDB1
chr11	119992642	119992688	TRIM29
chr11	119994041	119994150	TRIM29
chr11	120040518	120041489	AP000679.2
chr11	120040518	120041489	TRIM29
chr11	120382513	120382596	AP002348.1
chr11	120382513	120382596	GRIK4
chr11	120385694	120385732	AP002348.1
chr11	120385694	120385732	GRIK4
chr11	123301501	123301874	AP000783.1
chr11	123306142	123306175	AP000783.1
chr11	124134528	124134534	OR8G1
chr11	126164364	126164497	TIRAP
chr11	126164364	126164497	RP11-712L6.5
chr11	129936836	129936978	AP003041.2
chr11	129936996	129937066	AP003041.2
chr11	129942608	129942692	AP003041.2
chr11	129942608	129942692	APLP2
chr11	129942743	129942759	AP003041.2
chr11	129942743	129942759	APLP2
chr11	129943981	129944012	AP003041.2
chr11	129943981	129944012	APLP2
chr11	134132079	134132170	ACAD8

chr11	134225909	134225948	GLB1L2
chr11	134855393	134856643	AP003062.1
chr12	147052	147059	AC026369.1
chr12	147257	147377	AC026369.1
chr12	147257	147377	FAM138D
chr12	960859	960948	WNK1
chr12	2958397	2958691	ITFG2
chr12	2958397	2958691	AC005841.1
chr12	2964062	2964612	ITFG2
chr12	2964062	2964612	AC005841.1
chr12	2966121	2966213	ITFG2
chr12	2966121	2966213	AC005841.1
chr12	6459125	6459190	SCNN1A
chr12	10856122	10856218	YBX3
chr12	12509717	12509739	LOH12CR2
chr12	13526138	13526372	C12orf36
chr12	13527769	13527940	C12orf36
chr12	13529154	13529339	C12orf36
chr12	19459333	19459440	PLEKHA5
chr12	25381515	25381572	AC087239.1
chr12	25381515	25381572	KRAS
chr12	25384952	25384978	AC087239.1
chr12	25384952	25384978	KRAS
chr12	25390815	25390840	AC087239.1
chr12	25390815	25390840	KRAS
chr12	27071974	27072002	ASUN
chr12	29542439	29542786	OVCH1-AS1
chr12	29639819	29639899	OVCH1-AS1
chr12	29639819	29639899	OVCH1
chr12	31079886	31079939	TSPAN11
chr12	31477778	31478257	AC024940.1
chr12	31477778	31478257	FAM60A
chr12	40805409	40805429	RP11-115F18.1
chr12	40805409	40805429	MUC19
chr12	42599677	42599679	YAF2
chr12	42624050	42624093	AC020629.1
chr12	42624050	42624093	YAF2
chr12	42627985	42628075	AC020629.1
chr12	42627985	42628075	YAF2
chr12	48413844	48413952	RP1-228P16.4
chr12	48413844	48413952	RP1-228P16.5
chr12	48418603	48418892	RP1-228P16.4
chr12	48418603	48418892	RP1-228P16.5
chr12	48499185	48499236	PFKM
chr12	48499185	48499236	SENP1
chr12	48592180	48592575	DKFZP779L1853
chr12	48759940	48760392	AC024257.1

chr12	48759940	48760392	RP11-370I10.6
chr12	49121264	49121342	LINC00935
chr12	49129871	49129932	LINC00935
chr12	49152992	49153114	LINC00935
chr12	49158634	49158778	LINC00935
chr12	49159390	49159438	LINC00935
chr12	49976811	49976892	PRPF40B
chr12	49976811	49976892	FAM186B
chr12	50690489	50690512	AC140061.12
chr12	50691374	50691718	AC140061.12
chr12	51619860	51619875	AC139768.1
chr12	51620045	51620085	AC139768.1
chr12	51796945	51797073	SLC4A8
chr12	52203959	52204336	RP11-923I11.3
chr12	52203959	52204336	AC068987.1
chr12	52203959	52204336	SCN8A
chr12	52922915	52922980	AC055736.1
chr12	54067542	54067596	ATP5G2
chr12	57644957	57644976	STAC3
chr12	57644957	57644976	R3HDM2
chr12	57644957	57644976	RP11-123K3.4
chr12	57810198	57810536	AC126614.1
chr12	57810198	57810536	R3HDM2
chr12	57937658	57937663	DCTN2
chr12	57937919	57937927	DCTN2
chr12	59197114	59197131	RP11-362K2.2
chr12	59199723	59199841	RP11-362K2.2
chr12	59205704	59205815	RP11-362K2.2
chr12	59206196	59206430	RP11-362K2.2
chr12	59917185	59917236	RP11-272B17.2
chr12	59917484	59917692	RP11-272B17.2
chr12	62996723	62997118	RP11-631N16.2
chr12	62996723	62997118	C12orf61
chr12	65090189	65090329	AC025262.1
chr12	65090189	65090329	RASSF3
chr12	66291518	66291555	AC090673.2
chr12	66291518	66291555	HMGA2
chr12	66298638	66298794	AC090673.2
chr12	66298638	66298794	HMGA2
chr12	66317959	66317967	AC090673.2
chr12	66317959	66317967	HMGA2
chr12	69186336	69186590	AC124890.1
chr12	69186336	69186590	SLC35E3
chr12	71441430	71441459	CTD-2021H9.3
chr12	71441430	71441459	CTD-2021H9.2
chr12	71509630	71509738	CTD-2021H9.3
chr12	71511850	71511962	CTD-2021H9.3

chr12	71532931	71532942	TSPAN8
chr12	75796797	75796883	GLIPR1L2
chr12	77214838	77214867	ZDHHC17
chr12	77949297	77949313	AC073528.1
chr12	77949297	77949313	RP1-34H18.1
chr12	77950325	77950336	AC073528.1
chr12	77950325	77950336	RP1-34H18.1
chr12	77950556	77950568	AC073528.1
chr12	77950556	77950568	RP1-34H18.1
chr12	78537174	78537197	NAV3
chr12	80198742	80198758	AC073569.1
chr12	80198742	80198758	PPP1R12A
chr12	80855627	80855704	PTPRQ
chr12	80855763	80855833	PTPRQ
chr12	80856197	80856261	PTPRQ
chr12	80856304	80856345	PTPRQ
chr12	80856358	80856439	PTPRQ
chr12	80856508	80856558	PTPRQ
chr12	80858971	80859015	PTPRQ
chr12	80859253	80859315	PTPRQ
chr12	81695964	81695972	RP11-121G22.3
chr12	81695964	81695972	PPFIA2
chr12	86421882	86421962	MGAT4C
chr12	89853793	89853815	POC1B
chr12	94671640	94671913	RP11-1105G2.3
chr12	94671640	94671913	PLXNC1
chr12	95770990	95771145	RP11-167N24.6
chr12	95774634	95774672	RP11-167N24.6
chr12	96197041	96197048	RP11-536G4.2
chr12	96197041	96197048	RP11-536G4.1
chr12	96216867	96217047	RP11-536G4.2
chr12	96216867	96217047	RP11-536G4.1
chr12	98880881	98880966	RP11-181C3.1
chr12	98887209	98887262	RP11-181C3.1
chr12	98896361	98896390	RP11-181C3.1
chr12	98897538	98897610	RP11-181C3.1
chr12	99189417	99189423	ANKS1B
chr12	101111304	101111325	ANO4
chr12	101127552	101127635	ANO4
chr12	101133632	101133883	ANO4
chr12	103558189	103558215	RP11-328J6.1
chr12	103558189	103558215	RP11-552I14.1
chr12	103558469	103558541	RP11-328J6.1
chr12	103558469	103558541	RP11-552I14.1
chr12	103561816	103561913	RP11-328J6.1
chr12	103561816	103561913	RP11-552I14.1
chr12	104235268	104235412	RP11-650K20.3

chr12	104237953	104237984	RP11-650K20.3
chr12	104237953	104237984	RP11-642P15.1
chr12	105444470	105444485	ALDH1L2
chr12	106893854	106893960	POLR3B
chr12	106893854	106893960	RP11-144F15.1
chr12	106901540	106901588	POLR3B
chr12	106901540	106901588	RP11-144F15.1
chr12	107086844	107086937	RFX4
chr12	107086844	107086937	RP11-144F15.1
chr12	110390345	110390359	GIT2
chr12	110390345	110390359	TCHP
chr12	113275607	113275614	RPH3A
chr12	113279775	113279778	RPH3A
chr12	115800959	115800978	RP11-116D17.1
chr12	115801870	115802107	RP11-116D17.1
chr12	117739821	117739876	NOS1
chr12	118112088	118112161	KSR2
chr12	118112194	118112196	KSR2
chr12	120137621	120137707	RP1-127H14.3
chr12	120137621	120137707	CIT
chr12	121409319	121409723	HNF1A-AS1
chr12	121409319	121409723	AC079602.1
chr12	123101954	123101980	KNTC1
chr12	123491972	123491975	PITPNM2
chr12	125323790	125323792	SCARB1
chr12	125324519	125324533	SCARB1
chr12	131451577	131451610	GPR133
chr12	131478759	131478812	GPR133
chr12	131487299	131487382	GPR133
chr12	131514221	131514373	AC078925.1
chr12	131514221	131514373	GPR133
chr12	131514416	131514769	AC078925.1
chr12	131514416	131514769	GPR133
chr12	131555398	131555435	GPR133
chr12	131780941	131781585	AC092850.1
chr12	131780941	131781585	RP11-495K9.3
chr12	133049334	133049468	MUC8
chr12	133049473	133049712	MUC8
chr12	133049717	133049752	MUC8
chr12	133049757	133049792	MUC8
chr12	133049797	133049832	MUC8
chr12	133049837	133049995	MUC8
chr12	133050159	133050296	MUC8
chr12	133050580	133050726	MUC8
chr12	133310602	133310657	ANKLE2
chr12	133660701	133660792	ZNF140
chr12	133721490	133721552	ZNF10

chr12	133721490	133721552	ZNF268
chr12	133721490	133721552	CTD-2140B24.4
chr12	133787351	133787680	AC226150.4
chr13	19759450	19759482	RP11-408E5.4
chr13	19761260	19761466	RP11-408E5.4
chr13	20268779	20268961	AL354808.2
chr13	20268779	20268961	PSPC1
chr13	21978459	21978481	ZDHHC20
chr13	24322243	24322294	AL139080.1
chr13	24322243	24322294	MIPEP
chr13	24323858	24323901	AL139080.1
chr13	24323858	24323901	MIPEP
chr13	24334672	24334712	MIPEP
chr13	25802678	25802719	MTMR6
chr13	26442061	26442083	AL138815.1
chr13	26442061	26442083	ATP8A2
chr13	26442265	26442273	AL138815.1
chr13	26442265	26442273	ATP8A2
chr13	26445330	26445338	AL138815.1
chr13	26445330	26445338	ATP8A2
chr13	26447960	26447973	AL138815.1
chr13	26447960	26447973	ATP8A2
chr13	26452422	26452679	AL138815.2
chr13	26452422	26452679	AL138815.1
chr13	26452422	26452679	ATP8A2
chr13	26455046	26455095	AL138815.1
chr13	26455046	26455095	ATP8A2
chr13	28813770	28813833	PAN3
chr13	36939724	36939741	SPG20
chr13	36939724	36939741	SPG20OS
chr13	36939867	36939980	SPG20
chr13	36939867	36939980	SPG20OS
chr13	36942335	36942361	SPG20
chr13	36942335	36942361	SPG20OS
chr13	37541873	37541944	ALG5
chr13	40252229	40252281	COG6
chr13	41111138	41111323	AL133318.1
chr13	41794673	41794779	MTRF1
chr13	41837411	41837441	MTRF1
chr13	41936867	41936920	NAA16
chr13	43895228	43895235	ENOX1
chr13	44430576	44430758	CCDC122
chr13	46716861	46716935	LCP1
chr13	46844469	46844506	FAM206BP
chr13	46844469	46844506	LRRC63
chr13	46844600	46844757	FAM206BP
chr13	46844600	46844757	LRRC63

chr13	50007495	50007529	AL136218.1
chr13	50007495	50007529	CAB39L
chr13	50008222	50008359	AL136218.1
chr13	50008222	50008359	CAB39L
chr13	50018122	50018167	AL136218.1
chr13	50018122	50018167	CAB39L
chr13	50020549	50020554	AL136218.1
chr13	50020549	50020554	SETDB2
chr13	50121285	50121307	RCBTB1
chr13	64320934	64321323	AL445989.1
chr13	74988682	74989077	AL355390.1
chr13	74992909	74992947	AL355390.1
chr13	77692475	77692654	MYCBP2
chr13	88325735	88325829	SLITRK5
chr13	95921675	95921677	ABCC4
chr13	99498774	99498810	DOCK9
chr13	99667701	99667862	DOCK9
chr13	100529852	100529935	CLYBL
chr13	101833446	101833688	NALCN
chr13	109538788	109538810	MYO16
chr13	111521643	111522137	LINC00346
chr13	111919174	111919234	ARHGEF7
chr13	112278343	112278457	RP11-65D24.2
chr13	112278343	112278457	RP11-65D24.1
chr13	112297493	112297546	RP11-65D24.2
chr13	112324745	112324773	RP11-65D24.2
chr13	113755563	113755589	AL137002.1
chr13	113756399	113756422	AL137002.1
chr13	113756600	113756608	AL137002.1
chr13	114471109	114471238	TMEM255B
chr13	114502791	114502830	TMEM255B
chr13	114549939	114550056	GAS6
chr14	20919470	20919611	RP11-203M5.7
chr14	20919470	20919611	OSGEP
chr14	21501079	21501313	RP11-998D10.1
chr14	21501079	21501313	RNASE13
chr14	21501079	21501313	NDRG2
chr14	21501079	21501313	TPPP2
chr14	21779073	21779076	RPGRIP1
chr14	22689792	22689837	TRAV35
chr14	22690088	22690371	TRAV35
chr14	23005092	23005151	TRAJ8
chr14	23025867	23025993	AE000662.93
chr14	23025867	23025993	AE000662.92
chr14	23026459	23026595	AE000662.92
chr14	23027629	23027664	AE000662.92
chr14	23286463	23286497	AL135998.1

chr14	23286463	23286497	SLC7A7
chr14	23291720	23291750	AL135998.1
chr14	23291720	23291750	SLC7A7
chr14	23747269	23747309	HOMEZ
chr14	23788944	23789025	BCL2L2-PABPN1
chr14	24408734	24408931	DHRS4-AS1
chr14	24408734	24408931	DHRS4-AS1
chr14	24970802	24970942	RP11-80A15.1
chr14	25287292	25287315	STXBP6
chr14	29242014	29242061	C14orf23
chr14	29242566	29242639	C14orf23
chr14	29247064	29247197	C14orf23
chr14	29247312	29247349	C14orf23
chr14	29261086	29261467	C14orf23
chr14	29282179	29282284	C14orf23
chr14	29282179	29282284	RP11-966I7.3
chr14	31059860	31059966	G2E3
chr14	31803607	31803662	HEATR5A
chr14	31803607	31803662	RP11-176H8.1
chr14	32414132	32414314	RP11-187E13.1
chr14	32419295	32419351	RP11-187E13.1
chr14	32476113	32476205	RP11-187E13.2
chr14	32487714	32487785	RP11-187E13.2
chr14	39516571	39516642	SEC23A
chr14	50262951	50263015	NEMF
chr14	50300810	50300836	AL627171.1
chr14	50300810	50300836	NEMF
chr14	50311520	50311552	AL627171.1
chr14	50311520	50311552	NEMF
chr14	50459022	50459043	C14orf182
chr14	50459496	50459591	C14orf182
chr14	50472312	50472517	C14orf182
chr14	50550369	50550727	C14orf183
chr14	50550369	50550727	RP11-58E21.5
chr14	50550369	50550727	RP11-58E21.7
chr14	50551848	50552016	C14orf183
chr14	50551848	50552016	RP11-58E21.5
chr14	50551848	50552016	RP11-58E21.7
chr14	50555839	50555913	C14orf183
chr14	50555839	50555913	RP11-58E21.5
chr14	50555839	50555913	RP11-58E21.7
chr14	50558196	50558493	C14orf183
chr14	50559288	50559361	C14orf183
chr14	52383155	52383225	RP11-463J10.2
chr14	52383155	52383225	GNG2
chr14	52384052	52384166	RP11-463J10.2
chr14	52384052	52384166	GNG2

chr14	52436014	52436247	RP11-463J10.3
chr14	52436014	52436247	AL358333.1
chr14	52436014	52436247	GNG2
chr14	57192620	57192705	RP11-1085N6.3
chr14	57196954	57196990	RP11-1085N6.3
chr14	57672580	57672750	AL391152.1
chr14	57672580	57672750	EXOC5
chr14	58755636	58755797	AL132989.1
chr14	58755636	58755797	RP11-349A22.5
chr14	58755636	58755797	C14orf37
chr14	61119519	61119849	SIX1
chr14	61187841	61187862	SIX4
chr14	61448374	61448409	RP11-193F5.1
chr14	61448374	61448409	SLC38A6
chr14	62037416	62037478	RP11-47I22.3
chr14	62037416	62037478	RP11-47I22.4
chr14	62117680	62117735	RP11-47I22.3
chr14	62117680	62117735	RP11-47I22.4
chr14	62120341	62120497	RP11-47I22.3
chr14	62120341	62120497	RP11-47I22.4
chr14	64423148	64423164	SYNE2
chr14	66424458	66424573	CTD-2014B16.3
chr14	66455288	66455368	CTD-2014B16.3
chr14	66456398	66456461	CTD-2014B16.3
chr14	68053085	68053173	PIGH
chr14	68053085	68053173	PLEKHH1
chr14	70037483	70037749	CCDC177
chr14	71683031	71683462	AC004817.1
chr14	71683031	71683462	RP6-91H8.1
chr14	72457259	72457642	AC005477.1
chr14	72457259	72457642	RGS6
chr14	74185710	74185712	ELMSAN1
chr14	74341287	74341330	PTGR2
chr14	74341287	74341330	RP5-1021I20.4
chr14	74544899	74544943	CCDC176
chr14	74544899	74544943	AC005484.5
chr14	74544899	74544943	ALDH6A1
chr14	75158879	75159031	AREL1
chr14	75158879	75159031	AC007956.1
chr14	75165467	75165559	AREL1
chr14	75165467	75165559	AC007956.1
chr14	75327914	75327925	PROX2
chr14	75705632	75705892	RP11-293M10.1
chr14	75735883	75735972	RP11-293M10.1
chr14	75735883	75735972	RP11-293M10.2
chr14	76720557	76720670	RP11-361H10.3
chr14	76720557	76720670	GPATCH2L

chr14	77276560	77276597	ANGEL1
chr14	77295434	77295473	C14orf166B
chr14	77607313	77607753	ZDHHC22
chr14	77607313	77607753	AC007375.1
chr14	77607313	77607753	RP11-463C8.4
chr14	77607313	77607753	TMEM63C
chr14	78228010	78228033	AC008372.1
chr14	78228010	78228033	C14orf178
chr14	81360923	81361021	CEP128
chr14	85995027	85995202	RP11-497E19.1
chr14	85995027	85995202	RP11-497E19.2
chr14	85995348	85995468	RP11-497E19.1
chr14	85995348	85995468	RP11-497E19.2
chr14	87379867	87379903	RP11-322L20.1
chr14	87380986	87381027	RP11-322L20.1
chr14	87386605	87386678	RP11-322L20.1
chr14	87387809	87387952	RP11-322L20.1
chr14	90095459	90095480	RP11-944C7.1
chr14	90095459	90095480	RP11-33N16.3
chr14	90097711	90097910	RP11-944C7.1
chr14	90097711	90097910	RP11-33N16.3
chr14	90302907	90303005	EFCAB11
chr14	90302907	90303005	RP11-33N16.3
chr14	90303360	90303489	EFCAB11
chr14	90303360	90303489	RP11-33N16.3
chr14	91710702	91710843	CTD-2547L24.3
chr14	91710702	91710843	GPR68
chr14	91717109	91717422	CTD-2547L24.3
chr14	91717109	91717422	GPR68
chr14	92040510	92040956	AL133373.1
chr14	92344272	92344386	FBLN5
chr14	92537556	92537578	ATXN3
chr14	93532530	93532703	ITPK1
chr14	94407974	94408038	RP11-131H24.4
chr14	94407974	94408038	ASB2
chr14	94408546	94408605	RP11-131H24.4
chr14	94408546	94408605	ASB2
chr14	94410235	94410388	RP11-131H24.4
chr14	94410235	94410388	ASB2
chr14	94581664	94581672	IFI27
chr14	95078727	95078784	SERPINA3
chr14	95078727	95078784	RP11-986E7.7
chr14	95983344	95983634	RP11-1070N10.3
chr14	96556882	96557403	C14orf132
chr14	96742336	96742719	DKFZP434O1614
chr14	98217971	98218219	RP11-204N11.1
chr14	98435619	98435884	C14orf64

chr14	98444314	98444461	C14orf64
chr14	101295066	101295152	AL117190.2
chr14	101295066	101295152	MEG3
chr14	101295370	101295537	AL117190.2
chr14	101295370	101295537	MEG3
chr14	101359265	101359615	AL117190.3
chr14	102196792	102196824	RP11-796G6.2
chr14	102196792	102196824	RP11-1029J19.5
chr14	102197694	102197805	RP11-796G6.2
chr14	102198125	102198313	RP11-796G6.2
chr14	102198573	102198583	RP11-796G6.2
chr14	103411511	103411668	CDC42BPB
chr14	104177700	104178161	AL049840.1
chr14	104177700	104178161	XRCC3
chr14	104710618	104710782	C14orf144
chr14	104710618	104710782	RP11-260M19.2
chr14	105220055	105220225	SIVA1
chr14	105634692	105634757	JAG2
chr14	105634692	105634757	RP11-44N21.4
chr14	105935406	105935497	RP11-521B24.5
chr14	105935406	105935497	MTA1
chr14	106064226	106064317	AL928742.12
chr14	106064226	106064317	IGHE
chr14	106386952	106387117	KIAA0125
chr14	106388058	106388203	KIAA0125
chr14	106388400	106388506	KIAA0125
chr14	106539079	106539383	IGHV1-8
chr14	106539470	106539515	IGHV1-8
chr14	106552285	106552592	IGHV3-9
chr14	106552684	106552729	IGHV3-9
chr14	107082712	107082728	IGHV4-59
chr15	21004687	21005367	AC012414.1
chr15	22011370	22012050	DKFZP547L112
chr15	22456895	22456919	AC010760.1
chr15	22460344	22460351	AC010760.1
chr15	28195655	28195710	OCA2
chr15	29995564	29995638	TJP1
chr15	31233442	31233456	FAN1
chr15	31233442	31233456	MTMR10
chr15	32776882	32777442	AC135983.2
chr15	35271774	35271977	AC114546.1
chr15	35271774	35271977	ZNF770
chr15	38248873	38248891	TMCO5A
chr15	39544337	39544888	C15orf54
chr15	39544337	39544888	RP11-624L4.1
chr15	40662813	40662848	DISP2
chr15	42302277	42302445	CTD-2382E5.2

chr15	42302277	42302445	PLA2G4E
chr15	42646604	42646651	RP11-164J13.1
chr15	42646604	42646651	CAPN3
chr15	45492925	45493069	SHF
chr15	48483861	48484050	RP11-605F22.2
chr15	48483861	48484050	CTXN2
chr15	48483861	48484050	SLC12A1
chr15	49611169	49611212	GALK2
chr15	49688172	49688294	FAM227B
chr15	49688441	49688639	FAM227B
chr15	50200242	50200290	ATP8B4
chr15	52727841	52727867	MYO5A
chr15	54336622	54336644	UNC13C
chr15	56379666	56379707	RFX7
chr15	59088276	59088365	FAM63B
chr15	59439899	59440054	C15ORF31
chr15	59439899	59440054	MYO1E
chr15	62930726	62930817	RP11-625H11.1
chr15	62930726	62930817	TLN2
chr15	62932359	62932561	RP11-625H11.1
chr15	62932359	62932561	TLN2
chr15	62936716	62936765	RP11-625H11.1
chr15	62936716	62936765	TLN2
chr15	64557965	64558064	CSNK1G1
chr15	64557965	64558064	CTD-2116N17.1
chr15	66874554	66874586	RP11-321F6.1
chr15	66956700	66956790	RP11-321F6.1
chr15	66960679	66960792	RP11-321F6.1
chr15	66976337	66976380	RP11-321F6.1
chr15	66977608	66977667	RP11-321F6.1
chr15	67001043	67001076	SMAD6
chr15	67435106	67435294	RP11-342M21.2
chr15	67435106	67435294	SMAD3
chr15	73858111	73858118	NPTN
chr15	74420766	74421077	RP11-247C2.2
chr15	74420766	74421077	ISLR2
chr15	74421306	74421432	RP11-247C2.2
chr15	74421306	74421432	ISLR2
chr15	74509613	74509922	RP11-60L3.1
chr15	74509613	74509922	CCDC33
chr15	74510838	74511165	RP11-60L3.1
chr15	74510838	74511165	CCDC33
chr15	74536075	74536084	CCDC33
chr15	75917056	75917176	CTD-2026K11.3
chr15	75917056	75917176	SNUPN
chr15	75970841	75971218	CSPG4
chr15	75970841	75971218	AC105020.1

chr15	76030377	76030994	AC019294.1
chr15	76030377	76030994	RP11-24M17.4
chr15	76030377	76030994	DNM1P35
chr15	76298084	76298150	NRG4
chr15	78831032	78831283	AC027228.1
chr15	80215116	80215508	ST20
chr15	80215116	80215508	ST20-MTHFS
chr15	80215116	80215508	C15orf37
chr15	80215116	80215508	C15ORF37
chr15	80672443	80672495	RP11-210M15.2
chr15	80690286	80690348	RP11-210M15.2
chr15	80695844	80695898	RP11-210M15.2
chr15	81188634	81188702	RP11-351M8.2
chr15	81188634	81188702	RP11-351M8.1
chr15	81188634	81188702	KIAA1199
chr15	81189595	81189680	RP11-351M8.2
chr15	81189595	81189680	RP11-351M8.1
chr15	81189595	81189680	KIAA1199
chr15	81193296	81193306	RP11-351M8.2
chr15	81193296	81193306	RP11-351M8.1
chr15	81193296	81193306	KIAA1199
chr15	82384075	82384281	RP11-597K23.2
chr15	82387823	82387869	RP11-597K23.2
chr15	83209693	83209729	RP11-152F13.10
chr15	83209693	83209729	RP11-379H8.1
chr15	83217406	83217521	RP11-152F13.10
chr15	83217406	83217521	RP11-379H8.1
chr15	83217406	83217521	CPEB1
chr15	83230930	83230980	RP11-379H8.1
chr15	83230930	83230980	CPEB1
chr15	83837974	83837983	HDGFRP3
chr15	84278022	84278050	SH3GL3
chr15	85469771	85469785	SLC28A1
chr15	89174333	89174407	AEN
chr15	89999051	89999077	RHCG
chr15	90798987	90799117	TTLL13
chr15	90798987	90799117	RP11-697E2.6
chr15	90799373	90799529	TTLL13
chr15	90799373	90799529	RP11-697E2.6
chr15	90801975	90802187	TTLL13
chr15	90801975	90802187	RP11-697E2.6
chr15	90805238	90805423	TTLL13
chr15	90805238	90805423	RP11-697E2.6
chr15	90805724	90805901	TTLL13
chr15	90805724	90805901	RP11-697E2.6
chr15	90806198	90806377	TTLL13
chr15	90806198	90806377	RP11-697E2.6

chr15	90806987	90807290	TTLL13
chr15	90806987	90807290	RP11-697E2.6
chr15	90807952	90808199	TTLL13
chr15	90807952	90808199	RP11-697E2.6
chr15	90892076	90892429	GABARAPL3
chr15	93749423	93749968	AC112693.2
chr15	96831280	96831660	AC016251.1
chr15	96831280	96831660	NR2F2-AS1
chr15	96904487	96904900	AC087477.1
chr15	96904487	96904900	RP11-522B15.3
chr15	98286684	98286713	LINC00923
chr15	98301880	98301891	LINC00923
chr15	98417183	98417515	LINC00923
chr15	98462784	98462829	ARRDC4
chr15	100038447	100038578	AC015660.1
chr15	100038447	100038578	MEF2A
chr15	100353178	100353279	CTD-2054N24.2
chr16	68602	68789	WASH4P
chr16	68602	68789	Z84812.4
chr16	322365	322493	RGS11
chr16	322365	322493	ARHGDIG
chr16	832863	832926	MSLNL
chr16	1031252	1031269	RP11-161M6.2
chr16	1031252	1031269	AC009041.2
chr16	1031252	1031269	LMF1
chr16	1031643	1031663	AC009041.2
chr16	1130832	1130833	SSTR5
chr16	1131019	1131037	SSTR5
chr16	1433524	1433933	UNKL
chr16	1445645	1445813	UNKL
chr16	1455907	1456081	UNKL
chr16	1458464	1458549	UNKL
chr16	1476120	1476384	C16orf91
chr16	1478413	1478506	C16orf91
chr16	1479202	1479345	C16orf91
chr16	1670639	1670692	CRAMP1L
chr16	2031634	2031762	AC005606.1
chr16	2031634	2031762	TBL3
chr16	2031634	2031762	NOXO1
chr16	3197340	3197481	CASP16
chr16	3197804	3197920	CASP16
chr16	3198325	3198399	CASP16
chr16	3198937	3199040	CASP16
chr16	3199696	3199809	CASP16
chr16	3421816	3421902	MTRNR2L4
chr16	3421816	3421902	NAA60
chr16	9761094	9761231	RP11-297M9.1

chr16	9770531	9770692	RP11-297M9.1
chr16	11537275	11537316	CTD-3088G3.8
chr16	11561008	11561010	CTD-3088G3.6
chr16	11561008	11561010	CTD-3088G3.8
chr16	12062000	12062021	AC007216.2
chr16	12062000	12062021	RP11-166B2.1
chr16	12062209	12062333	AC007216.2
chr16	12062209	12062333	RP11-166B2.1
chr16	12181628	12182042	RP11-276H1.3
chr16	12181628	12182042	SNX29
chr16	12183626	12183795	RP11-276H1.3
chr16	12183626	12183795	SNX29
chr16	14564842	14565054	AC092291.2
chr16	14564842	14565054	PARN
chr16	15694834	15694909	C16orf45
chr16	15694834	15694909	KIAA0430
chr16	15696357	15696599	C16orf45
chr16	15696357	15696599	KIAA0430
chr16	19598715	19598720	C16orf62
chr16	20499542	20499949	AC137056.1
chr16	23649878	23649913	PALB2
chr16	27300131	27300274	CTD-3203P2.2
chr16	27301566	27301667	CTD-3203P2.2
chr16	27366341	27366354	IL4R
chr16	28510168	28510242	APOBR
chr16	29823290	29823329	AC009133.14
chr16	29823290	29823329	AC009133.20
chr16	29823290	29823329	PRRT2
chr16	29823290	29823329	MAZ
chr16	30681440	30681622	FBRS
chr16	34256757	34257278	CTD-2144E22.5
chr16	34256757	34257278	CTD-2144E22.6
chr16	52039536	52039546	C16orf97
chr16	52082667	52082719	C16orf97
chr16	52085725	52085739	C16orf97
chr16	54279597	54279713	RP11-324D17.1
chr16	54280735	54280873	RP11-324D17.1
chr16	56662971	56662987	AC026461.1
chr16	56666114	56666150	AC026461.1
chr16	56666114	56666150	MT1M
chr16	57723713	57723975	GPR97
chr16	57723713	57723975	RP11-405F3.4
chr16	59772016	59772068	RP11-105C20.2
chr16	59772685	59772756	RP11-105C20.2
chr16	59773754	59773837	RP11-105C20.2
chr16	59785865	59785871	RP11-105C20.2
chr16	66518447	66519052	BEAN1

chr16	66518447	66519052	RP11-403P17.5
chr16	66518447	66519052	AC132186.1
chr16	66757278	66757330	DYNC1LI2
chr16	66757278	66757330	RP11-63M22.2
chr16	66920450	66920453	PDP2
chr16	67244457	67244873	LRRC29
chr16	67244457	67244873	AC040160.1
chr16	67257729	67257830	LRRC29
chr16	67257729	67257830	AC040160.1
chr16	71840589	71840631	AP1G1
chr16	71841704	71841726	AP1G1
chr16	75728367	75728423	AC025287.1
chr16	75728367	75728423	TERF2IP
chr16	75729841	75730013	AC025287.1
chr16	75729841	75730013	TERF2IP
chr16	75730736	75730841	AC025287.1
chr16	75730736	75730841	TERF2IP
chr16	75733913	75734089	AC025287.1
chr16	75733913	75734089	TERF2IP
chr16	79015647	79015739	PIH1
chr16	79015647	79015739	WWOX
chr16	80673567	80673633	CDYL2
chr16	85204882	85205413	CTC-786C10.1
chr16	85218725	85218969	CTC-786C10.1
chr16	85391174	85391249	RP11-680G10.1
chr16	85392948	85393055	RP11-680G10.1
chr16	87728869	87729747	AC010536.1
chr16	87728869	87729747	JPH3
chr16	88928034	88928106	TRAPPC2L
chr16	88928034	88928106	PABPN1L
chr16	89749194	89749250	CDK10
chr16	89749194	89749250	RP11-368I7.4
chr16	89751709	89752044	CDK10
chr16	89751709	89752044	RP11-368I7.4
chr16	90095397	90095750	GAS8
chr16	90095397	90095750	C16orf3
chr16	90160431	90160487	TUBB8P7
chr16	90160750	90160858	TUBB8P7
chr16	90160937	90161047	TUBB8P7
chr17	813049	813172	RP11-676J12.7
chr17	813049	813172	NXN
chr17	813934	813989	RP11-676J12.7
chr17	813934	813989	NXN
chr17	914385	914475	ABR
chr17	2310298	2310524	AC006435.1
chr17	2310298	2310524	METTL16
chr17	2317689	2317765	AC006435.1

chr17	2317689	2317765	METTL16
chr17	2318482	2318651	AC006435.1
chr17	2318482	2318651	METTL16
chr17	3828081	3828095	ATP2A3
chr17	4545518	4545558	ALOX15
chr17	6593281	6593352	SLC13A5
chr17	6780107	6780206	ALOX12P2
chr17	6780107	6780206	AC027763.2
chr17	6784368	6784381	ALOX12P2
chr17	6784368	6784381	AC027763.2
chr17	6829480	6829602	ALOX12P2
chr17	6829480	6829602	AC027763.2
chr17	6888780	6888859	RP11-589P10.7
chr17	6888780	6888859	AC027763.2
chr17	6905803	6905939	RP11-589P10.7
chr17	6905803	6905939	ALOX12
chr17	6905803	6905939	AC027763.2
chr17	6915515	6915517	RP11-589P10.7
chr17	6915515	6915517	AC027763.2
chr17	7517269	7517283	AC007421.1
chr17	7517269	7517283	FXR2
chr17	8262810	8263223	AC135178.1
chr17	10049175	10049546	AC000003.2
chr17	10049175	10049546	GAS7
chr17	16227667	16227705	PIGL
chr17	16640852	16640989	CCDC144A
chr17	16640852	16640989	RP11-219A15.1
chr17	17714069	17714194	SREBF1
chr17	17714069	17714194	RAI1
chr17	19041448	19041452	AC007952.1
chr17	19041448	19041452	GRAPL
chr17	22023388	22023462	MTRNR2L1
chr17	25783670	25783900	KSR1
chr17	26638667	26638677	AC061975.10
chr17	26638667	26638677	KRT18P55
chr17	26642194	26642323	AC061975.10
chr17	26642194	26642323	KRT18P55
chr17	29634905	29634928	EVI2B
chr17	29634905	29634928	CTD-2370N5.3
chr17	29634905	29634928	NF1
chr17	29898165	29898380	AC003101.1
chr17	29899104	29899193	AC003101.1
chr17	29902530	29902658	MIR365B
chr17	29902530	29902658	AC003101.1
chr17	30470044	30470048	AC090616.2
chr17	30470044	30470048	RHOT1
chr17	30506379	30506885	AC116407.2

chr17	30506379	30506885	RHOT1
chr17	32581900	32581926	AC005549.3
chr17	36458712	36458833	MRPL45
chr17	36905801	36906391	CTB-58E17.5
chr17	36905801	36906391	PCGF2
chr17	38152954	38152962	PSMD3
chr17	38517235	38517393	CTD-2267D19.3
chr17	38517235	38517393	GJD3
chr17	39344697	39344713	KRTAP9-1
chr17	41994679	41995023	FAM215A
chr17	41994679	41995023	RP11-527L4.5
chr17	42015731	42015765	RP11-527L4.2
chr17	42015929	42016082	RP11-527L4.2
chr17	42355793	42355825	AC003043.1
chr17	47448194	47448297	RP11-81K2.1
chr17	47450375	47450448	RP11-81K2.1
chr17	47554239	47554297	RP11-81K2.1
chr17	48049541	48049548	DLX4
chr17	48593220	48593454	MYCBPAP
chr17	48610236	48610346	EPN3
chr17	50939750	50939831	AC102948.2
chr17	50975794	50975866	AC102948.2
chr17	50976691	50976703	AC102948.2
chr17	56282912	56283065	MKS1
chr17	58642747	58642800	RP11-15E18.2
chr17	58642747	58642800	RP11-15E18.4
chr17	58654558	58654699	RP11-15E18.4
chr17	58663669	58663748	RP11-15E18.4
chr17	60778723	60778739	RP11-156L14.1
chr17	60778723	60778739	10-Mar
chr17	65027509	65027514	AC005544.1
chr17	65027509	65027514	CACNG4
chr17	65027554	65028198	AC005544.1
chr17	65027554	65028198	CACNG4
chr17	70036465	70036617	AC007461.1
chr17	70036465	70036617	RP11-84E24.2
chr17	70036465	70036617	SOX9-AS1
chr17	71166793	71166870	SSTR2
chr17	71166793	71166870	RP11-143K11.5
chr17	72866073	72866114	FDXR
chr17	73138852	73138868	HN1
chr17	75878008	75878472	FLJ45079
chr17	76220514	76220634	BIRC5
chr17	76220514	76220634	AC087645.1
chr17	76220639	76220740	BIRC5
chr17	76220639	76220740	AC087645.1
chr17	77015620	77015698	C1QTNF1-AS1

chr17	77016084	77016227	C1QTNF1-AS1
chr17	77022002	77022017	C1QTNF1-AS1
chr17	77022002	77022017	C1QTNF1
chr17	78978059	78978529	AC127496.1
chr17	78978059	78978529	CHMP6
chr17	79206115	79206318	ENTHD2
chr17	79206115	79206318	AC027601.1
chr17	79380724	79380736	RP11-1055B8.7
chr17	79383860	79383878	RP11-1055B8.7
chr17	79385351	79385408	RP11-1055B8.7
chr17	79386905	79386959	RP11-1055B8.7
chr17	79395735	79395765	RP11-1055B8.7
chr17	79396630	79396633	RP11-1055B8.7
chr17	80214341	80214727	SLC16A3
chr17	80214341	80214727	CSNK1D
chr17	80214341	80214727	AC132872.2
chr17	80866173	80866283	TBCD
chr18	49501	49557	RP11-683L23.1
chr18	693170	693175	ENOSF1
chr18	6511501	6511609	C18orf64
chr18	6513638	6513725	C18orf64
chr18	6575989	6576103	C18orf64
chr18	6588810	6588866	C18orf64
chr18	6589446	6589474	C18orf64
chr18	6590534	6590543	C18orf64
chr18	8336437	8336454	AP001094.1
chr18	8336437	8336454	PTPRM
chr18	8336459	8336555	AP001094.1
chr18	8336459	8336555	PTPRM
chr18	8336980	8337038	AP001094.1
chr18	8336980	8337038	PTPRM
chr18	12658242	12658342	AP005482.1
chr18	12658550	12658562	AP005482.1
chr18	12658573	12658630	AP005482.1
chr18	19048687	19048748	GREB1L
chr18	19678334	19678606	RP11-595B24.2
chr18	20799218	20799220	CABLES1
chr18	20799218	20799220	TMEM241
chr18	21089569	21089641	C18orf8
chr18	21089569	21089641	NPC1
chr18	21229466	21229486	ANKRD29
chr18	22811435	22811494	ZNF521
chr18	30350917	30351071	AC012123.1
chr18	30350917	30351071	KLHL14
chr18	30353278	30353297	AC012123.1
chr18	32401437	32401439	DTNA
chr18	34809532	34809584	KIAA1328

chr18	39639833	39639858	PIK3C3
chr18	44139133	44139147	LOXHD1
chr18	47807534	47807586	MBD1
chr18	48577724	48577785	RP11-729L2.2
chr18	48577724	48577785	SMAD4
chr18	54388778	54388814	WDR7
chr18	54558064	54558179	WDR7
chr18	55111608	55111659	ONECUT2
chr18	55134392	55134452	ONECUT2
chr18	59785780	59785890	PIGN
chr18	59790740	59790777	PIGN
chr18	60491423	60491432	AC015989.1
chr18	60491423	60491432	PHLPP1
chr18	60492654	60492724	AC015989.1
chr18	60492654	60492724	PHLPP1
chr18	61609566	61609601	AC009802.1
chr18	66373517	66373645	TMX3
chr18	66567321	66567474	RP11-861L17.3
chr18	66567321	66567474	CCDC102B
chr18	66569425	66569477	RP11-861L17.3
chr18	66569425	66569477	CCDC102B
chr18	67728602	67728636	RTTN
chr18	68003616	68003740	RP11-484N16.1
chr18	68003616	68003740	RP11-41O4.1
chr18	68019215	68019287	RP11-41O4.1
chr18	69446812	69447012	RP11-723G8.2
chr18	70829180	70829208	RP11-169F17.1
chr18	70840184	70840197	RP11-169F17.1
chr18	70867888	70868024	RP11-169F17.1
chr18	70923048	70923151	RP11-169F17.1
chr18	73119952	73120028	RP11-321M21.3
chr18	73971141	73971284	RP11-94B19.4
chr18	73971772	73971798	RP11-94B19.4
chr18	74240909	74240920	LINC00908
chr18	74242341	74242421	LINC00908
chr18	74269932	74270021	LINC00908
chr18	74273820	74273834	LINC00908
chr18	74314695	74314745	LINC00908
chr18	74322802	74322861	LINC00908
chr18	74507493	74507614	RP11-162A12.2
chr18	74528711	74528765	RP11-162A12.2
chr18	77275812	77276057	AC018445.1
chr18	77275812	77276057	NFATC1
chr18	77906042	77906137	AC139100.2
chr18	77906305	77906427	AC139100.2
chr18	77920399	77920486	AC139100.2
chr18	77920399	77920486	PARD6G

chr18	77926702	77926820	AC139100.2
chr18	77926702	77926820	PARD6G
chr18	77926901	77927019	AC139100.2
chr18	77926901	77927019	PARD6G
chr18	77933763	77933818	AC139100.2
chr18	77933763	77933818	PARD6G
chr18	77935969	77936011	AC139100.2
chr18	77935969	77936011	PARD6G
chr19	538678	538768	CDC34
chr19	538932	539009	CDC34
chr19	641178	641268	FGF22
chr19	661356	661402	RNF126
chr19	999601	999621	AC004528.1
chr19	999914	999952	AC004528.1
chr19	999914	999952	AC004528.4
chr19	1071703	1071765	HMHA1
chr19	1416456	1416540	DAZAP1
chr19	1440838	1441275	AC027307.3
chr19	3610665	3611074	CACTIN-AS1
chr19	3610665	3611074	CACTIN
chr19	3611227	3611351	CACTIN-AS1
chr19	3611227	3611351	CACTIN
chr19	4042083	4042496	AC016586.1
chr19	5772104	5772446	CATSPERD
chr19	6279784	6279795	MLLT1
chr19	7011817	7012062	AC025278.1
chr19	8066936	8067000	ELAVL1
chr19	9732229	9732321	C19orf82
chr19	9737460	9737536	C19orf82
chr19	9738392	9738482	C19orf82
chr19	9742904	9743048	C19orf82
chr19	10751715	10751757	AC011475.1
chr19	10751715	10751757	SLC44A2
chr19	10752056	10752093	AC011475.1
chr19	10752056	10752093	SLC44A2
chr19	10922429	10922440	DNM2
chr19	11650567	11650569	CNN1
chr19	12163976	12164074	ZNF878
chr19	12167083	12167127	ZNF878
chr19	12358092	12359493	ZNF44
chr19	12360793	12360853	ZNF44
chr19	12361586	12361618	ZNF44
chr19	12882631	12882692	HOOK2
chr19	14502203	14502304	CD97
chr19	14834747	14834836	AC090427.1
chr19	14834747	14834836	ZNF333
chr19	14834839	14834892	AC090427.1

chr19	14834839	14834892	ZNF333
chr19	14834895	14834918	AC090427.1
chr19	14834895	14834918	ZNF333
chr19	14835005	14835121	AC090427.1
chr19	14835005	14835121	ZNF333
chr19	15549389	15551455	WIZ
chr19	16254543	16254584	HSH2D
chr19	17531770	17531905	MVB12A
chr19	17531770	17531905	CTD-2521M24.6
chr19	18653698	18653759	FKBP8
chr19	19439879	19439896	MAU2
chr19	20047769	20047782	AC007204.1
chr19	20047769	20047782	AC007204.2
chr19	20047924	20048018	AC007204.1
chr19	20047924	20048018	AC007204.2
chr19	20048512	20048639	AC007204.1
chr19	20048512	20048639	AC007204.2
chr19	20973438	20973578	ZNF66
chr19	20991839	20991922	ZNF66
chr19	21120116	21120167	ZNF85
chr19	21265152	21265154	ZNF714
chr19	24009964	24010851	RP11-255H23.4
chr19	24009964	24010851	RP11-255H23.2
chr19	24009964	24010851	RPSAP58
chr19	31640362	31640757	AC020952.1
chr19	31796563	31796669	TSHZ3
chr19	32896383	32896451	AC007773.3
chr19	32896383	32896451	AC007773.2
chr19	32896383	32896451	DPY19L3
chr19	34971874	34971931	WTIP
chr19	34972121	34972626	WTIP
chr19	35597290	35597736	AC020907.1
chr19	36389374	36389434	NFKBID
chr19	36389621	36389724	NFKBID
chr19	36393095	36393185	NFKBID
chr19	36602668	36602706	OVOL3
chr19	38578396	38578427	SIPA1L3
chr19	38622294	38622375	SIPA1L3
chr19	38826783	38826890	CATSPERG
chr19	39119143	39119208	EIF3K
chr19	39121342	39121460	EIF3K
chr19	39930212	39930481	AC011500.1
chr19	39930212	39930481	SUPT5H
chr19	39930580	39930624	AC011500.1
chr19	39930580	39930624	SUPT5H
chr19	39931930	39932082	AC011500.1
chr19	39931930	39932082	SUPT5H

chr19	41072952	41073080	SPTBN4
chr19	42747003	42747458	AC006486.1
chr19	42747003	42747458	AC006486.9
chr19	43863300	43863418	CD177
chr19	44143879	44143942	CADM4
chr19	44754318	44754400	ZNF235
chr19	44754318	44754400	ZNF233
chr19	45453408	45453457	CTB-129P6.11
chr19	45454193	45454283	CTB-129P6.11
chr19	45457152	45457241	CTB-129P6.11
chr19	45705699	45705765	AC005779.2
chr19	45705699	45705765	AC006126.3
chr19	45705699	45705765	MARK4
chr19	45719934	45720151	AC006126.3
chr19	45719934	45720151	EXOC3L2
chr19	45719934	45720151	MARK4
chr19	45842445	45842639	L47234.1
chr19	45842445	45842639	KLC3
chr19	46498339	46498418	CCDC61
chr19	46498683	46498762	CCDC61
chr19	46995339	46995444	PNMAL2
chr19	46995339	46995444	PPP5D1
chr19	47050313	47050398	PPP5D1
chr19	47050313	47050398	AC011551.3
chr19	47079856	47079974	PPP5D1
chr19	47079856	47079974	AC011551.3
chr19	47092403	47092492	PPP5D1
chr19	47092403	47092492	AC011551.3
chr19	47092821	47092903	PPP5D1
chr19	47092821	47092903	AC011551.3
chr19	48364059	48364769	TPRX2P
chr19	48707454	48707500	CTC-453G23.8
chr19	48707454	48707500	ZNF114
chr19	48707454	48707500	CARD8
chr19	49559433	49559852	CGB7
chr19	49559433	49559852	NTF4
chr19	49560378	49560453	CGB7
chr19	49560378	49560453	NTF4
chr19	49945535	49945560	SLC17A7
chr19	49982947	49983002	CTD-3148I10.9
chr19	49982947	49983002	FLT3LG
chr19	49982947	49983002	CTD-3148I10.15
chr19	50003781	50004614	MIR150
chr19	50003781	50004614	hsa-mir-150
chr19	50728081	50728085	MYH14
chr19	50818019	50818247	KCNC3
chr19	51377552	51377581	AC037199.1

chr19	51377552	51377581	KLK2
chr19	51979838	51979853	CEACAM18
chr19	52095889	52096304	AC018755.1
chr19	52097202	52097574	AC018755.1
chr19	54103573	54103688	CTB-167G5.5
chr19	54104519	54104626	CTB-167G5.5
chr19	54105251	54105417	CTB-167G5.5
chr19	54106606	54106643	CTB-167G5.5
chr19	54800046	54800104	LILRA3
chr19	54801927	54802229	LILRA3
chr19	54803016	54803318	LILRA3
chr19	54803466	54803753	LILRA3
chr19	54803943	54803978	LILRA3
chr19	54804140	54804221	LILRA3
chr19	54804604	54804606	LILRA3
chr19	54971319	54971327	LENG8
chr19	55147372	55147527	LILRB1
chr19	55147372	55147527	AC009892.10
chr19	55150145	55150189	AC009892.10
chr19	55458837	55458873	NLRP7
chr19	55459011	55459018	NLRP7
chr19	55856095	55856484	SUV420H2
chr19	55856095	55856484	AC020922.1
chr19	56662314	56662350	AC024580.1
chr19	56662314	56662350	ZNF444
chr19	56662571	56663250	AC024580.1
chr19	56662571	56663250	ZNF444
chr19	56751773	56751906	ZSCAN5A
chr19	56751773	56751906	ZSCAN5D
chr19	56756591	56756794	ZSCAN5A
chr19	56756591	56756794	ZSCAN5D
chr19	56757685	56757835	ZSCAN5A
chr19	56757685	56757835	ZSCAN5D
chr19	56758103	56758863	ZSCAN5A
chr19	56758103	56758863	ZSCAN5D
chr19	56784204	56784288	AC006116.20
chr19	56784204	56784288	ZSCAN5A
chr19	56794364	56794389	AC006116.20
chr19	56794364	56794389	ZSCAN5A
chr19	56795536	56795575	AC006116.20
chr19	56795536	56795575	ZSCAN5A
chr19	56796367	56796393	AC006116.20
chr19	56796367	56796393	ZSCAN5A
chr19	56797751	56797884	AC006116.20
chr19	56797751	56797884	ZSCAN5A
chr19	56806436	56806522	AC006116.20
chr19	56806436	56806522	ZSCAN5A

chr19	56807328	56807336	AC006116.20
chr19	56807328	56807336	ZSCAN5A
chr19	56807705	56807731	AC006116.20
chr19	56807705	56807731	ZSCAN5A
chr19	56808874	56808900	AC006116.20
chr19	56808874	56808900	ZSCAN5A
chr19	56813337	56813464	AC006116.20
chr19	56813337	56813464	ZSCAN5A
chr19	56814211	56814257	AC006116.20
chr19	56814211	56814257	ZSCAN5A
chr19	56816143	56816199	AC006116.20
chr19	56816143	56816199	ZSCAN5A
chr19	56821493	56821575	AC006116.20
chr19	56821493	56821575	ZSCAN5A
chr19	56960602	56960706	ZNF667
chr19	56978358	56978435	ZNF667
chr19	56981283	56981424	ZNF667
chr19	58203868	58204128	AC004017.1
chr19	58203868	58204128	ZNF551
chr19	58203868	58204128	AC003006.7
chr19	58513479	58514414	CTD-2368P22.1
chr19	58513479	58514414	ZNF606
chr19	58514579	58514704	CTD-2368P22.1
chr19	58514579	58514704	ZNF606
chr19	58515751	58515789	CTD-2368P22.1
chr19	58517275	58517367	CTD-2368P22.1
chr19	58520699	58520818	CTD-2368P22.1
chr20	1316935	1317330	AL136531.1
chr20	1316935	1317330	SDCBP2-AS1
chr20	2124430	2124542	STK35
chr20	2903905	2903931	PTPRA
chr20	4053172	4053311	RP11-352D3.2
chr20	4055394	4055595	RP11-352D3.2
chr20	11008541	11008554	C20orf187
chr20	11008810	11008936	C20orf187
chr20	11009839	11009854	C20orf187
chr20	13597663	13597770	TASP1
chr20	19738296	19738679	AL121761.2
chr20	19738296	19738679	RP1-122P22.2
chr20	23330015	23330039	RP3-322G13.7
chr20	23330015	23330039	AL096677.1
chr20	23330259	23330262	RP3-322G13.7
chr20	23330259	23330262	AL096677.1
chr20	23334039	23334066	NXT1
chr20	23334039	23334066	AL096677.1
chr20	25207122	25207370	AL035252.1
chr20	25207122	25207370	ENTPD6

chr20	25400320	25400441	GINS1
chr20	25745605	25745637	FAM182B
chr20	29625873	29625984	FRG1B
chr20	29630699	29630707	FRG1B
chr20	29633898	29633910	FRG1B
chr20	30309001	30309041	AL160175.1
chr20	30309001	30309041	BCL2L1
chr20	30309125	30309134	AL160175.1
chr20	30309125	30309134	BCL2L1
chr20	30525985	30526015	TTLL9
chr20	30531569	30531581	TTLL9
chr20	31189469	31189498	RP11-410N8.4
chr20	31196112	31196504	RP11-410N8.4
chr20	31823091	31823387	AL121901.1
chr20	33150292	33150369	PIGU
chr20	33721908	33721946	EDEM2
chr20	33814542	33814787	EDEM2
chr20	33814542	33814787	MMP24-AS1
chr20	33814542	33814787	MMP24
chr20	34756461	34756487	AL121895.1
chr20	34756461	34756487	EPB41L1
chr20	34756535	34756540	AL121895.1
chr20	34756535	34756540	EPB41L1
chr20	34758148	34758180	AL121895.1
chr20	34758148	34758180	EPB41L1
chr20	40111177	40111191	CHD6
chr20	43080751	43080813	C20orf62
chr20	43234346	43234385	PKIG
chr20	43234346	43234385	Z97053.1
chr20	43248095	43248126	PKIG
chr20	43248095	43248126	Z97053.1
chr20	44075127	44075525	AL031663.2
chr20	44095910	44095954	AL031663.1
chr20	44107438	44107452	AL031663.1
chr20	44107438	44107452	WFDC2
chr20	44501328	44501464	ZSWIM3
chr20	44600761	44600815	ZNF335
chr20	45005343	45005345	ELMO2
chr20	45010845	45010880	ELMO2
chr20	45947365	45947937	AL031666.2
chr20	45947365	45947937	ZMYND8
chr20	55045656	55045715	RTFDC1
chr20	55934021	55934095	MTRNR2L3
chr20	55934021	55934095	RAE1
chr20	56807967	56807978	PPP4R1L
chr20	56810296	56810430	PPP4R1L
chr20	56811210	56811330	PPP4R1L

chr20	56813267	56813367	PPP4R1L
chr20	56814297	56814458	PPP4R1L
chr20	56814716	56814901	PPP4R1L
chr20	56815571	56815666	PPP4R1L
chr20	56818568	56818761	PPP4R1L
chr20	56820839	56820860	PPP4R1L
chr20	56821014	56821308	PPP4R1L
chr20	56822332	56822562	PPP4R1L
chr20	56823171	56823329	PPP4R1L
chr20	56825943	56826008	PPP4R1L
chr20	56826813	56826920	PPP4R1L
chr20	56846419	56846561	PPP4R1L
chr20	56847825	56847931	PPP4R1L
chr20	56861370	56861502	PPP4R1L
chr20	56884282	56884326	PPP4R1L
chr20	56884475	56884481	PPP4R1L
chr20	57210133	57210420	MGC4294
chr20	60293804	60294226	CDH4
chr20	60293804	60294226	RP11-429E11.3
chr20	60917246	60917355	LAMA5
chr20	61273320	61273712	RP11-93B14.6
chr20	61274366	61274407	RP11-93B14.6
chr20	61274366	61274407	SLCO4A1
chr20	62042370	62042375	KCNQ2
chr20	62332542	62332740	ARFRP1
chr20	62474779	62475273	AL158091.1
chr20	62585007	62585495	AL118506.1
chr20	62585007	62585495	UCKL1
chr21	14741931	14741956	AL050302.1
chr21	14743755	14743756	AL050302.1
chr21	14743816	14743916	AL050302.1
chr21	14745372	14745386	AL050302.1
chr21	15051911	15052330	AL050303.1
chr21	18814099	18814161	C21orf37
chr21	18816401	18816518	C21orf37
chr21	18821183	18821226	C21orf37
chr21	22582881	22583010	NCAM2
chr21	22757924	22757928	NCAM2
chr21	27937126	27937144	AP001597.1
chr21	27937126	27937144	CYYR1
chr21	30397064	30397098	RP1-100J12.1
chr21	30397064	30397098	USP16
chr21	30973548	30973549	GRIK1-AS2
chr21	30973548	30973549	BACH1
chr21	30973548	30973549	GRIK1
chr21	33765532	33765717	C21orf119
chr21	34157189	34157230	C21orf49

chr21	34160923	34161012	C21orf49
chr21	34169249	34169356	C21orf62
chr21	34169249	34169356	C21orf49
chr21	34224059	34224091	C21orf49
chr21	34258240	34258293	C21orf49
chr21	34537977	34537997	C21orf54
chr21	34540748	34540886	C21orf54
chr21	34542001	34542138	C21orf54
chr21	34542446	34542474	C21orf54
chr21	34947431	34947451	AP000304.1
chr21	34947431	34947451	SON
chr21	34947431	34947451	DONSON
chr21	34947631	34947645	AP000304.1
chr21	34947631	34947645	SON
chr21	34947631	34947645	DONSON
chr21	36228710	36228744	RUNX1
chr21	37402368	37402428	AP000688.1
chr21	37858193	37858849	AP000695.1
chr21	37858193	37858849	PSMD4P1
chr21	37858193	37858849	AP000695.4
chr21	37858193	37858849	CLDN14
chr21	38888740	38888973	AP001421.1
chr21	38888740	38888973	DYRK1A
chr21	40969631	40969916	C21orf88
chr21	40969631	40969916	B3GALT5
chr21	40977887	40977929	C21orf88
chr21	40977887	40977929	B3GALT5
chr21	40978157	40978274	C21orf88
chr21	40978157	40978274	B3GALT5
chr21	40981510	40981592	C21orf88
chr21	40981510	40981592	B3GALT5
chr21	40984224	40984292	C21orf88
chr21	40984224	40984292	B3GALT5
chr21	43528406	43528492	C21orf128
chr21	43528406	43528492	UMODL1
chr21	43816051	43816251	TMPRSS3
chr21	43830225	43830239	UBASH3A
chr21	44579355	44579877	AP001631.10
chr21	44581199	44581362	AP001631.10
chr21	45587818	45588263	AP001055.1
chr21	45590937	45591047	AP001055.1
chr21	45593517	45593580	AP001055.1
chr21	45879823	45880557	LRRC3DN
chr21	45937677	45937718	C21orf90
chr21	45937677	45937718	TSPEAR
chr21	45938142	45938215	C21orf90
chr21	45938142	45938215	TSPEAR

chr21	45938511	45938592	C21orf90
chr21	45938511	45938592	TSPEAR
chr21	46354129	46354283	C21orf67
chr21	46354727	46355011	C21orf67
chr21	46355542	46355805	C21orf67
chr21	46357454	46357519	C21orf67
chr21	46359508	46359582	C21orf67
chr21	46492796	46492927	SSR4P1
chr21	46492796	46492927	AP001579.1
chr21	46511596	46511693	PRED57
chr21	46511596	46511693	ADARB1
chr21	46512040	46512202	PRED57
chr21	46512040	46512202	ADARB1
chr21	46515410	46515493	PRED57
chr21	46515410	46515493	ADARB1
chr21	46520728	46521105	ADARB1
chr21	46520728	46521105	PRED58
chr21	46525754	46525899	ADARB1
chr21	46525754	46525899	PRED58
chr21	46528690	46528753	ADARB1
chr21	46528690	46528753	PRED58
chr21	46534727	46534873	ADARB1
chr21	46534727	46534873	PRED58
chr21	47183565	47183690	PCBP3
chr21	47183565	47183690	PRED60
chr21	47185994	47186037	PCBP3
chr21	47185994	47186037	PRED60
chr21	47187934	47188060	PCBP3
chr21	47187934	47188060	PRED60
chr21	47189949	47190005	PCBP3
chr21	47189949	47190005	PRED60
chr21	47347679	47347697	PRED62
chr21	47347679	47347697	PCBP3
chr21	47351505	47351619	PRED62
chr21	47351505	47351619	PCBP3
chr21	47352373	47352477	PRED62
chr21	47352373	47352477	PCBP3
chr21	47612391	47612516	AP001468.1
chr21	47612391	47612516	LSS
chr21	47612518	47613088	AP001468.1
chr21	47612518	47613088	LSS
chr21	47613567	47613673	AP001468.1
chr21	47613567	47613673	LSS
chr21	47976316	47976342	DIP2A
chr22	17602819	17602929	AC006946.15
chr22	17603459	17603545	AC006946.15
chr22	17605545	17605661	AC006946.15

chr22	17611252	17611344	AC006946.15
chr22	18167375	18167460	BCL2L13
chr22	18721540	18721567	AC008132.1
chr22	18723545	18723603	AC008132.1
chr22	18724072	18724096	AC008132.1
chr22	18727043	18727129	AC008132.1
chr22	18732023	18732088	AC008132.1
chr22	18734087	18734148	AC008132.1
chr22	18734614	18734647	AC008132.1
chr22	18739382	18739404	AC008132.1
chr22	19048275	19048468	DGCR2
chr22	20692622	20692649	FAM230A
chr22	20695279	20695337	FAM230A
chr22	20695806	20695830	FAM230A
chr22	20698777	20698863	FAM230A
chr22	20703776	20703841	FAM230A
chr22	20705845	20705906	FAM230A
chr22	20705845	20705906	USP41
chr22	20706372	20706545	FAM230A
chr22	20706372	20706545	USP41
chr22	20708630	20710986	FAM230A
chr22	20708630	20710986	USP41
chr22	20739003	20739029	USP41
chr22	21358030	21358052	THAP7-AS1
chr22	21358030	21358052	AC002472.1
chr22	21358030	21358052	TUBA3FP
chr22	21360568	21360736	THAP7-AS1
chr22	21360568	21360736	AC002472.1
chr22	21360568	21360736	TUBA3FP
chr22	22901775	22901814	LL22NC03-63E9.3
chr22	22905268	22905377	LL22NC03-63E9.3
chr22	22906072	22906221	LL22NC03-63E9.3
chr22	23585005	23585129	BCR
chr22	24238016	24238100	AP000350.4
chr22	24376423	24376617	GSTT1
chr22	24376822	24376998	GSTT1
chr22	24379361	24379511	GSTT1
chr22	24381700	24381787	GSTT1
chr22	24382973	24383081	GSTT1
chr22	24384120	24384231	GSTT1
chr22	30217881	30218064	ASCC2
chr22	30814212	30814469	KIAA1658
chr22	30814212	30814469	RP4-539M6.19
chr22	30814212	30814469	RNF215
chr22	30814212	30814469	SEC14L2
chr22	31006222	31006247	TCN2
chr22	31478471	31478553	RP3-412A9.16

chr22	31478471	31478553	SMTN
chr22	31743328	31743723	AC005003.1
chr22	35727289	35727388	TOM1
chr22	36023489	36023706	MB
chr22	36023489	36023706	AL049747.1
chr22	36031002	36031026	MB
chr22	36031002	36031026	AL049747.1
chr22	37319837	37319859	CSF2RB
chr22	38044756	38044853	SH3BP1
chr22	38044756	38044853	Z83844.1
chr22	38489921	38489968	BAIAP2L2
chr22	39150647	39150711	SUN2
chr22	39150903	39150905	SUN2
chr22	39656865	39656884	AL031590.1
chr22	39657045	39657063	AL031590.1
chr22	39983748	39983756	CACNA1I
chr22	40052423	40052424	CACNA1I
chr22	40052713	40052714	CACNA1I
chr22	40297243	40297334	GRAP2
chr22	41284433	41284477	XPNPEP3
chr22	41303130	41303194	XPNPEP3
chr22	41673479	41673501	RANGAP1
chr22	41685388	41685407	AL035681.1
chr22	41685668	41685686	AL035681.1
chr22	43305527	43305571	PACSIN2
chr22	43814102	43814122	MPPED1
chr22	44761495	44761596	RP1-32I10.10
chr22	44761725	44761850	RP1-32I10.10
chr22	45126684	45126884	PRR5-ARHGAP8
chr22	45126684	45126884	ARHGAP8
chr22	45126684	45126884	PRR5
chr22	45913473	45913578	FBLN1
chr22	45961846	45961853	FBLN1
chr22	46207578	46207674	ATXN10
chr22	46493807	46493890	FLJ27365
chr22	46494374	46494438	FLJ27365
chr22	46499072	46499129	FLJ27365
chr22	46501289	46501741	FLJ27365
chr22	46505147	46505233	FLJ27365
chr22	46505644	46505890	FLJ27365
chr22	46844455	46844465	CELSR1
chr22	47857442	47857578	LL22NC03-75H12.2
chr22	47859067	47859137	LL22NC03-75H12.2
chr22	47882479	47882528	LL22NC03-75H12.2
chr22	48935196	48935260	CTA-299D3.8
chr22	48935196	48935260	FAM19A5
chr22	48940484	48940737	CTA-299D3.8

chr22	48940484	48940737	FAM19A5
chr22	48942325	48942395	CTA-299D3.8
chr22	48942325	48942395	FAM19A5
chr22	49834737	49834861	C22orf34
chr22	50016805	50017258	C22orf34
chr22	50051053	50051107	C22orf34
chr22	51048142	51048250	MAPK8IP2
chr22	51162555	51162581	SHANK3
chrX	2976652	2976662	ARSF
chrX	3189874	3189959	CXorf28
chrX	3190338	3190413	CXorf28
chrX	3195027	3195134	CXorf28
chrX	3202196	3202222	CXorf28
chrX	3735816	3735819	RP11-706O15.1
chrX	3736483	3736541	RP11-706O15.1
chrX	3747405	3747433	RP11-706O15.1
chrX	3761382	3761523	RP11-706O15.1
chrX	9754282	9754337	GPR143
chrX	11138115	11138165	HCCS
chrX	11138115	11138165	ARHGAP6
chrX	11283990	11284052	ARHGAP6
chrX	11369506	11369520	ARHGAP6
chrX	13770823	13770871	OFD1
chrX	13799034	13799124	GPM6B
chrX	13801898	13802040	GPM6B
chrX	15872811	15872936	AP1S2
chrX	19057380	19057398	GPR64
chrX	20073852	20073950	MAP7D2
chrX	22025264	22025318	SMS
chrX	22116112	22116153	PHEX
chrX	30864094	30864188	TAB3
chrX	38080579	38080696	RP13-43E11.1
chrX	38080579	38080696	SRPX
chrX	38080579	38080696	TM4SF2
chrX	38425575	38425608	TSPAN7
chrX	38425575	38425608	TM4SF2
chrX	40499456	40499572	CXorf38
chrX	41193846	41194022	DDX3X
chrX	41483517	41483534	CASK
chrX	46747025	46747127	CXorf31
chrX	46749728	46749814	CXorf31
chrX	46753959	46754008	CXorf31
chrX	47343004	47343288	CXorf24
chrX	48432859	48433248	AC115618.1
chrX	48432859	48433248	RBM3
chrX	48435853	48435947	RBM3
chrX	48758796	48758820	PQBP1

chrX	51795944	51796063	RP11-114H20.1
chrX	51797091	51797342	RP11-114H20.1
chrX	51942972	51943223	RP11-363G10.2
chrX	51944251	51944370	RP11-363G10.2
chrX	52238903	52239051	XAGE1B
chrX	52260122	52260270	XAGE1A
chrX	52511854	52512002	XAGE1C
chrX	52533062	52533210	XAGE1D
chrX	52545956	52546104	XAGE1E
chrX	55208353	55208427	MTRNR2L10
chrX	56101008	56101235	AL353698.1
chrX	64185333	64185370	ZC4H2
chrX	69635184	69635312	KIF4A
chrX	70756864	70756960	OGT
chrX	71494277	71494285	RPS4X
chrX	71494277	71494285	PIN4
chrX	71700299	71700301	HDAC8
chrX	71709112	71709113	HDAC8
chrX	91675526	91675538	PCDH11X
chrX	91824799	91824801	PCDH11X
chrX	96048615	96048629	DIAPH2
chrX	100268632	100268728	TRMT2B
chrX	103274127	103274222	H2BFM
chrX	107386937	107387062	ATG4A
chrX	107386937	107387062	COL4A6
chrX	109589931	109590137	GNG5P2
chrX	109589931	109590137	AMMECR1
chrX	114953262	114953549	RP1-241P17.4
chrX	114953262	114953549	AC005000.1
chrX	117904228	117904633	RP13-128O4.3
chrX	117904228	117904633	IL13RA1
chrX	118820523	118820612	6-Sep
chrX	122318792	122318852	GRIA3
chrX	128781653	128781894	APLN
chrX	133981913	133982001	FAM122C
chrX	133983197	133983258	FAM122C
chrX	134482679	134482708	ZNF449
chrX	135519163	135519215	GPR112
chrX	135819543	135819623	ARHGEF6
chrX	138774074	138774284	MCF2
chrX	138814595	138814684	ATP11C
chrX	139791962	139791970	LINC00632
chrX	139793786	139793827	LINC00632
chrX	139795682	139795948	LINC00632
chrX	140968647	140968650	MAGEC3
chrX	140982426	140982586	MAGEC3
chrX	148675471	148675569	HSFX2

chrX	151125930	151125958	GABRE
chrX	151127436	151127621	GABRE
chrX	152609656	152609664	ZNF275
chrX	152610129	152610237	ZNF275
chrX	152751358	152751554	U82695.9
chrX	152751358	152751554	HAUS7
chrX	152752088	152752268	U82695.9
chrX	152752088	152752268	HAUS7
chrX	153000798	153000881	ABCD1
chrX	153146127	153146173	LCA10
chrX	153146127	153146173	L1CAM
chrX	153149166	153149351	LCA10
chrX	153149166	153149351	L1CAM
chrX	153149403	153149727	LCA10
chrX	153149403	153149727	L1CAM
chrX	153150817	153150890	LCA10
chrX	153150817	153150890	L1CAM
chrX	153151262	153151431	LCA10
chrX	153151262	153151431	L1CAM
chrX	153152417	153152511	LCA10
chrX	153152417	153152511	L1CAM
chrX	153153951	153154051	LCA10
chrX	153153951	153154051	L1CAM
chrX	153518086	153518106	TEX28
