# Supplementary material for: Twist exome capture allows for lower average sequence coverage in clinical exome sequencing
Source: Hum Genomics. 2023 May 3;17:39. doi: 10.1186/s40246-023-00485-5 (PMC10155375; doi:10.1186/s40246-023-00485-5)
Supplement: Supplementary file 3 — Additional file 3. List of SolveRD consortium members with affiliations. [file 40246_2023_485_MOESM3_ESM.docx]

**Solve-RD consortium author list**

Olaf Riess^[[1]](#endnote-1),^^[[2]](#endnote-2)^, Tobias B. Haack^1^, Holm Graessner^1^,2, Birte Zurek^1^,2,Kornelia Ellwanger^1^,2, Stephan Ossowski^1^,^[[3]](#endnote-3)^, German Demidov^1^, Marc Sturm^1^, Julia M. Schulze-Hentrich^1^, Rebecca Schüle^[[4]](#endnote-4),^^[[5]](#endnote-5)^, Jishu Xu^4,5^, Christoph Kessler^4,5^, Melanie Wayand^4,5^, Matthis Synofzik^4,5^, Carlo Wilke^4,5^, Andreas Traschütz^4,5^, Ludger Schöls^4,5^, Holger Hengel^4,5^, Holger Lerche^[[6]](#endnote-6)^, Josua Kegele^6^, Peter Heutink^4,5^,Han Brunner^[[7]](#endnote-7),^^[[8]](#endnote-8),^^[[9]](#endnote-9)^, Hans Scheffer^7,8^, Nicoline Hoogerbrugge^7,^^[[10]](#endnote-10)^, Alexander Hoischen^7,10,^^[[11]](#endnote-11)^, Peter A.C. ’t Hoen^,10,^^[[12]](#endnote-12)^, Lisenka E.L.M. Vissers^7, 9^, Christian Gilissen^7,10^, Wouter Steyaert^7,10^, Karolis Sablauskas^7^, Richarda M. de Voer^7,10^, Erik-Jan Kamsteeg^7^, Bart van de Warrenburg^9^,^[[13]](#endnote-13)^, Nienke van Os^9,13^, Iris te Paske^7,10^, Erik Janssen^7,10^, Elke de Boer^7, 9^, Marloes Steehouwer^7^, Burcu Yaldiz^7^, Tjitske Kleefstra^7, 9^, Anthony J. Brookes^[[14]](#endnote-14)^, Colin Veal^14^, Spencer Gibson^14^, Vatsalya Maddi^14^, Mehdi Mehtarizadeh^14^, Umar Riaz^14^, Greg Warren^14^, Farid Yavari Dizjikan^14^, Thomas Shorter^14^, Ana Töpf^[[15]](#endnote-15)^, Volker Straub^15^, Chiara Marini Bettolo^15^, Jordi Diaz Manera^15^, Sophie Hambleton^[[16]](#endnote-16)^, Karin Engelhardt^[[17]](#endnote-17)^,Jill Clayton-Smith^[[18]](#endnote-18),^^[[19]](#endnote-19)^, Siddharth Banka^18,19^, Elizabeth Alexander^19^, Adam Jackson^18,19^,Laurence Faivre^[[20]](#endnote-20),^^[[21]](#endnote-21),^^[[22]](#endnote-22),^^[[23]](#endnote-23),^^[[24]](#endnote-24)^, Christel Thauvin^20,21,22,23,24^, Antonio Vitobello^22^, Anne-Sophie Denommé-Pichon^22^, Yannis Duffourd^22,23^, Ange-Line Bruel^22^, Christine Peyron^[[25]](#endnote-25),^^[[26]](#endnote-26)^, Aurore Pélissier^25,26^, Sergi Beltran^[[27]](#endnote-27),^^[[28]](#endnote-28)^, Ivo Glynne Gut^27,28^, Steven Laurie^27^, Davide Piscia^27^, Leslie Matalonga^27^, Anastasios Papakonstantinou^27^, Gemma Bullich^27^, Alberto Corvo^27^, Marcos Fernandez-Callejo^27^, Carles Hernández^27^, Daniel Picó^27^, Ida Paramonov^27^, Hanns Lochmüller^27^, Gulcin Gumus^[[29]](#endnote-29)^, Virginie Bros-Facer^[[30]](#endnote-30)^, Ana Rath^[[31]](#endnote-31)^, Marc Hanauer^31^, David Lagorce^31^,Oscar Hongnat^31^,Maroua Chahdil^31^,Emeline Lebreton^31^, Giovanni Stevanin^[[32]](#endnote-32),^^[[33]](#endnote-33),^^[[34]](#endnote-34),^^[[35]](#endnote-35),^^[[36]](#endnote-36)^, Alexandra Durr^32.33.34.35.^^[[37]](#endnote-37)^, Claire-Sophie Davoine^32.33.34.35.36^, Léna Guillot-Noel^32.33.34.35.36^, Anna Heinzmann^32.33.34.35.^^[[38]](#endnote-38)^, Giulia Coarelli^32.33.34.35.38^, Gisèle Bonne^[[39]](#endnote-39)^, Teresinha Evangelista^39^, Valérie Allamand^39^, Isabelle Nelson^39^, Rabah Ben Yaou^39,^^[[40]](#endnote-40),^^[[41]](#endnote-41)^, Corinne Metay^39,^^[[42]](#endnote-42)^, Bruno Eymard^39,40^, Enzo Cohen^39^, Antonio Atalaia^39^, Tanya Stojkovic^39,40^,Milan Macek Jr.^[[43]](#endnote-43)^, Marek Turnovec^43^, Dana Thomasová^43^, Radka Pourová Kremliková^43^, Vera Franková^43^, Markéta Havlovicová ^43^, Petra Lišková^[[44]](#endnote-44),^^[[45]](#endnote-45)^, Pavla Doležalová^[[46]](#endnote-46)^, Helen Parkinson^[[47]](#endnote-47)^, Thomas Keane^47^, Mallory Freeberg^47^, Coline Thomas^47^, Dylan Spalding^47^, Peter Robinson^[[48]](#endnote-48)^, Daniel Danis^48^, Glenn Robert^[[49]](#endnote-49)^, Alessia Costa^[[50]](#endnote-50)^, Christine Patch^[[51]](#endnote-51),^^[[52]](#endnote-52)^, Mike Hanna^[[53]](#endnote-53)^, Henry Houlden^[[54]](#endnote-54)^, Mary Reilly^53^, Jana Vandrovcova^54^, Stephanie Efthymiou^54^, Heba Morsy^54^, Elisa Cali^54^, Francesca Magrinelli^[[55]](#endnote-55)^, Sanjay M. Sisodiya^[[56]](#endnote-56)^, Jonathan Rohrer^[[57]](#endnote-57)^, Francesco Muntoni^[[58]](#endnote-58),^^[[59]](#endnote-59)^, Irina Zaharieva^58^, Anna Sarkozy^58^, Vincent Timmerman^[[60]](#endnote-60),^^[[61]](#endnote-61)^, Jonathan Baets^[[62]](#endnote-62),^^[[63]](#endnote-63)^, Geert de Vries^61,62^, Jonathan De Winter^61,62,63^, Danique Beijer^60,61,62^, Peter de Jonghe^61,63^, Liedewei Van de Vondel^60,61,62^, Willem De Ridder^61,62,63^, Sarah Weckhuysen^[[64]](#endnote-64),^^[[65]](#endnote-65)^, Vincenzo Nigro^[[66]](#endnote-66),^^[[67]](#endnote-67)^, Margherita Mutarelli^67,^^[[68]](#endnote-68)^, Manuela Morleo^67^, Michele Pinelli^67^, Alessandra Varavallo^67^, Sandro Banfi^66,67^, Annalaura Torella^66^, Francesco Musacchia^66,67^, Giulio Piluso^66^, Alessandra Ferlini^[[69]](#endnote-69)^, Rita Selvatici^69^, Francesca Gualandi^69^, Stefania Bigoni^69^, Rachele Rossi^69^, Marcella Neri^69^, Stefan Aretz^[[70]](#endnote-70),^^[[71]](#endnote-71)^, Isabel Spier^70,71^, Anna Katharina Sommer^70^, Sophia Peters^70^, Carla Oliveira^[[72]](#endnote-72),^^[[73]](#endnote-73),^^[[74]](#endnote-74)^, Jose Garcia Pelaez^72,73,^^[[75]](#endnote-75)^, Ana Rita Matos^72,73,^^[[76]](#endnote-76)^, Celina São José^72,73,75^, Marta Ferreira^72,73,^^[[77]](#endnote-77)^, Irene Gullo^72,73,^^[[78]](#endnote-78)^, Susana Fernandes^72,^^[[79]](#endnote-79)^, Luzia Garrido^[[80]](#endnote-80)^, Pedro Ferreira^72,73,^^[[81]](#endnote-81)^, Fátima Carneiro^72,73,78^, Morris A Swertz^[[82]](#endnote-82)^, Lennart Johansson^82^, Joeri K van der Velde^82^, Gerben van der Vries^82^, Pieter B Neerincx^82^, David Ruvolo^82^, Kristin M Abbott^[[83]](#endnote-83)^, Wilhemina S Kerstjens Frederikse^83,^^[[84]](#endnote-84)^, Eveline Zonneveld-Huijssoon^83,^^[[85]](#endnote-85)^, Dieuwke Roelofs-Prins^82^, Marielle van Gijn^83,85^, Sebastian Köhler^[[86]](#endnote-86)^,Alison Metcalfe^[[87]](#endnote-87),^^[[88]](#endnote-88)^, Alain Verloes^[[89]](#endnote-89),^^[[90]](#endnote-90)^, Séverine Drunat^89,90^, Delphine Heron^[[91]](#endnote-91),^^[[92]](#endnote-92)^, Cyril Mignot^91,^^[[93]](#endnote-93)^, Boris Keren^91^, Jean-Madeleine de Sainte Agathe^91^’, Caroline Rooryck^[[94]](#endnote-94)^, Didier Lacombe^94^, Aurelien Trimouille^[[95]](#endnote-95)^, Manuel Posada De la Paz^[[96]](#endnote-96)^, Eva Bermejo Sánchez^96^, Estrella López Martín^96^, Beatriz Martínez Delgado^96^, F. Javier Alonso García de la Rosa^96^, Andrea Ciolfi^[[97]](#endnote-97)^, Bruno Dallapiccola^97^, Simone Pizzi^97^, Francesca Clementina Radio^97^, Marco Tartaglia^97^, Alessandra Renieri^[[98]](#endnote-98),^^[[99]](#endnote-99),^^[[100]](#endnote-100)^, Simone Furini^98,99^, Chiara Fallerini^98,99^, Elisa Benetti^98,99^, Peter Balicza^[[101]](#endnote-101)^, Maria Judit Molnar^101^, Ales Maver^[[102]](#endnote-102)^, Borut Peterlin^102^, Alexander Münchau^[[103]](#endnote-103)^, Katja Lohmann^[[104]](#endnote-104)^, Rebecca Herzog^103,^^[[105]](#endnote-105)^, Martje Pauly^103,104^, Alfons Macaya^[[106]](#endnote-106),^^[[107]](#endnote-107)^, Ana Cazurro-Gutiérrez^106^, Belén Pérez-Dueñas^106^, Francina Munell^106^, Clara Franco Jarava^[[108]](#endnote-108),^^[[109]](#endnote-109)^, Laura Batlle Masó^[[110]](#endnote-110),^^[[111]](#endnote-111)^, Anna Marcé-Grau^106^, Roger Colobran^108,109,^^[[112]](#endnote-112)^, Andrés Nascimento Osorio^[[113]](#endnote-113)^ , Daniel Natera de Benito^113^, Hanns Lochmüller^[[114]](#endnote-114),^^[[115]](#endnote-115),^^[[116]](#endnote-116)^, Rachel Thompson^116^, Kiran Polavarapu^116^, Bodo Grimbacher^[[117]](#endnote-117),^^[[118]](#endnote-118),^^[[119]](#endnote-119),^^[[120]](#endnote-120),^^[[121]](#endnote-121)^, David Beeson^[[122]](#endnote-122)^, Judith Cossins^122^ ,Peter Hackman^[[123]](#endnote-123)^, Mridul Johari^123^, Marco Savarese^123^, Bjarne Udd^123,^^[[124]](#endnote-124),^^[[125]](#endnote-125)^, Rita Horvath^[[126]](#endnote-126)^, Patrick F. Chinnery^126,^^[[127]](#endnote-127)^, Thiloka Ratnaike^[[128]](#endnote-128)^, Fei Gao^126^, Katherine Schon^126,^^[[129]](#endnote-129)^, Gabriel Capella^[[130]](#endnote-130)^, Laura Valle^130^, Elke Holinski-Feder^[[131]](#endnote-131)^, Andreas Laner^[[132]](#endnote-132)^, Verena Steinke-Lange^131^, Evelin Schröck^[[133]](#endnote-133)^, Andreas Rump^133^,^[[134]](#endnote-134)^, Ayşe Nazlı Başak^[[135]](#endnote-135)^, Dimitri Hemelsoet^[[136]](#endnote-136),^^[[137]](#endnote-137)^, Bart Dermaut^137^^,^^[[138]](#endnote-138),^^[[139]](#endnote-139)^, Nika Schuermans^137,138,139^, Bruce Poppe^137,138,139^, Hannah Verdin^137138^, Davide Mei^[[140]](#endnote-140)^, Annalisa Vetro^140^, Simona Balestrini^140,^^[[141]](#endnote-141)^, Renzo Guerrini^140^, Kristl Claeys^[[142]](#endnote-142),^^[[143]](#endnote-143)^, Gijs W.E. Santen^[[144]](#endnote-144)^, Emilia K. Bijlsma^144^, Mariette J.V. Hoffer^144^, Claudia A.L. Ruivenkamp^144^, Kaan Boztug^[[145]](#endnote-145),^^[[146]](#endnote-146),^^[[147]](#endnote-147),^^[[148]](#endnote-148),^^[[149]](#endnote-149)^, Matthias Haimel^145,146,147^**,** Isabelle Maystadt^[[150]](#endnote-150),^^[[151]](#endnote-151),^ Isabell Cordts^[[152]](#endnote-152)^, Marcus Deschauer^152,^ Ioannis Zaganas^[[153]](#endnote-153)^, Evgenia Kokosali^153^, Mathioudakis Lambros^153^, Athanasios Evangeliou^[[154]](#endnote-154)^, Martha Spilioti^[[155]](#endnote-155)^, Elisabeth Kapaki^[[156]](#endnote-156)^, Mara Bourbouli^156,^ Pasquale Striano^[[157]](#endnote-157),^^[[158]](#endnote-158)^, Federico Zara^158,^^[[159]](#endnote-159)^, Antonella Riva^158,159^, Michele Iacomino^159,^^[[160]](#endnote-160)^, Paolo Uva^160^, Marcello Scala^157,158^, Paolo Scudieri^158,159^**,** Maria-Roberta Cilio^[[161]](#endnote-161)^, Evelina Carpancea^161^, Chantal Depondt^[[162]](#endnote-162)^, Damien Lederer^[[163]](#endnote-163)^, Yves Sznajer^[[164]](#endnote-164)^, Sarah Duerinckx^[[165]](#endnote-165)^, Sandrine Mary^163^, Christel Depienne^[[166]](#endnote-166),^^[[167]](#endnote-167)^, Andreas Roos^[[168]](#endnote-168),^^[[169]](#endnote-169),^^[[170]](#endnote-170)^, Patrick May^[[171]](#endnote-171)^

1. Institute of Medical Genetics and Applied Genomics, University of Tübingen, Tübingen, Germany. [↑](#endnote-ref-1)
2. Centre for Rare Diseases, University of Tübingen, Tübingen, Germany. [↑](#endnote-ref-2)
3. NGS Competence Center Tübingen (NCCT), University of Tübingen, Tübingen, Germany. [↑](#endnote-ref-3)
4. Department of Neurodegeneration, Hertie Institute for Clinical Brain Research (HIH), University of Tübingen, Tübingen, Germany. [↑](#endnote-ref-4)
5. German Center for Neurodegenerative Diseases (DZNE), Tübingen, Germany. [↑](#endnote-ref-5)
6. Department of Neurolgy and Epileptology, Hertie Institute for Clinical Brain Research (HIH), University of Tübingen, Tübingen, Germany. [↑](#endnote-ref-6)
7. Department of Human Genetics, Radboud University Medical Center, Nijmegen, the Netherlands. [↑](#endnote-ref-7)
8. Department of Clinical Genetics, Maastricht University Medical Centre, Maastricht, the Netherlands. [↑](#endnote-ref-8)
9. Donders Institute for Brain, Cognition and Behaviour, Radboud University Medical Center, Nijmegen, the Netherlands. [↑](#endnote-ref-9)
10. Radboud Institute for Molecular Life Sciences, Nijmegen, The Netherlands. [↑](#endnote-ref-10)
11. Department of Internal Medicine and Radboud Center for Infectious Diseases (RCI), Radboud University Medical Center, Nijmegen, the Netherlands. [↑](#endnote-ref-11)
12. Center for Molecular and Biomolecular Informatics, Radboud University Medical Center, Nijmegen, the Netherlands [↑](#endnote-ref-12)
13. Department of Neurology, Radboud University Medical Center, Nijmegen, The Netherlands. [↑](#endnote-ref-13)
14. Department of Genetics and Genome Biology, University of Leicester, Leicester, UK. [↑](#endnote-ref-14)
15. John Walton Muscular Dystrophy Research Centre, Translational and Clinical Research Institute, Newcastle University and Newcastle Hospitals NHS Foundation Trust, Newcastle upon Tyne, UK. [↑](#endnote-ref-15)
16. Primary Immunodeficiency Group, Translational and Clinical Research Institute, Newcastle University and Newcastle upon Tyne Hospitals NHS Foundation Trust, Newcastle upon Tyne, UK. [↑](#endnote-ref-16)
17. Primary Immunodeficiency Group, Translational and Clinical Research Institute, Newcastle University, Newcastle upon Tyne, UK. [↑](#endnote-ref-17)
18. Division of Evolution, Infection and Genomics, School of Biological Sciences, Faculty of Biology, Medicine and Health, University of Manchester, Manchester M13 9WL, UK. [↑](#endnote-ref-18)
19. Manchester Centre for Genomic Medicine, St Mary's Hospital, Manchester University Hospitals NHS Foundation Trust, Health Innovation Manchester, Manchester M13 9WL, UK. [↑](#endnote-ref-19)
20. Dijon University Hospital, Genetics Department, Dijon, France [↑](#endnote-ref-20)
21. Dijon University Hospital, Centre of Reference for Rare Diseases: Development disorders and malformation syndromes, Dijon, France [↑](#endnote-ref-21)
22. Inserm - University of Burgundy-Franche Comté, UMR1231 GAD, Dijon, France [↑](#endnote-ref-22)
23. Dijon University Hospital, FHU-TRANSLAD, Dijon, France [↑](#endnote-ref-23)
24. Dijon University Hospital, GIMI institute, Dijon, France [↑](#endnote-ref-24)
25. University of Burgundy-Franche Comté, Dijon Economics Laboratory, Dijon, France [↑](#endnote-ref-25)
26. University of Burgundy-Franche Comté, FHU-TRANSLAD, Dijon, France [↑](#endnote-ref-26)
27. CNAG‐CRG, Centre for Genomic Regulation (CRG), The Barcelona Institute of Science and Technology, Baldiri Reixac 4, Barcelona 08028, Spain [↑](#endnote-ref-27)
28. Universitat Pompeu Fabra (UPF), Barcelona, Spain. [↑](#endnote-ref-28)
29. EURORDIS-Rare Diseases Europe, Sant Antoni Maria Claret 167 - 08025 Barcelona, Spain [↑](#endnote-ref-29)
30. EURORDIS-Rare Diseases Europe, Plateforme Maladies Rares, 75014 Paris, France [↑](#endnote-ref-30)
31. INSERM, US14 - Orphanet, Plateforme Maladies Rares, 75014 Paris, France. [↑](#endnote-ref-31)
32. Institut National de la Santé et de la Recherche Medicale (INSERM) U1127, Paris, France. [↑](#endnote-ref-32)
33. Centre National de la Recherche Scientifique, Unité Mixte de Recherche (UMR) 7225, Paris, France. [↑](#endnote-ref-33)
34. Unité Mixte de Recherche en Santé 1127, Université Pierre et Marie Curie (Paris 06), Sorbonne Universités, Paris, France. [↑](#endnote-ref-34)
35. Institut du Cerveau -ICM, Paris, France. [↑](#endnote-ref-35)
36. Ecole Pratique des Hautes Etudes, Paris Sciences et Lettres Research University, Paris, France. [↑](#endnote-ref-36)
37. Centre de Référence de Neurogénétique, Hôpital de la Pitié-Salpêtrière, Assistance Publique-Hôpitaux de Paris (AP-HP), Paris, France. [↑](#endnote-ref-37)
38. Hôpital de la Pitié-Salpêtrière, Assistance Publique-Hôpitaux de Paris (AP-HP), Paris, France. [↑](#endnote-ref-38)
39. Sorbonne Université, Inserm, Institut de Myologie, Centre de Recherche en Myologie, F-75013 Paris, France [↑](#endnote-ref-39)
40. AP-HP, Centre de Référence de Pathologie Neuromusculaire Nord, Est, Ile-de-France, Institut de Myologie, G.H. Pitié-Salpêtrière, F-75013 Paris, France. [↑](#endnote-ref-40)
41. Institut de Myologie, Equipe Bases de données, G.H. Pitié-Salpêtrière, F-75013 Paris, France. [↑](#endnote-ref-41)
42. AP-HP, Unité Fonctionnelle de Cardiogénétique et Myogénétique Moléculaire et Cellulaire, G.H. Pitié-Salpêtrière, F-75013 Paris, France. [↑](#endnote-ref-42)
43. Department of Biology and Medical Genetics, Charles University Prague-2nd Faculty of Medicine and University Hospital Motol, Prague, Czech Republic. [↑](#endnote-ref-43)
44. Department of Paediatrics and Inherited Metabolic Disorders, First Faculty of Medicine, Charles University and General University Hospital in Prague, Prague, Czech Republic. [↑](#endnote-ref-44)
45. Department of Ophthalmology, First Faculty of Medicine, Charles University and General University Hospital in Prague, Prague, Czech Republic. [↑](#endnote-ref-45)
46. Centre for Paediatric Rheumatology and Autoinflammatory Diseases, Department of Paediatrics and Inherited Metabolic Disorders, 1st Faculty of Medicine, Charles University and General University Hospital in Prague, Czech Republic [↑](#endnote-ref-46)
47. European Bioinformatics Institute, European Molecular Biology Laboratory, Wellcome Genome Campus, Hinxton, Cambridge, United Kingdom. [↑](#endnote-ref-47)
48. Jackson Laboratory for Genomic Medicine, Farmington, CT 06032, USA. [↑](#endnote-ref-48)
49. Florence Nightingale Faculty of Nursing, Midwifery & Palliative Care, King's College, London, UK. [↑](#endnote-ref-49)
50. Wellcome Genome Campus Society and Ethics Research, Wellcome Genome Campus, UK [↑](#endnote-ref-50)
51. Genomics England, Queen Mary University of London, Dawson Hall, EC1M 6BQ, London, UK. [↑](#endnote-ref-51)
52. Society and Ethics Research, Connecting Science, Wellcome Genome Campus,

    Hinxton, UK [↑](#endnote-ref-52)
53. MRC Centre for Neuromuscular Diseases and National Hospital for Neurology and Neurosurgery, UCL Queen Square Institute of Neurology, London, UK. [↑](#endnote-ref-53)
54. Department of Neuromuscular Diseases, UCL Queen Square Institute of Neurology, London, UK. [↑](#endnote-ref-54)
55. Department of Clinical and Movement Neurosciences, UCL Queen Square Institute of Neurology, University College London, WC1N 3BG, UK. [↑](#endnote-ref-55)
56. Department of Clinical and Experimental Epilepsy, UCL Queen Square Institute of Neurology, London, UK. [↑](#endnote-ref-56)
57. Dementia Research Centre, Department of Neurodegenerative Disease, UCL Queen Square Institute of Neurology, London, UK. [↑](#endnote-ref-57)
58. Dubowitz Neuromuscular Centre, UCL Great Ormond Street Hospital, London, UK. [↑](#endnote-ref-58)
59. NIHR Great Ormond Street Hospital Biomedical Research Centre, London, United Kingdom. [↑](#endnote-ref-59)
60. Peripheral Neuropathy Research Group, University of Antwerp, Antwerp, Belgium. [↑](#endnote-ref-60)
61. Laboratory of Neuromuscular Pathology, Institute Born-Bunge, University of Antwerp, Antwerpen, Belgium [↑](#endnote-ref-61)
62. Translational Neurosciences, Faculty of Medicine and Health Sciences, University of Antwerp, Belgium [↑](#endnote-ref-62)
63. Neuromuscular Reference Centre, Department of Neurology, Antwerp University Hospital, Antwerpen, Belgium [↑](#endnote-ref-63)
64. Translational Neuroscience group, University of Antwerp, Belgium [↑](#endnote-ref-64)
65. VIB-CMN, Applied and Translational Neurogenomics Group [↑](#endnote-ref-65)
66. Dipartimento di Medicina di Precisione, Università degli Studi della Campania "Luigi Vanvitelli," Napoli, Italy. [↑](#endnote-ref-66)
67. Telethon Institute of Genetics and Medicine, Pozzuoli, Italy. [↑](#endnote-ref-67)
68. Istituto di Scienze Applicate e Sistemi Intelligenti "E.Caianiello" - ISASI -CNR [↑](#endnote-ref-68)
69. Unit of Medical Genetics, Department of Medical Sciences, University of Ferrara, Italy. [↑](#endnote-ref-69)
70. Institute of Human Genetics, Medical Faculty, University of Bonn, Bonn, Germany. [↑](#endnote-ref-70)
71. Center for Hereditary Tumor Syndromes, University Hospital Bonn, Bonn, Germany. [↑](#endnote-ref-71)
72. i3S - Instituto de Investigação e Inovação em Saúde, Universidade do Porto, Portugal. [↑](#endnote-ref-72)
73. IPATIMUP - Institute of Molecular Pathology and Immunology of the University of Porto, Portugal [↑](#endnote-ref-73)
74. Departament of Pathology, Faculty of Medicine, University of Porto, Portugal. [↑](#endnote-ref-74)
75. Doctoral Programme in Biomedicine, Faculty of Medicine, University of Porto, Portugal. [↑](#endnote-ref-75)
76. Doctoral Programme in BiotechHealth, School of Medicine and Biomedical Sciences, University of Porto, Portugal [↑](#endnote-ref-76)
77. Doctoral Programme in Computer Science, Faculty of Sciences, University of Porto, Portugal [↑](#endnote-ref-77)
78. Departament of Pathology, Faculty of Medicine, University of Porto, Portugal. [↑](#endnote-ref-78)
79. Departament of Genetics, Faculty of Medicine, University of Porto, Portugal. [↑](#endnote-ref-79)
80. CHUSJ, Centro Hospitalar e Universitário de São João, Porto, Portugal [↑](#endnote-ref-80)
81. Faculty of Sciences, University of Porto, Portugal [↑](#endnote-ref-81)
82. Department of Genetics, Genomics Coordination Center, University Medical Center Groningen, University of Groningen, Groningen, The Netherlands. [↑](#endnote-ref-82)
83. Department of Genetics, University Medical Center Groningen, University of Groningen, Groningen, The Netherlands. [↑](#endnote-ref-83)
84. ERN-GENTURIS [↑](#endnote-ref-84)
85. ERN-RITA: European Reference Network for Immunodeficiency, Autoinflammatory, Autimmune and Paediatric Rheumatic diseases, Utrecht, Netherlands [↑](#endnote-ref-85)
86. Ada Health GmbH, Karl-Liebknecht-Str. 1, 10178 Berlin, Germany. [↑](#endnote-ref-86)
87. College of Health, Well-being and Life-Sciences, Sheffield Hallam University, Sheffield, UK. [↑](#endnote-ref-87)
88. Florence Nightingale Faculty of Nursing and Midwifery, King's College, London, UK. [↑](#endnote-ref-88)
89. Dept of Genetics, Assistance Publique-Hôpitaux de Paris - Université de Paris, Robert DEBRE University Hospital, 48 bd SERURIER, Paris, France [↑](#endnote-ref-89)
90. INSERM UMR 1141 "NeuroDiderot", Hôpital R DEBRE, Paris, France [↑](#endnote-ref-90)
91. Department of genetics, Assistance Publique-Hôpitaux de Paris - Sorbonne Université, Pitié-Salpêtrière University Hospital, 83 Boulevard de l'Hôpital, Paris, France [↑](#endnote-ref-91)
92. Reference center of rare diseases "intellectuel disability of rare causes", Paris, France [↑](#endnote-ref-92)
93. Institut du Cerveau (ICM), UMR S 1127, Inserm U1127, CNRS UMR 7225, Sorbonne Université, 75013, Paris, France [↑](#endnote-ref-93)
94. Univ. Bordeaux, MRGM INSERM U1211, CHU de Bordeaux, Service de Génétique Médicale , F-33000 Bordeaux, France [↑](#endnote-ref-94)
95. Laboratoire de Génétique Moléculaire, Service de Génétique Médicale, CHU Bordeaux – Hôpital Pellegrin, Place Amélie Raba Léon, 33076 Bordeaux Cedex, France [↑](#endnote-ref-95)
96. Institute of Rare Diseases Research, Spanish Undiagnosed Rare Diseases Cases Program (SpainUDP) & Undiagnosed Diseases Network International (UDNI), Instituto de Salud Carlos III, Madrid, Spain [↑](#endnote-ref-96)
97. Genetics and Rare Diseases Research Division, Ospedale Pediatrico Bambino Gesù, IRCCS, 00146 Rome, Italy [↑](#endnote-ref-97)
98. Med Biotech Hub and Competence Center, Department of Medical Biotechnologies, University of Siena, Italy [↑](#endnote-ref-98)
99. Medical Genetics, University of Siena, Italy [↑](#endnote-ref-99)
100. Genetica Medica, Azienda Ospedaliero-Universitaria Senese, Italy [↑](#endnote-ref-100)
101. Institute of Genomic Medicine and Rare Diseases, Semmelweis University, Budapest, Hungary [↑](#endnote-ref-101)
102. Clinical institute of genomic medicine, University medical centre Ljubljana, Slovenia [↑](#endnote-ref-102)
103. Institute of Systems Motor Science, University of Lübeck, Lübeck, Germany. [↑](#endnote-ref-103)
104. Institute of Neurogenetics, University of Lübeck, Lübeck, Germany. [↑](#endnote-ref-104)
105. Department of Neurology, University Hospital Schleswig Holstein, Ratzeburger Allee 160, 23538, Lübeck, Germany [↑](#endnote-ref-105)
106. Pediatric Neurology Research Group, Vall d’Hebron Research Institute, Universitat Autònoma de Barcelona, Barcelona, Spain [↑](#endnote-ref-106)
107. Institut de Neurociències, Universitat Autònoma de Barcelona, Barcelona, Spain [↑](#endnote-ref-107)
108. Diagnostic Immunology Research Group, Vall d’Hebron Research Institute (VHIR), Barcelona, Spain [↑](#endnote-ref-108)
109. Immunology Division, Genetics Department. Vall d'Hebron University Hospital (HUVH), Barcelona, Spain [↑](#endnote-ref-109)
110. Infection in Immunocompromised Pediatric Patients Research Group, Vall d’Hebron Research Institute (VHIR), Barcelona, Spain [↑](#endnote-ref-110)
111. Pediatric Infectious Diseases and Immunodeficiencies Unit, Vall d’Hebron University Hospital (HUVH),Barcelona, Spain [↑](#endnote-ref-111)
112. Immunology Unit. Department of Cell Biology, Physiology and Immunology. Autonomous University of Barcelona (UAB), Bellaterra, Spain [↑](#endnote-ref-112)
113. Neuromuscular Disorders Unit , Department of Pediatric Neurology. Hospital Sant Joan de Déu, Barcelona, Spain [↑](#endnote-ref-113)
114. Department of Neuropediatrics and Muscle Disorders, Medical Center, Faculty of Medicine, University of Freiburg, Freiburg, Germany. [↑](#endnote-ref-114)
115. Centro Nacional de Análisis Genómico (CNAG-CRG), Center for Genomic Regulation, Barcelona Institute of Science and Technology (BIST), Barcelona, Spain. [↑](#endnote-ref-115)
116. Children's Hospital of Eastern Ontario Research Institute, University of Ottawa, Ottawa, ON, Canada. [↑](#endnote-ref-116)
117. Institute for Immunodeficiency, Center for Chronic Immunodeficiency (CCI), Medical Center, Faculty of Medicine, Albert-Ludwigs-University of Freiburg, Germany [↑](#endnote-ref-117)
118. Clinic of Rheumatology and Clinical Immunology, Center for Chronic Immunodeficiency (CCI), Medical Center, Faculty of Medicine, Albert-Ludwigs-University of Freiburg, Germany [↑](#endnote-ref-118)
119. DZIF – German Center for Infection Research, Satellite Center Freiburg, Germany [↑](#endnote-ref-119)
120. CIBSS – Centre for Integrative Biological Signalling Studies, Albert-Ludwigs University, Freiburg, Germany [↑](#endnote-ref-120)
121. RESIST – Cluster of Excellence 2155 to Hanover Medical School, Satellite Center Freiburg, Germany [↑](#endnote-ref-121)
122. Nuffield Department of Clinical Neurosciences, University of Oxford, UK [↑](#endnote-ref-122)
123. Folkhälsan Research Centre and Medicum, University of Helsinki, Helsinki, Finland [↑](#endnote-ref-123)
124. Tampere Neuromuscular Center, Tampere, Finland [↑](#endnote-ref-124)
125. Vasa Central Hospital, Vaasa, Finland [↑](#endnote-ref-125)
126. Department of Clinical Neurosciences, University of Cambridge, Cambridge, UK [↑](#endnote-ref-126)
127. Medical Research Council Mitochondrial Biology Unit, University of Cambridge, Cambridge, UK [↑](#endnote-ref-127)
128. Department of Paediatrics, University of Cambridge, Cambridge, UK [↑](#endnote-ref-128)
129. East Anglian Medical Genetics Service, Cambridge University Hospitals NHS Foundation Trust, Cambridge, UK [↑](#endnote-ref-129)
130. Bellvitge Biomedical Research Institute (IDIBELL), Barcelona, Spain [↑](#endnote-ref-130)
131. Medizinische Klinik und Poliklinik IV – Campus Innenstadt, Klinikum der Universität München, Munich, Germany and MGZ - Medical Genetics Center, Munich, Germany [↑](#endnote-ref-131)
132. Medical Genetics Center (MGZ), Munich, Germany [↑](#endnote-ref-132)
133. Institute for Clinical Genetics, Faculty of Medicine Carl Gustav Carus, Technical University Dresden, Dresden, Germany [↑](#endnote-ref-133)
134. Center for Personalized Oncology, University Hospital Carl Gustav Carus, Technical University Dresden, Dresden, Germany [↑](#endnote-ref-134)
135. Koç Universıty,School of Medicine, Translational Medicine Research Center, KUTTAM-NDAL Istanbul Turkey [↑](#endnote-ref-135)
136. Dpt. of Neurology, Ghent University Hospital [↑](#endnote-ref-136)
137. UD-PrOZA, Ghent University Hospital [↑](#endnote-ref-137)
138. Center for Medical Genetics, Ghent, University Hospital [↑](#endnote-ref-138)
139. Department of Biomolecular Medicine, Faculty of Medicine and Health Sciences, Ghent University [↑](#endnote-ref-139)
140. Neuroscience Department, Children's Hospital A. Meyer-University of Florence, 50139, Florence, Italy. [↑](#endnote-ref-140)
141. Department of Clinical and Experimental Epilepsy, UCL Queen Square Institute of Neurology, and Chalfont Centre for Epilepsy, Gerrard Cross, UK. [↑](#endnote-ref-141)
142. Department of Neurology, University Hospitals Leuven, Leuven, Belgium [↑](#endnote-ref-142)
143. Laboratory for Muscle Diseases and Neuropathies, Department of Neurosciences, and Leuven Brain Institute (LBI), KU Leuven - University of Leuven, Leuven, Belgium [↑](#endnote-ref-143)
144. Department of Clinical Genetics, Leiden University Medical Center, Leiden, The Netherlands [↑](#endnote-ref-144)
145. Ludwig Boltzmann Institute for Rare and Undiagnosed Diseases, Vienna, Austria [↑](#endnote-ref-145)
146. St. Anna Children’s Cancer Research Institute (CCRI), Vienna, Austria [↑](#endnote-ref-146)
147. CeMM Research Center for Molecular Medicine of the Austrian Academy of Sciences, Vienna, Austria [↑](#endnote-ref-147)
148. Department of Pediatrics and Adolescent Medicine, Medical University of Vienna, Vienna, Austria [↑](#endnote-ref-148)
149. St. Anna Children's Hospital, Department of Pediatrics and Adolescent Medicine, Medical University of Vienna, Vienna, Austria [↑](#endnote-ref-149)
150. Centre de Génétique Humaine, Institut de Pathologie et de Génétique, Gosselies, Belgium [↑](#endnote-ref-150)
151. Département de Médecine, Université de namur (Unamur), Namur, Belgique [↑](#endnote-ref-151)
152. Department of Neurology, Klinikum rechts der Isar, Technical University Munich, Munich, Germany [↑](#endnote-ref-152)
153. Neurology / Neurogenetics Laboratory University of Crete, Heraklion, Crete, Greece [↑](#endnote-ref-153)
154. Aristotle University of Thessaloniki, Thessaloniki, Greece [↑](#endnote-ref-154)
155. 1st Department of Neurology, Aristotle University of Thessaloniki, University General Hospital of Thessaloniki, AHEPA, Thessaloniki, Greece [↑](#endnote-ref-155)
156. Neurochemistry and Biomarker Unit, 1st Department of Neurology, School of Medicine, National and Kapodistrian University of Athens, Eginition Hospital, Athens, Greece [↑](#endnote-ref-156)
157. Pediatric Neurology and Muscular Disease Unit, IRCCS Istituto Giannina Gaslini, Genoa, Italy [↑](#endnote-ref-157)
158. Department of Neurosciences, Rehabilitation, Ophthalmology, Genetics, Maternal and Child Health, University of Genoa, Genoa, Italy. [↑](#endnote-ref-158)
159. Unit of Medical Genetics, IRCCS Istituto Giannina Gaslini, Genoa, Italy [↑](#endnote-ref-159)
160. Clinical Bioinformatics, IRCCS Istituto Giannina Gaslini, Genoa, Italy [↑](#endnote-ref-160)
161. Pediatric Neurology Department, Saint-Luc University Hospital, Université Catholique de Louvain, Brussels, Belgium [↑](#endnote-ref-161)
162. Neurology Department, Erasme Hospital, Université Libre de Bruxelles , Bruxelles, Belgium [↑](#endnote-ref-162)
163. Institute of Pathology and Genetics, Charleroi, Belgium [↑](#endnote-ref-163)
164. Human Genetics Department, Saint-Luc University Hospital, Université Catholique de Louvain, Brussels, Belgium [↑](#endnote-ref-164)
165. Institute of Interdisciplinary Research in Human and Molecular Biology, Human Genetics, IRIBHM, Université Libre de Bruxelles, Brussels, Belgium [↑](#endnote-ref-165)
166. Institute of Human Genetics, University Hospital Essen, University Duisburg-Essen, Essen, Germany [↑](#endnote-ref-166)
167. Institut du Cerveau et de la Moelle épinière (ICM), Sorbonne Université, UMR S 1127, Inserm U1127, CNRS UMR 7225, F-75013 Paris, France [↑](#endnote-ref-167)
168. Department of Pediatric Neurology, Developmental Neurology and Social Pediatrics, Children's Hospital University of Essen, Essen, Germany [↑](#endnote-ref-168)
169. Children's Hospital of Eastern Ontario Research Institute, University of Ottawa, Ottawa, Canada [↑](#endnote-ref-169)
170. Department of Neurology, Heimer Institute for Muscle Research, University Hospital Bergmannsheil, Ruhr-University Bochum, 44789 Bochum, Germany [↑](#endnote-ref-170)
171. Luxembourg Centre for Systems Biomedicine, University of Luxembourg, Esch-sur-Alzette, Luxembourg [↑](#endnote-ref-171)
